# Diversity under a magnifier lens: the case of Typhlotanaidae (Crustacea: Tanaidacea) in the N Atlantic

**DOI:** 10.1038/s41598-023-33616-y

**Published:** 2023-07-05

**Authors:** Marta Gellert, Magdalena Błażewicz, Tomasz Mamos, Graham J. Bird

**Affiliations:** 1 Department of Invertebrate Zoology and Hydrobiology, University of Lodz, Lodz, Poland; 2 Independent Researcher, Waikanae, New Zealand

**Keywords:** Biodiversity, Biooceanography, Marine biology

## Abstract

Research focusing on ‘stout-bodied’ typhlotanaids collected from several sites around Iceland and adjacent N Atlantic region has resulted in the description of 15 species new to science, as well as the creation of eight new genera. *Typhlotanais eximius* Hansen, 1913 is redescribed and transferred to a new genus, while *Typhlotanais crassus* and *Peraeospinosus adipatus* are transferred to the genus *Larsenotanais*. The morphological and the molecular data were combined to consolidate and confirm the validity of the results obtained from both approaches. The polyphyletic nature of the Typhlotanaidae and its serious of its taxonomic diversity are emphasized, although molecular analysis reveals that the ‘stout-bodied’ Typhlotanaidae form monophyletic clade. Depth and temperature are identified as the main environmental parameters determining the distribution of this group of Typhlotanaidae. Several species are clearly associated with the shelf and upper bathyal of Iceland. The Greenland-Iceland-Faroe Ridge is shown to be a distinct zoogeographical barrier for typhlotanaids inhabiting the deeper slope and abyssal regions around Iceland.

## Introduction

The oceanic floor below the continental shelf is the largest and scarcely known ecosystem and is inhabited by a high number of rare species many of which remain unknown to science^1,2^. The consequences of climate change, clearly visible and severe on land, also affect the fragile and unrecognized organisms living in the deepest part of the ocean^3–5^. Evolving under specific environmental conditions the fauna is potentially vulnerable to dynamic environmental changes that can disrupt their biological and physiological processes and reproductive cycles, leading to changes in population structure, shifts in ecosystem functioning or even extinction^6–9^. Besides, the deep sea is designated as a territory for large-scale economic operations that could inducing extreme ecosystem transformations that are difficult to assess for scale and direction^10^. For this reason, understanding the biodiversity, in the sites directly affected by human activity or consequences caused by climate change is a priority for current research.

The N Atlantic is an important sink for global ocean waters and the origin of the thermohaline circulation^11–13^. The dynamic warming observed in this part of the world (Atlantification) is of the highest concern due to its multifaceted threat to the climate-sensitive N Atlantic fauna^11,14–18^ and has become a natural observatory for climate change^19–21^. The knowledge about which factors structure or modify benthic communities in the N Atlantic is essential for assessing *whether* and *how* the changing ecosystem affects on its sensitive fauna. The region has many complexities, and the Greenland-Icelandic-Faroe Ridge aligned perpendicular to Mid-Atlantic Ridge, is a main topographical feature separating cold Arctic waters from warmer Atlantic waters^22,23^ and acts as a natural biological barrier^12,13,23^. It also limits biological migrations from south to north in the N Atlantic and is an ideal natural experimental zone for observing the zoogeographic shifts of fauna caused by environmental factors. For large or commercially important species (e.g. fish), the diversity and zoogeographical ranges are considered well understood when compared to smaller animals of lesser, or as yet unknown, commercial potential. Nevertheless, these smallest species are proving to be a highly diverse component of deep-sea ecosystems whose correct identification allows for reliable biological analyses, and are important object of taxonomic, phylogenetic and zoogeographic research^24–26^.

Among these small benthic organisms is the Typhlotanaidae Sieg, 1984— a diverse and poorly known family of the peracarid Tanaidacea. The family is represented by small and specialized taxa with a three-article antennule and a ‘clinging-type’ of pereopods 4–6. These appendages have a robust basis, shorter dactylus-unguis (claw), and also a ‘clinging apparatus’ located on the carpus^27,28^. The last is composed of complex structures such as prickly tubercles, microtrichia or serrated spines, and is assumed to help motility of the animal inside the self-constructed housing-tube. Typhlotanaids are believed to be unselective detritivores^29^ and also hosts for tantulocarids^30^ and endoparasitic nematodes^31^. As with other tanaids, they have limited dispersal abilities because of their brooding behaviour and demersal juvenile stages^32,33^ and so are considered an ideal model for zoogeographic studies and assessments of the effects of anthropogenic impact in deep-sea ecosystems^32,34^.

The N Atlantic is known for a high diversity of Typhlotanaidae, derived from a series of studies going back to pioneering work of these peracarids in the northern European seas by Lilljeborg^35^, and Sars 1879, 1882^36,37^, and off Iceland and Jan Mayen Island by Hansen 1913. Hansen was also the first to demonstrate the high diversity of Typhlotanaidae, and Tanaidacea in general, below the continental shelf. From 78 recorded tanaidaceans (27 species new to science) a quarter (19 species) were classified as typhlotanaids and six of them were new to science^38^. For this reason, the N Atlantic is relatively well-studied in the context of typhlotanaid taxonomy when compared to other areas of the world.

Currently, the Typhlotanaidae is represented by 100 nominal species distributed in 15 genera^39–42^ contributing about 5% of all tanaidacean taxa and 14.5% of all known paratanaoid species, making them the most diverse family of paratanaoids. While establishing the family Sieg (1986)^43^ stated that ‘Of these, *Typhlotanais* is still to be regarded as very heterogeneous, so that a further split is to be expected in the future’. With this observation he erected three genera (*Peraeospinosus* Sieg, 1986, *Meromonakantha* Sieg, 1986, and *Typhlotanoides* Sieg, 1983) to accommodate most morphologically distinct typhlotanaids so making the first step in further exploration of typhlotanaid relationships^27,28,44–49^. Typhlotanaids were provisionally divided into ‘slender-bodied’ and ‘stout-bodied’ with body length ≥ 8.0 L:W and ≤ 6.0 L:W, respectively^27,28^. The ‘stout-bodied’ are currently represented by 21 species from four genera: *Antiplotanais* Bamber, 2008*, Larsenotanais* Błażewicz-Paszkowycz, 2007*, Typhlotanais* Sars, 1882 and *Typhlotanoides* Sieg, 1983, and only two of them (*Typhlotanais cornutus* and *Typhlotanais inermis*) are recorded in the N Atlantic.

**Figure 1:**
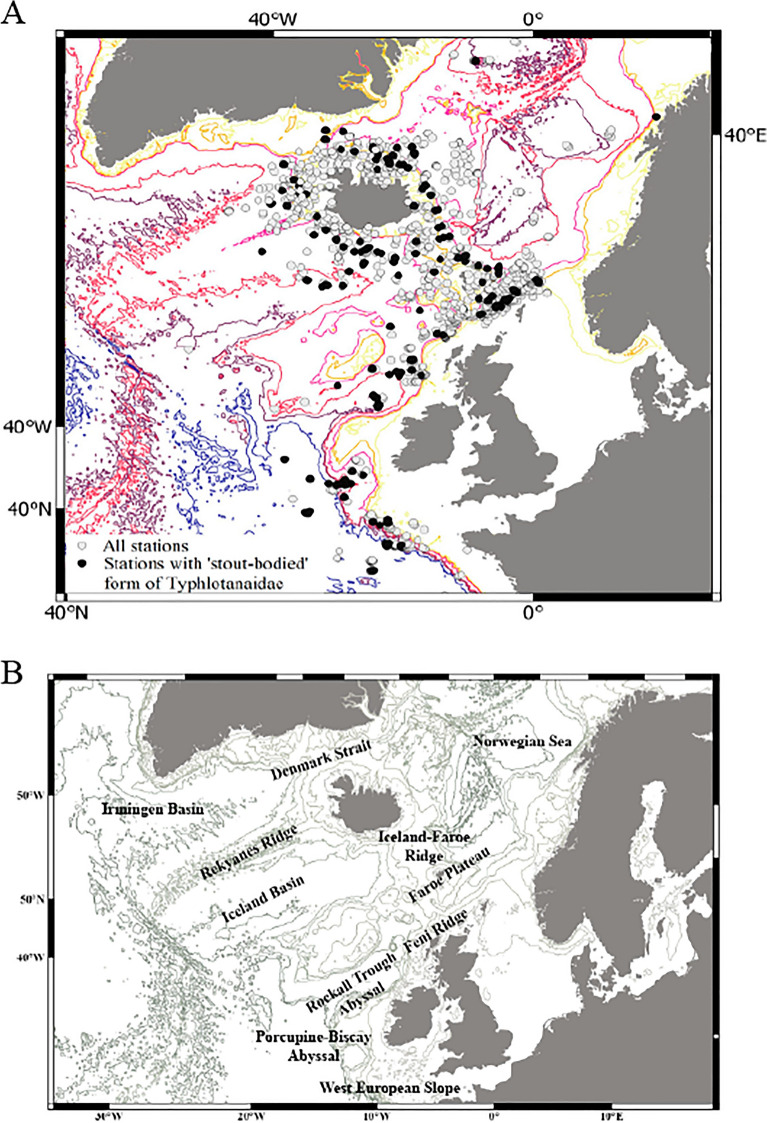
(**A**) The distribution of AFEN 1996 and 1998, BIOFAR, BIOGAS III and VI, BIOICE, Chain 106, Discovery, DTI 2000, IceAGE I and II, InCAL, POLYGAS, NORBI, Sarsia, SMBA, and Thalassa 71 and 73 stations analysed in the present study. (**B**) Marine regions around Iceland (QGIS 3.28 software https://www.qgis.org/pl/site/).

In this research we focused on a rich collection of Typhlotanaidae represented by ‘stout-bodied’ form sampled from the N Atlantic during 18 international programmes and scientific cruises exploring the oceanic floor surrounding Iceland and off the NE Atlantic coasts (e.g. AFEN 1996 and 1998, BIOFAR, BIOGAS III and VI, BIOICE, Chain 106, Discovery, DTI 2000, IceAGE I and II, InCAL, POLYGAS, NORBI, Sarsia, SMBA, and Thalassa 71 and 73) (Table S1, Fig. 1). Because the collections were made before the ground-breaking use of genetic methods for species identification^50^, most of the oldest collection material was fixed with formaldehyde, limiting the use of molecular methods in our study. Nevertheless, even with a limited genetic dataset applicable to the most recent collections, we could test a hypothesis that the ‘stout-bodied’ and the ‘slender-bodied’ forms comprise separate natural evolutionary lineages. Moreover, combining genetic results with meticulous examination of morphology, allows reliable identification and description of 15 new species and erection of eight new genera, which are presented in this paper. In addition to the morphological and molecular analysis, we have aimed to analyse the environmental parameters which shape the distribution of the studied species and their communities. The distribution of the studies species have been examined more thoroughly in relation to two environmental factors, *e*.*g*. depth and temperature, as this have been reported to significantly influence distribution of benthic peracarids in the N Atlantic^51^.

## Results

**Figure 2:**
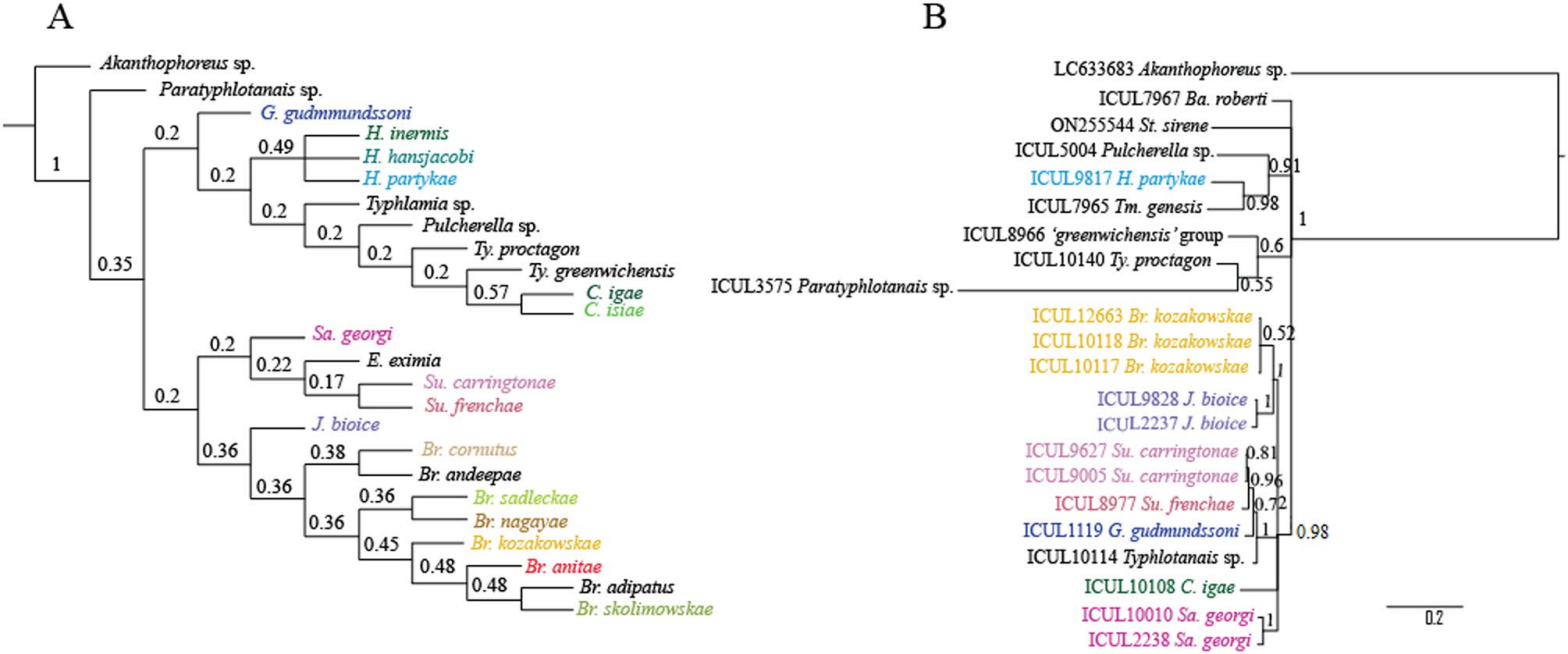
The phylogenetic relationships of the Typhlotanaidae (**A**) morphological tree; (**B**) Molecular tree reconstructed using the 18S sequences. The percentage of trees in which the associated taxa clustered together (bootstrap support) is shown next to the branches.

In our research we used three approaches: morphological, molecular, and environmental to delineate species in the collection of ‘stout-bodied’ typhlotanaids represented by 1919 individuals. Each of these approaches was applied separately, and the results were compared with each other to perform species delimitation (Fig. 2A, B).

### Molecular approach: Phylogenetic analysis

A total of 20 different 18S haplotypes and 26 H3 haplotypes are newly obtained from the University of Lodz Tanaidacea collection (GenBank accession numbers: OQ034236–OQ034255 for 18S and OQ107187–OQ107212 for H3; see Table 1). The 18S phylogenetic tree resulting from the Maximum Likelihood and Bayesian analysis shows relatively high support values for most clades (Fig. 2B). Typhlotanaid species are grouped into seven well-supported clades namely, (1) “*Sarsotanais”* (Italicized names are uses for nomina later described formally below); (2) “*Gudmundotanais”*; (3) “*Stuttotanais”*; 4) “*Jurundurella”*; (5) *Pulcherella*; (6) *Typhlamia*; and (7) “*Hansenotanais”*. In addition, *Baratheonus* and *Starkus* form separate clades. The first clade is characterized by pereonites with straight margins and large prickly tubercles on the carpus of pereopods 4–6 (longer than half of the carpus length), while the others are supported by presence of ventrodistal spines in the pereopods 2–3 carpus and propodus, and biarticulate uropodal exopod and endopod. *Pulcherella*, *Typhlamia* and “*Hansenotanais”* group together (p-dist *Pulcherella*—*Typhlamia* = 0.068 ± 0.009, *Typhlamia*—*“Hansenotanais”* = 0.058 ± 0.009, and *Pulcherella*—*“Hansenotanais”* = 0.063 ± 0.009) (Tab. S2) sharing the pereopod-1 carpus with at least one long distal seta, and uropods with a biarticulate endopod and uniarticulate exopod. There is good support (0.98) for the clade of “*Hansenotanais”* and *Typhlamia* which are characterized by presence of two cusps instead of prickly tubercles on the pereopods 4–6 carpus. A relatively low support (0.6) was calculated for the clade composed with *Ty. proctagon,* members of ‘greenwichensis’ group and *Paratyphlotanais*, which shared features such as the cheliped carpus with a ‘third’ seta and antennule article-3 with an apical spur. The clade with two genera, e.g. “*Brevitanais”* and “*Jurundurella”* (p-dist = 0.029 ± 0.006), groups the taxa with a spine on pereopods 2–3 carpus and propodus, a long dorsodistal seta on the pereopods 2–3 propodus, and a bifurcate unguis in pereopods 4–6. The next clade consists of “*Stuttotanais”, “Gudmundotanais”* and *Typhlotanais* sp. (support 1.0; p-dist “*Stuttotanais”*—“*Gudmundotanais”* = 0.008 ± 0.004, “*Stuttotanais”*—*Typhlotanais* sp. = 0.043 ± 0.004, and “*Gudmundotanais”*—*Typhlotanais* sp. = 0.046 ± 0.005). It groups the taxa with a basal seta shorter than maxilliped endites, and uropod with biarticulate endopod and uniarticulate exopod. The genus “*Caesatanais”* that has a long ventrodistal seta in pereopods 2–3 make a speared node, similar to “*Sarsotanais”* characterized by numerous spiniform nodules on the pereopod-3 merus.

**Table 1:** Voucher codes for the museum specimens and GenBank accession numbers for the histone H3 and 18S rDNA sequences used to build the molecular trees.

Species	Voucher	Histone H3 (GenBank accession numbers)	18S rDNA (GenBank accession numbers)	Reference
*Akanthophoreus gracilis*	–	MN382178		GenBank database
*Akanthophoreus* sp.	–		LC633683	Kakui & Hiruta (2022)
*Baratheonus roberti*	ICUL7967	OQ107202	OQ034249	Present study
*Brevitanais kozakowskae* n. sp.	ICUL9813	OQ107208		Present study
*Brevitanais kozakowskae* n. sp.	ICUL9832	OQ107211		Present study
*Brevitanais kozakowskae* n. sp.	ICUL9894	OQ107212		Present study
*Brevitanais kozakowskae* n. sp.	ICUL10117	OQ107188	OQ034239	Present study
*Brevitanais kozakowskae* n. sp.	ICUL10118	OQ107189	OQ034240	Present study
*Brevitanais kozakowskae* n. sp.	ICUL10121	OQ107190		Present study
*Brevitanais kozakowskae* n. sp.	ICUL10122	OQ107191		Present study
*Brevitanais kozakowskae* n. sp.	ICUL12663	OQ107193	OQ034243	Present study
*Caesatanais igae* n. sp.	ICUL10108	OQ107187	OQ034237	Present study
*Gudmundotanais gudmundssoni* n. sp.	ICUL1119		OQ034241	Present study
*Hansenotanais partykae* n. sp.	ICUL9817	OQ107209	OQ034254	Present study
*Jurundurella bioice* n. sp.	ICUL2237		OQ034244	Present study
*Jurundurella bioice* n. sp.	ICUL9828	OQ107210	OQ034255	Present study
*Meromonakantha* sp.	ICUL3590	OQ107198		Present study
*Obesutanais* sp.	ICUL11279	OQ107192		Present study
*Paratyphlotanais* sp.	ICUL915	OQ107207		Present study
*Paratyphlotanais* sp.	ICUL3575		OQ034246	Present study
*Pulcherella* sp.	ICUL5004		OQ034247	Present study
*Sarsotanais georgi* n. sp.	ICUL2238		OQ034245	Present study
*Sarsotanais georgi* n. sp.	ICUL10010		OQ034236	Present study
*Starkus sirene*	ICUL7900	OQ107200	ON255544	Present study, Gellert et al.^28^
*Stuttotanais carringtonae* n. sp.	ICUL9005	OQ107206	OQ034252	Present study
*Stuttotanais carringtonae* n. sp.	ICUL9627		OQ034253	Present study
*Stuttotanais frenchae* n. sp.	ICUL8977	OQ107205	OQ034251	Present study
*Torquella* sp.	ICUL15585	OQ107194		Present study
*Typhlamia genesis*	ICUL7965	OQ107201	OQ034248	Present study
*Typhlotanais 'greenwichensis'* group	ICUL8966	OQ107204	OQ034250	Present study
*Typhlotanais ‘trispinosus’* group	ICUL1665	OQ107195		Present study
*Typhlotanais plicatus*	ICUL8952	OQ107203		Present study
*Typhlotanais proctagon*	ICUL10140		OQ034242	Present study
*Typhlotanais* sp.	ICUL3566	OQ107197		Present study
*Typhlotanais* sp.	ICUL10114		OQ034238	Present study
*Typhlotanais 'variabilis'* group	ICUL1740	OQ107196		Present study
*Typhlotanoides* sp.	ICUL5356	OQ107199		Present study

The genetic clustering of 18S sequences in the ML and Bayesian trees corresponds to the morphological identification of the taxa. Pairwise genetic p-distances between 18S sequences ranged between 0 and 22.6% (Table S2), although intraspecific genetic variability was quite low, as would be expected given the limited sample size for each species. *Typhlamia* and *Hansenotanais* formed a well-supported monophyletic clade. The clade grouping all ‘stout-bodied’ typhlotanaids with a ‘third’ seta on the cheliped carpus ventral margin, and presence of clinging apparatus on the last three pairs of pereopods has bootstrap support of 100%.

The largest genetic divergences were observed when comparing *Ty. proctagon* Tattersall, 1904 with *Starkus* sp. (p-dist = 0.105 ± 0.012) and “*H. partykae”* with *Starkus* sp. (p-dist = 0.102 ± 0.012). For comparison, the smallest genetic distances were found between “*Su. carringtonae”* and *Typhlotanais* sp. (p-dist = 0.011 ± 0.004) or “*G. gudmundssoni”* and *Typhlotanais* sp. (p-dist = 0.014 ± 0.005). As with the Maximum Likelihood tree, genetic distances suggest that 'stout-bodied' forms of typhlotanaids are monophyletic.

The H3 phylogenetic tree resulting from the Bayesian analysis showed relatively low support values for most clades. Typhlotanaid species grouped into only three well-supported clades namely, (1) “*Caesatanais”*; (2) *Typhlotanais* sp.; (2) *Typhlamia* sp.; (3) “*Hansenotanais”*; (4) the ‘variabilis’ group, and (5) *Typhlotanais plicatus* (Fig. S1).

### Phylogenetic approach: Morphological analysis

As a result of the phylogenetic analysis, three parsimony equal trees with a length of 326 steps were obtained. A strict consensus tree was calculated from the trees obtained (Fig. 2A). The consistency index (CI) and retention index (RI) had values of 0.53 and 0.39, respectively.

In the obtained tree, the Typhlotanaidae species formed into two large clades. The first clade is composed of several 'stout-bodied' and 'slender-bodied' genera and spits into several smaller clades. In the first clade “*Gudmundotanais”* is most distinct taxon and makes a separate lineage supported by the following synapomorphies: antennule article-1 2.6 L:W, antenna article-2 without seta, pereopod-1 basis 0.6× other articles, pereopod-1 merus 2.1 L:W, pereopods 2–3 propodus with long dorsodistal seta (longer than dactylus) and uropod exopod 0.9× endopod. The more internal clade includes three species of the genus “*Hansenotanais”* (Bremer support 0.49) that share a long ventrodistal seta on the carpus (longer than half of the propodus) in pereopods 2–3. In three equal parsimony trees, these species swap places within this subclade. The most internal clade forms a cascade of branches composed of 'slender-bodied' typhlotanaids (*Typhlamia* sp., *Pulcherella* sp., *Ty. proctagon*, and *Ty. greenwichensis*) as well as two ‘stout-bodied’ species of the genus “*Caesatanais”* (Bremer 0.2)*.* Their common feature is a long ventrodistal seta on the pereopod-1propodus.

The other main clade consists of exclusively 'stout-bodied' typhlotanaids and is further split into two subclades. The first subclade is made by: “*Sarsotanais”*, “*Egregiella”* and “*Stuttotanais”* (Bremer 0.2) and is supported by: antenna article-2 with a seta, calcified microtrichia on the merus of pereopods 2–3, a carpus with short ventrodistal seta in pereopods 2–3, and the propodus with short dorsodistal seta in pereopods 4–6. The other subclade includes “*Jurundurella”* and “*Brevitanais”* (Bremer 0.36). “*Jurundurella”* is morphologically distinct taxon characterized by straight margins of the pereonites and long dorsodistal seta (longer than dactylus) in pereopods 2–3 propodus. The more internal clade is formed by eight species of “*Brevitanais”* (Bremer 0.36) with antenna article-2 1.6 L:W and long dorsodistal seta pereopods 4–6 propodus. The “*Brevitanais”* is further separated by articulation of the uropods and apical spur in antennule article-3. Therefore “*Br. andeepae”* and “*Br. cornutus”* as the only *Brevitanais* taxa with biarticulate uropod rami and antennal spur are grouped together (Bremer 0.38). The other “*Brevitanais”* taxa are further split into two clades. The first, with “*Br. sadleckae”* and “*Br. nagayae”* (support 0.36), groups taxa with several characters, e.g. antennule article-1 3.2 L:W, antenna article-2 1.8 L:W, antenna article-3 without seta, pereopod-1 basis 0.4× other articles and pereopod-1 merus 2.0 L:W. The other larger clade consists well supported clade with four species: “*Br. anitae”*, “*Br. adipatus”*, “*Br. kozakowskae”* and “*Br. skolimowskae”* (Bremer 0.45), which have medium-sized cusps in the maxilliped endites.

## Taxonomy

- **Family Typhlotanaidae Sieg, 1984**

### Diagnosis

(after Bird & Larsen, 2009^52^ see Gellert, Palero & Błażewicz, 2022^28^).

### Type genus

*Typhlotanais* G. O. Sars, 1882.

### Genera included

*Antiplotanais* Bamber, 2008; *Aremus* Segadilha, Gellert & Błażewicz, 2018; *Baratheonus* Gellert, Palero & Błażewicz, 2022; *Dimorphognathia* Sieg, 1986; *Larsenotanais* Błażewicz-Paszkowycz, 2007*; Lannisterella* Gellert, Palero & Błażewicz, 2022; *Meromonakantha* Sieg, 1986; *Obesutanais* Larsen, Błażewicz-Paszkowycz & Cunha, 2006; *Paratyphlotanais* Kudinova-Pasternak & Pasternak, 1978; *Peraeospinosus* Sieg, 1986; *Pulcherella* Błażewicz-Paszkowycz, 2007; *Starkus* Gellert, Palero & Błażewicz, 2022; *Targaryenella* Błażewicz & Segadilha, 2019; *Torquella* Błażewicz-Paszkowycz, 2007; *Typhlamia* Błażewicz-Paszkowycz, 2007; *Typhlotanais* Sars, 1882 and *Typhlotanoides* Sieg, 1983; *Brevitanais* n. gen.; *Caesatanais* n. gen.; *Egregiella* n. gen.; *Hansenotanais* n. gen.; *Gudmundotanais* n. gen.; *Jurundurella* n. gen.; *Sarsotanais* n. gen.; *Stuttotanais* n. gen.

### Remarks

Soon after it was erected, the family’s type genus—*Typhlotanais* become a repository taxon to include what are now suspected to be phylogenetically unrelated taxa. Apart from several distinct families such as paratanaids, nototanaids and pseudotanaids, almost all blind (Gk. *typhlos*/τυφλός) species with a three-article antennule and uni- or biarticulate uropod rami were regarded as a ‘typhlotanaids’. Błażewicz-Paszkowycz (2007)^27^ has stated that apart from the type species, *Typhlotanais aequiremis* (Lilljeborg, 1864), most taxa classified as *Typhlotanais* probably represent other, still undefined, genera^27,28^. She has also proposed a pragmatic classification of typhlotanaids into ‘long-bodied’ and ‘short-bodied’ forms (replaced by ‘slender-bodied’ and ‘stout-bodied’ respectively in this paper) and eight established morpho-groups^27,28^ proposing a preliminary systematic order for the typhlotanaids. Due to material limitations (many of the historical species taxa developed by Hansen (1913) were represented only by the holotype, and many taxa described by Kudinova-Pasternak are considered lost), she emphasised that proposed by her division is only provisional and requires further study using more species, based on more morphological characters and using genetic data. From all typhlotanaid taxa analysed by Błażewicz-Paszkowycz (2007), only two *Ty. cornutus* and *Ty. eximius* represented ‘stout-bodied’ forms.

### Invariant or near-invariant characters in the ‘stout-bodied’ typhlotanaid groups

- Rostrum shallow, acute.
- Antenna article-1 fused with cephalothorax.
- Labrum hood shaped, distally setulate.
- Mandible molar broad, with distal margin nodulose.
- Labium bilobed.
- Maxillule endite with eight spines.
- Maxilliped basis with distal seta.
- Maxilliped palp four-article; article-2 with three inner and one outer setae; article-3 with four inner setae; article-4 with five terminal and one subdistal setae.
- Cheliped basis separated from pereonite by short gap.
- Cheliped merus triangular/wedge-shaped with ventral seta.
- Cheliped carpus dorsal margin with dorsodistal chemosensory seta and proximal seta.
- Cheliped fixed finger with two ventral setae and three near cutting margin.
- Pereopods 1–3 walking type; pereopods 4–6 clinging type.
- Pereopods 1–3 ischium with one ventral seta; unguis simple.
- Pereopods 4–6 ischium with two ventral setae; propodus with one (P4–5) or three (P6) dorsodistal setae.
- Pleopod basal article naked.
- Uropod exopod with two unequal setae (length and thickness).

These are excluded from the following descriptions apart from where an exception is observed.

### ***Larsenotanais* Błażewicz-Paszkowycz, 2007**

#### Diagnosis

Body stout, pereonites margin rounded. Antennule article-1 short (< 4.0 L:W), inner margin with fewer than four setae. Maxilliped basis seta longer than endites, endite cusps small or medium size. Cheliped carpus long (> 2.5 L:W), carpus with long ventral seta (> 0.9× carpus W). Pereopod-1 merus L:W slender (> 3.0 L:W), carpus without long seta; pereopods 2–3 carpus ventrodistal seta short, microtrichia regular (if present); pereopods 4–6 carpus with prickly tubercles, propodus distodorsal seta short (< unguis and dactylus length), unguis simple. Uropod endopod and exopod uniarticulate. Male swimming type.

#### Type species

*Larsenotanais amabilis* Błażewicz-Paszkowycz, 2007.

#### Species included

*Larsenotanais amabilis* Błażewicz-Paszkowycz, 2007; *L. crassus* (Dojiri & Sieg, 1997) n. comb.; *L. kamchatikus* Błażewicz-Paszkowycz, 2007; *L. martini* n. sp.; *L. siegi* n. sp.; *L. tillardi* Bamber, 2014.

#### Remarks

Błażewicz-Paszkowycz (2007)^27^ established *Larsenotanais* for one species. As a result of the present study, the genus now comprises five species including the incompletely described *Typhlotanais crassus* (Dojiri & Sieg, 1997).

*Larsenotanais* is morphologically similar to the *Brevitanais* n. gen. group-1 and *Caesatanais* (see below) by having uniarticulate uropod rami, but the simple unguis on pereopods 4–6 distinguishes it from the other two groups (Table 2) of similar uropodal form. Another useful character separating *Larsenotanais* from *Brevitanais* group-1 and *Caesatanais* is the short distodorsal propodal seta in pereopods 4–5 (long in *Brevitanais* and *Caesatanais*). It should be noted that at least several typhlotanaids ‘stout-bodied’ tanaids have short distodorsal propodal seta in pereopods 4–5, however, the articulation of the uropods (Table 2) in combination with the cheliped character, or setation and ornamentation of the appendages, distinguishes *Larsenotanais*. For example, *Larsenotanais* has a relatively stout cheliped carpus of (2.4–3.1 L:W) that is clearly more slender in *Egregiella* (about 5.0 L:W), and lacks a long ventrodistal seta on pereopods 2–3 carpus that is present in *Hansenotanais*. *Larsenotanais* has only regular microtrichia on the pereopods 2–3 merus that are robust and well calcified in *Sarsotanais* and *Stuttotanais*. Furthermore, *Larsenotanais* lacks an apical spur on antennule article-3 that is present in *Gudmundotanais*, and has rounded pereonite margins, which are straight in *Jurundurella*.

**Table 2:** Characteristics of species currently assigned to 'stout-bodied' forms of Typhlotanaidae.

Genus	Species	Body	Pereonites margin	Antennule			Antennule				
				Article-1 (L:W)	Article-3 apical spur	Article-3 aesthetasc	Article-2 (L:W)	Article-2 with	Article-2 seta	Article-3 ornam	article-3 seta
*Larsenotanais*	*L. amabilis*	Compact	Rounded	2.7	Absent	Absent	n/a	Seta	Naked	Seta	Naked
	*L. crassus*	Compact	Rounded	2.8	Absent	Absent	n/a	n/a	n/a	n/a	n/a
	*L. kamchaticus*	Compact	Rounded	3.9	Absent	Absent	0.9	Seta	Short	Seta	Short
	*L. martini*	Compact	Rounded	3.4	Absent	Absent	1.9	2 setae	Robust	Robust	Long
	*L. siegi*	Compact	Rounded	2.9	Absent	Present	2.7	2 setae	Short	Naked	Naked
	*L. tillardi*	Compact	Rounded	3.5	Absent	Absent	2.1	Seta	Short	Seta	Short
*Brevitanais* gr-1	*Br. adipatus*	Slender	Rounded	3.2	Absent	Absent	1.8	Naked	Naked	Naked	Naked
	*Br. anitae*	Slender	Rounded	2.4	Present	Absent	1.0	Seta	Short	Seta	Short
	*Br. skolimowskae*	Slender	Rounded	2.7	Present	Absent	1.8	Seta	Short	Seta	Short
*Brevitanais* gr-2	*Br. kozakowskae*	Slender	Rounded	2.3	Present	Absent	1.6	Seta	Short	Seta	Short
	*Br. nagayae*	Slender	Rounded	3.0	Present	Absent	1.8	Naked	Naked	Naked	Naked
	*Br. sadleckae*	Slender	Rounded	3.3	Present	Absent	1.8	Seta	Short	Naked	Naked
*Brevitanais* gr-3	*Br. andeepae*	Slender	Rounded	2.1	Absent	Absent	1.5	Seta	Short	Seta	Short
	*Br. cornutus*	Slender	Rounded	2.3	Absent	Absent	1.5	Seta	Short	Seta	Short
*Caesarella*	*C. igae*	Compact	Straight	5.4	Absent	Present	2.3	Seta	Long	Seta	Long
	*C. isiae*	Compact	Straight	4.1	Absent	Absent	1.8	Seta	Long	Seta	Long
*Egriegiella*	*E. eximia*	Compact	Rounded	5.4	Present	Absent	1.5	Seta	Long	Seta	Long
*Gudmundotanais*	*G. gudmunssoni*	Compact	Rounded	2.6	Present	Absent	1.3	Naked	Naked	Seta	Long
*Hansenotanais*	*H. inermis*	Slender	Rounded	n/a	Present	Present	n/a	2 setae	Short	n/a	n/a
	*H. hansjacobi*	Compact	Rounded	n/a	Present	Absent	1.3	Seta	Long	Naked	Naked
	*H. partykae*	Compact	Rounded	4.1	Present	Absent	1.3	Seta	Short	Seta	Long
*Jurundurella*	*J. bioice*	Slender	Straight	2.3	Present	Absent	1.3	n/a	n/a	Seta	Short
*Sarsotanais*	*Sa. georgi*	Compact	Rounded	4.75	Absent	Absent	1.3	Seta	Robust	Naked	Naked
	*Sarsotanais* sp. A	Compact	Rounded	n/a	n/a	n/a	n/a	n/a	n/a	n/a	n/a
*Stuttotanais*	*Su. carringtonae*	Compact	Rounded	4.4	Present	Absent	1.9	Seta	Long	Seta	Long
	*Su. frenchae*	Slender	Rounded	3.7	Present	Absent	1.9	Seta	Long	Seta	Long

Species	Pereopod-2		Pereopods 2-3								
	Carpus (L:W)	Carpus setae	Carpus ventrodistal seta	Merus calcified microtrichia	Propodus dorsodistal setation	Propodus ventrodistal margin	Propodus ventrodistal margin seta/spine	Propodus dorsodistal seta (> dactylus)			
*L. amabilis*	1.7	4	Short	Absent	2 intermediate	Spine	Short	Intermediate			
*L. crassus*	1.7	n/a	n/a	Absent	2 intermediate	n/a	n/a	n/a			
*L. kamchaticus*	2.1	5	Short	Absent	2 intermediate	Spine	Short	Intermediate			
*L. martini*	1.9	n/a	Short	Absent	2 intermediate	Seta	Short	Intermediate			
*L. siegi*	2.4	5	Short	Absent	2 intermediate	Spine	Short	Intermediate			
*L. tillardi*	2.1	4	Short	Absent	2 intermediate	Spine	Short	Intermediate			
*Br. adipatus*	n/a	n/a	Short	Absent	2 intermediate	n/a	n/a	n/a			
*Br. anitae*	1.8	2	Short	Absent	2 intermediate	spine	short	Intermediate			
*Br. skolimowskae*	1.1	5	Short	Absent	2 intermediate	spine	short	Intermediate			
*Br. kozakowskae*	1.6	3	Short	Absent	2 intermediate	spine	short	Intermediate			
*Br. nagayae*	1.2	2	Short	Absent	2 intermediate	Seta	Long	Intermediate			
*Br. sadleckae*	1.6	3	Short	Absent	2 intermediate	Spine	Short	Intermediate			
*Br. andeepae*	1.4	3	Short	Absent	2 intermediate	Spine	Short	Intermediate			
*Br. cornutus*	1.9	1	Short	Absent	2 intermediate	Naked	Naked	Intermediate			
*C. igae*	2.2	5	Long	Absent	2 intermediate	Spine	Long	Intermediate			
*C. isiae*	2.1	4	Long	Absent	2 intermediate	Spine	Long	Intermediate			
*E. eximia*	3.5	3	Short	Present	1 intermediate	Seta	Short	Intermediate			
*G. gudmunssoni*	2.4	2	Short	Absent	1 intermediate	Spine	Short	Long			
*H. inermis*	n/a	n/a	Long	Absent	1 intermediate	n/a	n/a	Intermediate			
*H. hansjacobi*	2.7	4	Long	Absent	n/a	Spine	Short	Intermediate			
*H. partykae*	2.5	5	Long	Absent	1 intermediate	Spine	Short	Intermediate			
*J. bioice*	1.5	3	Short	Absent	Long and short	Spine	Short	Long			
*Sa. georgi*	2.3	4	Short	Present	Naked	Spine	Long	n/a			
*Sarsotanais* sp. A	n/a	n/a	n/a	n/a	n/a	n/a	n/a	n/a			
*Su. carringtonae*	1.8	3	Short	Present	1 intermediate	Seta	Short	Intermediate			
*Su. frenchae*	2.5	3	Short	Present	2 intermediate	Naked	Naked	Intermediate			

Species	Pereopods 4-6					Uropod					
	Ischium setae	Carpus prickly tubercles	Propodus dorsodisal seta	Unguis	Pleopods	Uropod endopod	Uropod exopod	Uropod exopod/endopod			
*L. amabilis*	2	Present	Short	Simple	Regular	Uniarticulate	Uniarticulate	0.8			
*L. crassus*	2	Present	Short	Simple	Regular	Uniarticulate	Uniarticulate	0.7			
*L. kamchaticus*	2	Present	Short	Simple	Regular	Uniarticulate	Uniarticulate	0.7			
*L. martini*	2	Present	Short	Simple	Regular	Uniarticulate	Uniarticulate	0.9			
*L. siegi*	2	Present	Short	Simple	Regular	Uniarticulate	Uniarticulate	0.8			
*L. tillardi*	2	Present	Short	Simple	Regular	Uniarticulate	Uniarticulate	0.8			
*Br. adipatus*	2	Present	Long	n/a	Regular	Uniarticulated	Uniarticulated	0.8			
*Br. anitae*	2	Present	Long	Bifurcate	Regular	Uniarticulate	Uniarticulate	0.8			
*Br. skolimowskae*	2	Present	Long	Bifurcate	Regular	Uniarticulate	Uniarticulate	0.9			
*Br. kozakowskae*	2	Present	Long	Bifurcate	Regular	Uniarticulate	Biarticulate	0.8			
*Br. nagayae*	1	Present	Long	Bifurcate	Regular	Uniarticulate	Biarticulate	0.9			
*Br. sadleckae*	2	Present	Long	Bifurcate	Regular	Uniarticulate	Biarticulate	0.8			
*Br. andeepae*	2	Present	Long	Bifurcate	Regular	Biarticulate	Biarticulate	0.8			
*Br. cornutus*	2	Present	Long	Bifurcate	Regular	Biarticulate	Biarticulate	0.8			
*C. igae*	2	Present	Long	Bifurcate	Rudimentary	Uniarticulate	Uniarticulate	0.7			
*C. isiae*	2	Cusps	Long	Bifurcate	Regular	Uniarticulate	Uniarticulate	0.7			
*E. eximia*	2	Present	Short	Simple	Regular	Biarticulate	Biarticulate	1			
*G. gudmunssoni*	2	Cusps	Short	Bifurcate	Regular	Biarticulate	Uniarticulate	0.9			
*H. inermis*	n/a	Cusps	n/a	Bifurcate	Regular	n/a	n/a	0.6			
*H. hansjacobi*	2	Cusps	Short	Bifurcate	Regular	Biarticulate	Uniarticulate	0.6			
*H. partykae*	2	Cusps	Short	Bifurcate	Regular	Biarticulate	Uniarticulate	0.7			
*J. bioice*	2	Present	Short	Bifurcate	Regular	Uniarticulate	Uniarticulate	0.8			
*Sa. georgi*	2	Present	Short	Bifurcate	Regular	Biarticulate	Uniarticulate	0.7			
*Sarsotanais* sp. A	n/a	n/a	n/a	n/a	n/a	n/a	n/a	n/a			
*Su. carringtonae*	1	Present	Short	Simple	Regular	Biarticulate	Uniarticulate	0.8			
*Su. frenchae*	2	Present	Short	Bifurcate	Regular	Biarticulate	Uniarticulate	0.8

- ***Larsenotanais amabilis* Błażewicz-Paszkowycz, 2007**
- *Larsenotanais amabilis*—Błażewicz-Paszkowycz (2007)^27^: 6, 26, 41–46.

#### Diagnosis

Body 6.0 L:W. Pereonite-1 as long as pereonite-2. Antenna article-2 with short seta; article-3 without seta. Cheliped carpus 2.7 L:W. Pereopod-1 basis with middorsal seta; pereopod-2 carpus 1.7 L:W. Uropod exopod 0.7× endopod.

#### Distribution

Known only from the type locality: N Weddell Sea, W Antarctic, from 2893 to 3683 m^27^.

#### Remarks

*Larsenotanais amabilis* has pereonite-1 as long as pereonite-2, distinguishing it from *L. kamchatikus* and *L. tillardi*, where it is shorter than pereonite-2.

- ***Larsenotanais crassus*** **(Dojiri & Sieg, 1997) n. comb.**
- *Typhlotanais crassus*—Dojiri & Sieg (1997)^53^: 256–258.

#### Diagnosis

Body 5.0 L:W. Pereonite-1 shortest, half as long as pereonite-2. Cheliped carpus 2.4 L:W. Pereopod-1 basis with four dorsal setae; pereopod-2 carpus 1.7 L:W. Uropod exopod 0.7× endopod.

#### Distribution

Known only from the type locality: E Pacific, Santa Monica Bay, from 77–80 m^53^.

#### Remarks

*Larsenotanais crassus* and *L. kamchatikus* have a uropod exopod 0.7× endopod, distinguishing them from *L. amabilis, L. tillardi* and *L. siegi* that have a slightly longer exopod, 0.8× endopod. Furthermore, the relatively stout cheliped carpus (2.4 L:W) distinguishes *L. crassus* from *L. kamchatikus* whose carpus is 3.4 L:W (Table 2).

- ***Larsenotanais kamchatikus*** **Błażewicz-Paszkowycz, 2007**
- *Larsenotanais kamchatikus*—Błażewicz-Paszkowycz (2007)^47^: 2, 13–17; Larsen & Shimomura (2007)^54^: 11; Stępień et al. (2019)^55^: 3; Gellert et al. (2022)^28^: 3.

#### Diagnosis

Body 6.5 L:W. Pereonite-1 shortest, half as long as pereonite-2. Antenna article-2 with short seta; article-3 with short seta. Cheliped carpus 3.1 L:W. Pereopod-1 basis with two middorsal and one proximoventral setae; pereopod-2 carpus 2.1 L:W. Uropod exopod 0.7× endopod.

#### Distribution

Known only from its type locality: Kuril–Kamchatka Trench, at depths 3145–3265 m^47^*. Larsenotanais kamchatikus* is the only species of the genus recorded from the NW Pacific. The other species in the Pacific is *L*. *crassus*.

#### Remarks

*Larsenotanais kamchatikus* is distinguished from its four congeners by the slender cheliped carpus (3.1 L:W). In the other two species this article is much stouter, e.g. *L. amabilis* and *L. martini* 2.7 L:W, 2.4 L:W in *L. crassus,* and 2.5 L:W in *L. siegi*. The stoutest cheliped carpus is present in *L. tillardi* (3.0 L:W) (Table 2).

**Figure 3:**
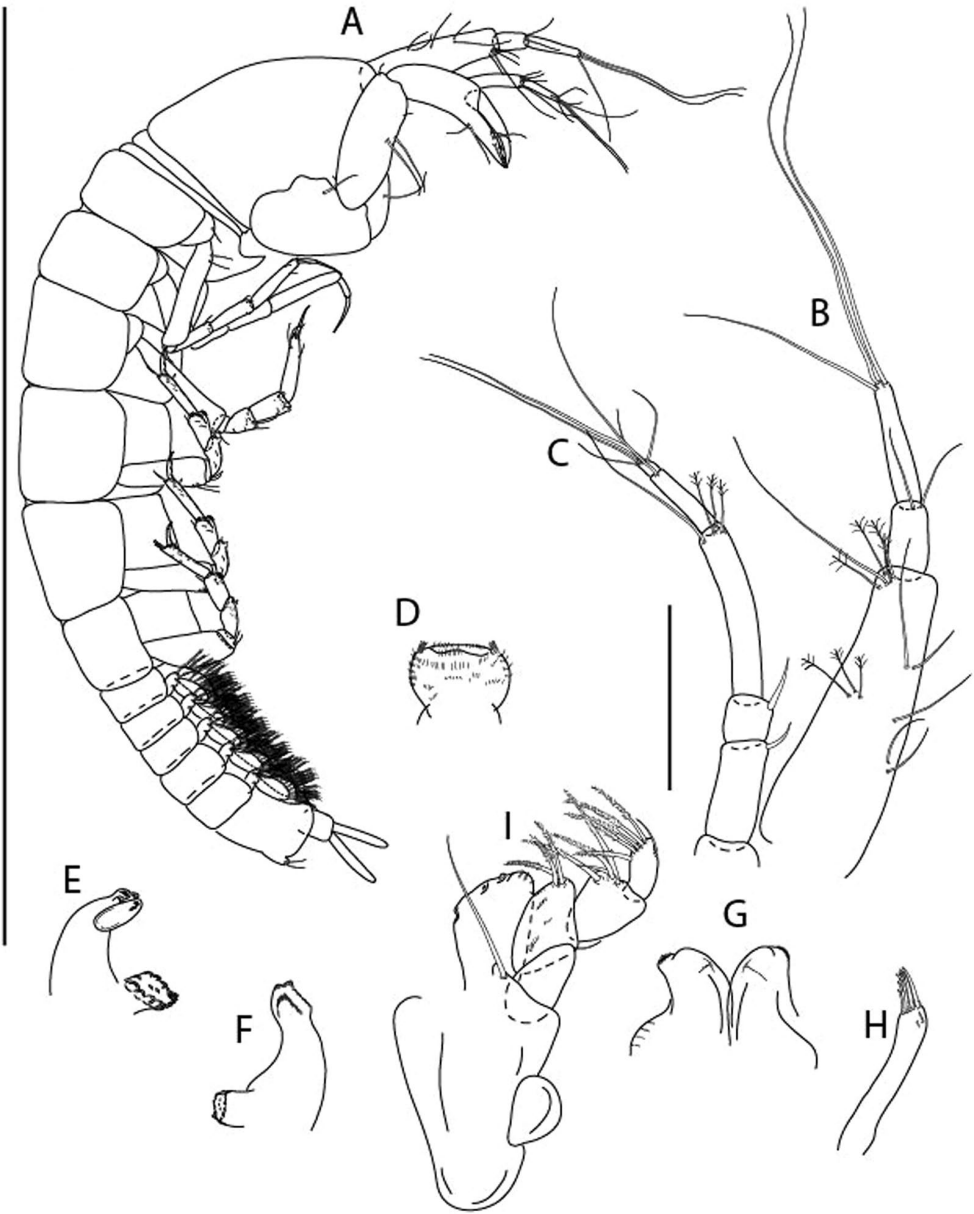
*Larsenotanais martini* n. sp., neuter, (**A**) Body, lateral view; (**B**) Antennule; (**C**) Antenna; (**D**) Labrum; (**E**) Left mandible; (**F**) Right mandible; (**G**) Labium; (**H**) Maxillule; (**I**) Maxilla; (**I**) Maxilliped. Scale: A = 1 mm, B–I = 0.1 mm (A = ICUL10031, ZMHK-64308; B = ICUL3096, ZMHK-64309, ZMHK-64309).

**Figure 4:**
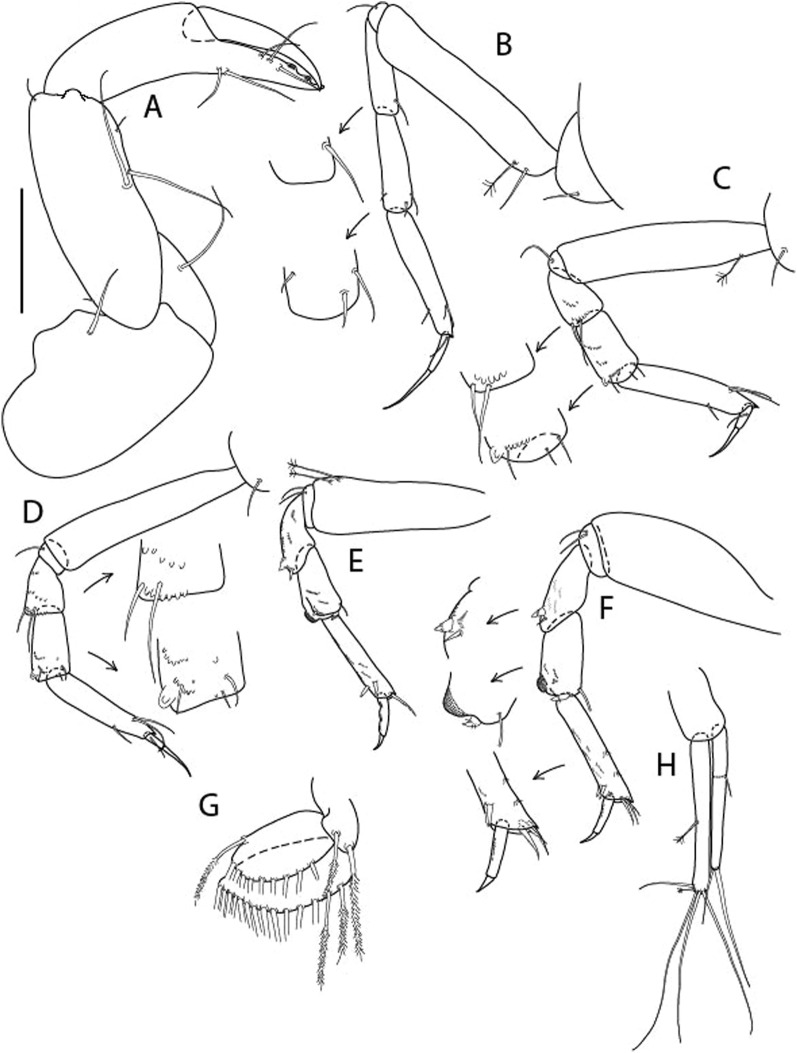
*Larsenotanais martini* n. sp., neuter (ICUL3096, ZMHK-64309), (**A**) Cheliped; (**B**) Pereopod-1; (**C**) Pereopod-2; (**D**) Pereopod-3; (**E**) Pereopod-4; (**F**) Pereopod-6; (**G**) Pleopod; (**H**) Uropod. Scale: A–H = 0.1 mm.

- ***Larsenotanais martini*** **Gellert, Błażewicz & Bird n. sp.**
- LSID urn:lsid:zoobank.org:act:3E1CF5AC-2FD7-41F6-9163-A45E28949022.
- (Figs. 3, 4).

#### Material examined

Holotype, IceAGE, 1072-1, neuter 0.9 mm (ICUL10031, ZMHK-64308). Paratype, IceAGE, 1072-1, neuter 1 mm (ICUL3096, ZMHK-64309; dissected).

#### Other material

BIOICE St. 2839, neuter; BIOICE St. 3278, neuter; BIOICE St. 3280, six neuters 1.4−1.6 mm; BIOICE St. 3515, four neuters; BIOICE St. 3518, seven neuters and two preparatory males 1.0 mm and 1.2 mm; BIOICE 3527, neuter 1.5 mm; BIOICE St. 3533, neuter; BIOICE St. 3539, neuter 1.7 mm.

#### Diagnosis

Body 4.3 L:W. Pereonite-1 shorter than pereonite-2. Antenna article-2 with stiff seta; article-3 with stiff seta. Cheliped carpus 2.7 L:W. Pereopod-1 basis with proximodorsal seta and PSS; pereopod-2 carpus 1.9 L:W. Uropod exopod 0.9× endopod.

#### Etymology

This species is dedicated to the first author’s friends—Karolina Mielcarek-Jeserich, Martin Jeserich and their daughter Helenka Jeserich, for the years of friendship.

#### Description of neuter,

length 1.0 mm. Body (Fig. 3A) stout, compact, 4.3 L:W. Cephalothorax trapezoidal, 1.3 L:W, 3.4× pereonite-1, naked. Pereonites 1−6: 0.3, 0.4, 0.5, 0.5, 0.6 and 0.6 L:W, respectively; all pereonites with rounded lateral margins. Pereonite-1 0.8× pereonite-2; pereonite-2 0.8× pereonite-3; pereonite-3 0.9× pereonite-4; pereonite-4 as long as pereonite-5; pereonite-5, 1.4× pereonite-6. Pleon 0.2× BL; pleonites 1−5: of similar size – 0.3 L:W. Pleotelson 3.4× pereonite-6.

Antennule (Fig. 3B) 1.1× cephalothorax; article-1, 0.6 of antennule length, 3.7 L:W, with five setae and three PSS at midlength, and long seta (longer than article-2) and four PSS distally; article-2, 2.2 L:W, 0.3× article-1, with two distal setae; article-3, 5.3 L:W, 1.5× article-2, with three terminal setae.

Antenna (Fig. 3C) article-2, 2.4 L:W, with distal seta; article-3, 1.2 L:W, 0.5× article-2, with distal seta; article-4 5.8 L:W, 3.4× article-3, with long seta (longer than article-5) and three PSS distally; article-5 3.5 L:W, 0.5× article-4, with long seta; article-6 1.9 L:W, with five distal setae.

Mouthparts. Labrum (Fig. 3D) typical. Mandible (Fig. 3E−F) molar typical. Left mandible (Fig. 3E) incisor with three cusps, *lacinia mobilis* well developed, with single cusp; right mandible (Fig. 3F) incisor with two cusps. Labium (Fig. 3G) typical, outer corner of inner lobe and outer lobe with minute setae. Maxillule (Fig. 3H) typical. Maxilla (Fig. 3I) triangular.

Maxilliped (Fig. 3I) basis 1.6 L:W, endite cusps moderate; palp article-1 naked; article-2 typical, with microtrichia along article; articles 3–4 typical. Epignath lost during dissection.

Cheliped (Fig. 4A) basis slender, with dorsolateral seta; 1.9 L:W; merus seta long; carpus 2.7 L:W, with two long and short setae ventrally, dorsal margin setation typical; chela longer than carpus, 4.0 L:W; palm 1.5× fixed finger; fixed finger cutting edge with two blunt distal cusps; dactylus slightly curved.

Pereopod-1 (Fig. 4B) overall 15.4 L:W; coxa with seta; basis 5.2 L:W, with single dorsoproximal seta and PSS; merus 3.5 L:W with dorsodistal seta; carpus 3.9 L:W, 1.1× merus, with two dorsodistal setae and ventrodistal seta; propodus 5.4 L:W, 1.3× carpus, with two dorsodistal and one dorsoventral setae; dactylus 0.8× unguis, together 0.8× propodus.

Pereopod-2 (Fig. 4C) overall 11.2 L:W; coxa with seta; basis 4.3 L:W, with dorsoproximal PSS; merus 1.8 L:W, with two ventrodistal setae and calcified microtrichia along article; carpus 2.0 L:W, 1.1× merus, with two dorsodistal setae, two ventrodistal setae and short spine, and calcified microtrichia along article; propodus 4.8 L:W, as long as merus and carpus combined, with two dorsodistal and one ventrodistal setae; dactylus 0.8× unguis, together 0.8× propodus.

Pereopod-3 (Fig. 4D) similar to pereopod-2 but more slender, overall, 13.2 L:W; basis 5.5 L:W, naked; merus 1.6 L:W; carpus 2.1 L:W, 1.3× merus, with three setae and spine distally; propodus 4.3 L:W, 1.6× carpus, with two dorsodistal and one ventrodistal setae; dactylus 0.6× unguis, together 0.5× propodus.

Pereopod-4 (Fig. 4E) overall 8.9 L:W; basis robust, 3.5 L:W, with two distal PSS; merus 2.7 L:W, with two, unequal, distal spines; carpus 2.4 L:W, 1.1× merus, with moderate prickly tubercles, dorsodistal chemosensory seta, and distal spine/crotchet; propodus 5.1 L:W, with two ventrodistal spines, dorsodistal serrate seta typical; dactylus 2.4× unguis, together 0.5× propodus; unguis simple.

Pereopod-5 the same as pereopod-4.

Pereopod-6 (Fig. 4F) as pereopod-4 but basis naked; propodus with the three dorsodistal serrate setae more slender.

Pleopod (Fig. 4G) exopod with eleven plumose setae on outer margin; endopod with fifteen.

Uropod (Fig. 4H) endopod slender, 10 L:W, with PSS at midlength, and PSS and four setae distally; exopod with fusion line and associated seta, 0.9× endopod, other setation typical.

#### Distribution

**Figure 5:**
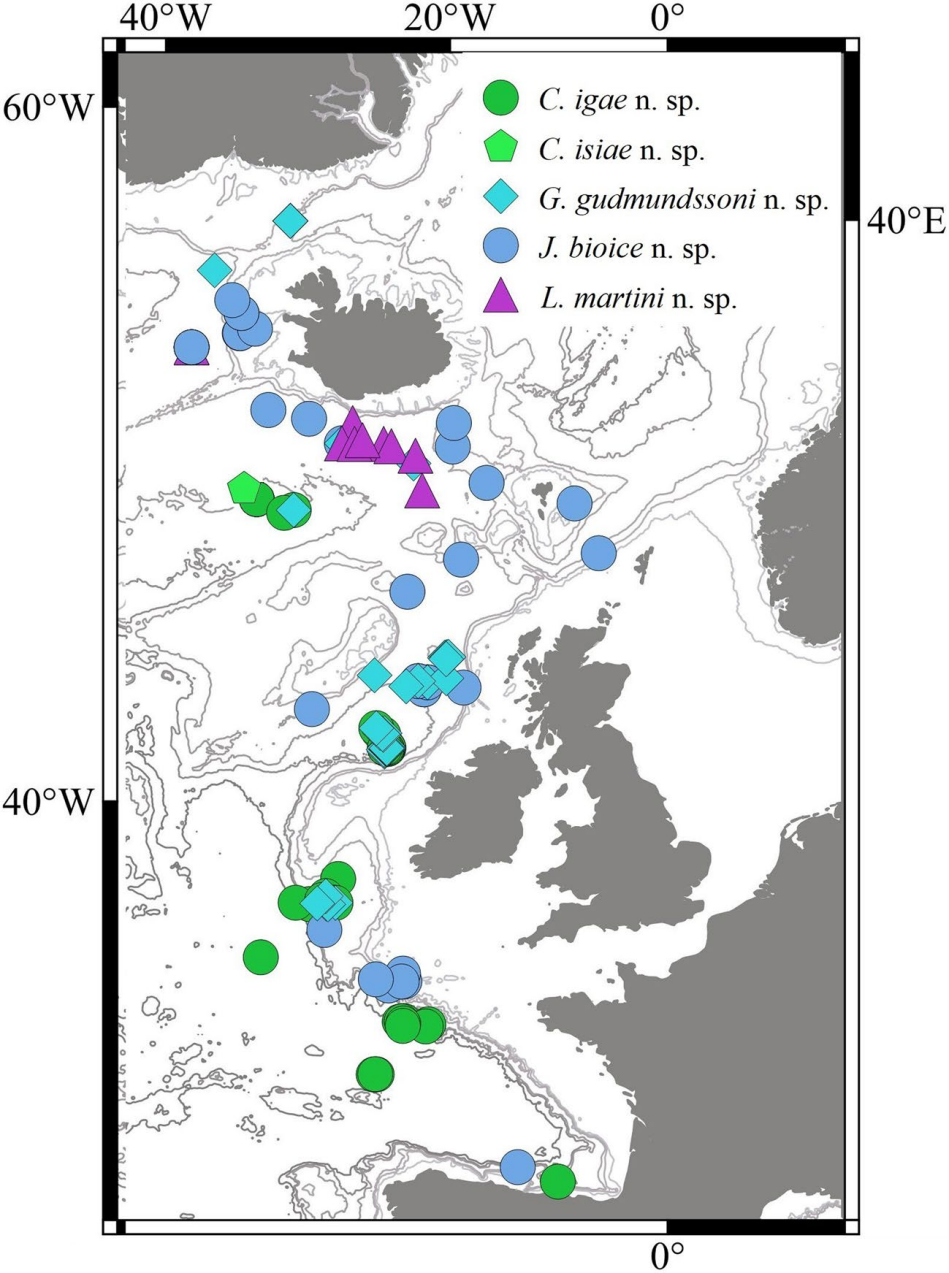
The depth distribution of *C. igae, C. isiae, G. gudmundssoni, J. bioice* and *L. martini* (QGIS 3.28 software https://www.qgis.org/pl/site/).

Known from two locations off Iceland (Iceland and Irminger Basins) (Fig. 5), from a depth of 988–1693 m (this study).

#### Remarks

The antenna article-3 supporting a stiff seta distinguishes *L. martini* n. sp. from *L. amabilis*, which lacks a seta, but also from *L. kamchatikus* and *L. tillardi*, which have a short and slender seta. Additionally, in *L. martini* the uropod exopod is 0.9× endopod, proportionately longer than in all other known species of *Larsenotanais* (Table 2).

- ***Larsenotanais siegi*** **Gellert & Błażewicz n. comb.**
- LSID urn:lsid:zoobank.org:act:52C0E8CB-274A-4894-B2DA-08B9E9A97D15.

#### Synonyms

*Peraeospinosus adipatus*—Sieg (1986)^56^: xi, 5, 9–10, 79, 85–94, 96.

*Typhlotanais greenwichensis*—Błażewicz-Paszkowycz (2007)^27^: 98.

#### Diagnosis

Body 6.0 L:W. Pereonite-1 shortest, half as long as pereonite-2. Antenna article-2 with two setae; article-3 naked. Cheliped carpus 2.5 L:W. Pereopod-1 basis two dorsal and one ventral setae; pereopod-2 carpus 2.4 L:W. Uropod exopod 0.8× endopod.

#### Distribution

Known from one location south of the Antarctic Peninsula, from the depth range 51–285 m^56^.

#### Remarks

Sieg (1986) illustrated and described a species that is considered to be conspecific with *Typhlotanais adipatus* described by Tzareva (1982)^57^ from the shelf of the Cosmonaut Sea in E Antarctica based on collections made on the shelf of the western Antarctic Peninsula. Also, he considered his species to be a representative of the genus *Peraeospinosus*. As a consequence, the species described by Tzareva (*Ty. adipatus*) has become member of *Peraeospinosus*. In light of current knowledge and with a better understanding of the morphology of typhlotanaids, it is clear that Sieg (1986)^56^ and Tzareva (1982)^57^ studied two distinct species belonging to two different genera. This difference was perceived by Błażewicz-Paszkowycz (2007)^27^ who classified Tzareva’s *Ty. adipatus* to the ‘cornutus’ group, but considered Sieg’s *Ty. adipatus* a synonym of *Typhlotanais greenwichensis* Shiino, 1970. Unfortunately, Tzareva incompletely illustrated her specimen, but in her figure of the pereopod-4 (Tzareva 1982: Fig. 9^57^) has a propodus distodorsal seta clearly longer than in the same appendage illustrated by Sieg (Sieg 1986: Fig. 55^56^). Besides, the two taxa have a quite different body habitus, where short pereonites and a short cephalothorax are clear in Tzareva’s species and more elongated pereonites and a slender cephalothorax are present in Sieg’s species. The set of these features is sufficient to assess that the species described by Tzareva is closely related to *Typhlotanais cornutus* G.O. Sars, 1879, in contrast to the species described by Sieg. Since it has all the characteristics that define genus *Larsenotanais*, we have made the decision to transfer it to this genus, and name it as *Larsenotanais siegi* n. sp.

*Larsenotanais amabilis* and *L. siegi* are two Antarctic *Larsenotanais* congeners which could be distinguished by the length of pereonite-1 (clearly shorter than pereonite-2 in *L. siegi*, and almost as long as pereonite-2 in *L. amabilis*), and the setation of antenna article-2 (one seta in *L. amabilis*, and two in *L. siegi*). Finally, the distodorsal seta on the propodus of pereopods 4–5 is long in *L. siegi* (reaching the end of dactylus) and short in *L. amabilis* (0.5× dactylus).

- ***Larsenotanais tillardi*** **Bamber, 2014**
- *Larsenotanais tillardi*—Bamber (2014)^58^: 18–23.

#### Diagnosis

Body 5.0 L:W. Pereonite-1 shortest, half as long as pereonite-2. Antenna article-2 with short seta; article-3 with short seta. Cheliped carpus 3.0 L:W. Pereopod-1 basis naked. Uropod exopod 0.8× endopod.

#### Distribution

Known only from the type locality: Azores, Sabrina Bank, from 140 to 200 m^58^.

#### Remarks

As *L. tillardi*, three other *Larsenotanais* species have an uropod exopod reaching 0.8× of endopod length. Nevertheless *L. tillardi* differs from *L. amabilis* and *L. siegi* by the lack of mesial setation on the uropod endopod, where *L. amabilis* has a PSS and *L. siegi* has four simple and two PSS. Also, *L. tillardi* has a naked pereopod-1 basis, where *L. amabilis* has a middorsal seta and *L. siegi* has two middorsal setae and proximoventral seta.

### Key for identification of *Larsenotanais* neuters


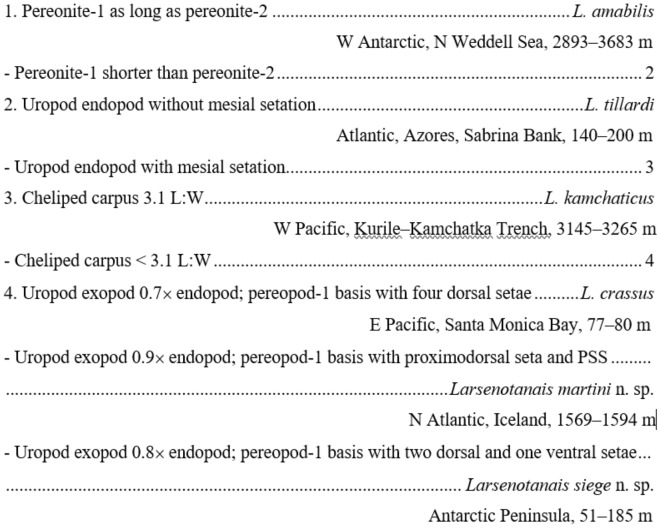


#### Diagnosis

Body stout, pereonite margins convex (rounded). Antennule article-1 short (< 4.0 L:W), mesial margin with fewer than four setae. Maxilliped basis seta longer than endites, endite cusps moderate. Cheliped carpus short (< 2.5 L:W), carpus with short ventral seta. Pereopod-1 merus L:W short (< 3.0 L:W), carpus without long seta; pereopods 2–3 carpus ventrodistal seta short, microtrichia regular (if present); pereopods 4–6 carpus with prickly tubercles, propodus distodorsal seta long, unguis bifurcate. Uropod endopod and exopod uniarticulate or biarticulate.

#### Type genus

*Brevitanais andeepae* (Błażewicz-Paszkowycz, 2007).

#### Species included

*Brevitanais adipatus* (Tzareva, 1982) n. comb; *Br. andeepae* (Błażewicz-Paszkowycz, 2007) n. comb.; *Br. cornutus* (G. O. Sars, 1879) n. comb; *Br. anitae* n. sp.; *Br. kozakowskae* n. sp.; *Br. nagayae* n. sp.; *Br. sadleckae* n. sp.; *Br. skolimowskae* n. sp.

#### Etymology

Brevis [Gr.] means short, reflecting the stout habitus of species classified in this genus.

#### Remarks

Błażewicz-Paszkowycz (2007)^27^ proposed a morpho-group ‘cornutus’ for typhlotanaids with a ‘stout-bodied’ habitus as: *Ty. cornutus*; *Ty. adipatus*; *Ty. crassus*, and *Ty. andeepae* Błażewicz-Paszkowycz, 2007, and noted that these species have biarticulate uropod rami. A further detailed analysis of nine species (four listed species and five new species studied during the current study), which morphologically fit well into the group, support establishing a new genus—*Brevitanais*.

The new genus is defined by several morphological characters that allows separation from the other ‘stout-bodied’ typhlotanaids (Table 2). A long dorsodistal seta on the pereopods 4–6 propodus is characteristic of *Brevitanais* and *Caesatanais*, although the former as genus has a short antennule article-1 (long in *Caesatanais*) and rounded pereonite margins (straight in *Caesatanais*). Because of the variety in uropod articulation, the genus is provisionally divided into three groups based on the number of articles in both uropod rami: group-1 with uniarticulate uropodal rami; group-2 with uniarticulate uropodal exopod and biarticulate uropodal endopod, and group-3 with biarticulate uropodal rami.

- ***Brevitanais* group-1**

#### Diagnosis

Uropod rami uniarticulate; exopod about 0.8–0.9× endopod.

#### Species included

*Brevitanais adipatus* (Tzareva, 1982); *Brevitanais skolimowskae* n. sp. (see below); *Brevitanais anitae* n. sp. (see below).

#### Remarks

The species classified to *Brevitanais* n. gen. group-1 are most similar to *Larsenotanais* species by having uniarticulate uropod rami and short antennule article-1, although they also have a bifurcate unguis in pereopods 4–6 (simple in *Larsenotanais*) and a shorter cheliped carpus, which is < 2.4 L:W (> 2.4 L:W in *Larsenotanais*).

*Typhlotanais bolarticulus* Segadilha & Serejo, 2022 has been provisionally classified in the ‘cornutus’ group^59^. Because of its unarticulate exopod rami it could be specifically classified within the *Brevitanais* group-1, although several morphological characters highlight its morphological distinctiveness, e.g.: large distoventral spine on the pereopods 2–3 carpus (relatively small in *Brevitanais*), simple pereopods 4–6 unguis (bifurcate in *Brevitanais*) and large prickly tubercles on the pereopods 4–6 carpus, which cover most of the article’s ventral side (small in *Brevitanais*). Besides, *Ty. bolarticulus* has parallel pereonite margins in dorsal view, which are clearly rounded in all *Brevitanais* species*.* For this reason, we conclude that *Ty. bolarticulus* probably is not closely related to any of the taxa related to *Brevitanais* (‘earlier’ as ‘cornutus’ group), and leave its in original generic classification (*Typhlotanais*) until its taxonomic position is better understood. Nevertheless, it is worth emphasizing that by having certain features, i.e. pereopods 2–3 carpus and propodus with spines and pereopods 4–6 carpus with large prickly tubercles *Ty. bolarticulus* may resemble *Starkus* Gellert, Palero & Błażewicz, 2022, although having uniarticulate uropods and a ‘stout-bodied’ form, exclude the species from that genus.

- ***Brevitanais adipatus*** **(Tzareva, 1982) n. comb.**

#### Synonyms

*Typhlotanais adipatus*—Tzareva (1982)^57^: 53–54; Larsen (2005)^60^: 216; Błażewicz-Paszkowycz (2007)^27^: 6, 25, 33–34, 46, 98, 126, 131; Błażewicz-Paszkowycz (2014)^61^: 484; Błażewicz-Paszkowycz et al. (2014)^33^: 417–418, 427, 449; Segadilha & Serejo (2022)^59^: 21, 27.

*Peraeospinosus adipatus*—Błażewicz-Paszkowycz & Jażdżewski (2000)^62^: 176; Błażewicz-Paszkowycz & Sekulska-Nalewajko (2004)^63^: 226–227; Błażewicz-Paszkowycz (2005)^46^: 3847–3849; Larsen & Shimomura (2007)^54^: 27, 28.

non *Peraeospinosus adipatus* Sieg (1986)^56^: xi, 5, 9–10, 79, 85–94, 96 (see *Larsenotanais siegi* n. sp.).

#### Diagnosis

Antennule article-1 3.2 L:W. Antenna article-2 1.8 L:W; antenna articles 2–3 naked. Cheliped carpus 2.3 L:W. Pereopod-1 merus 2.3 L:W; carpus with seta, propodus with short ventrodistal seta. Uropod exopod 0.8× endopod.

#### Distribution

Known only from the type locality: E Antarctica, Cosmonauts Sea, from a depth of 45–58 m^57^.

#### Remarks

*Brevitanais adipatus* can be immediately recognized by the presence of a short seta on the propodus of pereopod-1, where the other members of the group, i.e. *Brevitanais skolimowskae* (see below) and *Brevitanais anitae* (see below), have a long seta (Table 2). Besides, *Br. adipatus* has a relatively long antennule article-2 relatively long (> 3.0 L:W) and antenna article-3 without a seta, whereas the other species from *Brevitanais* group-1 have a much shorter antennule article-2, and one seta on antenna article-3.

**Figure 6:**
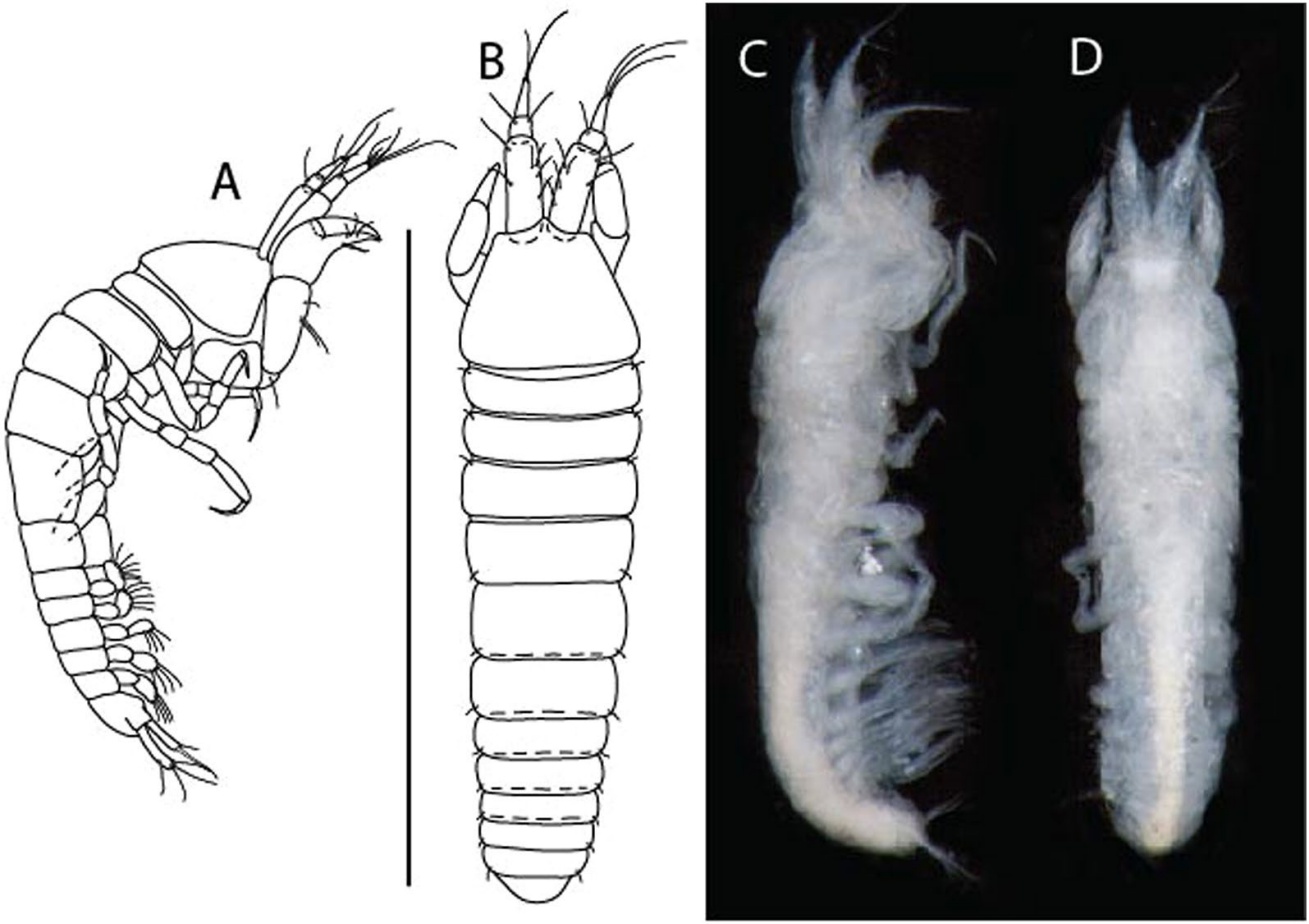
*Brevitanais anitae* n. sp., neuter (ICUL3382, ZMHK-64312), (**A**, **C**) Body, lateral view; (**B**, **D**) Body, dorsal view. Scale = 1 mm.

**Figure 7:**
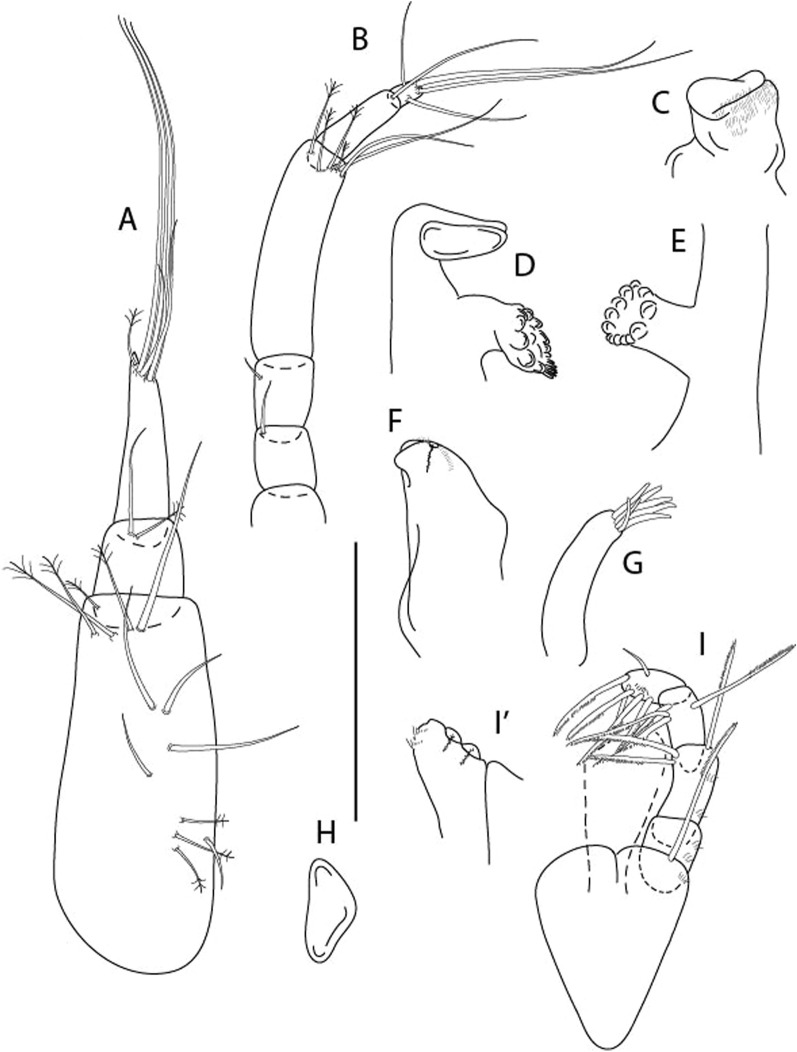
*Brevitanais anitae* n. sp., neuter (ICUL3381, ZMHK-64313), (**A**) Antennule; (**B**) Antenna; (**C**) Labrum; (**D**) Left mandible; (**E**) Right mandible; (**F**) Labium; (**G**) Maxillule; (**H**) Maxilla; (**I**, **I’**) Maxilliped. Scale: A–I’ = 0.1 mm.

**Figure 8:**
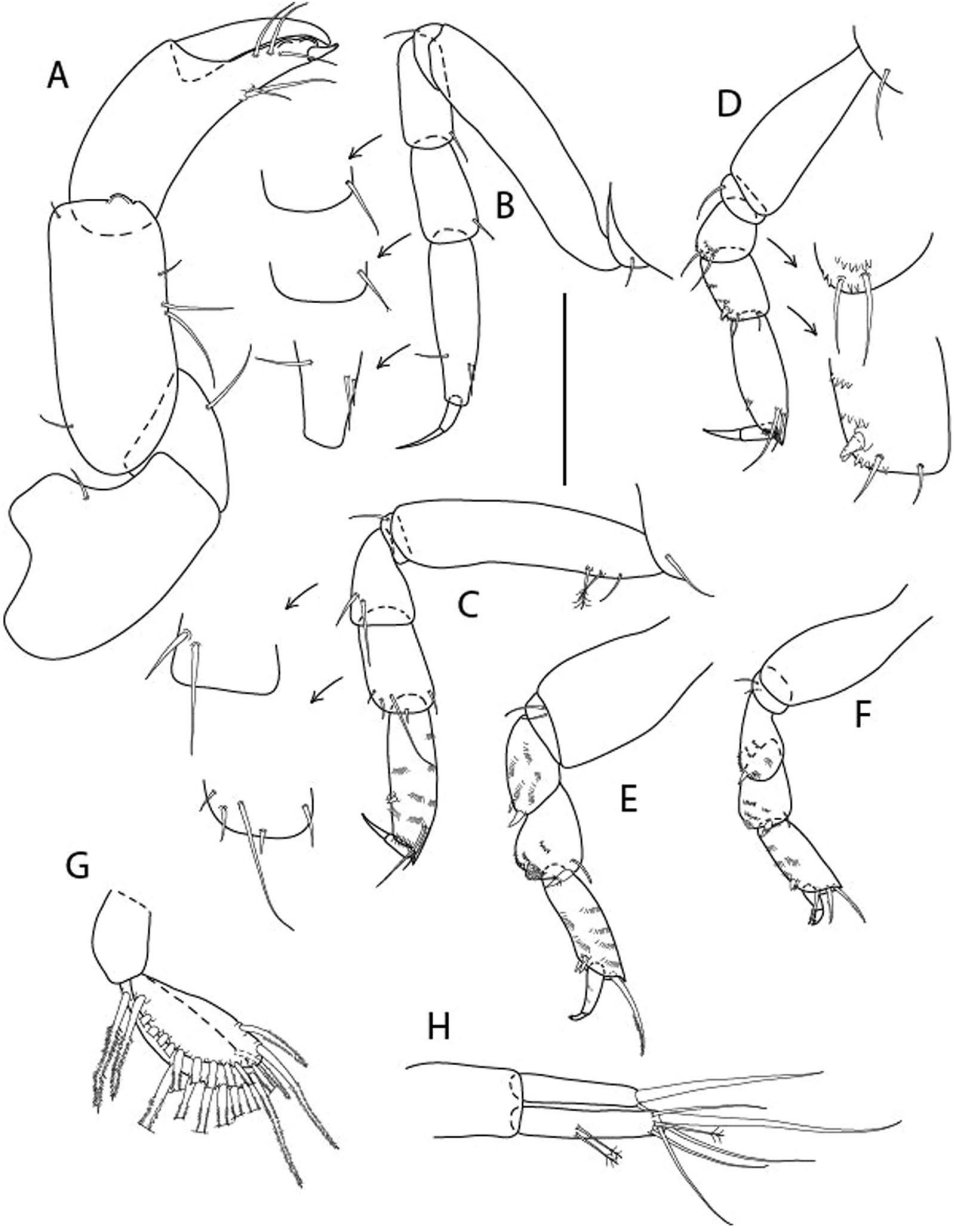
*Brevitanais anitae* n. sp., neuter, (**A**) Cheliped; (**B**) Pereopod-1; (**C**) Pereopod-2; (**D**) Pereopod-3; (**E**) Pereopod-5; (**F**) Pereopod-6; (**G**) Pleopod; (**H**) Uropod. Scale: A–H = 0.1 mm. (A–H = ICUL3381, ZMHK-64313).

- ***Brevitanais anitae*** **Gellert & Błażewicz n. sp.**
- LSID urn:lsid:zoobank.org:act:91A0F4B8-4434-4027-BA2B-AA984B1666D8.
  (Figs. 6, 7 and 8).

#### Material examined

Holotype, neuter 1 mm IceAGE, St. 1043-1, (ICUL3383, ZMHK-64311). 

Paratypes IceAGE, St. 1043-1, neuter 1 mm (ICUL3382, ZMHK-64310); IceAGE, St. 1043-1, neuter 1 mm (ICUL3382, ZMHK-64312); IceAGE, St. 1043-1, neuter 0.8 mm (ICUL3381, ZMHK-64313; dissected); IceAGE, St. 1043–1, neuter 1.1 mm (ICUL3376, ZMHK-64314).

#### Diagnosis

Antennule article-1 2.4 L:W. Antenna article-2 1.0 L:W, antenna articles 2–3 with seta. Cheliped carpus 2.4 L:W. Pereopod-1 merus 2.1 L:W, carpus with seta, propodus with long ventrodistal seta; pereopod-2 merus 1.7 L:W, with two short inferodistal setae, carpus 1.8 L:W; pereopod-3 carpus 1.7 L:W. Uropod exopod 0.8× endopod.

#### Etymology

This species is dedicated to athlete Anita Włodarczyk, the Polish Olympic champion and European and national record-holder in the hammer throw.

#### Description of neuter,

Length 1.0 mm. Body (Fig. 6A−D) very stout, 3.9 L:W. Cephalothorax trapezoidal, 0.9 L:W, 3.6× pereonite-1, naked. Pereonites 1−6: 0.2, 0.2, 0.3, 0.4, 0.5 and 0.4 L:W, respectively; all pereonites rounded, with short lateral seta. Pereonite-1 0.8× pereonite-2; pereonite-2 0.8× pereonite-3; pereonite-3 0.8× pereonite-4; pereonite-4 0.8× pereonite-5; pereonite-5 1.2× pereonite-6. Pleon 0.2× BL; pleonites 1−5: of similar size—0.3 L:W, with short lateral seta. Pleotelson 3.2× pereonite-6.

Antennule (Fig. 7A) 1.1× cephalothorax; article-1, 0.6 of antennule length, 2.5 L:W, with five setae and three PSS at midlength and long seta (longer than article-2) and four PSS distally; article-2, 1.3 L:W, 0.3× article-1, with a PSS and seta distally; article-3, 4.8 L:W, 1.8× article-2, with distal spur, PSS and five terminal setae.

Antenna (Fig. 7B) article-2, 1.2 L:W, with distal seta; article-3, 1.5 L:W, 1.2× article-2, with distal seta; article-4, 3.8 L:W, 2.6× article-3, with two long setae (longer than article-5) and four PSS distally; article-5, 3.5 L:W, 0.5× article-4, with long seta; article-6 2.0 L:W, with four distal setae.

Mouthparts. Labrum (Fig. 7C) typical. Mandible (Fig. 7D−E) molar typical. Left mandible (Fig. 7D) incisor with single cusp, *lacinia mobilis* well developed, with single cusp; right mandible (Fig. 7E) incisor broken. Labium (Fig. 7F) typical, with cusps. Maxillule (Fig. 7G) endite with seven terminal spines. Maxilla (Fig. 7H) triangular.

Maxilliped (Fig. 7I, I’) basis 1.7 L:W, seta finely serrate; endite cusps moderate; palp article-1 with numerous microtrichia; article-2 long, setation typical, with numerous microtrichia; articles 3–4 typical. Epignath lost during the dissection.

Cheliped (Fig. 8A) basis slender, with short dorsolateral seta; 2.0 L:W; merus seta fairly short; carpus 2.4 L:W, with two long setae (shorter than carpus width) and short seta ventrally, dorsal margin setation typical; chela longer than carpus, 3.2 L:W; palm 1.7× fixed finger; fixed finger cutting edge with two blunt distal cusps; dactylus slightly curved.

Pereopod-1 (Fig. 8B) overall 12.2 L:W; coxa with seta, basis 4.6 L:W, naked; merus 2.1 L:W with dorsodistal seta; carpus 2.1 L:W, 0.9× merus, with distal bifurcate seta; propodus 3.9 L:W, 1.6× carpus, with two dorsodistal setae and one ventrodistal setae; dactylus 0.7× unguis, together 0.5× propodus.

Pereopod-2 (Fig. 8C) overall 7.2 L:W; coxa with seta; basis 2.7 L:W, with dorsoproximal seta and two PSS; merus 1.7 L:W, with two ventrodistal setae; carpus 1.7 L:W, 1.1× merus, with dorsodistal seta and four ventrodistal setae (one long, others short); propodus 3.5 L:W, 0.8× merus and carpus combined, with two dorsodistal setae and small ventrodistal spine, and microtrichia along article; dactylus 0.9× unguis, together 0.4× propodus.

Pereopod-3 (Fig. 8D) similar to pereopod-2 but more slender, overall, 10.2 L:W; basis 2.9 L:W, naked; merus 1.4 L:W, with two ventrodistal setae, and calcified microtrichia; carpus 1.4 L:W, 1.1× merus, with numerous calcified microtrichia, two distal setae, and ventrodistal spine; propodus 2.7 L:W, 1.8× carpus; dactylus 1.1× unguis, together 0.6× propodus.

Pereopod-4 the same as pereopod-5.

Pereopod-5 (Fig. 8E) overall 5.0 L:W; basis robust, 2.7 L:W, naked; merus 1.7 L:W, with two distal spines; carpus 1.4 L:W, as long as merus, with moderate prickly tubercles, dorsodistal chemosensory seta, and distal spine/crotchet; propodus 2.1 L:W, with two ventrodistal spines, dorsodistal long serrate seta longer than dactylus and unguis; dactylus 1.3× unguis, together 0.6× propodus.

Pereopod-6 (Fig. 8F) as pereopod-5 but propodus three dorsodistal setae shorter.

Pleopod (Fig. 8G) exopod with eight plumose setae on outer margin; endopod with thirteen.

Uropod (Fig. 8H) endopod 5.0 L:W, with two PSS at midlength, and PSS and five setae distally; exopod 0.8× endopod, with two setae (one thin and one thick).

#### Distribution

**Figure 9:**
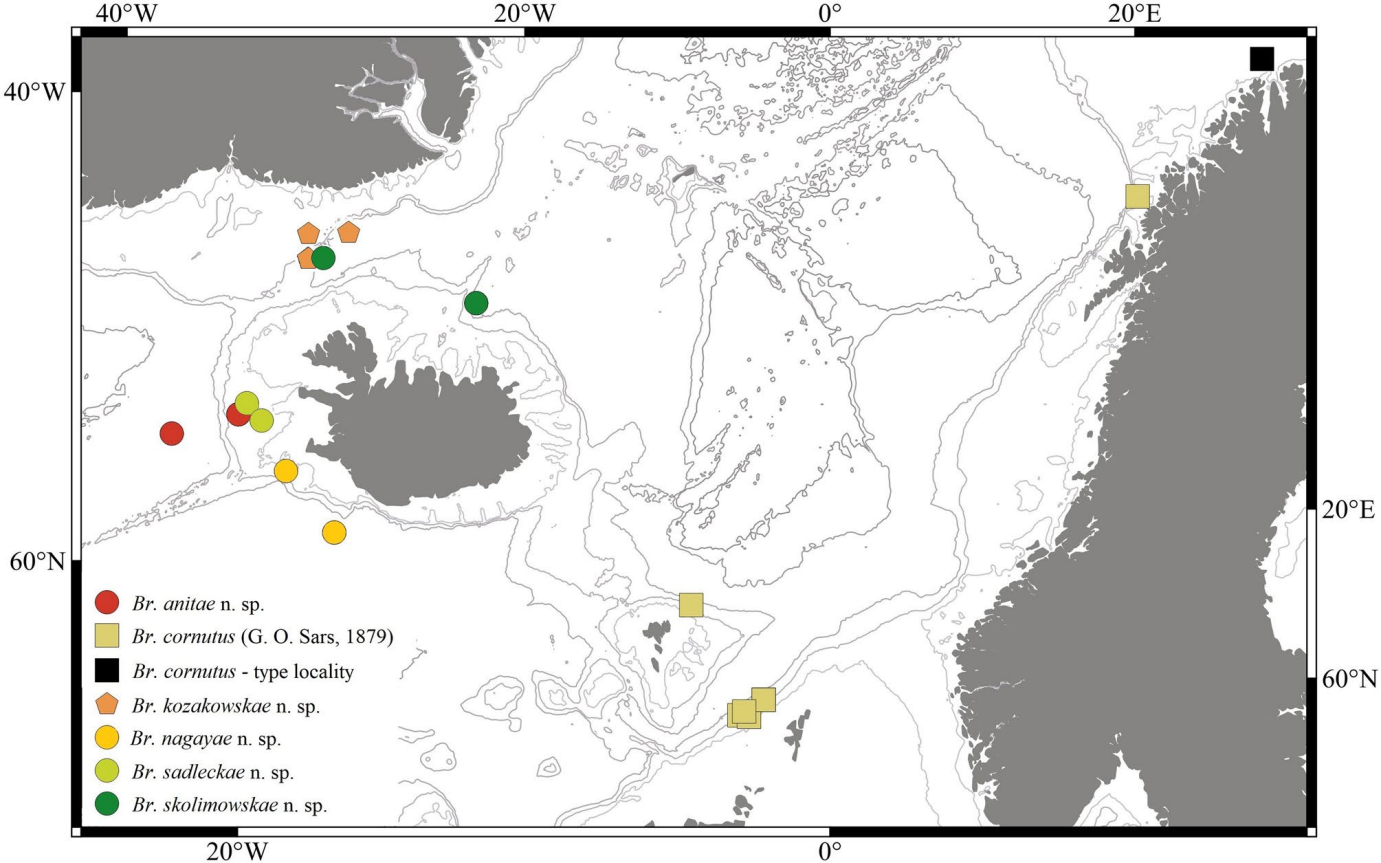
The depth distribution of *Br. anitae, Br. cornutus, Br. kozakowskae, Br. nagayae, Br. sadleckae,* and *Br. skolimowskae* (QGIS 3.28 software https://www.qgis.org/pl/site/).

Known from one location off Iceland (Reykjanes Ridge) (Fig. 9) from a depth of 213.9−224.9 m (this study).

#### Remarks

*Brevitanais anitae* n. sp. can be distinguished from the Antarctic species *Br. adipatus* by the short ventrodistal seta on the pereopod-1 propodus. Furthermore, it has a stout merus (1.7 L:W) and carpus (1.8 L:W) on pereopods 2–3, respectively, which are only 1.4 L:W and 1.1 L:W, respectively in *Br. skolimowskae* n. sp. (see below) (Table 2).

**Figure 10:**
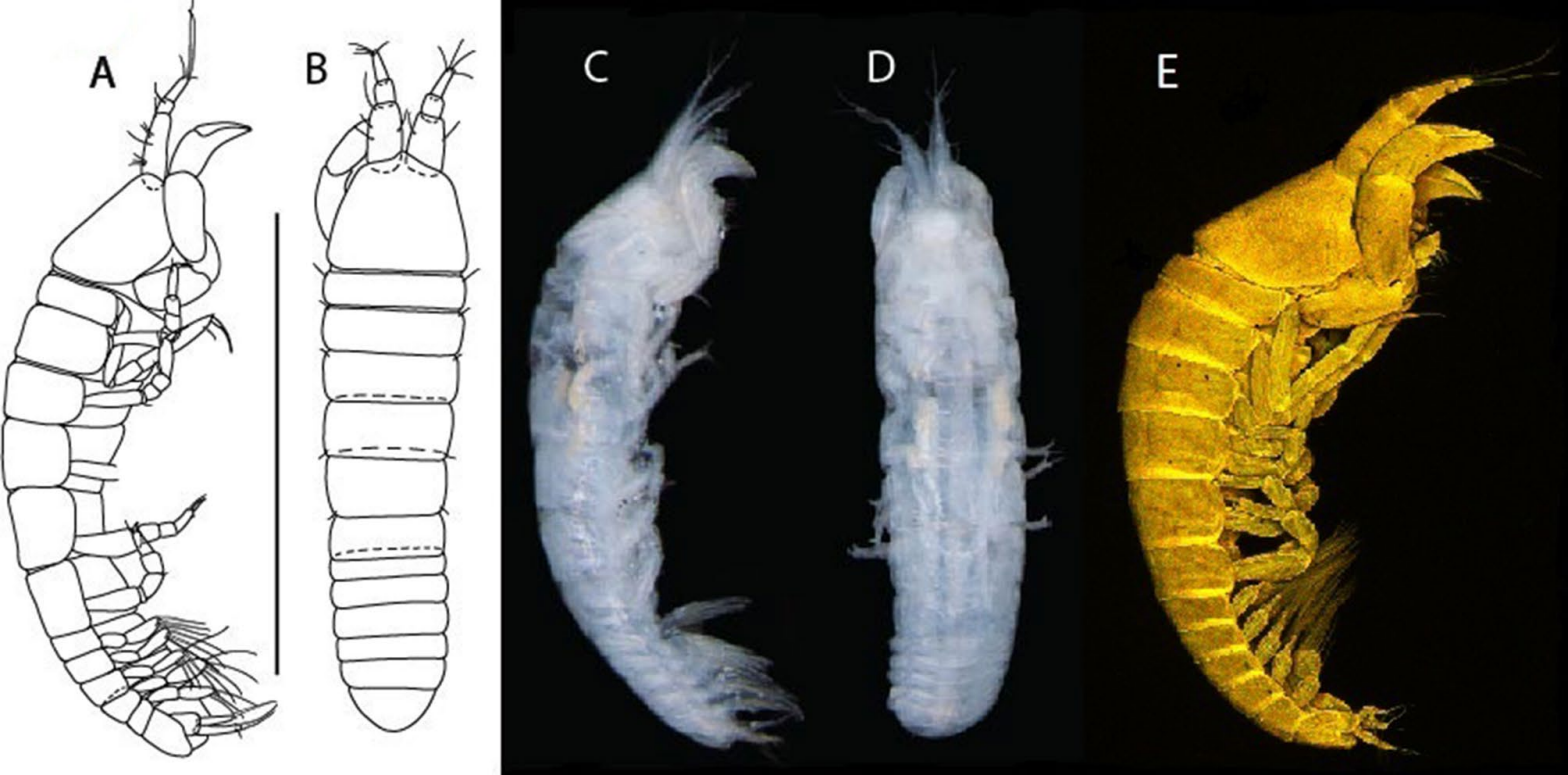
*Brevitanais skolimowskae* n. sp., neuter (ICUL3378, ZMHK-64325), (**A**, **C**) Body, lateral view; (**B**, **D**) Body, dorsal view; (**E**) CLSM images: Body, lateral view. Scale = 1 mm.

**Figure 11:**
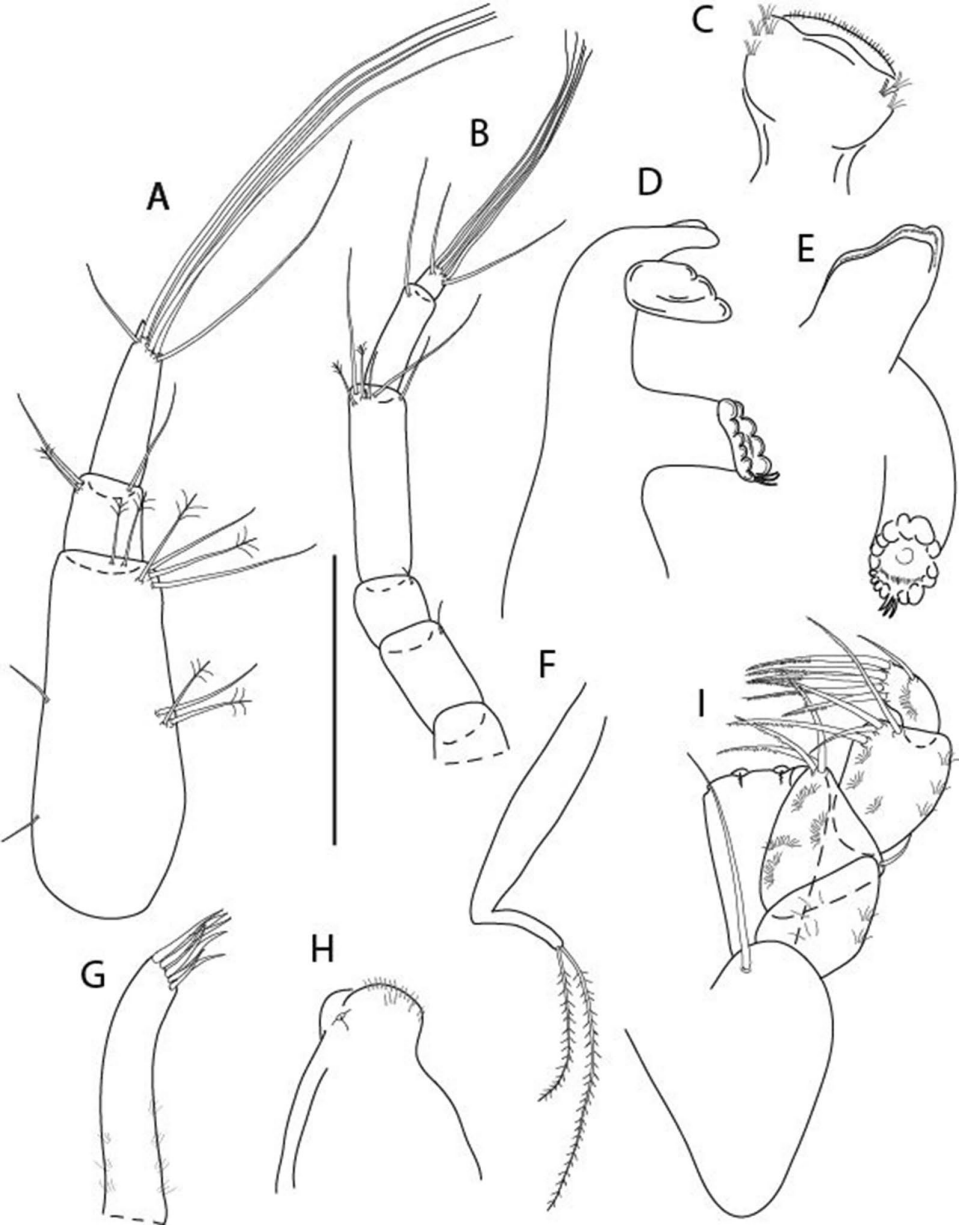
*Brevitanais skolimowskae* n. sp., neuter (ICUL3372, ZMHK-64321), (**A**) Antennule; (**B**) Antenna; (**C**) Labrum; (**D**) Left mandible; (**E**) Right mandible; (**F**) Palp; (**G**) Maxillule; (**H**) Labium; (**I**) Maxilliped. Scale: A–I = 0.1 mm.

**Figure 12:**
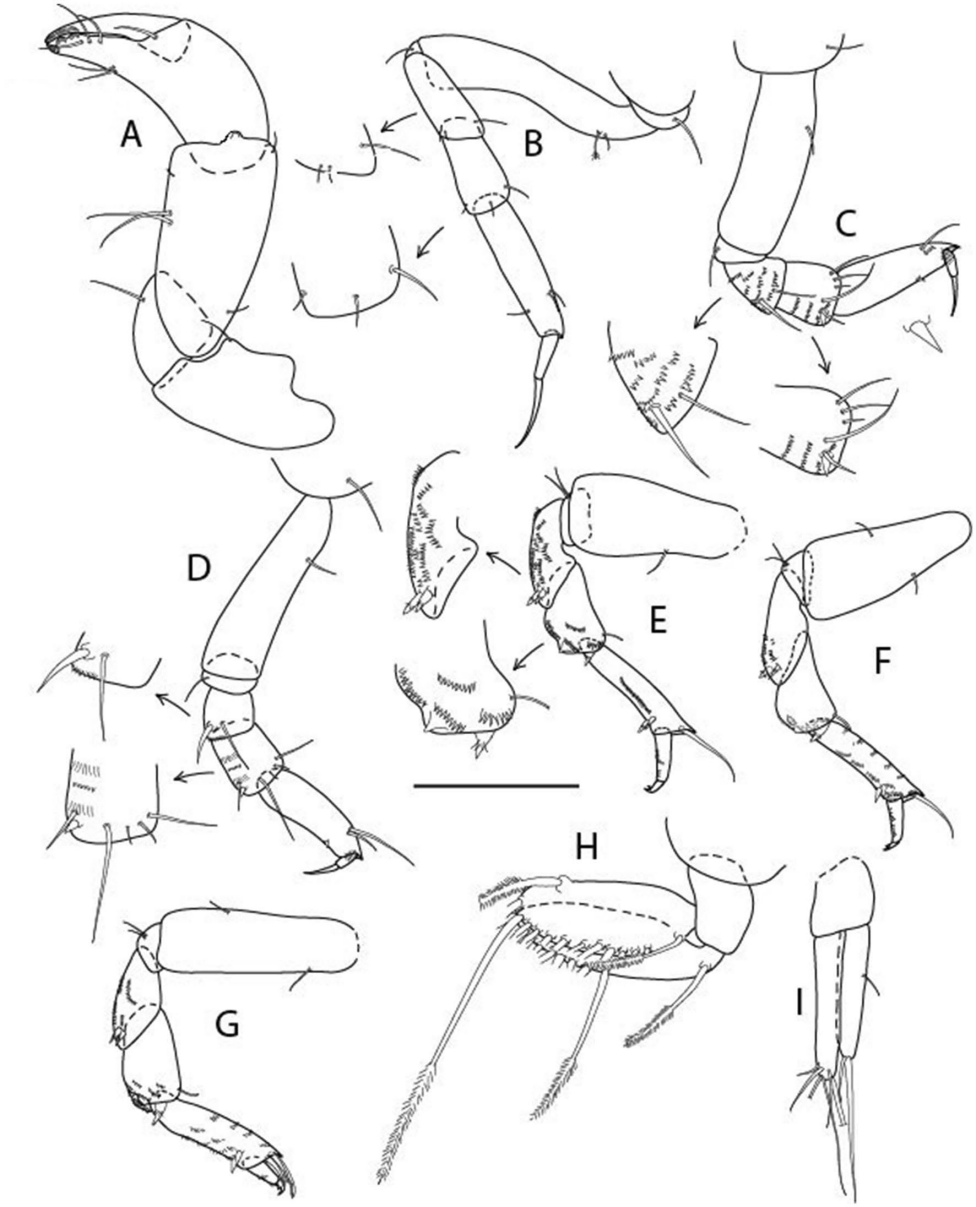
*Brevitanais skolimowskae* n. sp., neuter, (**A**) Cheliped; (**B**) Pereopod-1; (**C**) Pereopod-2; (**D**) Pereopod-3; (**E**) Pereopod-4; (**F**) Pereopod-5; (**G**) Pereopod-6; (**H**) Pleopod; (**I**) Uropod. Scale: A–I = 0.1 mm (A–D = ICUL3364, ZMHK-64323; E–I = ICUL3372, ZMHK-64321).

- ***Brevitanais skolimowskae*** **Gellert & Błażewicz n. sp.**
- LSID urn:lsid:zoobank.org:act:6E18A965-0572-418C-BF99-0DBD8DC55DF0.
- (Figs. 10, 11 and 12).

#### Material examined

Holotype, neuter 1.7 mm, IceAGE, St. 1119-1 (ICUL3357, ZMHK-64315).

Paratypes IceAGE, St. 1119-1, neuter 1.4 mm (ICUL3380, ZMHK-64316); IceAGE, St. 1119-1, manca-3 0.9 mm (ICUL3375, ZMHK-64317); IceAGE, St. 1119-1, neuter 1.3 mm (ICUL3370, ZMHK-64318; dissected); IceAGE, St. 1129-1, neuter 1.1 mm (ICUL3377, ZMHK-64319); IceAGE, St. 1129-1, neuter 1.2 mm (ICUL3374, ZMHK-64320); IceAGE, St. 1129-1, neuter 1.3 mm (ICUL3372, ZMHK-64321; dissected); IceAGE, St. 1129-1, neuter 1 mm (ICUL3371, ZMHK-64322); IceAGE, St. 1129-1, neuter 1.3 mm (ICUL3364, ZMHK-64323; dissected); IceAGE, St. 1136-1, neuter 1.2 mm (ICUL3379, ZMHK-64324); IceAGE, St. 1136-1, neuter 1.2 mm (ICUL3378, ZMHK-64325).

#### Diagnosis

Antennule article-1 2.7 L:W. Antenna article-2 1.8 L:W, antenna articles 2–3 with setae. Cheliped carpus 2.3 L:W. Pereopod-1 merus 2.4 L:W, carpus with three setae, propodus with long ventrodistal seta; pereopod-2 merus 1.4 L:W with two long inferodistal setae, carpus 1.1 L:W; pereopod-3 carpus 1.3 L:W. Uropod exopod 0.9× endopod.

#### Etymology

This species is dedicated to Kamila Skolimowska, Polish Olympic Games gold medallist in the hammer throw.

#### Description of neuter,

Length 1.2 mm. Body (Fig. 10A−E) stout, compact, 4.1 L:W. Cephalothorax square-shaped, 0.9 L:W, 3.6× pereonite-1. Pereonites 1−6: 0.2, 0.3, 0.4, 0.5, 0.6 and 0.4 L:W, respectively; all pereonites rounded. Pereonites 1−5 with short lateral seta, pereonite-6 naked. Pereonite-1 0.9× pereonite-2; pereonite-2 0.8× pereonite-3; pereonite-3 0.8× pereonite-4; pereonite-4 0.9× pereonite-5; pereonite-5 1.7× pereonite-6. Pleon 0.1× BL; pleonites 1−5: of similar size – 0.2 L:W. Pleotelson 4.2× pereonite-6.

Antennule (Fig. 11A) 1.0× cephalothorax; article-1 0.6 of antennule length, 2.7 L:W, with two setae at midlength on inner margin, seta and two PSS at midlength on outer margin, and two setae and four PSS distally; article-2 1.3 L:W, 0.3× article-1, with PSS and two setae distally (one seta on inner and seta and a PSS on outer margin); article-3 3.8 L:W, 1.9× article-2, with distal spur and six terminal setae.

Antenna (Fig. 11B) article-2 2.0 L:W, with distal seta; article-3 1.1 L:W, 0.6× article-2, with distal seta; article-4 3.9 L:W, 2.7× article-3, with two PSS and four setae distally; article-5 3.5 L:W, 0.6× article-4, with long seta; article-6 1.0 L:W, with seven distal setae.

Mouthparts. Labrum (Fig. 11C) typical. Mandible (Fig. 11D−E) molar typical. Left mandible (Fig. 11D) incisor with two cusps, *lacinia mobilis* well developed, with single cusp; right mandible (Fig. 11E) incisor with two cusps. Labium (Fig. 11H) typical, outer corner of inner lobe and outer lobe with minute setae. Maxillule (Fig. 11G) endite typical; palp (Fig. 11F) with two terminal serrate setae. Maxilla lost during the dissection.

Maxilliped (Fig. 11I) basis 1.5 L:W; endite cusps moderate; palp article-1 with numerous microtrichia; articles 2–4 typical, article-4 with numerous microtrichia. Epignath lost during the dissection.

Cheliped (Fig. 12A) basis slender, with short dorsolateral seta; 2.5 L:W; merus ventral seta fairly short; carpus 2.2 L:W, with two long setae (shorter than carpus W) and short seta ventrally, dorsal margin setation typical; chela longer than carpus, 2.6 L:W, with seta; palm 2.0× fixed finger; fixed finger cutting edge with three blunt distal cusps; dactylus slightly curved.

Pereopod-1 (Fig. 12B) overall 15 L:W; coxa with seta, basis 5.3 L:W, with single dorsoproximal seta and PSS; merus 2.4 L:W, with dorsodistal seta and two ventrodistal short setae; carpus 2.5 L:W, 0.9× merus, with three distal setae; propodus 3.2 L:W, 1.6× carpus, with two dorsodistal setae and ventrodistal seta; dactylus 0.7× unguis, together 0.7× propodus.

Pereopod-2 (Fig. 12C) overall 7.4 L:W; coxa with seta; basis 2.9 L:W, with dorsoproximal seta; merus 1.4 L:W, with two ventrodistal setae and numerous calcified microtrichia along article; carpus 1.3 L:W, as long as merus, with numerous calcified microtrichia, three dorsodistal setae (one shorter than the others), two ventrodistal setae (one longer than the other) and spine; propodus 3.2 L:W, 1.1× merus and carpus combined, with two dorsodistal setae (one broken) and small ventrodistal spine; dactylus 0.8× unguis, together 0.5× propodus.

Pereopod-3 (Fig. 12D) similar to pereopod-2 but more slender, overall, 9.7 L:W; basis 4.4 L:W, with dorsoproximal seta; merus 1.2 L:W; carpus 1.3 L:W, 1.3× merus, with numerous microtrichia, three dorsodistal setae (two short), and two setae (one long) and spine ventrodistally; propodus 3.4 L:W, 1.8× carpus; dactylus 0.7× unguis, together 0.4× propodus.

Pereopod-4 (Fig. 12E) overall 6.7 L:W; basis robust, 2.5 L:W, with dorsoproximal seta; merus 2.4 L:W, with two serrate distal spines, and numerous calcified microtrichia along article; carpus 1.9 L:W, 1.1× merus, with moderate prickly tubercles, dorsodistal chemosensory seta, and distal spine/crotchet; propodus 4.9 L:W, with two ventrodistal serrate spines, and dorsodistal seta longer than dactylus and unguis; dactylus 2.7× unguis, together 0.6× propodus.

Pereopod-5 (Fig. 12F) similar to pereopod-4 but overall 6.3 L:W; basis robust, 2.4 L:W, with middorsal and midventral setae; merus 2.2 L:W; carpus 1.9 L:W, 0.9× merus; propodus 4.6 L:W; dactylus 2.9× unguis, together 0.6× propodus.

Pereopod-6 (Fig. 12G) as pereopod-5, but propodus three dorsodistal setae shorter.

Pleopod (Fig. 12H) exopod with ten plumose setae on outer margin; endopod with fourteen.

Uropod (Fig. 12I) endopod 6.0 L:W, with six distal setae; exopod 0.9× endopod, with midlength seta, other setation typical.

#### Distribution

Known from one location off Iceland (Denmark Strait) (Fig. 9), at depths of 315.1−706.4 m (this study).

#### Remarks

*Brevitanais skolimowskae* n. sp. is the second species of the *Brevitanais* group-1 in the N Atlantic. The antennule article-1 (2.7 L:W) and three serrate setae on the pereopod-1 carpus separates *Br. skolimowskae* from *Br. anitae* (2.4 L:W, and one carpal seta). Additionally, *Br. anitae* has a relatively long merus and carpus on pereopods 2–3 (1.7–1.8 L:W), while they are only 1.1–1.4 L:W in *Br. skolimowskae. Br. skolimowskae* is distinguished from *Br. adipatus*, by the short ventrodistal seta on the pereopod-1 propodus (long in *Br. adipatus*) (Table 2).

- ***Brevitanais*** **group-2**

#### Diagnosis

Uropod endopod biarticulate and exopod unarticulate; exopod about 0.8–0.9× endopod.

#### Species included

*Brevitanais kozakowskae* n. sp.; *Brevitanais nagaye* n. sp.; *Brevitanais sadleckae* n. sp.

**Figure 13:**
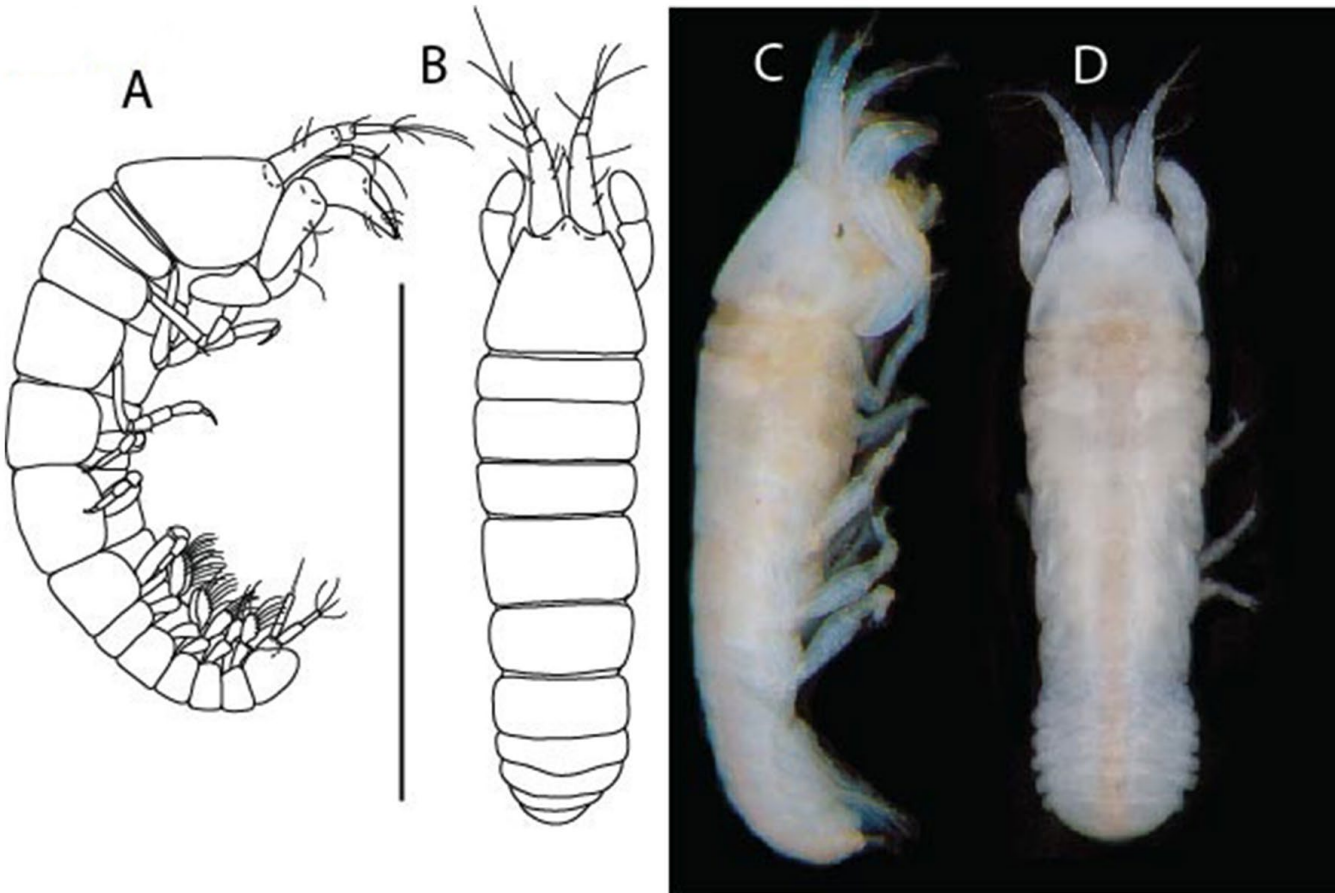
*Brevitanais kozakowskae* n. sp., neuter (ICUL9993, ZMHK-64327), (**A**, **C**) Body, lateral view; (**B**, **D**) Body, dorsal view. Scale = 1 mm.

**Figure 14:**
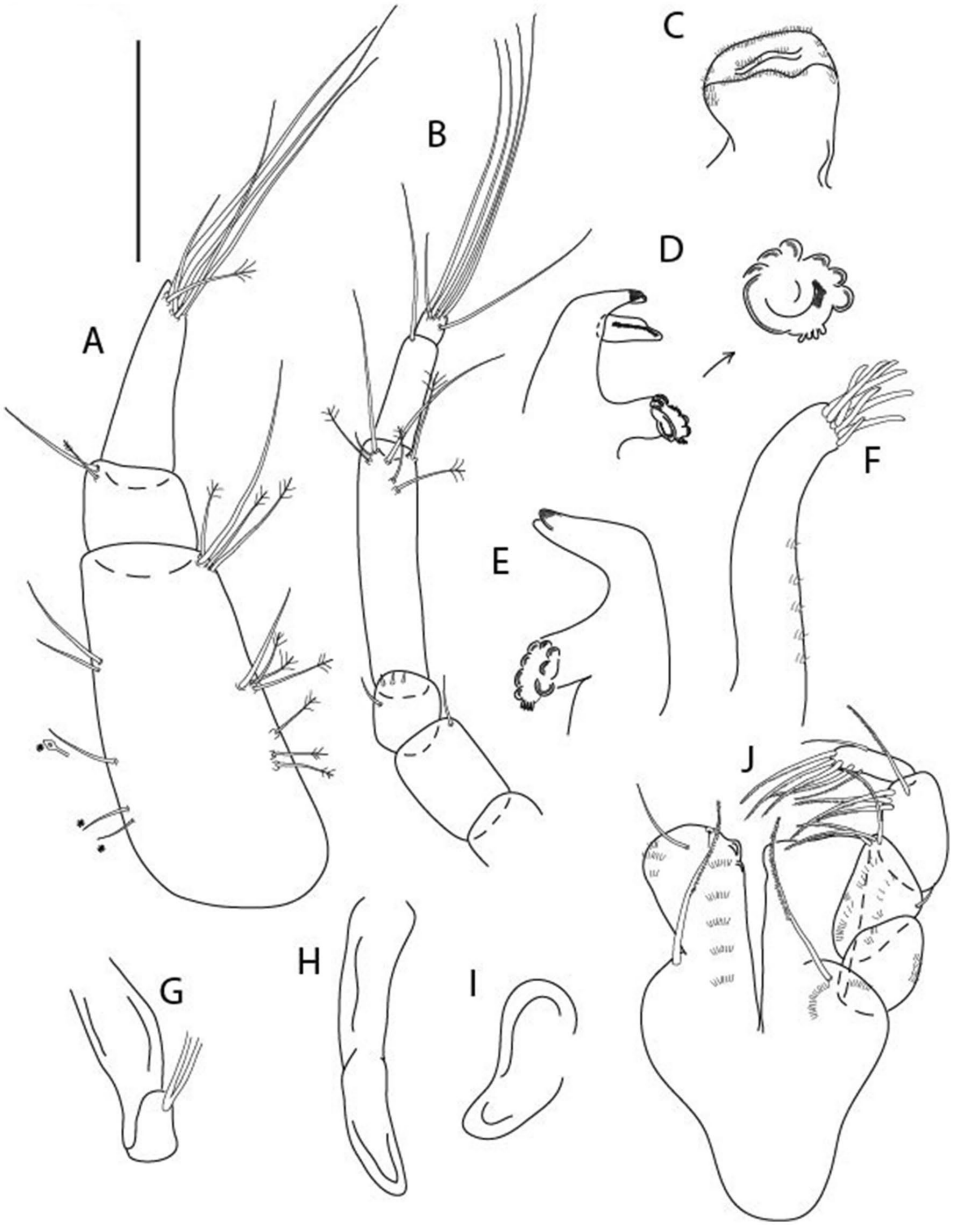
*Brevitanais kozakowskae* n. sp., neuter (ICUL3093, ZMHK-64328), (**A**) Antennule; (**B**) Antenna; (**C**) Labrum; (**D**) Left mandible; (**E**) Right mandible; (**F**) Maxillule; (**G**) Maxilla; (**H**) Epignath; (**I**) Maxilla; (**J**) Maxilliped. Scale: A–J = 0.1 mm.

**Figure 15:**
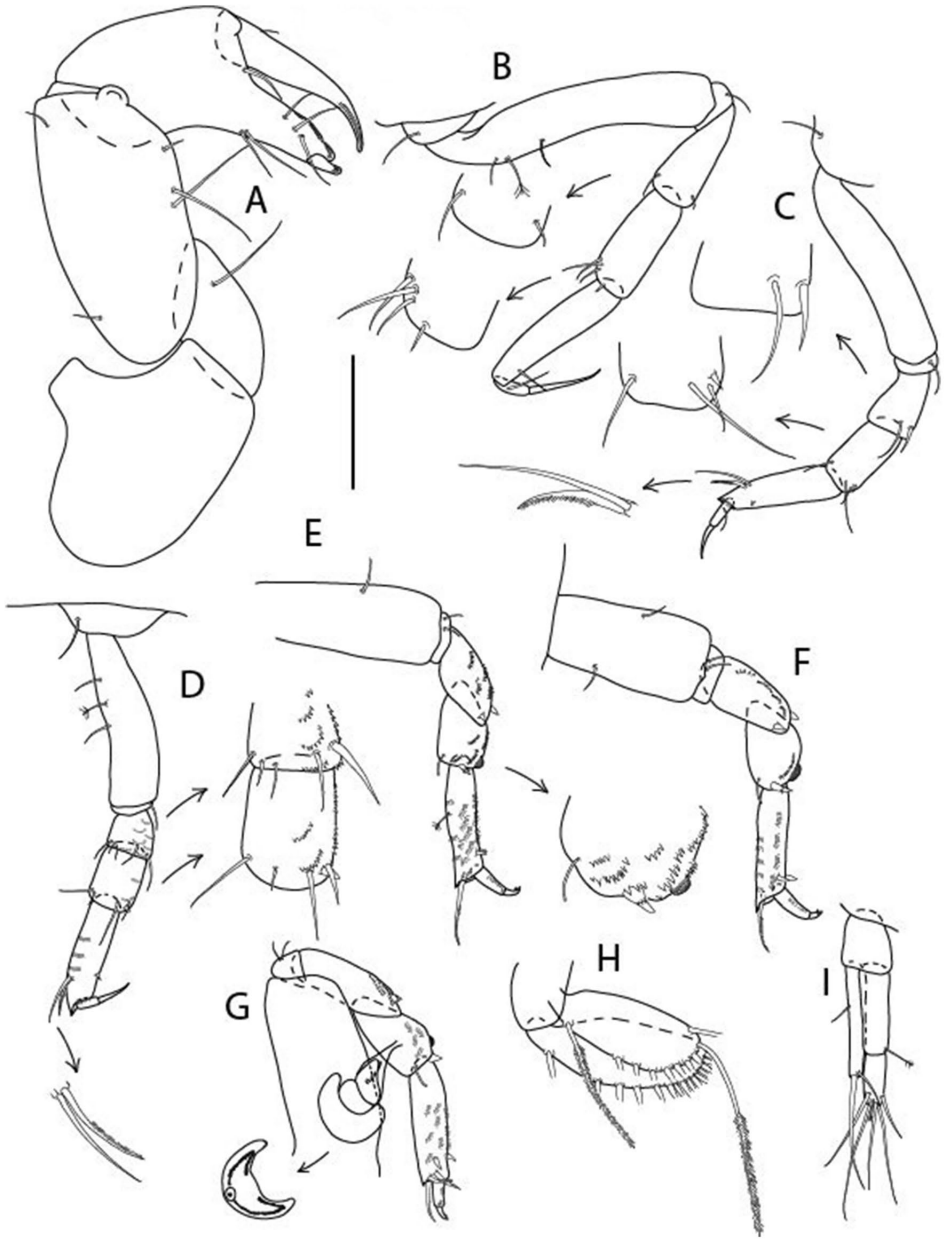
*Brevitanais kozakowskae* n. sp., neuter (ICUL3093, ZMHK-64328), (**A**) Cheliped; (**B**) Pereopod-1; (**C**) Pereopod-2; (**D**) Pereopod-3; (**E**) Pereopod-4; (**F**) Pereopod-5; (**G**) Pereopod-6; (**H**) Pleopod; (**I**) Uropod. Scale: A–I = 0.1 mm.

- ***Brevitanais kozakowskae*** **Gellert & Błażewicz n. sp.**
- LSID urn:lsid:zoobank.org:act:605F4E81-7A8E-4ED3-B7B9-A60AED2A9C62.
  (Figs. 13, 14 and 15).

#### Material examined

Holotype, neuter 1.3 mm, IceAGE, St. 1119-1 (ICUL3082, ZMHK-64326).

Paratypes IceAGE, St. 1123-1, manca-2 0.7 mm, neuter 1.3 mm 0.7 mm, 1.3 mm (ICUL9993, ZMHK-64327); IceAGE, St. 1123-1, neuter 1.4 mm (ICUL3093, , ZMHK-64327; dissected); IceAGE, St. 1123-1, neuter 1.3 mm (ICUL10117; extraction*); IceAGE, St. 1123-1, neuter (ICUL10118, ZMHK-64329; extraction, broken); IceAGE, St. 1123-1, neuter 1.2 mm (ICUL13958, ZMHK-64330; extraction); IceAGE, St. 1123-1, neuter 1.3 mm (ICUL9894, ZMHK-64331; dissected); IceAGE, St. 1123-1, two manca-3 1.1 mm (ICUL8997, ZMHK-64332); IceAGE, St. 1130-1, neuter 1.4 mm (ICUL285, ZMHK-64333).*Individual not recovered after DNA extraction.

#### Diagnosis

Antennule article-1 2.3 L:W. Antenna article-2 1.6 L:W; articles 2–3 with short seta. Cheliped carpus 2.0 L:W. Pereopod-1 merus 2.9 L:W, carpus with four setae, propodus with ventrodistal seta; pereopod-2 merus 1.4 L:W, with two short inferodistal setae; carpus 1.6 L:W; pereopods 2–3 propodus with ventrodistal spine; pereopod-3 carpus 1.5 L:W. Uropod exopod 0.8× endopod.

#### Etymology

This species is dedicated to Róża Kozakowska, Polish paralympic champion and world record holder in the club throw.

#### Description of neuter,

Length 1.5 mm. Body (Fig. 13A−D) stout, 4.3 L:W. Cephalothorax trapezoidal, 0.5 L:W, 4.0× pereonite-1, naked. Pereonites 1−6: 0.2, 0.3, 0.3, 0.5, 0.5 and 0.4 L:W, respectively; all pereonites rounded. Pereonite-1 0.7× pereonite-2; pereonite-2 1.1× pereonite-3; pereonite-3 0.6× pereonite-4; pereonite-4 1.2× pereonite-5; pereonite-5 1.3× pereonite-6. Pleon 0.3× BL; pleonites 1−5: of similar size – 0.2 L: W. Pleotelson 2.7× pereonite-6.

Antennule (Fig. 14A) 1.0× cephalothorax; article-1, 2:2 L:W, with three chemosensory and two setae on inner margin, and seta and six PSS on outer margin and long (longer than article-2) and three PSS; article-2 1.0 L:W, 0.3× article-1, with distal seta and PSS on inner margin; article-3, 4.1 L:W, 0.5× article-2, with PSS, five setae, and terminal spur.

Antenna (Fig. 14B) article-2 1.4 L:W, with distal seta; article-3 1.2 L:W, 0.7× article-2, with seta and spinules; article-4 4.3 L:W, 3.1× article-3, with three setae (two longer than article-5) and four PSS distally; article-5 3.1 L:W, 0.5× article-4, with long seta; article-6 1.3 L:W, with six distal setae.

Mouthparts. Labrum (Fig. 14C) typical. Mandible (Fig. 14D−E) molar typical. Left mandible (Fig. 14D) incisor with two cusps, *lacinia mobilis* well developed, with single cusp; right mandible (Fig. 14E) incisor with two cusps. Maxillule (Fig. 14F) endite typical, three innermost terminal spines shorter than others, microtrichia along endite; palp (Fig. 14G) shorter than endite. Maxilla (Fig. 14J) rounded. Maxilla lost during dissection.

Maxilliped (Fig. 14J) basis seta finely serrate; endite cusps small, with seta in the middle, and microtrichia on outer margin; palp article-1 with numerous microtrichia; articles 2–4 typical. Epignath (Fig. 14H) as long as maxillule endite, rounded distally.

Cheliped (Fig. 15A) basis slender 1.7 L:W; merus ventral seta long; carpus 2.0 L:W, with two long setae (shorter than carpus W) and short seta ventrally, dorsal margin setation typical; chela longer than carpus, 1.5 L:W, with seta on outer margin; palm 1.3× fixed finger; fixed finger cutting edge with three weak, blunt distal cusps; dactylus slightly curved, with seta.

Pereopod-1 (Fig. 15B) overall 16.0 L:W; coxa with seta, basis 5.9 L:W, with dorsoproximal PSS and two setae; merus 2.9 L:W, with single dorsodistal and ventrodistal setae; carpus 2.5 L:W, as long as merus, with four dorsodistal setae; propodus 4.7 L:W, 1.4× carpus, with ventrodistal seta; dactylus 0.6× unguis, with seta, together 1.1× propodus.

Pereopod-2 (Fig. 15C) overall 12.2 L:W; coxa with seta; basis 4.8 L:W, naked; merus 2.0 L:W, with two ventrodistal setae; carpus 2.3 L:W, 1.1× merus, with dorsodistal seta, two ventrodistal setae, and small ventrodistal spine; propodus 3.4 L:W, 0.7× merus and carpus combined, with simple and serrate dorsodistal setae, and ventrodistal spine; dactylus 0.6× unguis, with seta, together 0.6× propodus.

Pereopod-3 (Fig. 15D) similar to pereopod-2 but stouter, overall, 9.0 L:W; basis 3.6 L:W, with middorsal PSS and two setae; merus 1.1 L:W, with two ventrodistal setae and three dorsodistal setae, and numerous calcified microtrichia along article; carpus 1.5 L:W, 1.6× merus, with two dorsodistal setae, two ventrodistal setae, small ventrodistal spine, and numerous calcified microtrichia along article; propodus 3.8 L:W, 1.8× carpus, numerous calcified microtrichia along article; dactylus 0.6× unguis, with seta, together 0.6× propodus.

Pereopod-4 (Fig. 15E) overall 7.4 L:W; basis robust, 2.9 L:W, with midventral seta; merus 2.2 L:W, with two distal spines, and numerous calcified microtrichia along article; carpus 1.7 L:W, 0.8× merus, with moderate prickly tubercles, dorsal chemosensory seta, and distal spine/crotchet; propodus 4.5 L:W, 1.6× carpus, with middorsal PSS, two ventrodistal spines, dorsodistal serrate seta longer than claw, and numerous calcified along article; dactylus 2.0× unguis, together 0.4× propodus.

Pereopod-5 (Fig. 15F) similar to pereopod-4 but overall 5.6 L:W; basis robust, 2.0 L:W, with middorsal and midventral setae; merus 2.2 L:W; carpus 1.7 L:W, 0.9× merus; propodus 4.6 L:W, 1.5× carpus; dactylus 3.5× unguis, together 0.5× propodus.

Pereopod-6 (Fig. 15G) as pereopod-5 but basis naked; propodus three dorsodistal setae as long as claw.

Pleopod (Fig. 15H) exopod with ten plumose setae on outer margin; endopod with fifteen.

Uropod (Fig. 15I) endopod 5.0 L:W, proximal article 1.7× distal article, with a distal PSS; distal article with PSS and five long terminal setae; exopod 1.3× endopod proximal article, with seta at midlength, other setation typical.

#### Distribution

Known from one location off Iceland (Denmark Strait) (Fig. 9) at 318.9–726 m depth (this study).

#### Remarks

*Brevitanais kozakowskae* n. sp. has a ventrodistal spine on the propodus of pereopods 2–3, which distinguishes it from *Br. nagayae* (see below) with a ventrodistal seta (Table 2). From *Brevitanais sadleckae* n. sp. (see below) and *Br. nagayae, Br. kozakowskae* can be distinguished by the four setae on the pereopod-1 carpus (three in *Br. nagayae* and *Br. sadleckae*).

**Figure 16:**
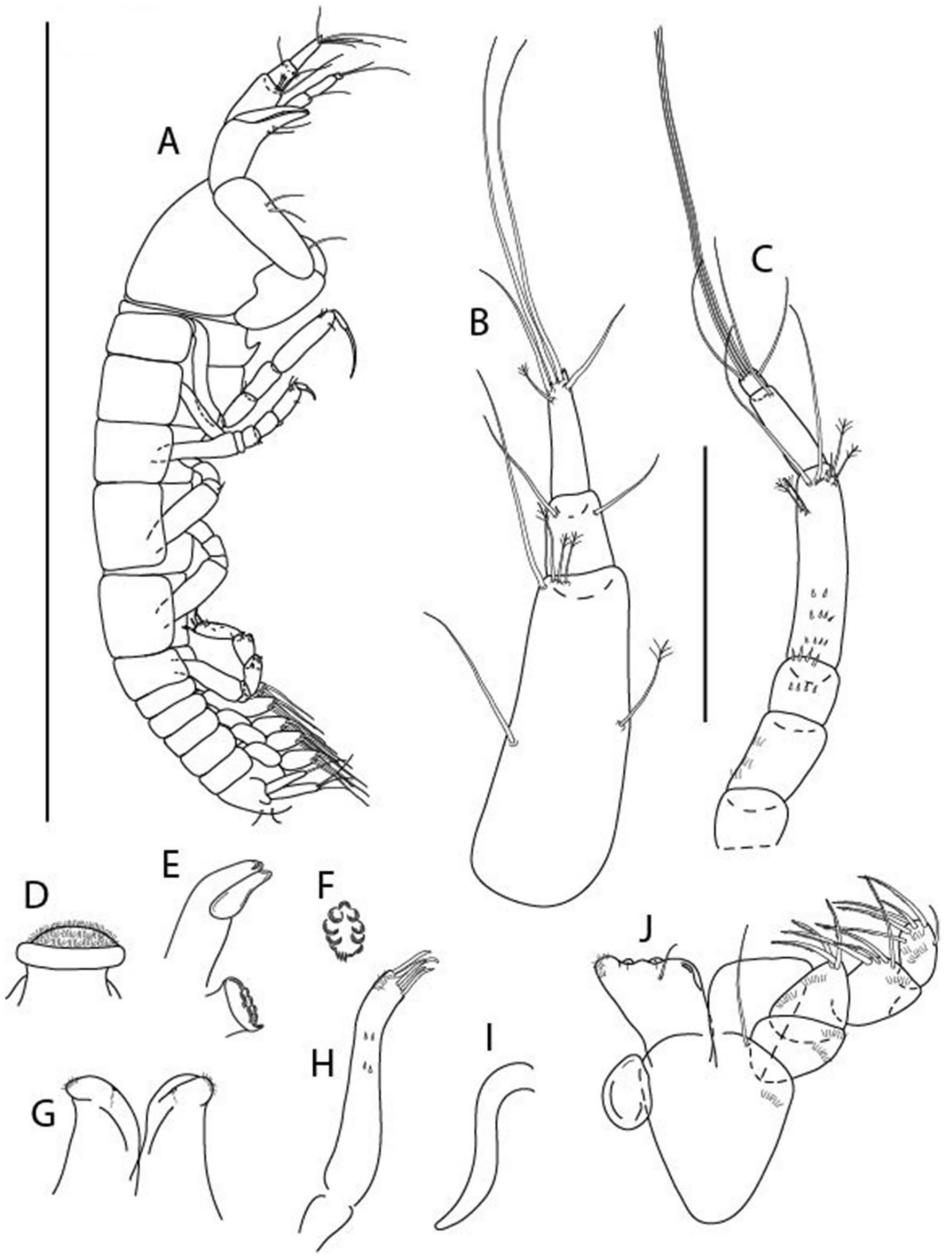
*Brevitanais nagayae* n. sp., neuter, (**A**) Body, lateral view; (**B**) Antennule; (**C**) Antenna; (**D**) Labrum; (**E**) Left mandible; (**F**) Right mandible; (**G**) Labium; (**H**) Maxillule; (**I**) Epignath; (**J**) Maxilla; (**J**) Maxilliped. Scale: A = 1 mm, B–J = 0.1 mm (A = ICUL3366, ZMHK-64337; B–J = ICUL3367, ZMHK-64336).

**Figure 17:**
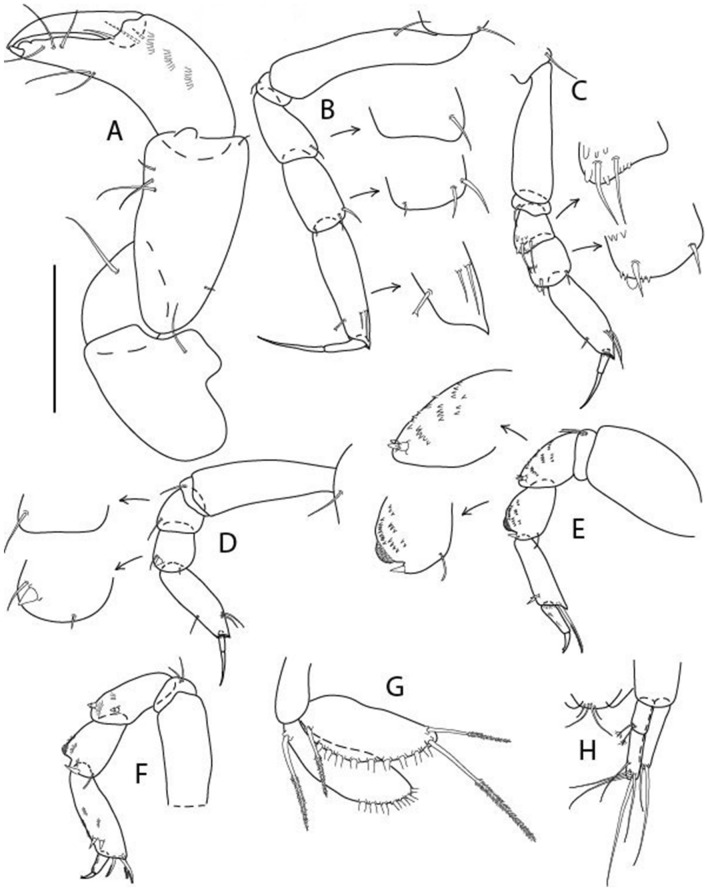
*Brevitanais nagayae* n. sp., neuter, (**A**) Cheliped; (**B**) Pereopod-1; (**C**) Pereopod-2; (**D**) Pereopod-3; (**E**) Pereopod-5; (**F**) Pereopod-6; (**G**) Pleopod; (**H**) Uropod. Scale: A–H = 0.1 mm (A–C, E, H = ICUL3367, ZMHK-64336;, D, F, G = ICUL3366, ZMHK-64337).

- ***Brevitanais nagayae*** **Gellert & Błażewicz n. sp.**
- LSID urn:lsid:zoobank.org:act:24E4827B-9769-4E43-9C24-304ACDBA47CE.
- (Figs. 16, 17).

#### Material examined

Holotype, manca-3 0.7 mm. IceAGE, St. 1010-1 (ICUL13950, ZMHK-64334).

Paratypes IceAGE, St. 1010-1, two manca-3 0.8 mm (ICUL3368, ZMHK-64335); IceAGE, St. 1010-1, manca-3 0.8 mm (ICUL3367, ZMHK-64336; dissected); IceAGE, St. 1032-1, neuter 0.9 mm (ICUL3366, ZMHK-64337; dissected).

#### Diagnosis

Antennule article-1 3.0 L:W. Antenna article-2 1.8 L:W; articles 2–3 naked. Pereopod-1 merus 1.9 L:W, with three setae, propodus with bifurcate ventrodistal seta; pereopod-2 merus 1.1 L:W, with two short inferodistal setae, carpus 1.2 L:W; pereopods 2–3 propodus with ventrodistal seta; pereopod-3 carpus 1.1 L:W. Uropod exopod 0.9× endopod.

#### Etymology

This species is dedicated to Dr. Agnieszka Nagay, Polish rifle shooter, European champion, and Ph.D. in Biological Sciences at University of Lodz (Poland).

#### Description of neuter,

Length 0.9 mm. Body (Fig. 16A) very stout, 3.9 L:W. Cephalothorax trapezoidal, 1.1 L:W, 3.1× pereonite-1, naked. Pereonites 1−6: 0.5, 0.8, 0.7, 0.9, 0.7 and 0.4 L:W, respectively; all pereonites rounded. Pereonite-1 0.6× pereonite-2; pereonite-2 1.1× pereonite-3; pereonite-3 0.7× pereonite-4; pereonite-4 as long as pereonite-5; pereonite-5 2.4× pereonite-6. Pleon 0.2× BL; pleonites 1−5: of similar size – 0.3 L:W. Pleotelson 4.0× pereonite-6.

Antennule (Fig. 16B) 0.9× cephalothorax; article-1 0.6 of antennule length, 2.9 L:W, with PSS on inner margin, seta on outer margin, and long seta (longer as article-2) and three PSS distally; article-2 1.7 L:W, 0.3× article-1, with two long distal setae (longer than half of article-3, one on inner and one on outer margin); article-3 4.6 L:W, 1.4× article-2, with PSS, two short and two long terminal setae.

Antenna (Fig. 16C) article-2 1.7 L:W, with microtrichia along article; article-3 1.1 L:W, 0.7× article-2, with microtrichia at midlength, and four distal spinules; article-4 4.4 L:W, 2.8× article-3, with short, two long (longer than article-5) and four PSS distally, and numerous microtrichia along article; article-5 3.7 L:W, 0.5× article-4, with long seta; article-6 1.0 L:W, with distal five setae.

Mouthparts. Labrum (Fig. 16D) typical. Mandible (Fig. 16E−F) molar typical. Left mandible (Fig. 16E) incisor with two cusps, *lacinia mobilis* well developed, with single cusp; right mandible (Fig. 16F) incisor broken during dissection. Labium (Fig. 16G) typical, outer corner of inner lobe and outer lobe with minute setae. Maxillule (Fig. 16H) typical.

Maxilliped (Fig. 16J) basis 1.4 L:W; endite cusps moderate, with seta in the middle, and microtrichia on outer margin; palp article-1 with numerous microtrichia; articles 2–4 typical, article-4 microtrichia along article. Epignath (Fig. 15I) elongate, shorter than maxillule endite, tip rounded.

Cheliped (Fig. 17A) slender; basis 1.7 L:W, with dorsolateral seta; merus ventral seta long; carpus 2.0 L:W, with two long setae (about half carpus W) and short seta ventrally, dorsal margin setation typical; chela longer than carpus, 2.6 L:W, with two setae (one on outer and two on inner margin) and microtrichia; palm 1.5× fixed finger; fixed finger cutting edge with three very weak, blunt distal cusps; dactylus slightly curved.

Pereopod-1 (Fig. 17B) overall 15.3 L:W; coxa with seta, basis 4.9 L:W, with ventroproximal seta; merus 1.9 L:W, with dorsodistal seta; carpus 1.9 L:W, 1.1× merus, with two dorsodistal setae and ventrodistal seta; propodus 3.4 L:W, 1.4× carpus, with two dorsodistal setae and ventrodistal bifurcate seta; dactylus 0.9× unguis, together as long as propodus.

Pereopod-2 (Fig. 17C) overall 9.4 L:W; basis 3.5 L:W naked; merus 1.1 L:W, with two ventrodistal setae and numerous spinules (enlarged microtrichia) along article; carpus 1.1 L:W, 1.2× merus, with calcified microtrichia, dorsodistal seta and ventrodistal seta, and small conical spine; propodus 3.1 L:W, 1.1× merus and carpus combined, with two dorsodistal setae and ventrodistal seta; dactylus 0.6× unguis, together 0.7× propodus.

Pereopod-3 (Fig. 17D) similar to pereopod-2 but stouter, overall, 7.7 L:W; basis 3.2 L:W, naked; merus 1.1 L:W, with ventrodistal seta; carpus 1.2 L:W, 1.1× merus, without microtrichia; propodus 3.0 L:W, 1.2× carpus; dactylus 0.7× unguis, together 0.6× propodus.

Pereopod-4 the same as pereopod-5.

Pereopod-5 (Fig. 17E) overall 4.9 L:W; basis robust, 1.7 L:W, naked; merus 1.6 L:W, with two serrate distal spines, and numerous calcified microtrichia along article; carpus 1.6 L:W, as long as merus, with moderate prickly tubercles, dorsodistal chemosensory seta, distal spine/crotchet, and calcified microtrichia along article; propodus 3.1 L:W, with two ventrodistal spines, dorsodistal serrate seta longer than dactylus and unguis; dactylus 1.7× unguis, together 0.8× propodus.

Pereopod-6 (Fig. 17F) as pereopod-5, but propodus three dorsodistal setae about as long as or slightly shorter than claw.

Pleopod (Fig. 17G) exopod with eight plumose setae on outer margin; endopod with twelve.

Uropod (Fig. 17H) endopod about 5.0 L:W, proximal article 0.8× distal article, with two distal PSS, distal article with five long terminal setae; exopod 1.9× endopod proximal article, distal setation typical.

#### Distribution

Known from one location off Iceland (Iceland Basin) (Fig. 9) at a depth of 1389 m (this study).

#### Remarks

*Brevitanais nagayae* n. sp. is distinguished from members of *Brevitanais* group-2 by the presence of a ventrodistal seta on the propodus of pereopods 2–3 (spines in *Br. kozakowskae* and *Br. sadleckae*). Furthermore, *Br. nagayae* can be distinguished from *Br. kozakowskae* by three setae on the pereopod-1 carpus (four seta in *Br. kozakowskae*) and a relatively short (1.9 L:W) merus in pereopod-1 (2.9 L:W in *Br. kozakowskae*).

**Figure 18:**
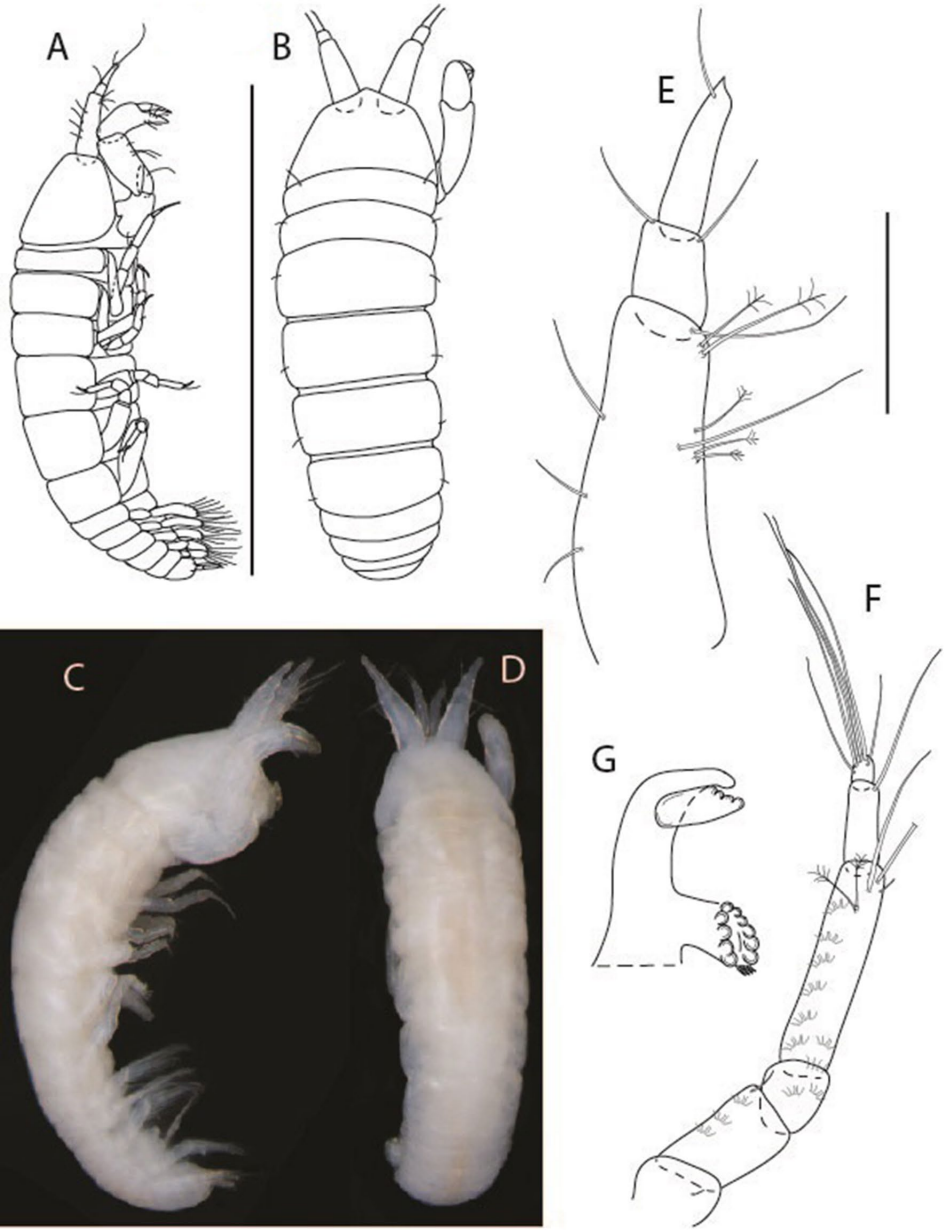
*Brevitanais sadleckae* n. sp., neuter (ICUL3394, ZMHK-64340), (**A**, **C**) Body, lateral view; (**B**, **D**) Body, dorsal view; (**E**) Antennule; (**F**) Antenna; (**G**) Left mandible. Scale: A = 1 mm, B–G = 0.1 mm.

**Figure 19:**
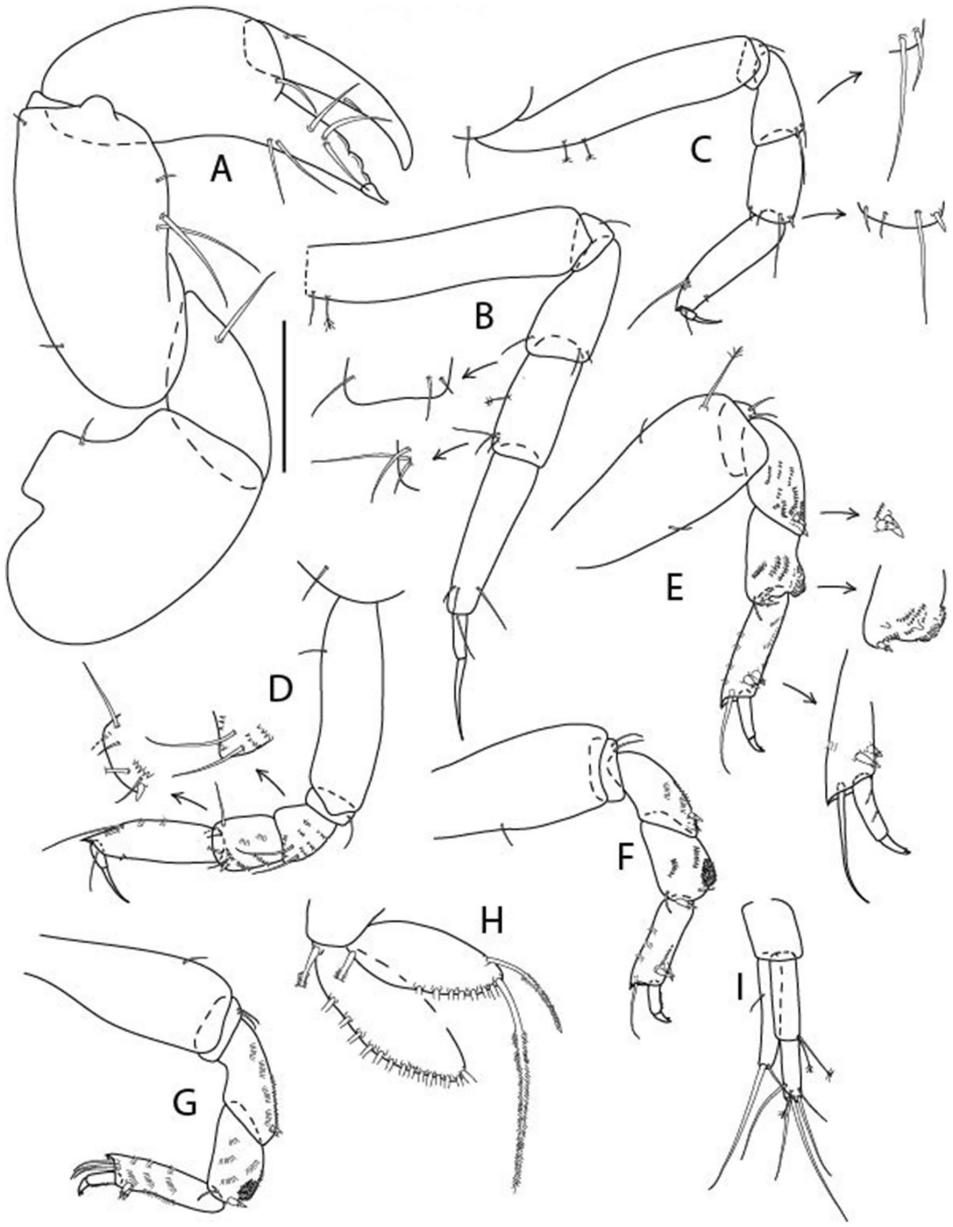
*Brevitanais sadleckae* n. sp., neuter (ICUL3394, ZMHK-64340), (**A**) Cheliped; (**B**) Pereopod-1; C. Pereopod-2; (**D**) Pereopod-3; (**E**) Pereopod-4; (**F**) Pereopod-5; (**G**) Pereopod-6; (**H**) Pleopod; I. Uropod. Scale: A–I = 0.1 mm.

- ***Brevitanais sadleckae*** **Gellert & Błażewicz n. sp.**
  LSID urn:lsid:zoobank.org:act:E8385A0E-8BCD-46F7-8082-E8231070E3C3.
- (Figs. 18, 19).

#### Material examined

Holotype, neuter 1.3 mm, IceAGE, St. 1045-1 (ICUL2129, ZMHK-64338).

Paratypes IceAGE, 1045-1, four mancas-2 and three mancas-3 0.5−0.6 mm (ICUL13954, ZMHK-64339); IceAGE, 1045-1, neuter 0.5 mm (ICUL12663, ZMHK-64341; extraction); IceAGE, 1047-1, neuter 1.2 mm (ICUL3394, ZMHK-64340; dissection).

#### Diagnosis

Antennule article-1 3.3 L:W. Antenna article-2 1.8 L:W; articles 2–3 with short seta. Cheliped carpus 2.1 L:W. Pereopod-1 merus 2.1 L:W, carpus with three setae, propodus with ventrodistal seta; pereopod-2 merus 1.6 L:W, with two (long and short) inferodistal setae, carpus 1.6 L:W; pereopods 2–3 propodus with ventrodistal spine; pereopod-3 carpus 1.9 L:W. Uropod exopod 0.8× endopod.

#### Etymology

This species is dedicated to Magdalena Sadłecka, Polish mountain biker, silver medallist of the World Championships.

#### Description of neuter,

Length 1.0 mm.Body (Fig. 18A−D) very stout, 3.3 L:W. Cephalothorax trapezoidal, 0.6 L:W, 2.4× pereonite-1, naked. Pereonites 1−6: 0.3, 0.3, 0.4, 0.4, 0.3 and 0.2 L:W, respectively; all pereonites rounded. Pereonite-1 0.4× pereonite-2; pereonite-2 0.8× pereonite-3; pereonite-3 1.1× pereonite-4; pereonite-4 1.2× pereonite-5; pereonite-5 2.4× pereonite-6. Pereonites 1−6 with short lateral seta. Pleon 0.2× BL; pleonites 1−5: of similar size – 0.2 L:W. Pleotelson 2.6× pereonite-6.

Antennule (Fig. 18E) 1.1× cephalothorax; article-1, 0.6 of antennule length, 2.8 L:W, with three setae on inner margin, long seta, three PPS on outer margin at midlength, and long seta and two PSS distally; article-2, 1.1 L:W, 0.2× article-1, with two long distal setae (one on outer and one on inner margin); article-3, 3.4 L:W, 1.9× article-2, setae broken.

Antenna (Fig. 18F) article-2 1.7 L:W, with microtrichia along article and distal seta; article-3 1.3 L:W, 0.6× article-2, with microtrichia along article; article-4 4.5 L:W, 3.1× article-3, with microtrichia along article and two long setae (one longer than article-5, one broken) and short and two PSS distally; article-5 3.0 L:W, 0.4× article-4 with long seta; article-6 1.7 L:W, with two short and four long distal setae.

Mouthparts. Left mandible (Fig. 18G) incisor with single cusp, *lacinia mobilis* well developed, with single cusp. Labrum, labium, right mandible, maxillule, maxilliped, maxilla and epignath in very bad condition, not drawn.

Cheliped (Fig. 19A) basis slender; 1.4 L:W, with short dorsolateral seta; merus seta long; carpus 2.1 L:W, with two long setae (shorter than carpus W) and short seta ventrally, dorsal margin setation typical; chela longer than carpus, 1.7 L:W; palm 1.4× fixed finger; fixed finger cutting edge with three weak, blunt distal cusps; dactylus slightly curved, with a short dorsoproximal seta and short seta on inner margin.

Pereopod-1 (Fig. 19B) coxa and part of basis broken during dissection; basis with dorsoproximal seta and PSS; merus 2.1 L:W with two ventrodistal setae and dorsodistal seta; carpus 2.1 L:W; as long as merus, with three dorsodistal setae; propodus 4.2 L:W, 1.4× carpus, with two dorsodistal setae and long ventrodistal seta; dactylus 0.6× unguis, with seta, together 0.7× propodus.

Pereopod-2 (Fig. 19C) overall 11.1 L:W; coxa with seta; basis 5.1 L:W with two PSS; merus 1.6 L:W, with two ventrodistal setae (short and long); carpus 1.9 L:W, 1.2× merus, with two short dorsodistal setae, and long ventrodistal seta and small spine; propodus 3.6 L:W, 0.8× merus and carpus combined, with two long dorsodistal setae (one broken) and ventrodistal spine; dactylus 1.2× unguis, with seta, together 0.2× propodus.

Pereopod-3 (Fig. 19D) similar to pereopod-2 but more slender, overall, 8.5 L:W; basis 3.6 L:W, with dorsoproximal seta; merus 0.9 L:W, with numerous calcified microtrichia along article; carpus 1.3 L:W, 1.4× merus, with numerous calcified microtrichia along article, long and two short setae dorsodistally and two setae and spine ventrodistally; propodus 3.0 L:W, 1.9× carpus; dactylus 0.6× unguis, with seta, together 0.6× propodus.

Pereopod-4 (Fig. 19E) overall 5.5 L:W; basis robust, 2.2 L:W, with two middorsal setae (one on dorsal and one on ventral margin) and ventrodistal PSS; merus 1.9 L:W, with numerous calcified microtrichia along article and two serrate distal spines; carpus 0.8 L:W, 0.9× merus, with numerous microtrichia along article, moderate prickly tubercles, dorsodistal chemosensory seta, distal spine/crotchet, and numerous calcified microtrichia along article; propodus 3.5 L:W, with numerous microtrichia, two ventrodistal serrate spines, and dorsodistal seta longer than claw; dactylus 2.3× unguis, together 0.6× propodus.

Pereopod-5 (Fig. 19F) similar to preopod-4; overall 5.5 L:W; basis 2.1 L:W, with middorsal seta on dorsal margin; merus 2.0 L:W; carpus 1.9 L:W, as long as merus; propodus 3.1 L:W; dactylus 1.8× unguis, together 0.6× propodus; unguis bifurcate.

Pereopod-6 (Fig. 19G) as pereopod-5, but basis with midventral seta; propodus three dorsodistal setae as long as claw.

Pleopod (Fig. 19H) exopod with nine plumose setae on outer margin; endopod with fifteen.

Uropod (Fig. 19I) endopod 5.5 L:W, proximal article 1.4× distal article, with two distal PSS, distal article with PSS and five long terminal setae; exopod 1.3× endopod proximal article, with seta at midlength, other setation typical.

#### Distribution

Known from one location on the shelf of the Reykjanes Ridge (Fig. 9), from 209.4–218.4 m depth (this study).

#### Remarks

*Brevitanais sadleckae* n. sp. is similar to *Br. nagayae* but has three setae on the pereopod-1 carpus, where *Br. kozakowskae* has four. It has the longest antennule article-1 (3.3 L:W), which separates it from *Br. nagayae* (3.0 L:W) and *Br. kozakowskae* (2.3 L:W). Moreover, it has a ventrodistal spine on the propodus of pereopods 2–3 (setae in *Br. nagayae*), and a slender pereopod-3 carpus (1.6 L:W), that is as long as wide in *Br. kozakowskae*.

- ***Brevitanais*** **group-3**

#### Diagnosis

Uropod endopod and exopod biarticulate; exopod 0.8× endopod.

#### Species included

*Brevitanais andeepae* (Błażewicz-Paszkowycz, 2007); *Brevitanais cornutus* (G. O. Sars, 1879).

- ***Brevitanais andeepae*** **(Błażewicz-Paszkowycz, 2007) n. comb.**

#### Synonyms

*Typhlotanais andeepae* Błażewicz-Paszkowycz (2007)^27^: 26–27, 33, 126–131. Segadilha & Serejo (2022)^59^: 21, 27.

#### Diagnosis

Antennule article-1 2.1 L:W; cheliped carpus 2.3 L:W; pereopod-1 merus 2.7 L:W, carpus with three setae; pereopod-2 merus 1.5 L:W; pereopod-3 carpus 1.7 L:W; pereopod-2 carpus 1.4 L:W; pereopods 2–3 propodus with ventrodistal spine.

#### Distribution

Known from the E Weddell Sea, from depths 1030–4931 m^27^.

#### Remarks

The Antarctic *Br. andeepae* can be distinguished from the N Atlantic *Br. cornutus* by its more slender cheliped carpus (2.3 L:W; 1.8 L:W in *Br. cornutus*), and presence of three setae on the pereopod-1 carpus (two in *Br. cornutus*).

- ***Brevitanais cornutus*** **(G. O. Sars, 1879) n. comb.**

#### Synonyms

*Paratanais cornutus*—G.O. Sars (1879)^36^: 431; Lang (1973)^64^: 218, 219.

*Typhlotanais cornutus*—G.O. Sars (1899)^65^: 24, 25, pl. 11; Hansen (1913)^38^: 61; Kussakin and Tzareva (1972)^66^: 238; Błażewicz-Paszkowycz (2007)^27^: 6, 25, 33, 126, 131; Błażewicz-Paszkowycz et al. (2019)^67^: 4; Stępień et al. (2019)^55^: 3; Segadilha and Serejo (2022)^59^: 27.

#### Material examined

AFEN 1996, St. 53727#1, neuter; AFEN 1996, St. 53734#1, female; AFEN 1996, St. 53755#2, neuter; AFEN 1996, St. 53764#2, two neuters; AFEN 1996, St. 53895#1, neuter; BIOFAR, St. 9018, four neuters; NORBI, St. CP11, 19 neuters.

#### Diagnosis

Antennule article-1 2.3 L:W; cheliped carpus 1.8 L:W; pereopod-1 merus 2.1 L:W, carpus with two setae; pereopod-2 merus 1.8 L:W; pereopod-3 carpus 2.2 L:W; pereopod-2 carpus 1.9 L:W; pereopods 2–3 propodus without ventrodistal seta.

#### Distribution

Known from the Norwegian Sea, at a depth of 349.5 m^36^ and three new localities, i.e. the Iceland-Faroe Rise, Tromso, and W Shetland Slope, from depths 300–554 m (this study).

#### Remarks

See remarks for *Br. andeepae.*

### Key for identification of *Brevitanais* neuters


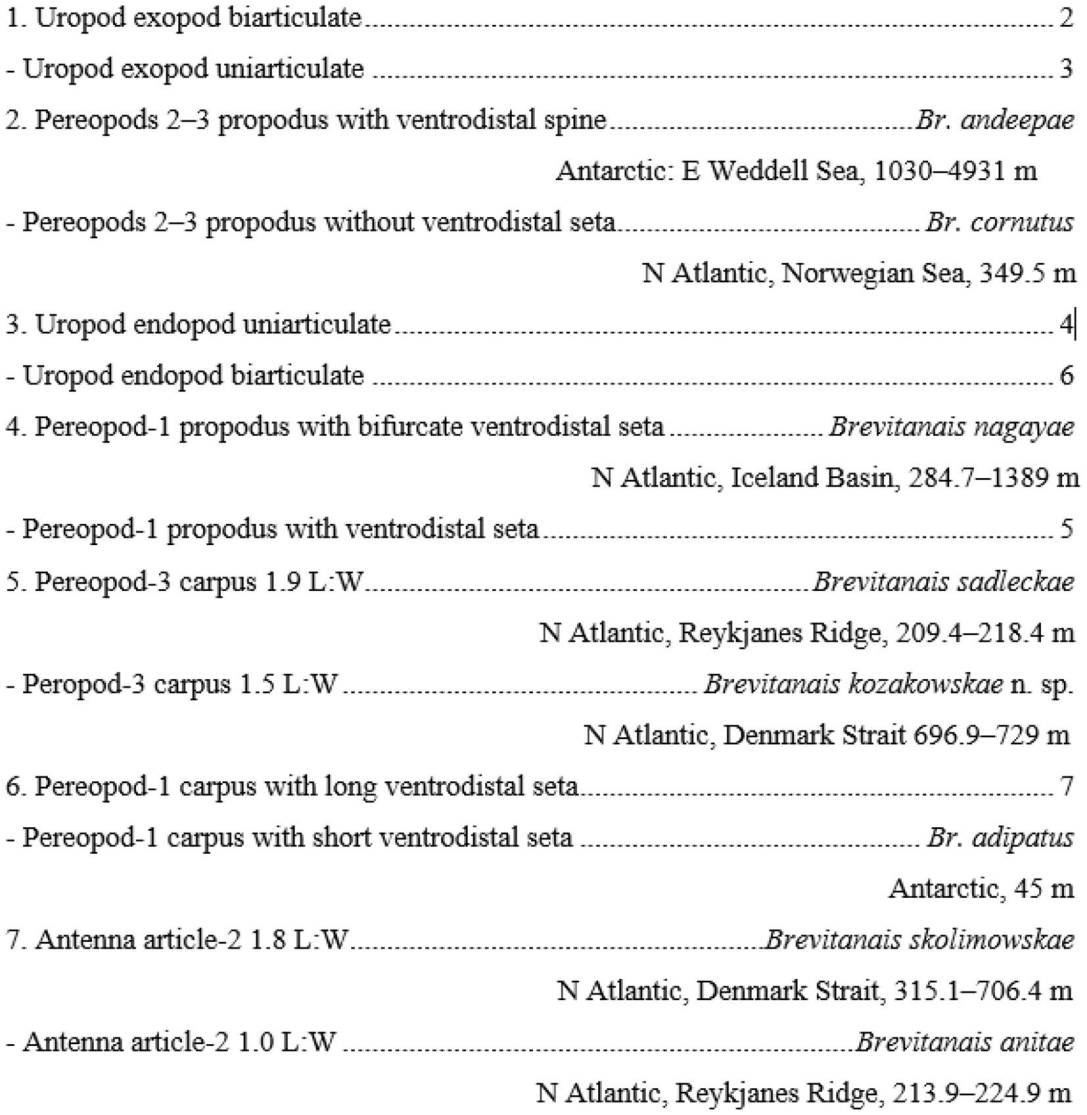


#### Diagnosis

Body short, pereonite margins straight. Antennule article-1 slender (> 4.0 L:W), mesial margin with seta. Maxilliped basis seta longer than endites, endite cusps small. Cheliped carpus short (< 2.5 L:W), carpus with short ventral seta. Pereopod-1 merus L:W slender (> 3.0 L:W), carpus with at least three long setae; pereopods 2–3 carpus ventrodistal seta long, microtrichia regular (if present); pereopods 4–6 carpus with prickly tubercles, propodus distodorsal seta long, unguis bifurcate. Uropod endopod and exopod uniarticulate.

#### Type genus

*Caesatanais igae* n. sp.

#### Etymology

*Caesaires* [Lat.] means long hair, alluding to the presence of uniquely long setae on various the pereopod articles.

#### Species included

*Caesatanais igae* n. sp.; *Caesatanais isiae* n. sp.

#### Remarks

*Caesatanais* n. gen. is defined by the presence of straight-margined pereonites, a long ventrodistal seta on the carpus of pereopods 2–3, and uniarticulate uropod rami. The straight pereonal margins is a unique character for ‘stout-bodied’ typhlotanaids so far, while the long setae on the carpus of pereopods 2–3 is shared with *Hansenotanais*. Nevertheless, members of *Hansenotanais* have a biarticulate uropod endopod. The uniarticulate uropods rami are shared with *Larsenotanais* and *J. bioice* although these both have a simple unguis on pereopods 4–6.

**Figure 20:**
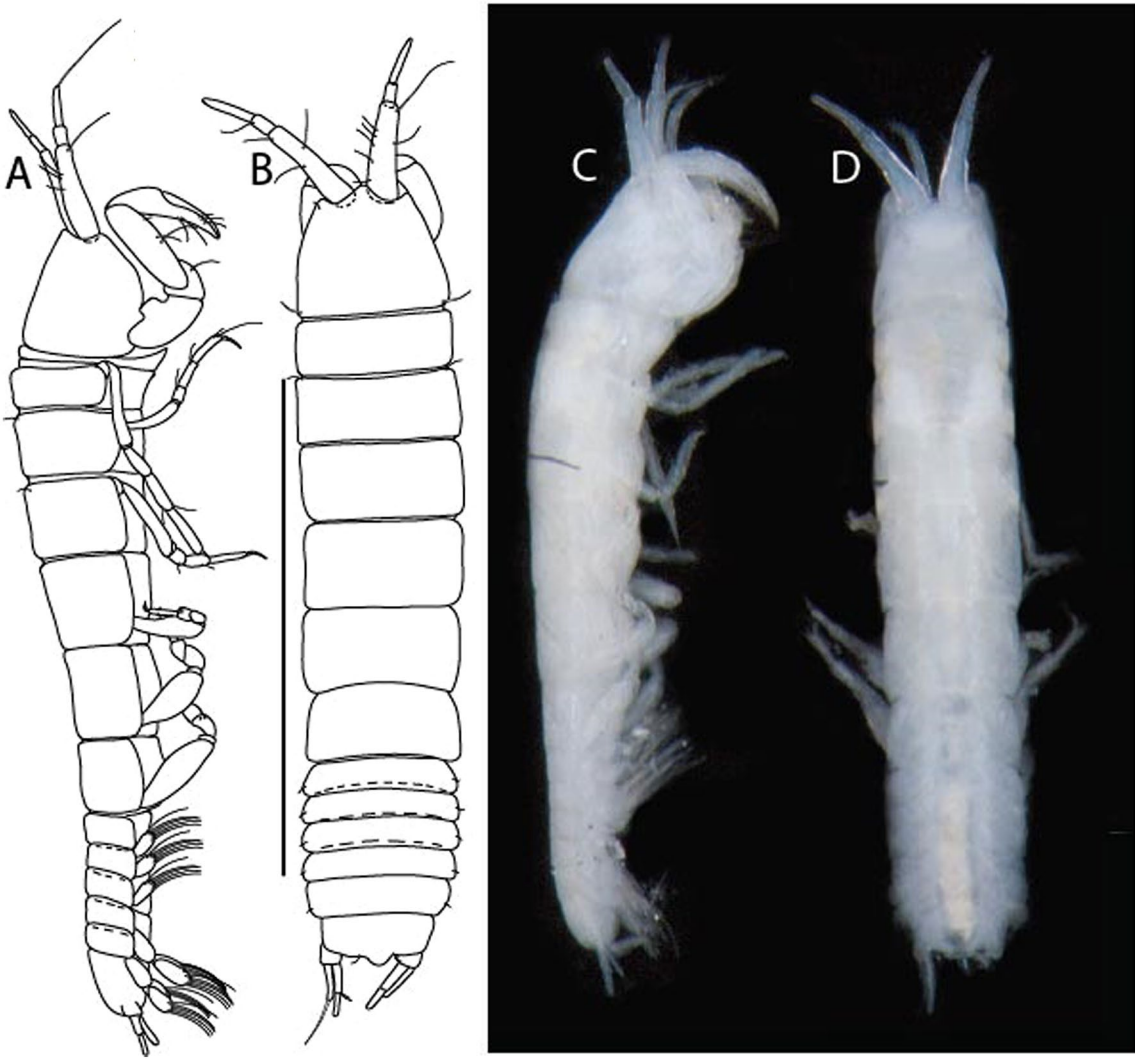
*Caesatanais igae* n. sp., neuter (ICUL13949, ZMHK-64342), (**A**, **C**) Body, lateral view; (**B**, **D**) Body, dorsal view. Scale: A = 1 mm.

**Figure 21:**
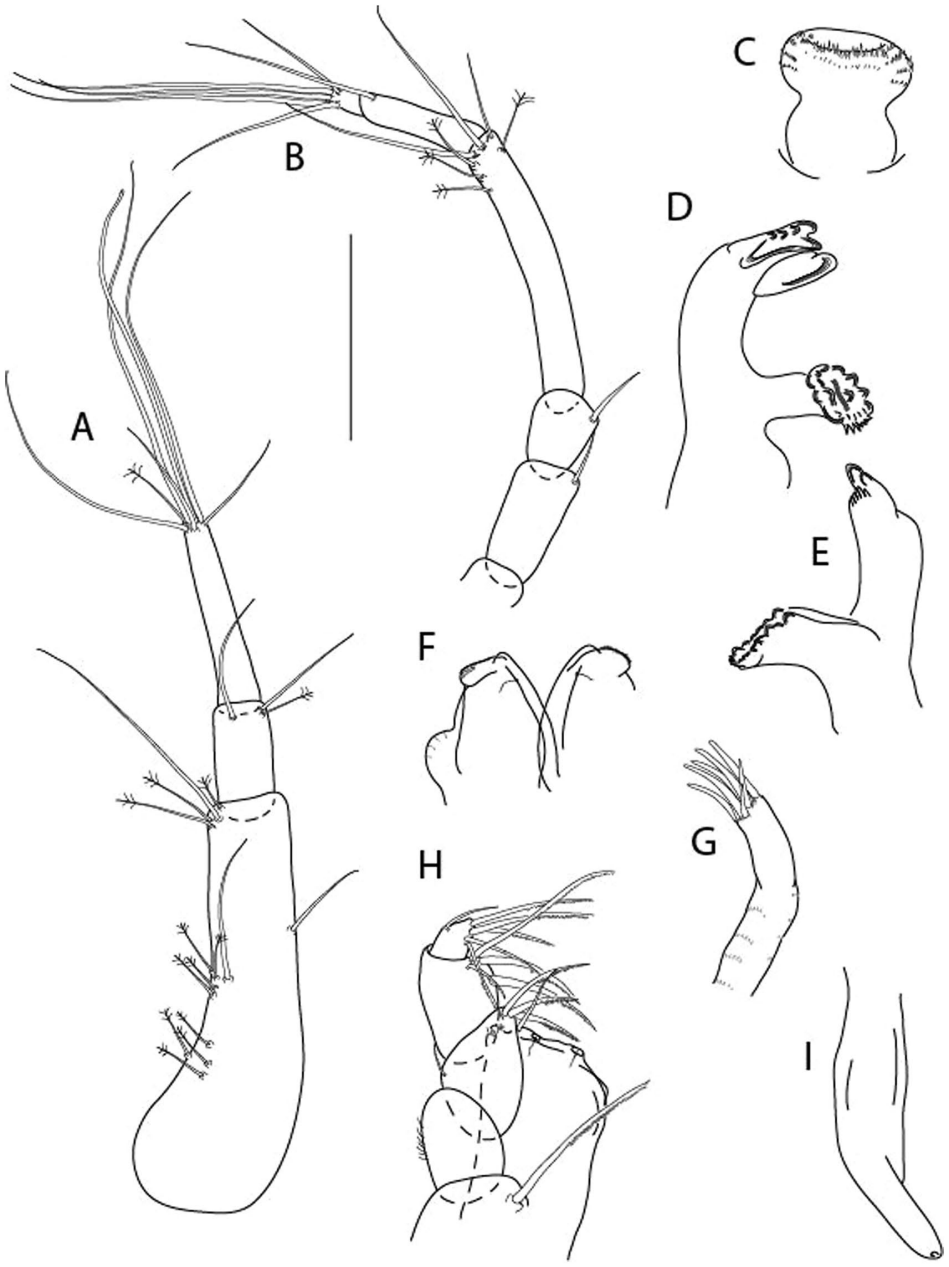
*Caesatanais igae* n. sp., neuter (ICUL13951), (**A**) Antennule; (**B**) Antenna; (**C**) Labrum; (**D**) Left mandible; (**E**) Right mandible; (**F**) Labium; (**G**) Maxillule; (**H**) Maxilliped; (**I**) Epignath. Scale: A–I = 0.1 mm.

**Figure 22:**
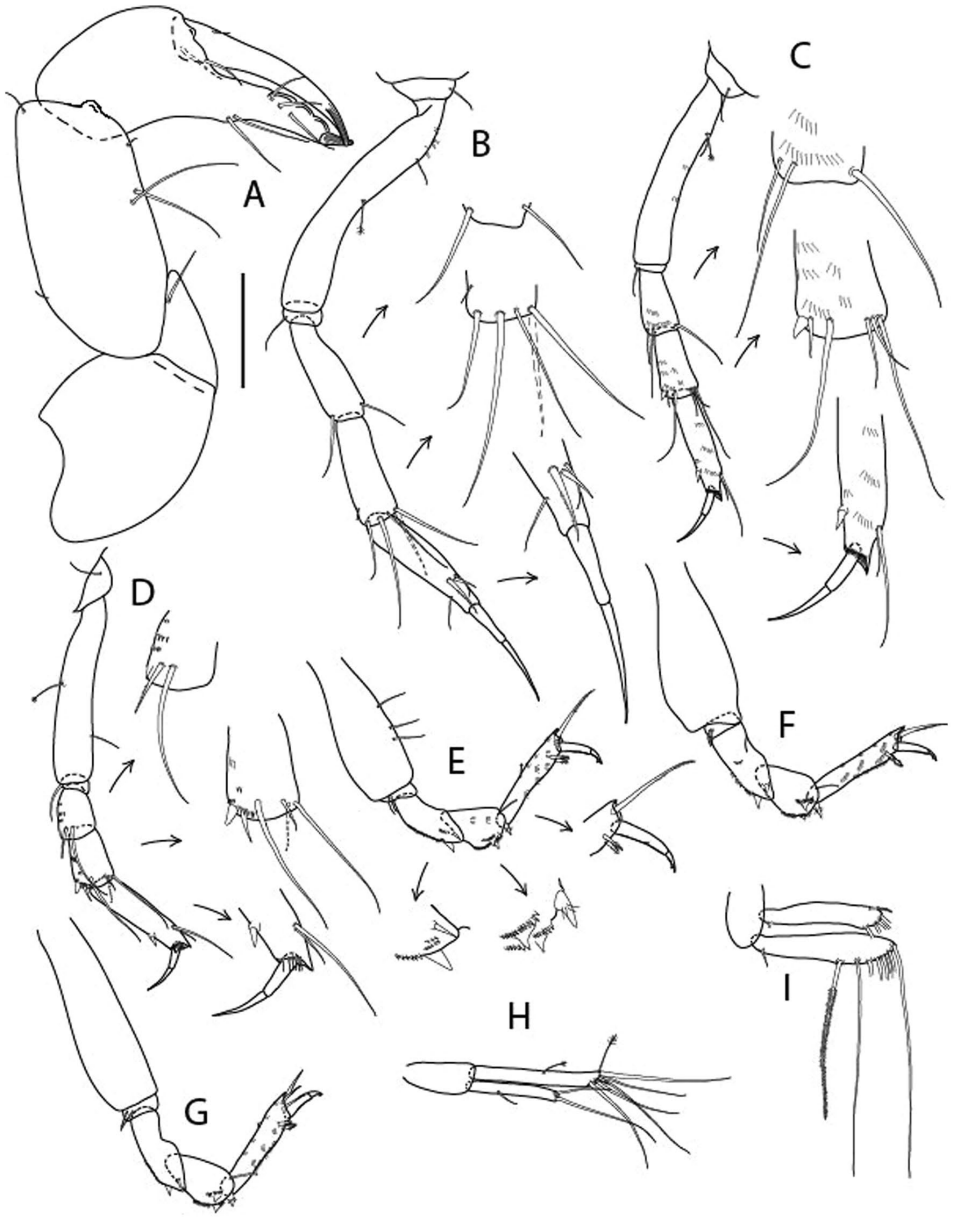
*Caesatanais igae* n. sp., neuter (ICUL13951), A. Cheliped; (**B**) Pereopod-1; (**C**) Pereopod-2; (**D**) Pereopod-3; (**E**) Pereopod-4; (**F**) Pereopod-5; (**G**) Pereopod-6; (**H**) Pleopod; (**I**) Uropod. Scale: A–I = 0.1 mm.

- ***Caesatanais igae*** **n. sp. Gellert, Błażewicz & Bird**
  LSID urn:lsid:zoobank.org:act:7FFE345B-4A1C-4FE8-8F9F-228B620CAC0A.
- (Figs. 20, 21 and 22).

#### Synonyms

*Typhlotanais* sp. L: Holdich & Bird (1985)^68^: 445, Table 1.

#### Material examined

Holotype, neuter 1.5 mm, IceAGE, St. 967-1 (ICUL13949, ZMHK-64342).

#### Paratypes

IceAGE, St. 963-1, manca-2 0.6 mm, neuter 0.9 mm (ICUL8960, ZMHK-64343); IceAGE, St. 967-1, neuter 1.4 mm (ICUL10108, ZMHK-64344; extraction); IceAGE, St. 967-1, neuter 1.5 mm (ICUL13951, ZMHK-64348; dissected); IceAGE, St. 967-1, two neuters 1.5−1.7 mm (ICUL10041, ZMHK-64345; extraction); IceAGE, St. 967-1, two neuters 1.6−1.7 mm (ICUL10045, ZMHK-64346; extraction); IceAGE, St. 967-1, two neuters 1.5−1.7 mm (ICUL10318, ZMHK-64347).

#### Other material

BIOGAS III, St. DS42, neuter; BIOGAS III, St. DS44, neuter; BIOGAS VI, St. DS76, three neuters; BIOGAS VI, St. DS77, two neuters; CHAIN 106, St. 316, 18 neuters; CHAIN 106, St. 318, 18 neuters; CHAIN 106, St. 321, four neuters; CHAIN 106, St. 323, seven neuters; CHAIN 106, St. 326, 16 neuters; DISCOVERY, St. 10112#3, neuter; DISCOVERY, St. 7709#73, six neuters; DISCOVERY, St. 7709#85, neuter; INCAL, St. CP04, two neuters; INCAL, St. CP06, three neuters; INCAL, St. CP07, five neuters; INCAL, St. CP08, two neuters; INCAL, St. CP09, neuter; INCAL, St. CP10, two neuters; INCAL, St. DS05, 18 neuters; INCAL, St. DS06, six neuters; INCAL, St. DS07, neuter; INCAL, St. DS09, 15 neuters; INCAL, St. DS10, neuter; INCAL, St. DS13, neuter; INCAL, St. OS01, two neuters; INCAL, St. OS04, two neuters; INCAL, St. OS07, four neuters; INCAL, St. WS01, five neuters; INCAL St. WS02, 90 neuters; INCAL, St. WS05, neuter; INCAL, St. WS09, neuter; POLYGAS, St. DS20, 16 neuters; POLYGAS, St. DS21, 14 neuters; POLYGAS, St. DS22, six neuters; SARSIA, St. n50, five neuters; SMBA, St. ES10, 194 neuters; SMBA, St. ES172, two neuters; SMBA, St. ES180, four neuters; SMBA, St. ES185, six neuters; SMBA, St. ES190, neuter; SMBA, St. ES207, eight neuters; SMBA, St. ES231, two neuters; SMBA, St. ES27, neuter; SMBA, St. ES283, eight neuters; SMBA, St. ES285, neuter; SMBA, St. ES34, 78 neuters; SMBA, St. ES56, neuter; SMBA, St. ES59, neuter.

#### Diagnosis

Antennule article-1 5.4 L:W, article-2 2.3 L:W. Cheliped carpus 2.2 L:W. Pereopod-1 merus 2.7 L:W; pereopod-2 carpus 2.2 L:W. Pleopods small and narrow.

#### Etymology

This species is dedicated to Iga Świątek – Polish world-champion tennis player.

#### Description of neuter,

Length 1.55 mm. Body (Fig. 20A−D) stout, 4.9 L:W. Cephalothorax trapezoidal, 0.9 L:W, 2.2× pereonite-1, with short lateral seta. Pereonites 1−6: 0.4, 0.4, 0.5, 0.6, 0.5 and 0.5 L:W, respectively; all pereonites rounded. Pereonite-1 0.7× pereonite-2; pereonite-2 0.8× pereonite-3 with short lateral seta; pereonite-3 0.9× pereonite-4; pereonite-4 as long as pereonite-5; pereonite-5 1.1× pereonite-6. Pleon 0.3× BL; pleonites 1−5: of similar size – 0.3 L:W. Pleonites, with short lateral seta. Pleotelson 2.9× pereonite-6.

Antennule (Fig. 21A) 1.4× cephalothorax; article-1, 0.6 of antennule length, 4.5 L:W, with two setae and eight PSS at midlength and long seta (longer than article-2) and three PSS distally; article-2, 1.9 L:W, 0.3× article-1, with seta and PSS on inner margin, and seta on outer margin; article-3, 6.4 L:W, 1.8× article-2, with PSS, two short and three long terminal setae and aesthetasc.

Antenna (Fig. 21B) article-2 2.3 L:W, with distal seta; article-3 1.7 L:W, 0.7× article-2, with seta; article-4 7.2 L:W, 3.0× article-3, with four PSS and three (two longer than article-5) setae distally; article-5 4.9 L:W, 0.5× article-4, with long seta; article-6 1.7 L:W, with five distal setae.

Mouthparts. Labrum (Fig. 21C) typical. Mandible (Fig. 21D−E) molar typical. Left mandible (Fig. 21D) incisor with two cusps, *lacinia mobilis* well developed, with single cusp; right mandible (Fig. 21E) incisor with two cusps. Labium (Fig. 21F) typical, outer corner of inner lobe and outer lobe with minute setae, with cusps. Maxillule (Fig. 21G) endite typical, microtrichia along article.

Maxilliped (Fig. 21H) basis finely serrate seta not reaching beyond endites; endite cusps moderate; palp article-1 with microtrichia along article; articles 2–4 typical. Epignath (Fig. 21I) elongate, tip rounded.

Cheliped (Fig. 22A) slender; basis 1.7 L:W, naked; merus (seta about merus W); carpus 2.1 L:W, with two long setae and short seta ventrally, dorsal margin setation typical; chela longer than carpus, 2.7 L:W, with two setae (one in inner and one in outer margin); palm 0.8× fixed finger; fixed finger cutting edge with three weak, distal blunt cusps; dactylus slightly curved, with seta.

Pereopod-1 (Fig. 22B) overall 17.2 L:W; coxa with seta, basis 6.2 L:W, with dorsoproximal PSS, seta and numerous spinules; merus 2.7 L:W, with dorsodistal and ventrodistal setae; carpus 2.9 L:W, as long as merus, with six distal setae (five as long as half of propodus or longer); propodus 4.3 L:W, 1.1× carpus, with three dorsodistal (one serrate and two simple) setae and ventrodistal seta; dactylus 0.7× unguis, together 0.9×.

Pereopod-2 (Fig. 22C) overall 14 L:W; coxa with seta; basis 5.6 L:W, with single dorsoproximal PSS and seta, and microtrichia along article; merus 2.1 L:W, with two ventrodistal setae (one as long as carpus) and dorsodistal seta (as long as carpus) and microtrichia along article; carpus 2.3 L:W, 1.1× merus, with three dorsodistal (one longer than half of propodus) and two ventrodistal setae (one longer than the other) setae, spine and microtrichia along article; propodus 4.6 L:W, 0.8× merus and carpus combined, with two dorsodistal setae and ventrodistal spine and microtrichia along article; dactylus 0.7× unguis, together 0.6× propodus.

Pereopod-3 (Fig. 22D) similar to pereopod-2 but stouter, overall, 11.7 L:W; basis 4.8 L:W, with middorsal and midventral setae; merus 1.5 L:W, with two ventrodistal setae; carpus 2.5 L:W, 1.1× merus, with five setae (two long) and spine distally, and microtrichia along article; propodus 3.8 L:W, 1.7× carpus; dactylus 0.8× unguis; together 0.6× propodus.

Pereopod-4 (Fig. 22E) overall 6.8 L:W; basis robust, 2.6 L:W, with three middorsal setae; merus 2.7 L:W, with two distal spines, and microtrichia along article; carpus 1.6 L:W, 1.1× merus, with prickly tubercles, dorsodistal chemosensory seta, and distal spine/crotchet; propodus 5.7 L:W, 1.7× carpus, with middorsal PSS, two ventrodistal serrate spines, and dorsodistal long serrate seta longer than claw, and microtrichia along article; dactylus 2.1× unguis, together 0.5× propodus.

Pereopod-5 (Fig. 22F) similar to pereopod-4 but overall 6.4 L:W; basis naked; merus 3.2 L:W; carpus 1.6 L:W, 0.7× merus; propodus 6.1 L:W; dactylus 2.4× unguis, together 0.4× propodus.

Pereopod-6 (Fig. 22G) as pereopod-5; propodus three dorsodistal setae shorter than claw.

Pleopod (Fig. 22H) relatively small compared to pleon, rami slender about 5.0–7.0 L:W exopod with five plumose setae on outer margin; endopod with eight.

Uropod (Fig. 22I) endopod 10 L:W, with PSS seta at midlength, and two PSS and five long terminal setae; exopod 0.7× endopod, with seta at midlength, other setation typical.

#### Distribution

Known from eight locations in the N Atlantic, i.e. N Biscay, Abyssal Porcupine, Biscay Abyssal Plain, Iceland Basin, Porcupine Abyssal Plain, Porcupine Seabright, Rockall Trough and S Biscay (Fig. 5) from depths 2191–4823 m (this study).

#### Remarks

*Caesatanais igae* n. sp. is distinguished from *Caesatanais isiae* (see below) by the presence of proportionately small pleopods (regular in *C. isiae*) and a slender antennule article-1 5.4 L:W (4.1 L:W in *C. isiae*).

**Figure 23:**
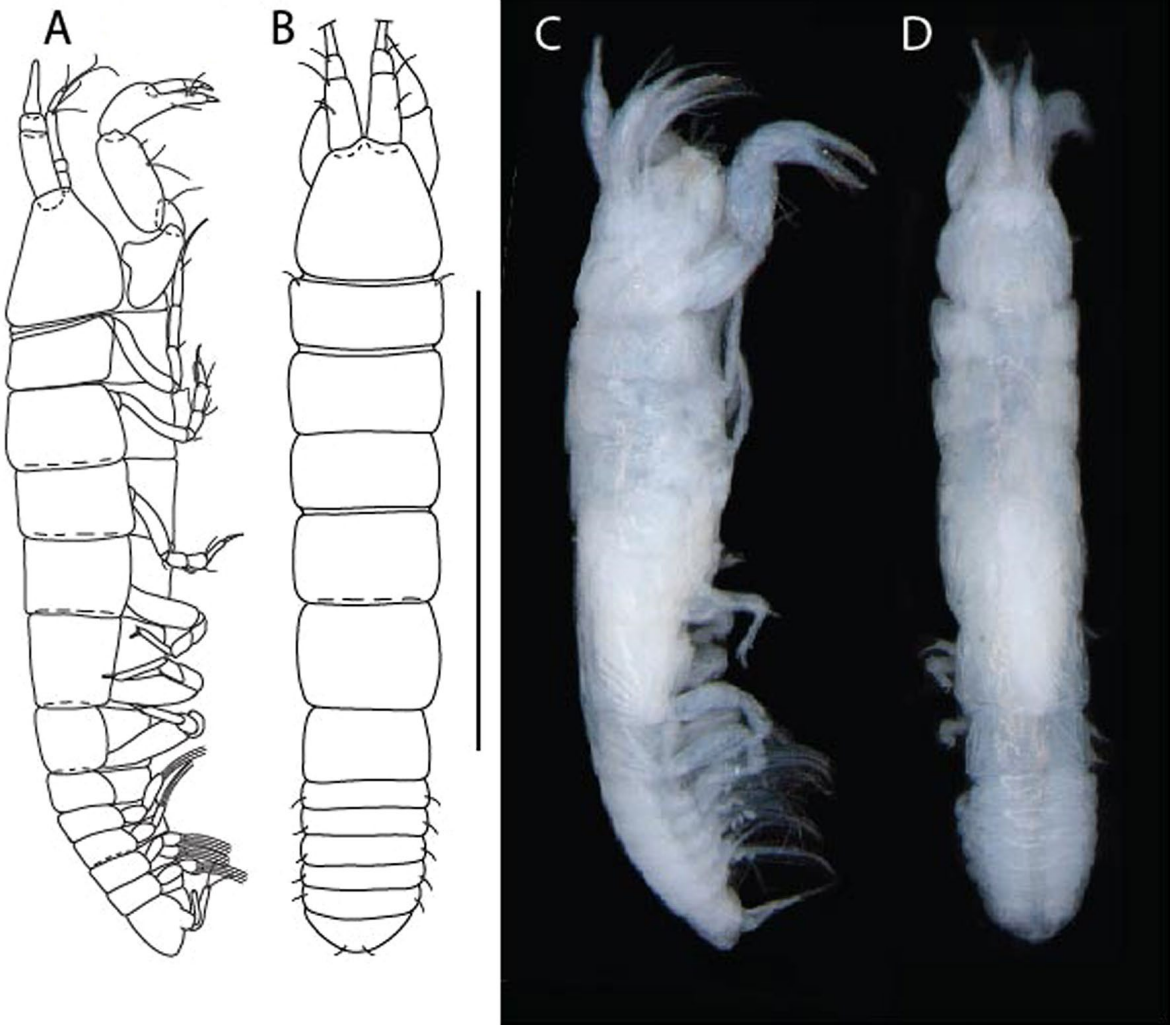
*Caesatanais isiae* n. sp., neuter (ICUL3360, ZMHK-64349), (**A**, **C**) Body, lateral view; (**B**, **D**) Body, dorsal view. Scale: A = 1 mm.

**Figure 24:**
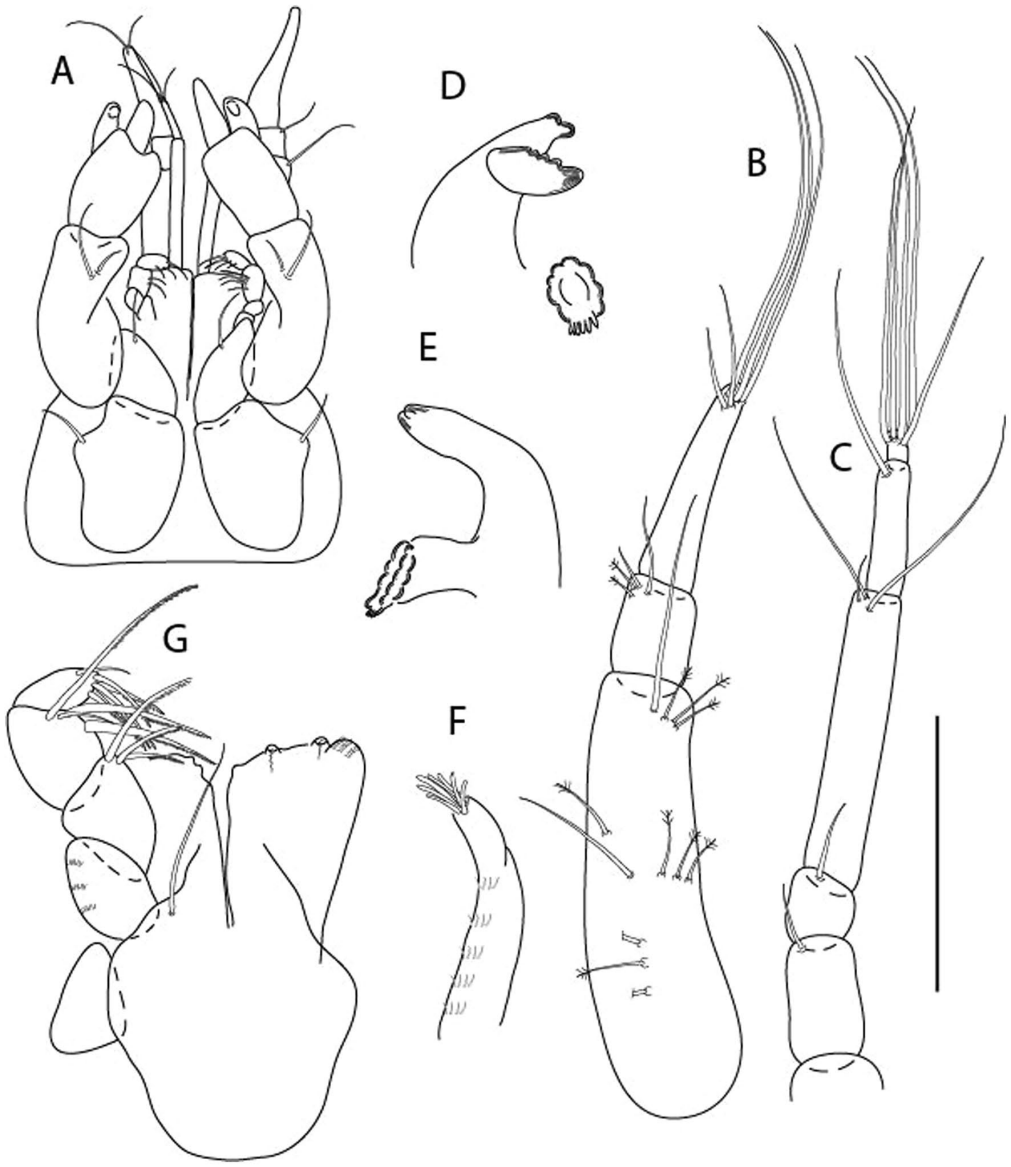
*Caesatanais isiae* n. sp., neuter (ICUL3359, ZMHK-64354), (**A**) Carapace ventral side; (**B**) Antennule; (**C**) Antenna; (**D**) Left mandible; (**E**) Right mandible; (**F**) Maxillule; (**G**) Maxilliped. Scale: B–F = 0.1 mm.

**Figure 25:**
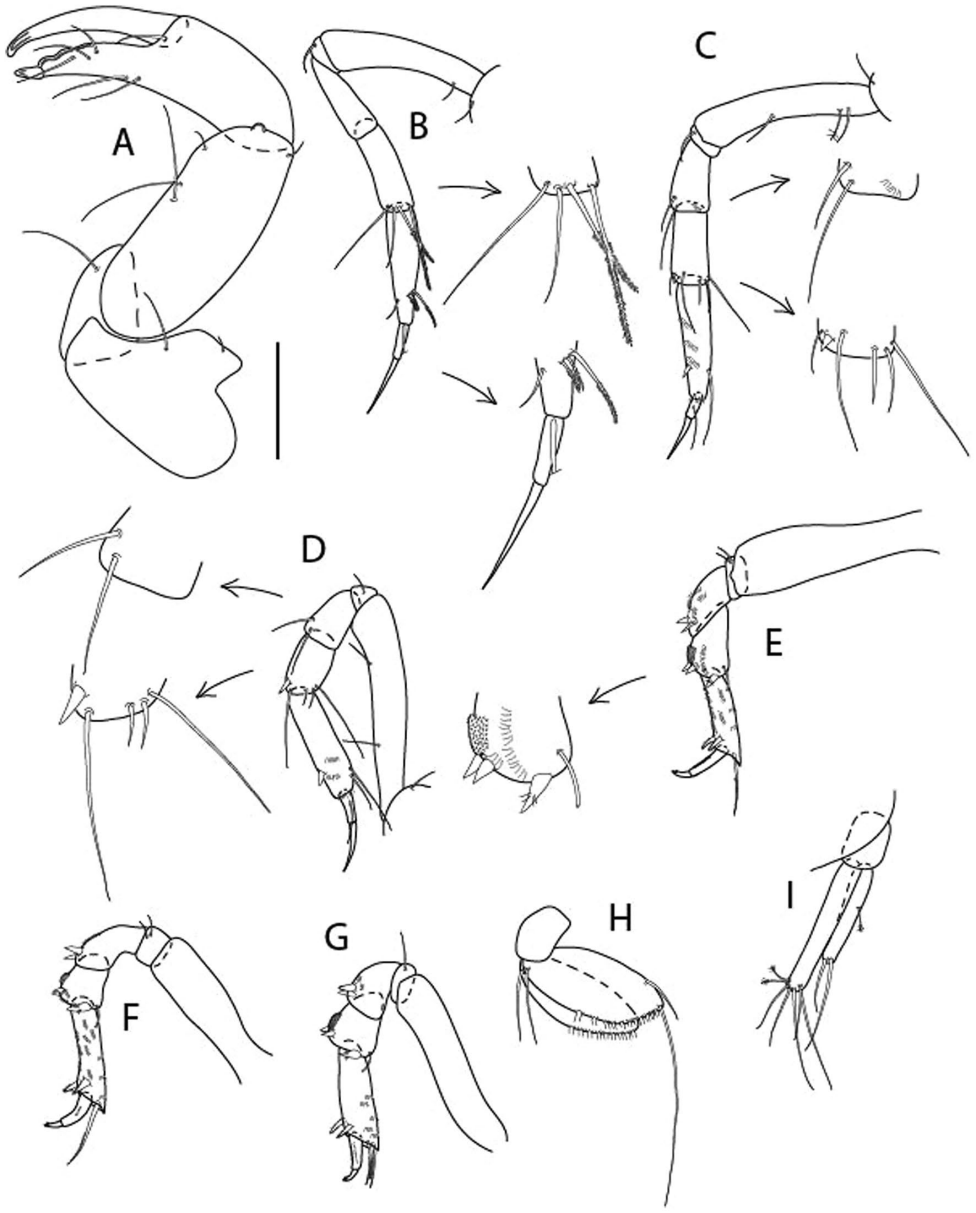
*Caesatanais isiae* n. sp., neuter (ICUL3359, ZMHK-64354), (**A**) Cheliped; (**B**) Pereopod-1; (**C**) Pereopod-2; (**D**) Pereopod-3; (**E**) Pereopod-4; (**F**) Pereopod-5; (**G**) Pereopod-6; (**H**) Pleopod; (**I**) Uropod. Scale: A–I = 0.1 mm.

- ***Caesatanais isiae*** **Gellert & Błażewicz n. sp.**
- LSID urn:lsid:zoobank.org:act:AB9A52DD-3B78-4CD8-BC04-E8DCC27B92CF.
- (Figs. 23, 24 and 25).

#### Material examined

Holotype, neuter 1.5 mm, IceAGE, St. 963-1 (ICUL3360, ZMHK-64349).

Paratypes IceAGE, St. 967-1, neuter 1.3 mm (ICUL3363, ZMHK-64350); IceAGE, St. 967-1, neuter 1.5 mm (ICUL3362, ZMHK-64351); IceAGE, St. 967-1, neuter (ICUL3361, ZMHK-64352; broken); IceAGE, St. 967-1, neuter 1.8 mm (ICUL3359, ZMHK-64354; dissected); IceAGE, St. 967-1, neuter 2 mm (ICUL3084, ZMHK-64353).

#### Diagnosis

Antennule article-1 4.1 L:W, article-2 1.8 L:W. Cheliped carpus 2.5 L:W. Pereopod-1 merus 3.5 L:W. Pereopod-2 carpus 2.1 L:W. Pleopods normal shape (not reduced).

#### Etymology

This species is dedicated to Agnieszka Radwańska “Isia”, Polish tennis player, UNICEF goodwill ambassador.

#### Description of neuter,

Length 1.7 mm. Body (Fig. 23A−D) moderately stout, 5.4 L:W. Cephalothorax trapezoidal, 1.0 L:W, 4.0× pereonite-1, naked. Pereonites 1−6: 0.4, 0.5, 0.5, 0.6, 0.8 and 0.6 L:W, respectively; all pereonites rounded. Pereonite-1 0.7× pereonite-2, with short lateral seta; pereonite-2 0.9× pereonite-3; pereonite-3 0.9× pereonite-4; pereonite-4 0.8× pereonite-5; pereonite-5 1.6× pereonite-6. Pereonites 1−6 with short lateral seta. Pleon 0.1× BL; pleonites 1−5: of similar size – 0.2 L:W. Pleotelson 2.4× pereonite-6.

Antennule (Fig. 24B) 1.0× cephalothorax; article-1, 0.6 of antennule length, 3.9 L:W, with seta and seven PSS (two broken) at midlength, and long seta (longer than article-2) and three PSS distally; article-2 1.4 L:W, 0.2× article-1, with two distal setae and two distal PSS on inner margin; article-3 6.2 L:W, 2.1× article-2, with two short and three long terminal setae.

Antenna (Fig. 24C) article-2 1.9 L:W, with distal seta; article-3 1.2 L:W, 0.6× article-2, with distal seta; article-4, 5.0 L:W, 3.9× article-3 with short and two long (longer than article-5) setae distally; article-5 4.4 L:W, 0.5× article-4, with long seta; article-6 1.8 L:W, with five distal setae.

Mouthparts. Mandible (Fig. 24D−E) molar typical. Left mandible (Fig. 24D) incisor with two cusps, *lacinia mobilis* well developed, with single cusp; right mandible (Fig. 24E) incisor with two cusps. Maxillule (Fig. 24F) typical, microtrichia along article. Maxilla (Fig. 24G) triangular. Labrum and labium lost during dissection.

Maxilliped (Fig. 24G) basis 1.1 L:W, with seta reaching beyond endites; endite cusps moderate, microtrichia on outer margin; palp article-1 with numerous microtrichia along article; articles 2–4 typical. Epignath lost during dissection.

Cheliped (Figs. 24A, 25A) slender; basis 2.0 L:W, with two dorsolateral setae, one short, one long; merus seta long; carpus 2.5 L:W, with two long setae (as long as carpus W) and short seta ventrally, dorsal margin setation typical; chela longer than carpus, 2.0 L:W, with seta; palm 1.2× fixed finger; fixed finger cutting edge with three very weak, distal blunt cusps; dactylus slightly curved, with seta.

Pereopod-1 (Fig. 25B) overall 15.5 L:W; coxa with seta, basis 4.2 L:W, with dorsoproximal seta; merus 3.5 L:W, naked; carpus 3.1 L:W, 1.1× merus, with two setae and two serrate setae (longer than half of propodus) distally; propodus 4.0 L:W, 1.2× carpus, with three dorsodistal serrate setae and ventrodistal seta (broken); dactylus 0.8× unguis, with seta (broken), 0.8× propodus.

Pereopod-2 (Fig. 25C) overall 15.3 L:W; coxa with seta; basis 5.4 L:W with single dorsoproximal seta and PSS, and middorsal seta; merus 2.0 L:W, with two ventrodistal setae; carpus 2.3 L:W, 1.3× merus, with five setae (two as long as half of propodus) and spine distally; propodus 4.7 L:W, 0.8× merus and carpus combined, with dorsodistal long seta and ventrodistal spine, and microtrichia along article; dactylus 0.5× unguis, with seta, together 0.6× propodus.

Pereopod-3 (Fig. 25D) similar to pereopod-2 but stouter, overall, 12.8 L:W; basis 5.1 L:W; merus 1.8 L:W; carpus 1.8 L:W, 1.1× merus, with four setae (two as long as half of propodus) and spine distally; propodus 4.3 L:W, 1.7× carpus, with numerous microtrichia along article, two dorsodistal setae and ventrodistal spine; dactylus 0.7× unguis, with seta, together 0.8× propodus.

Pereopod-4 (Fig. 25E) overall 7.9 L:W; basis robust, 3.8 L:W, naked; merus 2.1 L:W, with numerous microtrichia along article and distal two spines; carpus 2.0 L:W, 1.4× merus, with prickly tubercles, dorsodistal chemosensory seta, three distal spines/crotchets, with numerous microtrichia along article; propodus 4.3 L:W, with numerous microtrichia, two ventrodistal serrate spines, and dorsodistal serrate seta as long as claw; dactylus 3.0× unguis, together 0.7× propodus.

Pereopod-5 (Fig. 25F) similar to pereopod-4 but overall 8.7 L:W; basis robust, 3.5 L:W; merus 2.7 L:W; carpus 1.7 L:W, 0.7× merus; propodus 5.4 L:W; dactylus 2.6× unguis, together 0.5× propodus.

Pereopod-6 (Fig. 25G) as pereopod-5; propodus three dorsodistal serrate setae L.

Pleopod (Fig. 25H) exopod with ten setae on outer margin; endopod with fourteen.

Uropod (Fig. 25I) endopod 6.5 L:W, with two PSS and five long terminal setae; exopod 0.7× endopod, with a PSS at midlength, the other setation typical.

#### Distribution

Known from one location off Iceland (Iceland Basin) (Fig. 5), at depths 2747–2750.4 m  (this study).

#### Remarks

Oval pleopods separate *Caesatanais isiae* n. sp. from *C. igae*, which has small and narrow pleopodal rami. *Caesatanais igae* has an antennule article-1 5.4 L:W, while in *C. isiae* it is a slightly stouter (4.1 L:W).

- ***Egregiella*** **Gellert, Błażewicz & Bird n. gen.**
- LSID urn:lsid:zoobank.org:act:35388ACC-59A3-4257-9CF2-16EAF603CE8D.

#### Diagnosis

Body stout, pereonite margins rounded. Antennule article-1 long (5.4 > L:W), mesial margin with three setae. Maxilliped basis seta shorter than endites, endite cusps small. Cheliped carpus slender (> 2.5 L:W), carpus with short ventral seta. Pereopod-1 merus L:W slender (> 3.0 L:W), carpus without long seta; pereopods 2–3 carpus ventrodistal seta short, microtrichia calcified; pereopods 4–6 carpus with prickly tubercles, propodus distodorsal seta short, unguis simple. Uropods long and slender, endopod biarticulate, exopod biarticulate.

#### Type species

*Egregiella eximia* (Hansen, 1913) (by monotypy).

#### Species included

*Egregiella eximia* (Hansen, 1913) n. comb.

#### Etymology

*Egregie* [Lat.] means magnificent, which alluding to the delight Hansen surely felt when he described the species, and which is captured in the species name (*eximius* [Lat.] means *super*).

#### Remarks

The peculiar body habitus of *Egregiella eximia* and a unique set of morphological features are sufficient to define a new genus-level morphological group for this species. It has a very long and slender antennule article-1 (> 5.5 L:W; often much shorter then 4.0 L:W in most ‘stout-bodied’ typhlotanaids) and elongate, biarticulate uropod rami. From the all ‘stout-bodied’ typhlotanaids, this type of uropod is present in *Brevitanais* group-3, although all *Brevitanais* species have bifurcate unguis in pereopods 4–6, which is simple in *E. eximia*. Similarly, the long antennule article-1 is observed in several ‘stout-bodied’ typhlotanaids e.g.: *Hansenotanais*, *Caesatanais*, *Sarsotanais* and *Stuttotanais* (see below) but again they have (with exception of *Stuttotanais*) a bifurcate pereopods 4–6 unguis. *Stuttotanais*, has uniarticulate uropod exopods, excluding it from the group.

**Figure 26:**
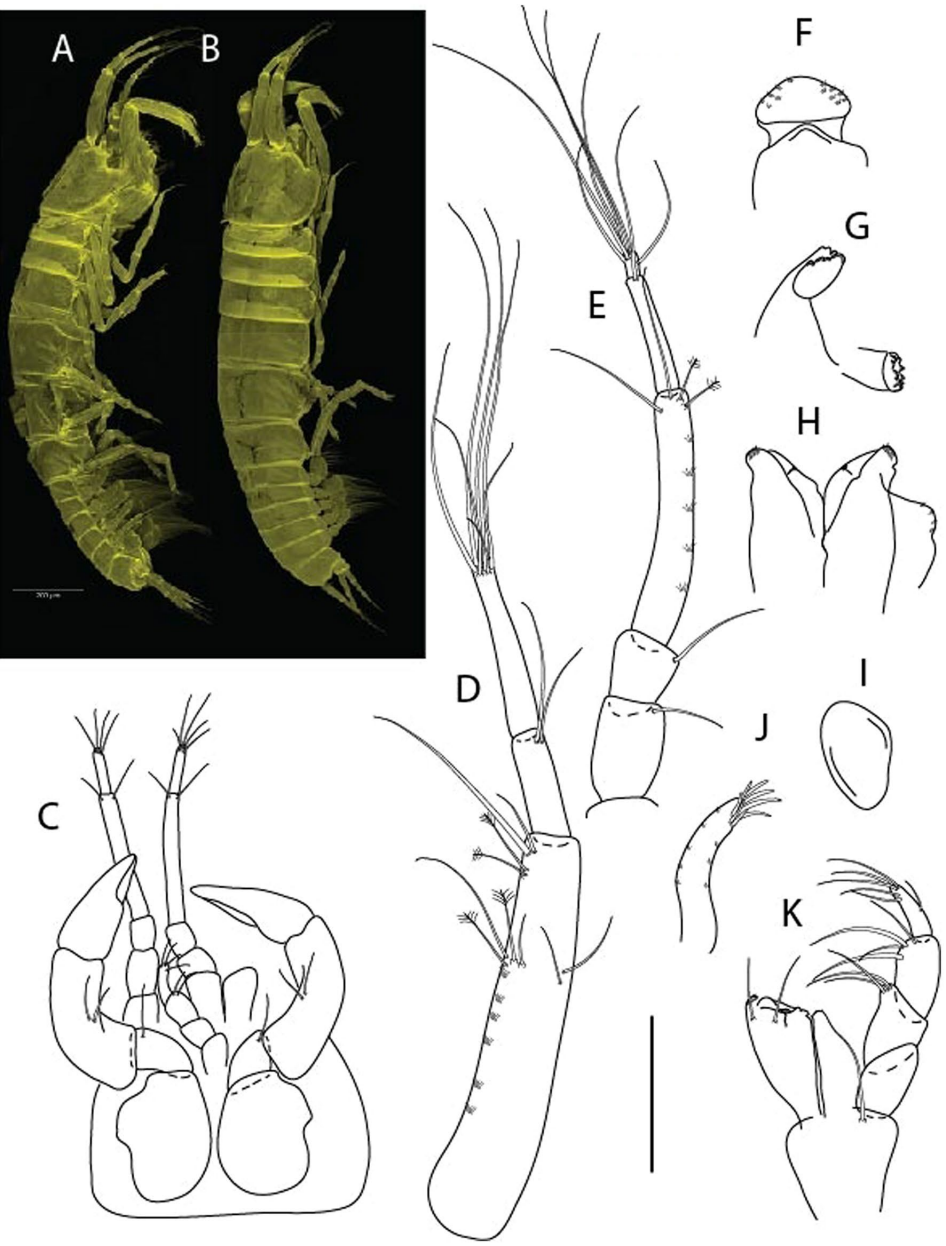
*Egregiella eximia* (Hansen, 1913), neuter (CRU 6380), (**A**) Body, lateral view; (**B**) Body, dorsal view; (**C**) Carapace ventral side; (**D**) Antennule; (**E**) Antenna; (**F**) Labrum; (**G**) Left mandible; (**H**) Labium; (**I**) Maxilla; (**J**) Maxillule; (**K**) Maxilliped. Scale: A = 1 mm, B–J = 0.1 mm.

**Figure 27:**
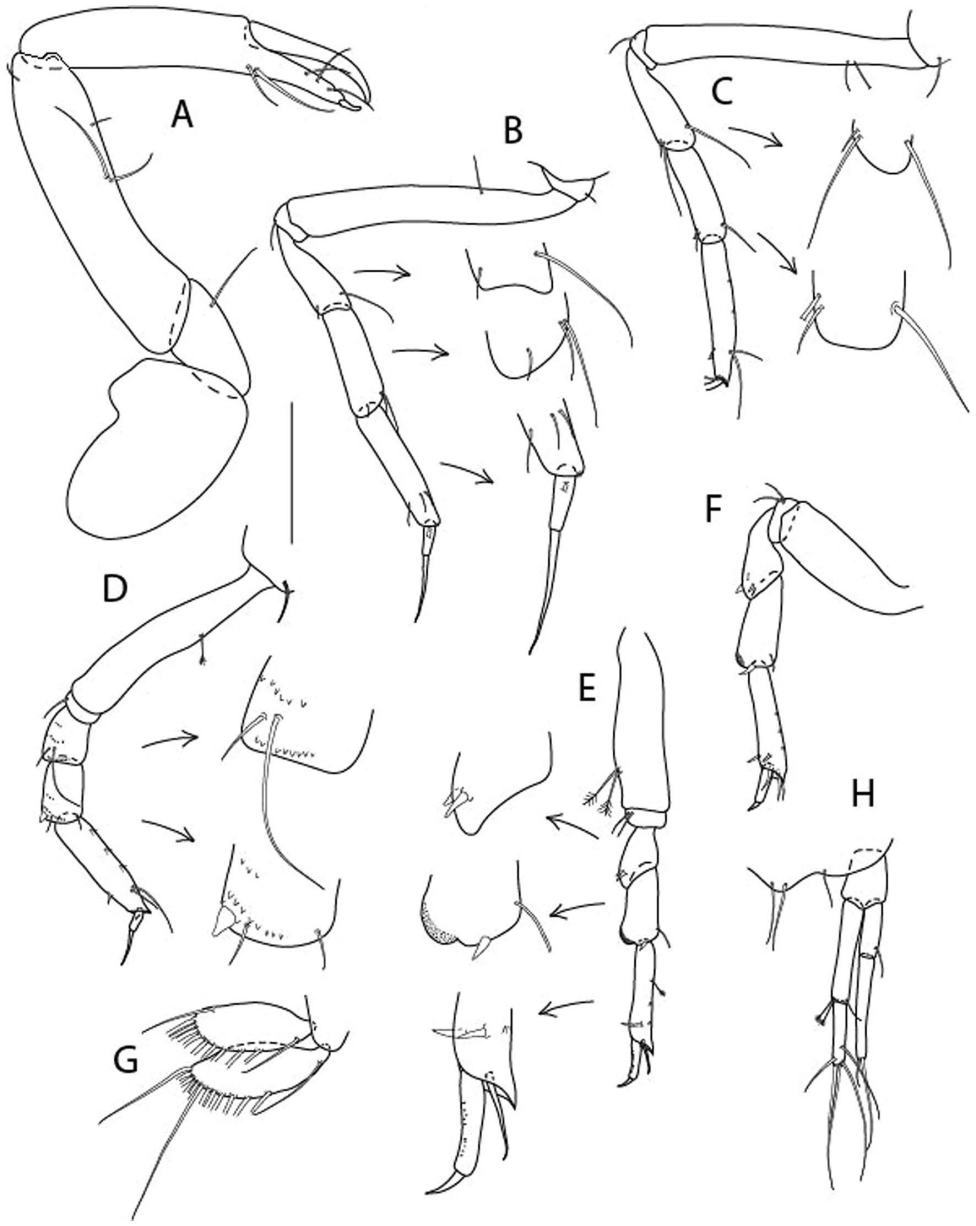
*Egregiella eximia* (Hansen, 1913), neuter (CRU 6380), (**A**) Cheliped; (**B**) Pereopod-1; (**C**) Pereopod-2; (**D**) Pereopod-3; (**E**) Pereopod-5; (**F**) Pereopod-6; (**G**) Pleopod; (**H**) Uropod. Scale: A–I = 0.1 mm.

- ***Egregiella eximia*** **(Hansen, 1913) n. comb.**
- *Typhlotanais eximius*—Hansen, 1913^38^: 44–45; Larsen (2005)^60^: 216; Błażewicz-Paszkowycz, 2007^27^: 132.
- ù(Figs. 26, 27).

#### Material examined

Holotype, neuter 1.3 mm, the Danish Ingolf-Expedition, St. 78.

Paratypes neuters 0.8−1.1 mm, the Danish Ingolf-Expedition, St. 78.

#### Diagnosis

As for ‘eximius’ group.

#### Description of neuter,

Length 1.3 mm. Body (Fig. 25A, B) moderately stout, 5.2 L:W. Cephalothorax trapezoidal, 0.9 L:W, 3.2× pereonite-1. Pereonites 1−6: 0.3, 0.4, 0.5, 0.7, 0.4 and 0.6 L:W, respectively; all pereonites rounded. Pereonite-1 0.6× pereonite-2; pereonite-2 0.9× pereonite-3; pereonite-3 0.8× pereonite-4; pereonite-4 as long as pereonite-5; pereonite-5 1.3× pereonite-6. Pleon 0.3× BL; pleonites 1−5: of similar size – 0.3 L:W. Pleotelson 3.0× pereonite-6.

Antennule (Fig. 26D) 1.4× cephalothorax; article-1 0.6 of antennule length, 5.5 L:W, with two setae at midlength on inner margin, two setae and two PSS at midlength on outer margin, and two setae (one longer than article-2) and two PSS distally, and microtrichia along article; article-2 3.3 L:W, 0.3× article-1, with two distal setae (on inner margin); article-3 6.4 L:W, 1.5× article-2, with four short and three long terminal setae.

Antenna (Fig. 26E) article-2 1.4 L:W, with distal long seta (as long as article-3); article-3, 1.4 L:W, 0.9× article-2, with long distal seta; article-4, 7.4 L:W, 3.1× article-3 with two long setae (as long as article-5) and two PSS distally; article-5 5.9 L:W, 0.5× article-4 with long seta; article-6 1.8 L:W, with six distal setae.

Mouthparts. Labrum (Fig. 26F) typical. Left mandible (Fig. 26G) incisor with two cusps, *lacinia mobilis* well developed, with single cusp. Labium (Fig. 26H) typical, outer corner of inner lobe, with cusps, outer lobe with minute setae. Maxillule (Fig. 26I) endite typical, four innermost spines shorter than the others. Maxilla (Fig. 26J) oval.

Maxilliped (Fig. 26K) basis with seta as long as endites; endite cusps moderate; palp article-1 naked; articles 2–4 typical.

Cheliped (Figs. 26C, 27A) slender; 1.8 L:W; merus seta long; carpus 5.3 L:W, with two long setae and short seta ventrally, short dorsodistal chemosensory seta; chela longer than carpus, 3.9 L:W; palm 1.8× fixed finger; fixed finger cutting edge with one weak distal blunt cusp; dactylus slightly curved.

Pereopod-1 (Fig. 27B) overall 18.5 L:W; coxa with seta, basis 7.3 L:W, with dorsoproximal seta; merus 3.4 L:W, with long dorsodistal seta and short ventrodistal seta; carpus 3.1 L:W, as long as merus, with three distal setae (one longer than the others); propodus 5.0 L:W, 1.2× carpus, with two dorsodistal setae and ventrodistal seta; dactylus 0.6× unguis, together 0.8× propodus.

Pereopod-2 (Fig. 27C) coxa with seta; basis 7.9 L:W; with ventroproximal simple and bifurcate setae; merus 2.9 L:W, with two ventrodistal setae (one longer as half of carpus) and dorsodistal seta (longer as half of carpus); carpus 3.9 L:W, as long as merus, with dorsodistal seta and two ventrodistal setae (one broken); propodus 5.2 L:W, 0.6× merus and carpus combined, with two dorsodistal setae, ventrodistal seta, and microtrichia along article; dactylus and unguis broken.

Pereopod-3 (Fig. 27D) similar to pereopod-2 but stouter, overall, 12.5 L:W; basis 5.5 L:W, with middorsal PSS; merus 1.2 L:W, with one of two ventrodistal setae as long as carpus, and calcified microtrichia along article; carpus 1.8 L:W, 1.3× merus, with dorsodistal seta and seta and small spine ventrodistally; propodus 3.7 L:W, 1.7× carpus; dactylus 0.8× unguis, with seta (broken), together 0.5× propodus.

Pereopod-4 as pereopod-5.

Pereopod-5 (Fig. 27E) overall 10.2 L:W; basis slender, 4.0 L:W, with two ventrodistal PSS; merus 1.4 L:W, with two distal spines; carpus 2.5 L:W, 1.5× merus, with moderate prickly tubercles, dorsodistal chemosensory seta, and distal spine/crotchet; propodus 5.9 L:W, with midventral PSS seta, two ventrodistal spines, dorsodistal shorter than dactylus, and numerous microtrichia along article; dactylus 2.4× unguis, together 0.6× propodus.

Pereopod-6 (Fig. 27F) as pereopod-5 but basis naked; propodus three dorsodistal setae shorter than dactylus.

Pleopod (Fig. 27G) exopod with ten setae on outer margin; endopod with twelve.

Uropod (Fig. 27H) endopod 10 L:W, proximal article 1.6× distal article, with one distal and two distal PSS; distal article with five long terminal setae; exopod proximal article 0.5× distal article, with; 1.5× endopod proximal article, distal setation typical.

#### Distribution

Known from SW of Iceland at 1462 m depth^38^.

- ***Gudmundotanais*** **Gellert, Błażewicz & Bird n. gen.**
- LSID urn:lsid:zoobank.org:act:46B86CAD-2D2A-4831-8A11-1E58C675F5B8.

#### Diagnosis

Body stout and compact, pereonites margin rounded. Antennule article-1 short (< 4.0 L:W), inner margin with fewer than four setae. Maxilliped basis seta longer than endites, endite cusps medium. Cheliped carpus fairly slender (> 2.5 L:W), carpus with short ventral seta. Pereopod-1 merus L:W stout (< 3.0 L:W), carpus without long seta; pereopods 2–3 carpus ventrodistal seta short, microtrichia regular (if present); pereopods 4–6 carpus with prickly tubercles, unguis bifurcate, propodus distodorsal seta short. Uropod endopod biarticulate, exopod uniarticulate.

#### Type species

*Gudmundotanais gudmundssoni* n. sp. (by monotypy).

#### Etymology

Genus dedicated to Dr. Guðmundur Guðmundsson, the Curator of Marine Invertebrates in Náttúrufræðistofnun Íslands (Icelandic Institute of Natural History) in Iceland.

#### Remarks

The bifurcate unguis of pereopods 4–6 of *Gudmundotanais gudmundssoni* n. sp. is similar to those present in members of the genus *Caesatanais.* Nevertheless, *G. gudmundssoni* has a relatively slender cheliped carpus (> 2.5 L:W), that is somewhat stouter (< 2.5 L:W) in *Caesatanais*, and a stouter antennule article-1 (< 4.0 L:W), which is relatively more slender in *Caesatanais*. Besides, *G. gudmundssoni* is immediately recognised by the long and deflexed propodal seta on pereopod-1.

**Figure 28:**
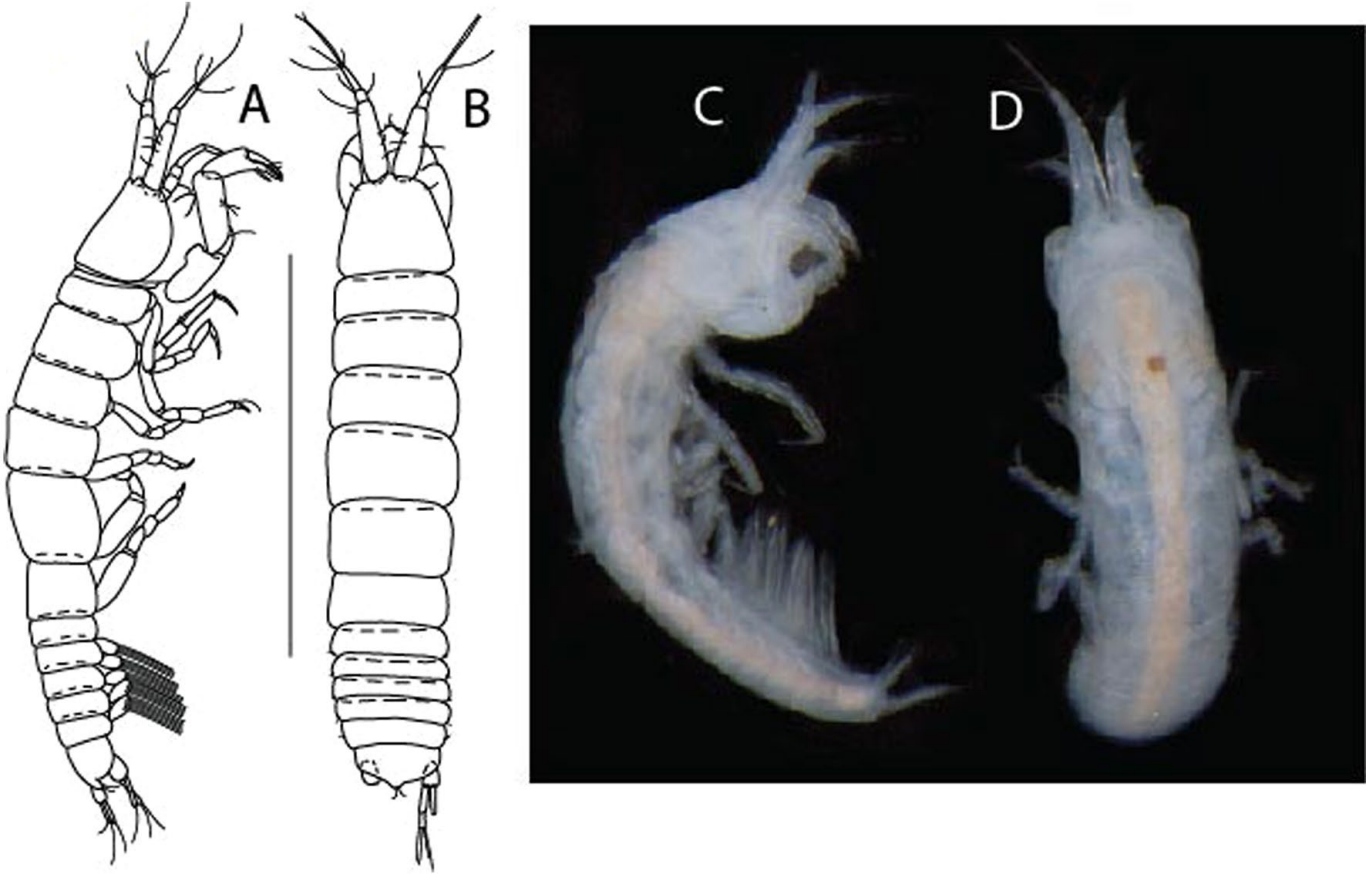
*Gudmundotanais gudmundssoni* n. sp., neuter (ICUL13944, ZMHK-64355), (**A**, **C**) Body, lateral view; (**B**, **D**) Body, dorsal view. Scale: A = 1 mm.

**Figure 29:**
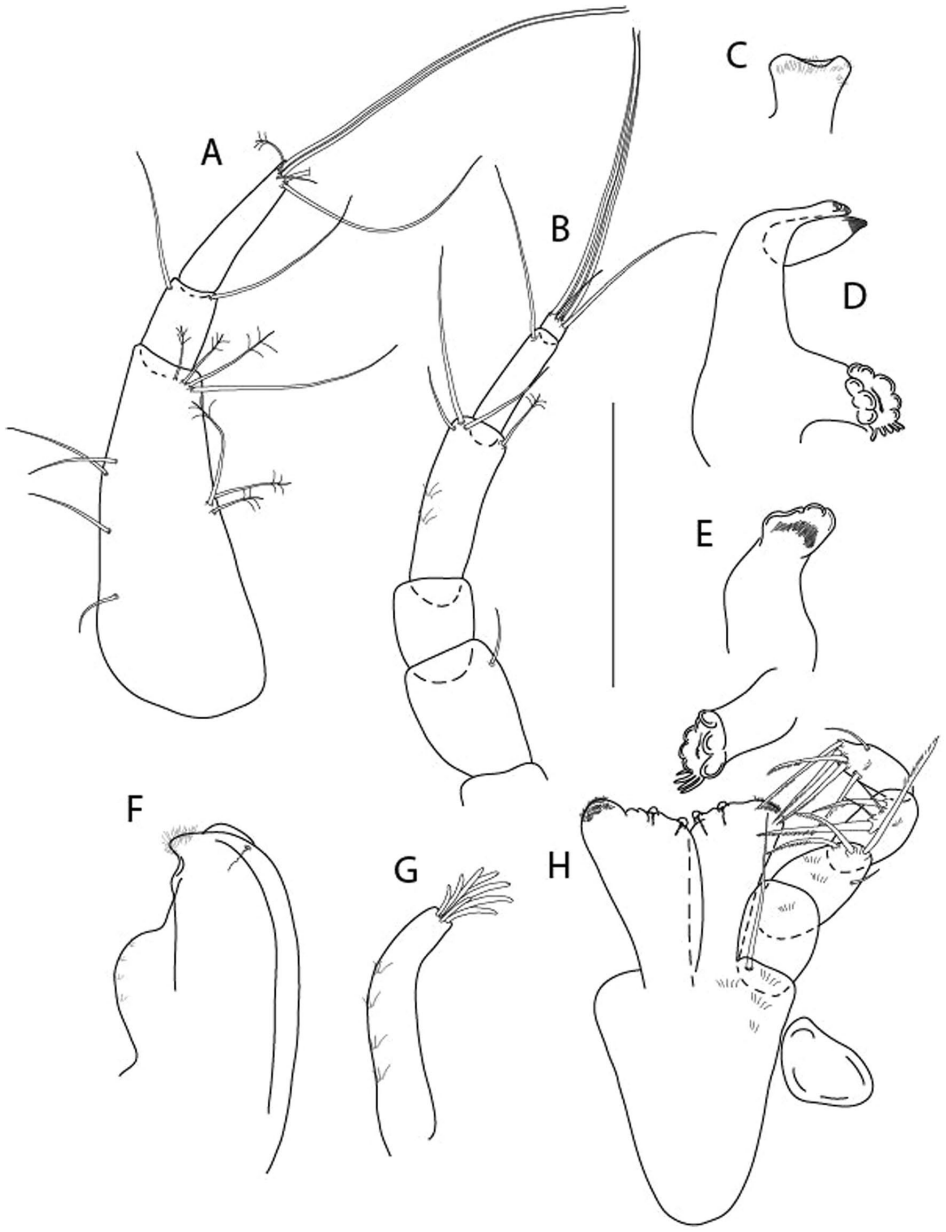
*Gudmundotanais gudmundssoni* n. sp., neuter (ICUL13945, ZMHK-64359), (**A**) Antennule; (**B**) Antenna; (**C**) Labium; (**D**) Left mandible; (**E**) Right mandible; (**F**) Labrum; (**G**) Maxillule; (**H**) Maxilliped. Scale: A–H = 0.1 mm.

**Figure 30:**
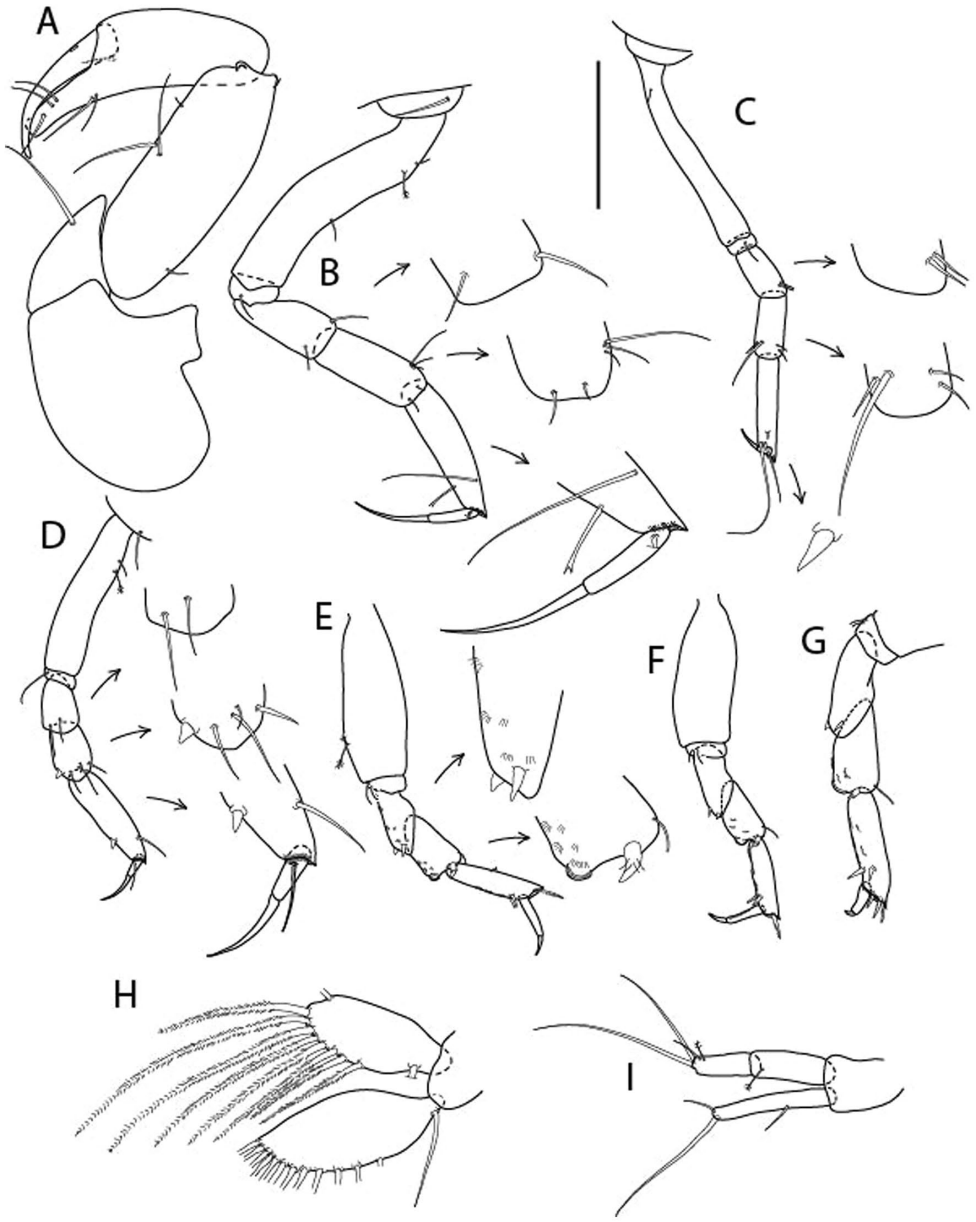
*Gudmundotanais gudmundssoni* n. sp., neuter (ICUL13945, ZMHK-64359), (**A**) Cheliped; (**B**) Pereopod-1; (**C**) Pereopod-2; (**D**) Pereopod-3; (**E**) Pereopod-4; (**F**) Pereopod-5; (**G**) Pereopod-6; (**H**) Pleopod; (**I**) Uropod. Scale: A–I = 0.1 mm.

- ***Gudmundotanais gudmundssoni*** **Gellert, Błażewicz & Bird n. sp.**
- LSID urn:lsid:zoobank.org:act:28BE6394-A103-4EAB-B40F-D8E6C2636FE6.
- (Figs. 28, 29 and 30).

#### Material examined

Holotype: neuter 1.5 mm, IceAGE, 1123-1 (ICUL13944, ZMHK-64355).

Paratypes IceAGE, St. 1123-1, neuter 1.1 mm (ICUL1119, ZMHK-64356; extraction); IceAGE, St. 1123-1, three neuters 1.1–1.4 mm (ICUL8991, ZMHK-64357); IceAGE, St. 1123-1, two neuters 1.1–1.2 mm (ICUL8992, ZMHK-64358); IceAGE, St. 1123-1, neuter 1.1 mm (ICUL13945, ZMHK-64359).

#### Other material

BIOICE, St. 2912, neuter; BIOICE, St. 3515, neuter; BIOICE, St. 3538, neuter; CHAIN 106, St. 318, 191 neuters; CHAIN 106, St. 321, two neuters; DISCOVERY, St. 7709#73, three neuters; INCAL, St. CP05, four neuters; INCAL, St. CP06, two neuters; INCAL, St. CP08, two neuters; INCAL, St. DS08, two neuters; INCAL, St. DS09, 11 neuters; INCAL, St. OS01, four neuters; INCAL, St. WS02, seven neuters; SMBA, St. ES10, 52 neuters; SMBA, St. ES12, four neuters; SMBA, St. ES129, two neuters; SMBA, St. ES135, neuter; SMBA, St. ES137, eight neuters; SMBA, St. ES143, neuter; SMBA, St. ES147, four neuters; SMBA, St. ES152, two neuters; SMBA, St. ES164, six neuters; SMBA, St. ES172, eight neuters; SMBA, St. ES180, four neuters; SMBA, St. ES185, ten neuters; SMBA, St. ES190, eight neuters; SMBA, St. ES197, four neuters; SMBA, St. ES204, two neuters; SMBA, St. ES207, five neuters; SMBA, St. ES218, three neuters; SMBA, St. ES244, neuter; SMBA, St. ES266, neuter; SMBA, St. ES283, four neuters; SMBA, St. ES285, neuter; SMBA, St. ES289, neuter; SMBA, St. ES34, six neuters; SMBA, St. ES56, neuters; SMBA, St. ES59, four neuters; SMBA, St. ES6, neuter; SMBA, St. SBC205, neuter; SMBA, St. SBC263, neuter.

#### Diagnosis

As for the genus.

#### Etymology

Species named after Dr. Guðmundur Guðmundsson, the Curator of Marine Invertebrates in Náttúrufræðistofnun Íslands (Icelandic Institute of Natural History) in Iceland.

#### Description of neuter,

Length 1.5 mm. Body (Fig. 28A−D) moderately stout, 5.0 L:W. Cephalothorax trapezoidal, 1.0 L:W, 2.1× pereonite-1, naked. Pereonites 1−6: 0.4, 0.5, 0.5, 0.7, 0.6 and 0.5 L:W, respectively; all pereonites rounded. Pereonite-1 0.6× pereonite-2; pereonite-2 as long as pereonite-3; pereonite-3 0.7× pereonite-4; pereonite-4 1.2× pereonite-5; pereonite-5 1.3× pereonite-6. Pleon 0.3× BL; pleonites 1−5 of similar size– 0.2 L:W. Pleotelson 2.1× pereonite-6.

Antennule (Fig. 29A) 1.2× cephalothorax; article-1, 0.6 of antennule length, 2.6 L:W, with four setae at midlength on inner margin, three PSS at midlength on outer margin, and long seta (longer than article-2) and three PSS distally; article-2, 1.6 L:W, 0.3× article-1, with two distal setae (one on inner and one on outer margin); article-3, 4.5 L:W, 1.8× article-2, with PSS, short and four terminal setae (one broken) and terminal spur.

Antenna (Fig. 29B) article-2, 1.3 L:W, with distal seta; article-3 1.3 L:W, 0.8× article-2, naked; article-4 3.3 L:W, 1.9× article-3, with PSS and three setae distally and microtrichia along article; article-5 4.3 L:W, 0.7× article-4 with long seta; article-6 1.3 L:W, with five distal setae.

Mouthparts. Labrum (Fig. 29C) typical. Mandible (Fig. 29D−E) molar typical. Left mandible (Fig. 29D) incisor with two cusps, *lacinia mobilis* well developed, with single cusp; right mandible (Fig. 29E) incisor with two cusps. Labium (Fig. 29F) typical, outer corner of inner lobe, with numerous microtrichia; with cusps. Maxillula (Fig. 29G) endite typical, three innermost spines short than the others. Maxilla (Fig. 29H) triangular.

Maxilliped (Fig. 29H) basis 1.7 L:W; endite cusps small; palp article-1 with microtrichia; article-2 long, setation typical, with microtrichia along article; article-3 typical, two setae shorter than the others; article-4 typical. Epignath lost in dissection.

Cheliped (Fig. 30A) slender; basis 1.5 L:W, naked; merus seta long; carpus 2.8 L:W, with two long setae (about carpus W) and short seta ventrally, dorsal margin setation typical; chela longer than carpus, 3.5 L:W; fixed finger (broken) cutting edge with distal blunt cusps (broken); dactylus slightly curved, with seta (broken).

Pereopod-1 (Fig. 30B) overall 13.9 L:W; coxa with seta, basis 4.9 L:W, with dorsoproximal PSS and seta and middorsal seta; merus 2.1 L:W, with single dorsodistal and ventrodistal setae; carpus 2.5 L:W, 1.1× merus, with five distal setae (two shorter than the others); propodus 3.7 L:W, 1.3× carpus, with deflexed dorsodistal seta and ventrodistal bifurcate seta; dactylus 0.7× unguis, with seta (broken), together as long as propodus.

Pereopod-2 (Fig. 30C) overall 19.5 L:W; coxa with seta; basis 8.6 L:W, with ventroproximal seta; merus 2.1 L:W, with two ventrodistal setae; carpus 2.5 L:W, 1.1× merus, with two dorsodistal setae (one longer than the other) and two ventrodistal setae; propodus 5.1 L:W, 0.7× merus and carpus combined, with dorsodistal seta, and long dorsodistal seta and ventrodistal spine; dactylus 0.7× unguis, with seta (broken), together 0.4× propodus.

Pereopod-3 (Fig. 30D) similar to pereopod-2 but stouter, overall, 12.1 L:W; basis 4.5 L:W, with single dorsoproximal seta and PSS; merus 1.9 L:W; carpus 1.7 L:W, the same length as long as merus, with four setae and spine distally; propodus 4.0 L:W, 1.8× carpus, with dorsodistal seta; dactylus 0.6× unguis, together 0.6× propodus.

Pereopod-4 (Fig. 30E) overall 7.5 L:W; basis robust, 2.2 L:W, with two midventral PSS; merus 2.2 L:W, with two distal spines, and microtrichia along article; carpus 1.9 L:W, 0.8× merus, with prickly tubercles, dorsodistal chemosensory seta, and distal spine/crotchet; propodus 4.6 L:W, with middorsal seta (broken), two ventrodistal spines, and dorsodistal serrate seta about half as long as dactylus; dactylus 1.8× unguis, together 0.7× propodus.

Pereopod-5 (Fig. 30F) similar to pereopod-4 but slightly stouter, overall, 6.2 L:W; basis robust, 2.4 L:W, naked; merus 2.5 L:W; carpus 1.8 L:W, 0.9× merus; propodus 4.2 L:W, without middorsal seta; dactylus 2.3× unguis, together 0.7× propodus.

Pereopod-6 (Fig. 30G) as pereopod-5; propodus three dorsodistal setae shorter than dactylus.

Pleopod (Fig. 30H) exopod with ten plumose setae on outer margin; endopod with thirteen.

Uropod (Fig. 30I) endopod proximal article 1.4× distal article, with a distal PSS; distal article with a PSS and three long terminal setae; exopod 0.9× endopod proximal article, with seta at midlength, the other setation typical.

#### Distribution

Known from seven locations off Iceland (Denmark Strait, Iceland Basin), the N Rockall Trough, Porcupine Seabright, Rockall Trough, South Biscay, and South Feni Ridge) (Fig. 5), at depths 716.5–2946 m (this study).

- ***Hansenotanais*** **Gellert, Błażewicz & Bird n. gen.**
- LSID urn:lsid:zoobank.org:act:E59A8A66-E71A-467B-9B41-89997C770695.

#### Diagnosis

Body stout and robust, pereonite margins rounded. Antennule article-1 long and slender (> 4.0 L:W), inner margin with three setae. Maxilliped basis seta longer than endites, endite cusps moderate. Cheliped carpus stout (< 2.5 L:W), carpus with short ventral seta. Pereopod-1 merus L:W relatively stout (< 3.0 L:W), carpus without long seta; pereopods 2–3 carpus ventrodistal seta long, microtrichia regular (if present); pereopods 4–6 carpus with low cusps, propodus distodorsal seta short, unguis bifurcate. Uropod endopod biarticulate, exopod uniarticulate.

#### Type genus

*Hansenotanais partykae* n. sp. (by designation).

#### Etymology

The name of the genus is in honour of Hans Jacob Hansen—Danish zoologist and pioneer in research of deep water Typhlotanaidae.

#### Species included

*Hansenotanais inermis* (Hansen, 1913) n. comb.; *Hansenotanais hansjacobi* n. sp.; *Hansenotanais partykae* n. sp.

#### Remarks

The genus *Hansenotanais* n. gen. is characterized by the rounded pereonite margins, presence of a long seta on the pereopods 2–3 carpus, cusps (not prickly tubercles) on pereopods 4–6 carpus, and biarticulate uropodal endopod and unarticulate exopod. The lack of prickly tubercles and presence of carpal cusp clearly distinguishes *Hansenotanai*s from most of the ‘stout-bodied’ typhlotanaids. So far only *Obesutanais* lacks prickly tubercles in the pereopods 4–6 carpus, although both genera can be distinguished by the long dorsodistal seta on pereopods 4–6 in *Obesutanais* and short dorsodistal seta on pereopods 4–6 in *Hansenotanais*. It is worthy to noting that similar carpal cusps are present in the genus *Typhlamia*, which belongs to the ‘slender-bodied’ Typhlotanaidae. Considering the species present in the N Atlantic, only *Hansenotanais* and *Caesatanais* have long setae on pereopods 2–3 although *Hansenotanais* has rounded pereonite margins (straight in *Caesatanais*) and biarticulate uropod rami (uniarticulate in *Caesatanais*).

- ***Hansenotanais inermis*** **(Hansen, 1913) n. comb.**
- *Typhlotanais inermis* Hansen, 1913^38^: 46–47, pl. IV 6a–6 g.

#### Material examined

AFEN 1996, St. 53803#1, neuter; AFEN 1996, St. 53878#2, neuter; AFEN 1996, St. 53896#(1)/2, neuter; AFEN 1996, St. 53899#1, neuter; AFEN 1996, St. 53915#1, neuter; BIOFAR, St. 15, 10 neuters; BIOFAR, St. 168, two neuters; BIOFAR, St. 169, four neuters; BIOFAR, St. 170, 10 neuters; BIOFAR, St. 171, two neuters; BIOFAR, St. 477, neuter; BIOFAR, St. 482, neuter; BIOFAR, St. 274, two neuters; BIOFAR, St. 275, neuter; BIOFAR, St. 458, 12 neuters; BIOFAR, St. 424, two neuters; BIOFAR, St. 167, three neuters; BIOFAR, St. 167, three neuters; BIOFAR, St. 9009, two neuters; BIOICE, St. 2570, seven neuters; BIOICE, St. 2579, two neuters; BIOICE, St. 2591, four neuters; BIOICE, St. 2741, 29 neuters; BIOICE, St. 2744, neuter; BIOICE, St. 2023, neuter; BIOICE, St. 2088, neuter; BIOICE, St. 2089, four neuters; BIOICE, St. 2091, 18 neuters; BIOICE, St. 2619, two neuters; BIOICE, St. 2629, two neuters; BIOICE, St. 2644, neuter; BIOICE, St. 2648, neuter; BIOICE, St. 2660, neuter; BIOICE, St. 2779, neuter; BIOICE, St. 2003, two neuters; BIOICE, St. 2011, neuter; BIOICE, St. 2014, neuter; BIOICE, St. 2315, neuter; BIOICE, St. 2317, three neuters; BIOICE, St. 2318, neuter; BIOICE, St. 2323, three neuters; BIOICE, St. 2363, neuter; BIOICE, St. 2364, five neuters; BIOICE, St. 3247, two neuters; BIOICE, St. 2118, two neuters; BIOICE, St. 2124, two neuters; BIOICE, St. 2136, neuter; BIOICE, St. 2786, four neuters; BIOICE, St. 3115, neuter; BIOICE, St. 3124, 55 neuters; DTI 2000, St. 55273#1, neuter; DTI 2000, St. 55300#1, preparatory male; DTI 2000, St. 55404#1,2, neuter; NORBI, St. DS17, neuter; SMBA, St. ES87, neuter.

#### Diagnosis

Antennule article-3 with aesthetasc. Antenna article-2 with two short setae. Pereopods 2–3 carpus ventrodistal seta short. Uropod exopod 0.6 L:W of endopod.

#### Distribution

**Figure 31:**
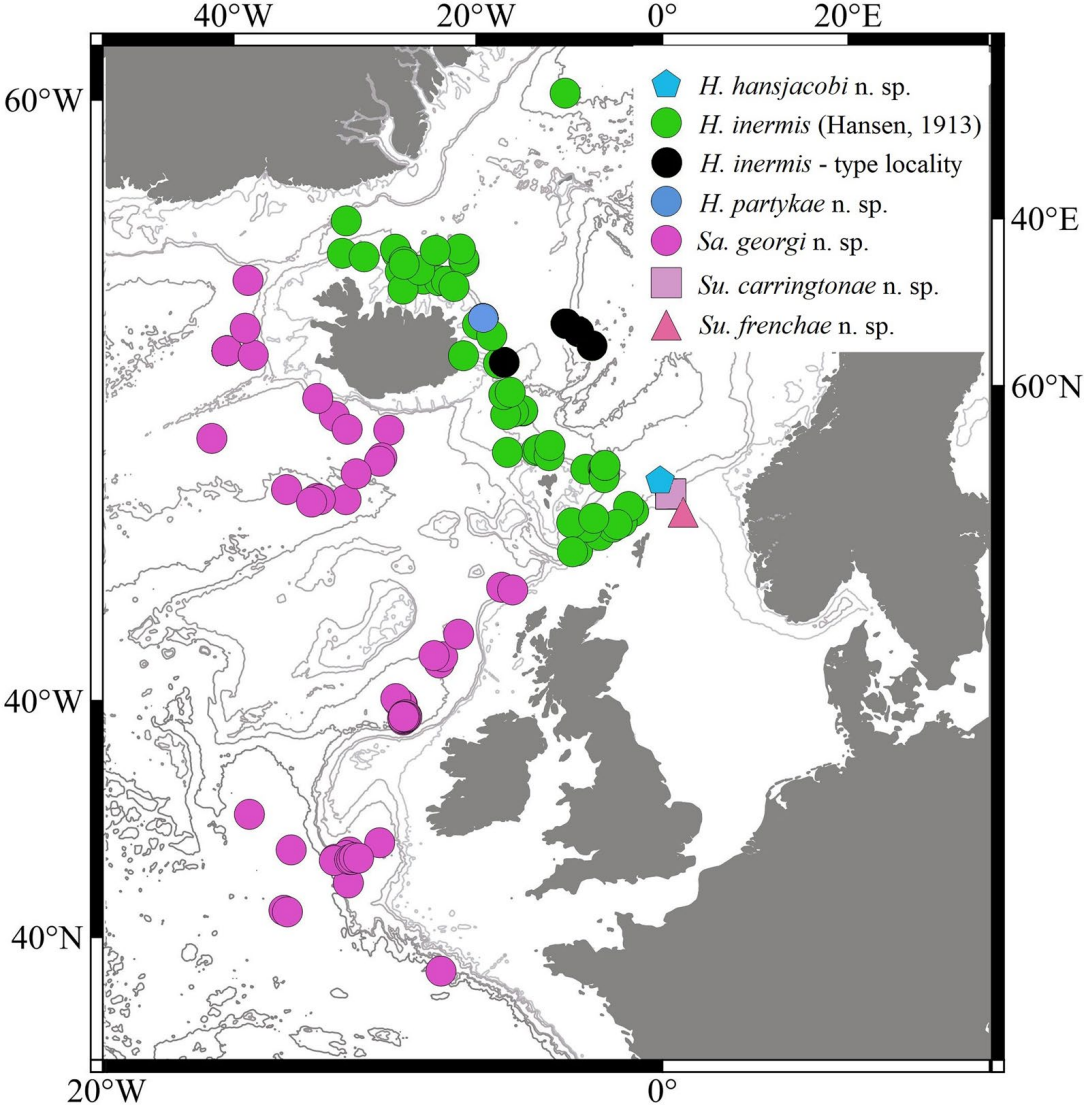
The depth distribution of *H. hansjacobi* n. sp., *H. inermis*, *H. partykae* n. sp., *Sa. georgi* n. sp., *Su. carringtonae* n. sp. and *Su. frenchae* n. sp (QGIS 3.28 software https://www.qgis.org/pl/site/).

Known from N of the Faroes, at depths of 862–1285 m (Fig. 31) (this study), and E of Iceland at depths of 1372.5 m^38^

#### Remarks

From all members of *Hansenotanais inermis* n. sp., the nominal species can be distinguished by the presence of a short ventrodistal seta on the carpus of pereopods 2–3

**Figure 32:**
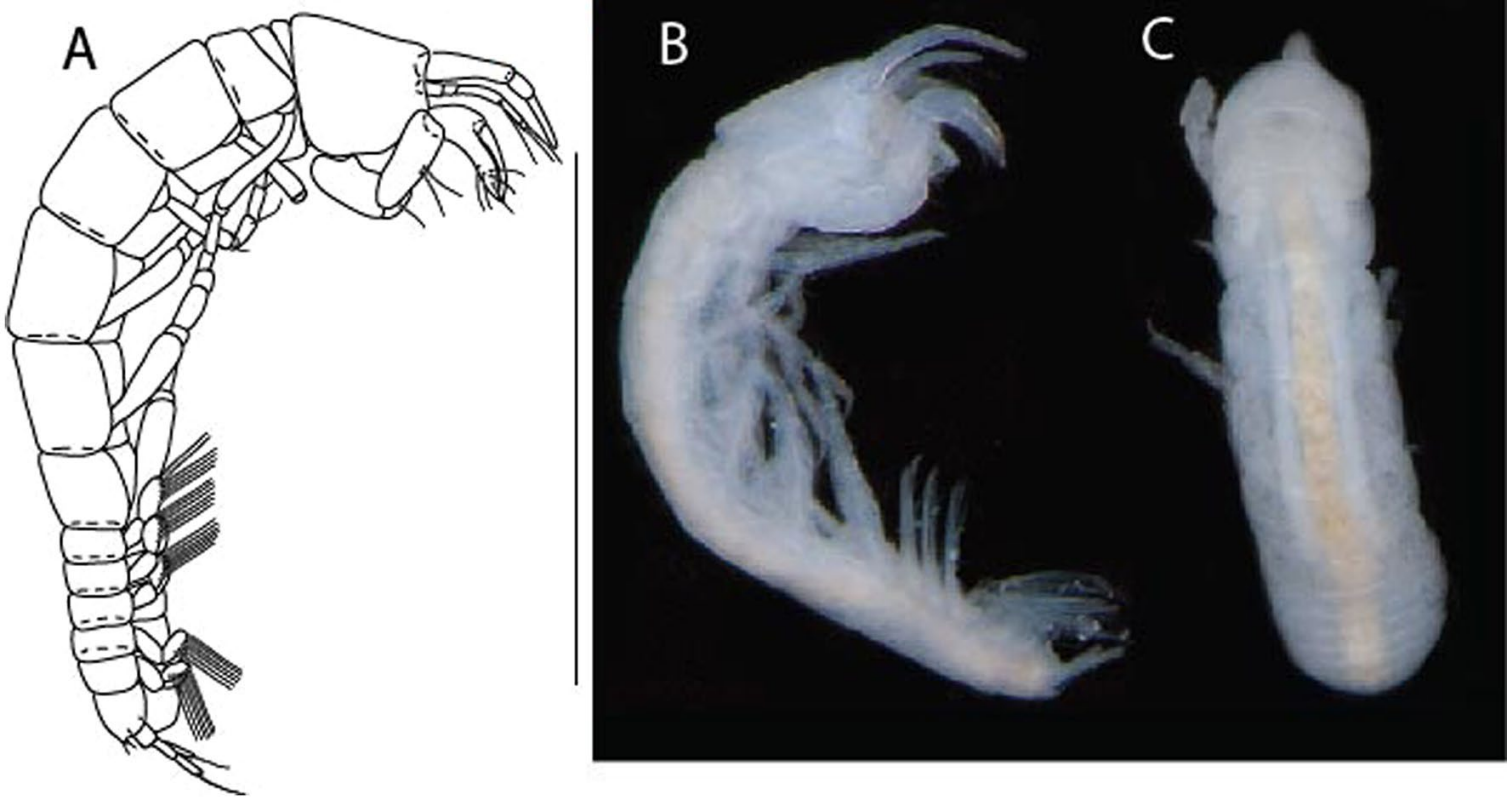
*Hansenotanais hansjacobi* n. sp., neuter (ICUL13953, ZMHK-64360), (**A**, **B**) Body, lateral view; (**C**) Body, dorsal view. Scale: A = 1 mm.

**Figure 33:**
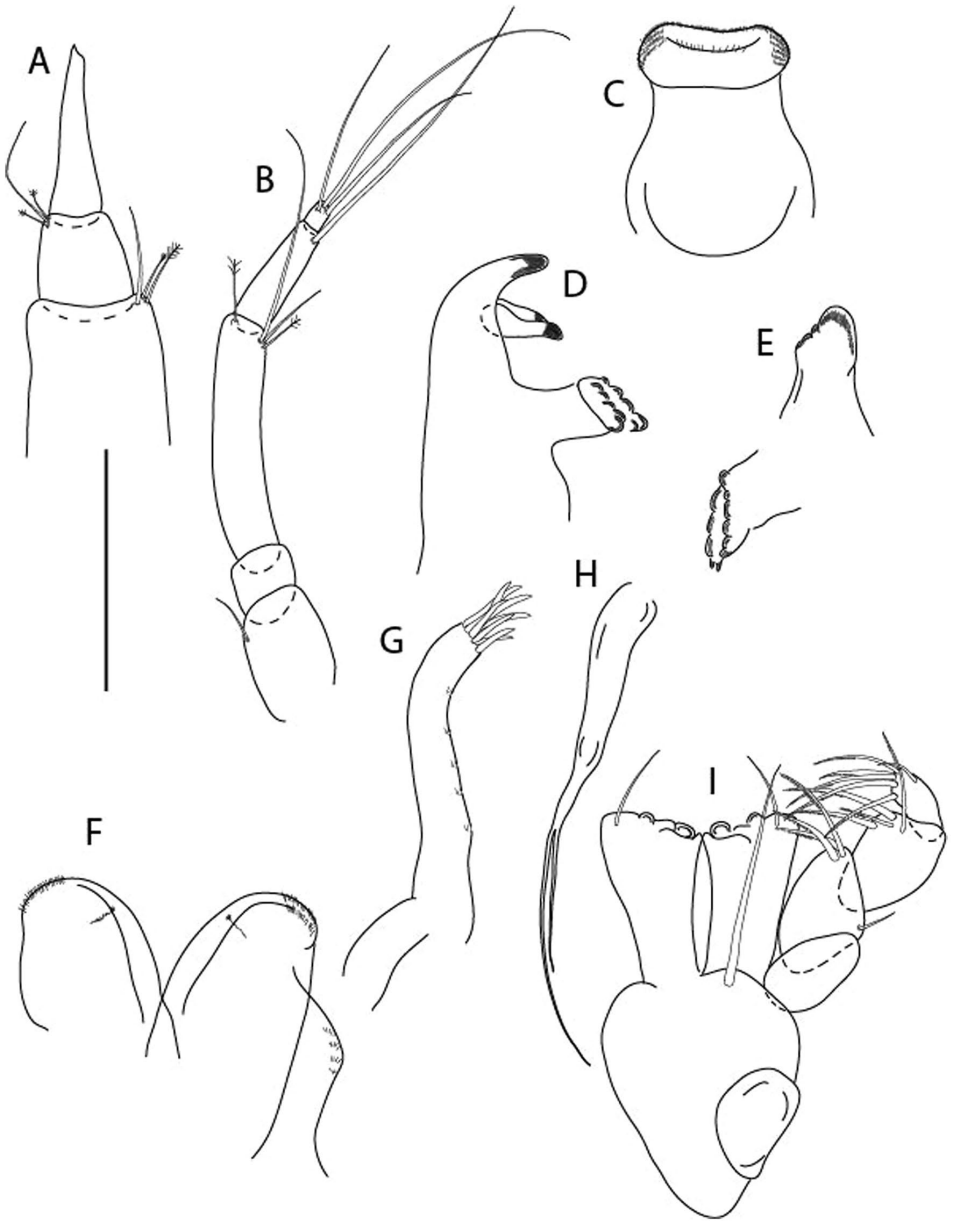
*Hansenotanais hansjacobi* n. sp., neuter (ICUL8982, ZMHK-64361), (**A**) Antennule; (**B**) Antenna; (**C**) Labium; (**D**) Left mandible; (**E**) Right mandible; (**F**) Labrum; (**G**) Maxillule; (**H**) Palp; (**I**) Maxilla; (**I**) Maxilliped. Scale: A–I = 0.1 mm.

**Figure 34:**
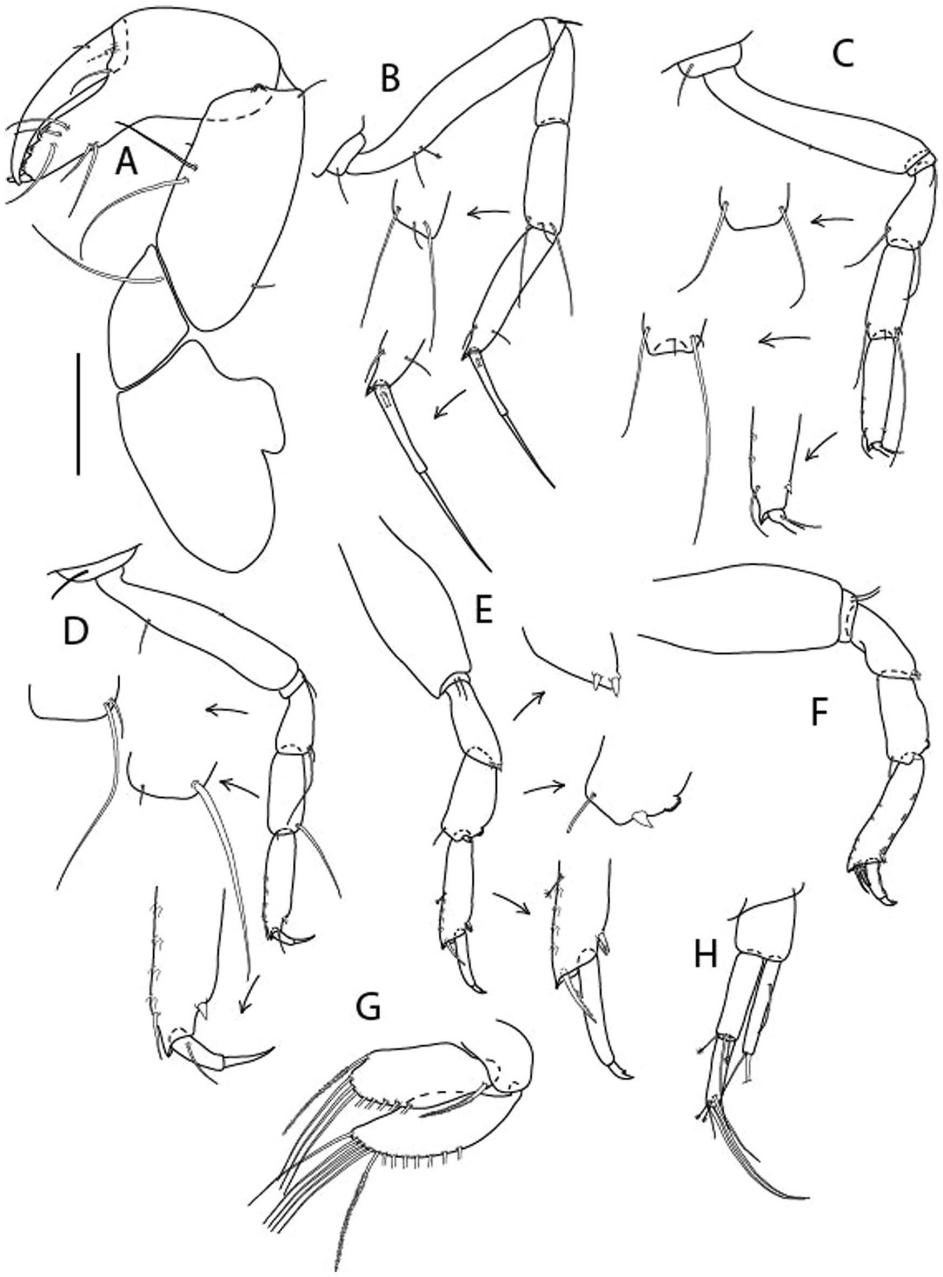
*Hansenotanais hansjacobi* n. sp., neuter (ICUL8982, , ZMHK-64361), (**A**) Cheliped; (**B**) Pereopod-1; (**C**) Pereopod-2; (**D**) Pereopod-3; (**E**) Pereopod-4; (**F**) Pereopod-6; (**G**) Pleopod; (**H**) Uropod. Scale: A–H = 0.1 mm.

- ***Hansenotanais hansjacobi*** **Gellert & Błażewicz n. sp.**
- LSID urn:lsid:zoobank.org:act:F2FB1597-170E-4173-BE69-34DFDF4E4285.
- (Figs. 32, 33 and 34).

#### Material examined

Holotype, neuter 1.6 mm, IceAGE II, St. 868-3 (ICUL13953, ZMHK-64360). Paratypes IceAGE II, St. 868-3, neuter (ICUL8982, ZMHK-64361; broken, dissected).

#### Diagnosis

Antennule article-3 without aesthetasc. Antenna article-2 with long seta. Pereopod-1 merus 3.0 L:W, carpus with three setae; pereopod-2 merus 2.4 L:W, with long seta, carpus 2.7 L:W; pereopod-3 carpus 2.8 L:W; pereopods 2–3 carpus ventrodistal seta long. Uropod exopod 0.6 L:W of endopod.

#### Etymology

The name is a combination of two names of Hans Jacob Hansen.

#### Description of neuter,

Length 1.6 mm. Body (Fig. 32A−C) moderately stout, 5.0 L:W. Cephalothorax trapezoidal, 1.3 L:W, 2.2× pereonite-1, naked. Pereonites 1−6: 0.3, 0.6, 0.6, 0.6, 0.7 and 0.5 L:W, respectively; all pereonites rounded. Pereonite-1 0.4× pereonite-2; pereonite-2 as long as pereonite-3; pereonite-3 as long as pereonite-4; pereonite-4 as long as pereonite-5; pereonite-5 1.4× pereonite-6. Pleon 0.2× BL; pleonites 1−5: of similar size – 0.3 L:W. Pleotelson 2.8× pereonite-6.

Antennule (Fig. 33A) 1.2× cephalothorax; article-1, broken, with seta and two PSS distally; article-2 1.1 L:W, 0.3× article-1, with long seta and two PSS distally; article-3 5.2 L:W, 1.8× article-2, setae missing.

Antenna (Fig. 33B) article-2 1.4 L:W, with distal seta; article-31.2 L:W, 0.6× article-2, naked; article-4 5.4 L:W, 3.3× article-3, with two PSS and two setae (one longer than article-5) distally; article-5 3.2 L:W, 0.5× article-4, with long seta; article-6 1.8 L:W, with three distal setae.

Mouthparts. Labrum (Fig. 33C) typical. Mandible (Fig. 33D−E) molar typical. Left mandible (Fig. 33D) incisor with single cusp, *lacinia mobilis* well developed, with single cusp; right mandible (Fig. 33E) incisor with two cusps. Labium (Fig. 33F) typical, outer corner of inner lobe and outer lobe with short setae; with cusps. Maxillule (Fig. 33G) endite typical, three innermost spines shorter than the others, microtrichia along endite; palp (Fig. 33H) typical. Maxilla (Fig. 33I) rounded.

Maxilliped (Fig. 33I) basis with seta reaching beyond endites; endite cusps large; palp article-1 naked; articles 2–4 typical. Epignath lost during dissection.

Cheliped (Fig. 34A) slender; basis 1.7 L:W, naked; merus seta long; carpus 2.3 L:W, with two long setae (one longer than carpus W) and short seta ventrally, dorsal margin setation typical; chela longer than carpus, 1.6 L:W, with four setae (three in inner and one in outer margin); palm 1.1× fixed finger; fixed finger with cutting edge with three big, distal blunt cusps; dactylus slightly curved, with proximal inner seta.

Pereopod-1 (Fig. 34B) overall 17.5 L:W; coxa with seta, basis 5.8 L:W, with dorsoproximal PSS and seta; merus 3.0 L:W naked; carpus 2.8 L:W, 1.2× merus, with three distal setae (two as long as half of propodus); propodus 5.0 L:W, 1.3× carpus, with dorsodistal and ventrodistal setae; dactylus 0.8× unguis, with seta (broken), together 1.1× propodus.

Pereopod-2 (Fig. 34C) coxa with seta; basis 5.9 L:W, with middorsal (broken) seta; merus 2.4 L:W, with ventrodistal and dorsodistal setae; carpus 2.8 L:W, 1.2× merus, with two short and two long setae (one longer than propodus); propodus 4.1 L:W, 0.6× merus and carpus combined, dorsodistal seta and ventrodistal spine, and microtrichia along article; dactylus and unguis broken, dactylus with seta.

Pereopod-3 (Fig. 34D) similar to pereopod-2 but stouter, overall, 12.1 L:W; basis 5.1 L:W, with middorsal and midventral (broken) setae; merus 2.4 L:W, with two ventrodistal setae (one as long as carpus); carpus 2.4 L:W, 1.4× merus, with short and long setae (almost as long as propodus) distally; propodus 3.6 L:W, 1.3× carpus; dactylus 1.4× unguis, with seta, together 0.5× propodus.

Pereopod-4 (Fig. 34E) overall 6.8 L:W; basis robust, 2.5 L:W, naked; merus 2.0 L:W, with two distal spines; carpus 2.0 L:W, 1.2× merus, with low cusp, dorsodistal chemosensory seta, and distal spine/crotchet; propodus 4.0 L:W, 1.2× carpus, with middorsal PSS, two ventrodistal serrate spines, dorsodistal serrate seta shorter than dactylus, and microtrichia along article; dactylus 3.4× unguis, together 0.7× propodus.

Pereopod-5 as pereopod-4.

Pereopod-6 (Fig. 34F) as pereopod-4; propodus three dorsodistal setae shorter than dactylus.

Pleopod (Fig. 34G) exopod with ten plumose setae on outer margin; endopod with thirteen.

Uropod (Fig. 34H) endopod about 6.5 L:W, proximal article 1.2× distal article, with distal seta and two PSS; distal article with two PSS and four long terminal setae; exopod 1.2× endopod proximal article, with seta at midlength, the other setation typical.

#### Distribution

 Known from one location in the Faroe Channel at depths of 587–614 m (Fig. 31) (this study).

#### Remarks

A long ventrodistal long seta on the pereopods 2–3 carpus separates *Hansenotanais hansjacobi* n. sp. from *H. inermis*, which has a short seta. Furthermore, the long seta on antenna article-2 and three setae on the pereopod-1 carpus distinguishes *H. hansjacobi* from *Hansenotanais partykae* (see below) with its short seta on article-2 and only a seta on the pereopod-1 carpus. Also, the *H. hansjacobi* has a long pereopod-1 merus (3.0 L:W), which is stouter in *H. partykae* (2.1 L:W).

**Figure 35:**
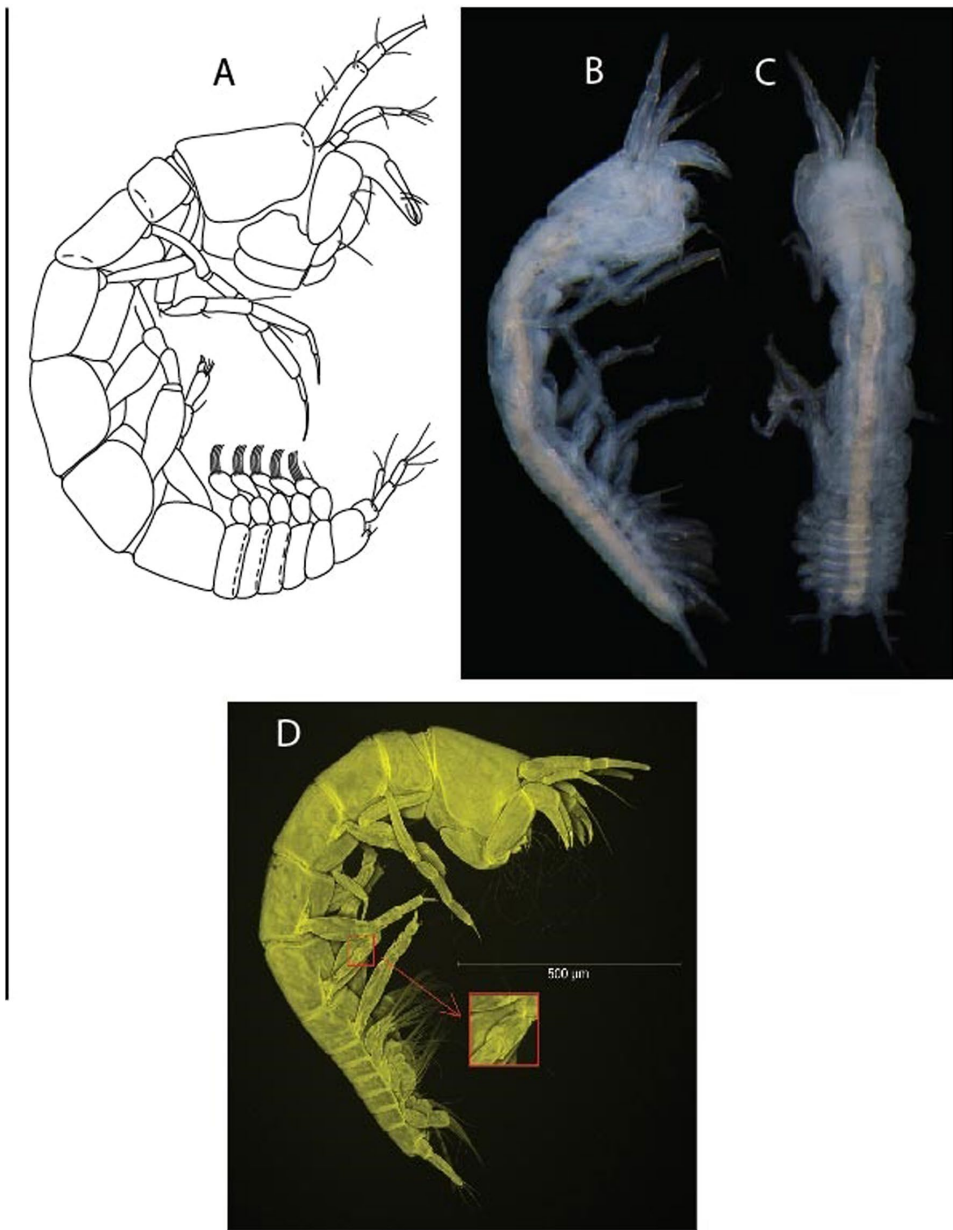
*Hansenotanais partykae* n. sp., neuter (ICUL13957, ZMHK-64362), (**A**, **B**) Body, lateral view; (**C**) Body, dorsal view; (**D**) CLSM images: Body, lateral view, pereopods 4–6 carpus with two cusps. Scale: A = 1 mm, D = 0.5 mm.

**Figure 36:**
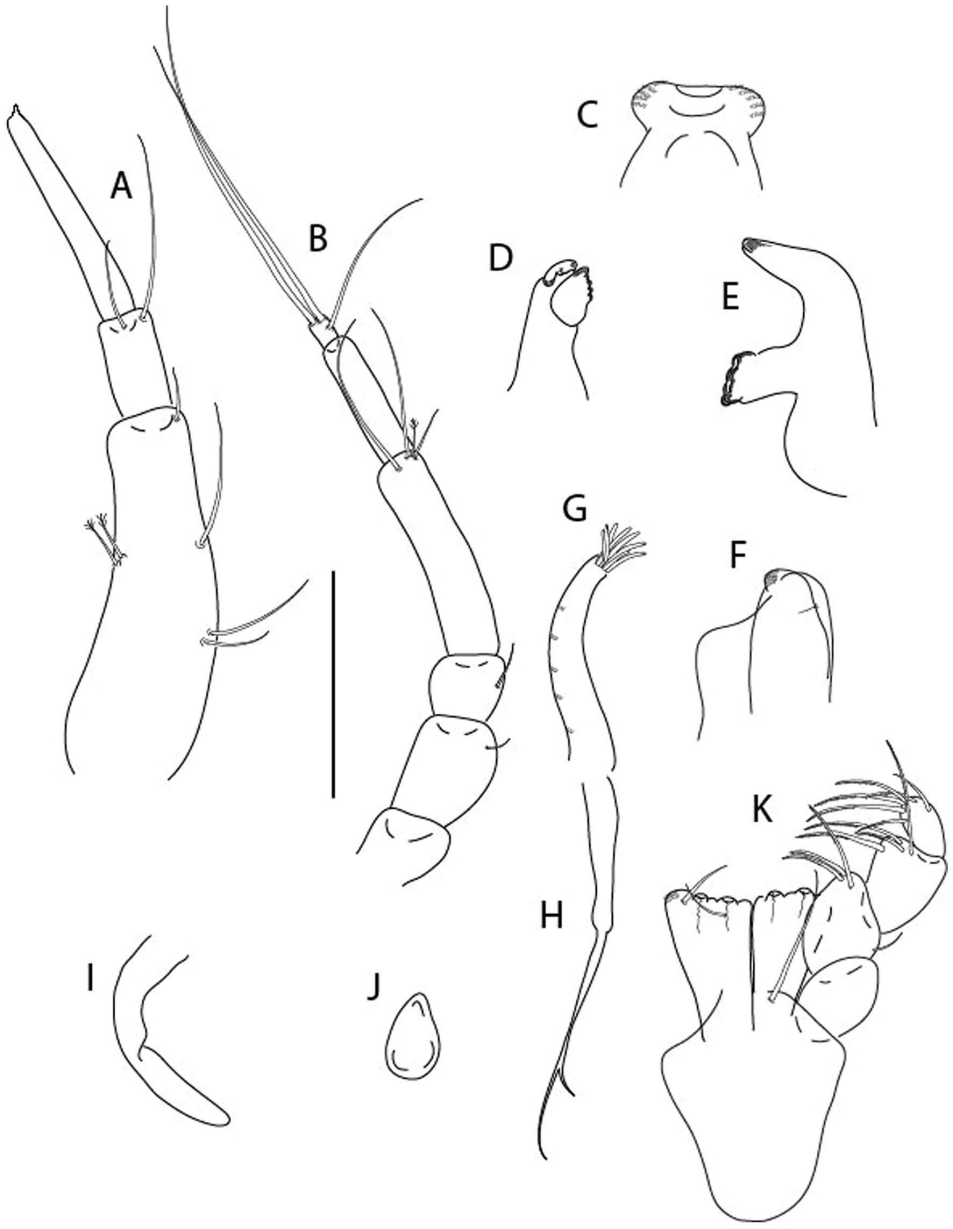
*Hansenotanais partykae* n. sp., neuter (ICUL13961, ZMHK-64365), (**A**) Antennule; (**B**) Antenna; (**C**) Labrum; (**D**) Left mandible; (**E**) Right mandible; (**F**) Labium; (**G**) Maxillule; (**H**) Palp; (**I**) Epignath; (**J**) Maxilla; (**K**) Maxilliped. Scale: A–K = 0.1 mm.

**Figure 37:**
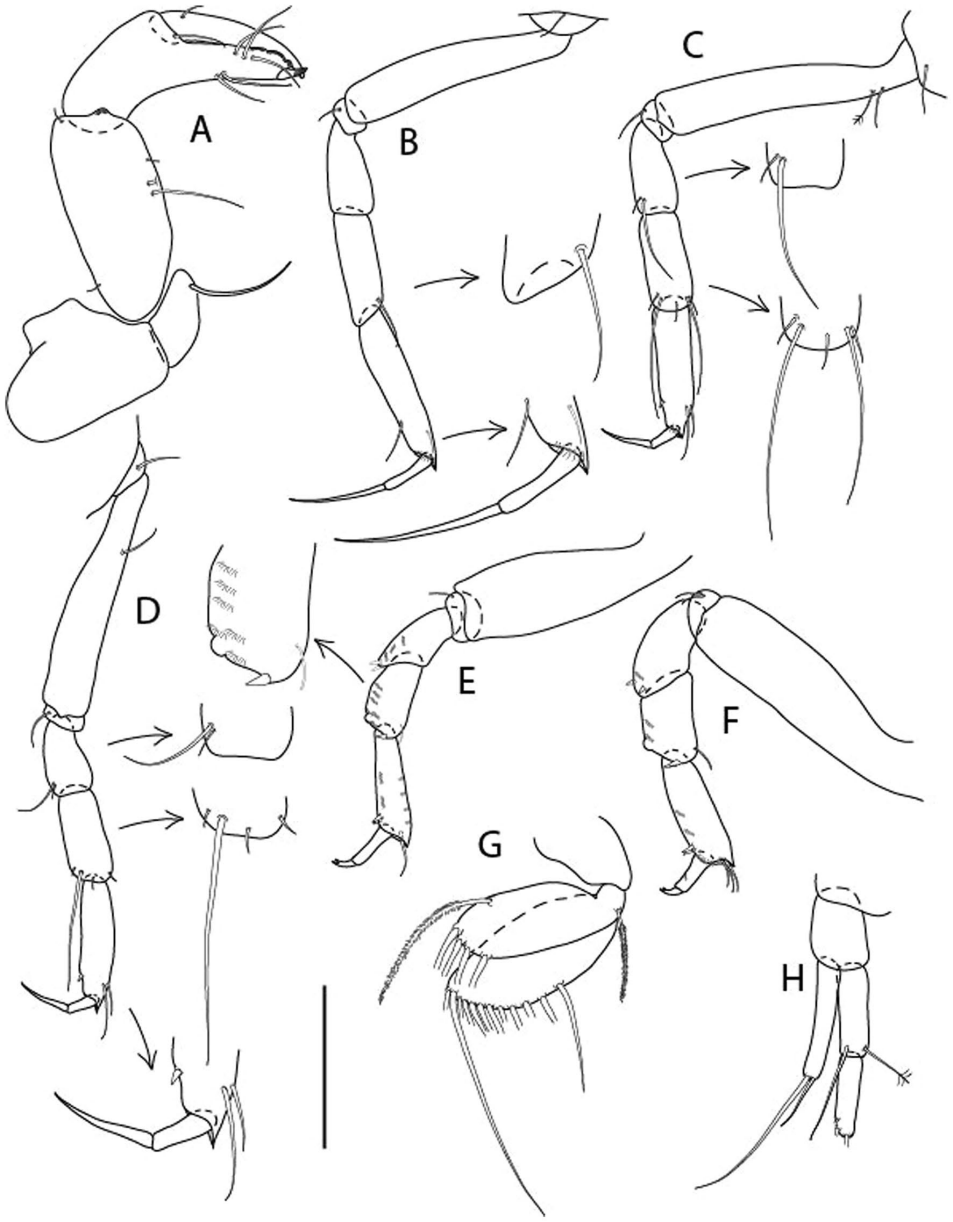
*Hansenotanais partykae* n. sp., neuter (ICUL13961, ZMHK-64365), (**A**) Cheliped; (**B**) Pereopod-1; (**C**) Pereopod-2; (**D**) Pereopod-3; (**E**) Pereopod-4; (**F**) Pereopod-6; (**G**) Pleopod; (**H**) Uropod. Scale: A–H = 0.1 mm.

- ***Hansenotanais partykae*** **Gellert & Błażewicz n. sp.**
- LSID urn:lsid:zoobank.org:act:49A60433-70 EB-44B4-9197-C01C348DB6C3.
- (Figs. 35, 36 and 37).

#### Material examined

Holotype, neuter 1 mm, IceAGE, St. 1209-1 (ICUL13957, ZMHK-64362).

Paratypes IceAGE, St. 1209-1, neuter (ICUL9630, broken, extraction*); IceAGE, St. 1209-1, neuter 1 mm (ICUL9629, ZMHK-64363; extraction); IceAGE, St. 1209-1, neuter 1.3 mm (ICUL13960, ZMHK-64364); IceAGE, St. 1209-1, neuter 1.2 mm (ICUL13961, ZMHK-64365; dissected); IceAGE, St. 1209-1, neuter 1.1 mm (ICUL12666, ZMHK-64366); IceAGE, St. 1209-1, neuter 1.1 mm (ICUL2180, ZMHK-64367); IceAGE, St. 1209-1, neuter 1.2 mm (ICUL9817, ZMHK-64368).*Individual not recovered after DNA extraction.

#### Diagnosis

Antennule article-3 without aesthetasc. Antenna article-2 with short seta. Pereopod-1 merus 2.1 L:W, carpus 2.2 L:W, with seta; pereopod-2 merus 2.1 L:W, with two (long and short) setae; pereopod-3 carpus 2.5 L:W; pereopods 2–3 carpus ventrodistal seta long. Uropod exopod 0.7 L:W of endopod.

#### Etymology

This species is dedicated to Natalia Partyka, Polish table tennis player, four-time Paralympic champion.

#### Description of neuter,

Length 0.8 mm. Body (Fig. 35A−D) stout, 4.6 L:W. Cephalothorax trapezoidal, 0.9 L:W, 2.4× pereonite-1, naked. Pereonites 1−6: 0.5, 0.9, 0.9, 0.7, 0.5 and 0.5 L:W, respectively; all pereonites rounded. Pereonite-1 0.5× pereonite-2; pereonite-2 as long as pereonite-3; pereonite-3 as long as pereonite-4; pereonite-4 1.2× pereonite-5; pereonite-5 1.2× pereonite-6. Pleon 0.2× BL; pleonites 1−5: of similar size – 0.2 L:W. Pleotelson 2.1× pereonite-6.

Antennule (Fig. 36A) 1.4× cephalothorax; article-1, 0.6 of antennule length, 3.6 L:W, with three setae on inner margin and two PSS on outer margin, and distal seta; article-2, 2.2 L:W, 0.3× article-1, with two distal setae on inner margin; article-3, 8.5 L:W, 2.0× article-2, naked (setae broken).

Antenna (Fig. 36B) article-2 1.5 L:W, with distal seta; article-3 1.1 L:W, 0.6× article-2, with seta; article-4, 4.5 L:W, 2.7× article-3 with PSS and three setae distally (two as long as article-5); article-5 3.9 L:W, 0.6× article-4, with long seta; article-6 1.7 L:W, with three distal setae.

Mouthparts. Labrum (Fig. 36C) typical. Mandible (Fig. 36D−E) molar typical. Left mandible (Fig. 36D) incisor with single cusp, *lacinia mobilis* well developed, with single cusp; right mandible (Fig. 36E) incisor with two cusps. Labium (Fig. 36G) typical, outer corner of inner lobe; with cusps. Maxillule (Fig. 36H) typical, microtrichia along endite; palp (Fig. 36I) shorter than endite. Maxilla (Fig. 36K) triangular.

Maxilliped (Fig. 36K) basis 1.6 L:W, with seta reaching beyond endites; endite cusps moderate; palp article-1 naked; articles 2–4 typical. Epignath (Fig. 36I) as long as maxillule endite, rounded distally.

Cheliped (Fig. 37A) slender; basis 1.9 L:W, naked; merus seta long; carpus 2.2 L:W, with two long (one broken) setae (as long as carpus W) and short seta ventrally, dorsal margin setation typical; chela longer than carpus, 2.0 L:W, with seta; fixed finger cutting edge with three very weak, distal blunt cusps; dactylus slightly curved, with seta.

Pereopod-1 (Fig. 37B) overall 16.3 L:W; coxa with seta, basis 5.0 L:W, naked; merus 2.5 L:W, naked; carpus 3.0 L:W, 1.2× merus, with dorsodistal seta; propodus 3.7 L:W, 1.3× carpus, with single dorsodistal and ventrodistal setae; dactylus 0.6× unguis; dactylus and unguis together 0.8× propodus.

Pereopod-2 (Fig. 37C) overall 14.4 L:W; coxa with seta; basis 5.8 L:W, with dorsoproximal seta and PSS; merus 2.2 L:W, with two ventrodistal setae (one longer than half of propodus); carpus 2.5 L:W, 1.2× merus, with five setae (two longer than half of propodus); propodus 5.1 L:W, 0.7× merus and carpus combined, with two dorsodistal setae and ventrodistal spine; dactylus 0.6× unguis, together 0.6× propodus.

Pereopod-3 (Fig. 37D) similar to pereopod-2 but stouter, overall, 13.1 L:W; basis 5.4 L:W, with dorsoproximal seta; merus 2.1 L:W; carpus 2.4 L:W, 0.9× merus, with long seta (as long as propodus) and three short setae distally; propodus 4.2 L:W, 1.4× carpus; dactylus 0.6× unguis; together 0.6× propodus.

Pereopod-4 (Fig. 37E) overall 6.9 L:W; basis robust, 3.0 L:W, naked; merus 2.4 L:W, with two distal spines, and microtrichia along article; carpus 1.8 L:W, 1.5× merus, with two cusp, dorsodistal chemosensory seta, distal spine/crotchet; propodus 3.7 L:W, with two ventrodistal spines, and dorsodistal seta shorter than dactylus; dactylus 2.1× unguis, together 0.6× propodus.

Pereopod-5 as pereopod-4.

Pereopod-6 (Fig. 37F) as pereopod-4; propodus three dorsodistal setae shorter than dactylus.

Pleopod (Fig. 37G) with six plumose setae on outer margin; endopod with thirteen.

Uropod (Fig. 37H) endopod 6.0 L:W, proximal article as long as distal article, with distal seta and PSS; distal article with three setae (broken) and three long terminal setae; exopod 0.9× endopod proximal article, with seta at midlength, the other setation typical.

#### Distribution

Known from one location off Iceland (Norwegian Sea) (Fig. 31) from 315.9 to 316.6 m (this study).

#### Remarks

The presence of a ventrodistal long seta on the carpus of pereopods 2–3 separates *Hansenotanais partykae* n. sp. from *H. inermis*, which has only a short seta on the carpus of pereopods 2–3. See also remarks for *H. hansjacobi*.

### Key for identification of *Hansenotanais* neuters


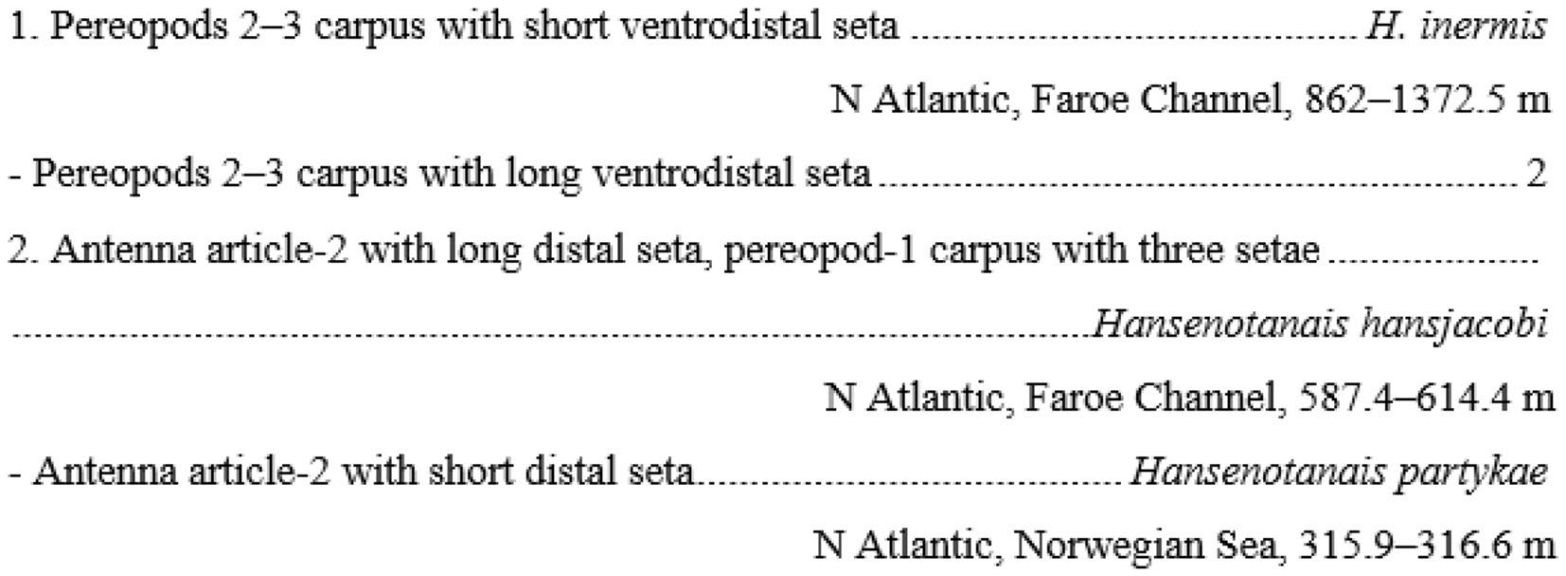

- ***Jurundurella*** **Gellert, Błażewicz & Bird n. gen.**
- LSID urn:lsid:zoobank.org:act:79BC9044-AE64-47D1-8219-EDF8B00D38FE.

#### Diagnosis

Body stout, pereonite margins straight. Antennule article-1 short (< 4.0 L:W), mesial margin with 4 ≤ setae. Maxilliped basis seta shorter than endites, endite cusps small. Cheliped carpus stout (< 2.5 L:W) carpus with short ventral seta. Pereopod-1 merus L:W stout (< 3.0 L:W), carpus without long seta; pereopods 2–3 carpus ventrodistal seta short, microtrichia regular (if present); pereopods 4–6 carpus with prickly tubercles, propodus distodorsal seta short, unguis bifurcate. Uropod endopod and exopod uniarticulate.

#### Type species

*Jurundurella bioice* n. sp. (by monotypy).

#### Etymology

The genus is name in honour of Prof. Jörundur Svavarsson, the scientific leader of the project BIOICE, acknowledged specialist in marine fauna and a great lover of Iceland's biodiversity.

#### Remarks

A combination of uniarticulate uropod rami and bifurcate pereopods 4–6 unguis makes *Jurundurella* similar to the *Brevitanais* group-1 and *Caesatanais*. However, *Jurundurella* has a short ventrodistal seta on the pereopods 2–3 carpus (long in *Caesatanais*), and straight pereonites (rounded in *Brevitanais*). *Jurundurella* has the pereopods 1–6 basis armed with several distinct spines. So far, similar spines have been observed in *Typhlotanais bolarticulus* Segadilha & Serejo, 2022, but present only on the basis of the pereopods 1–3. Besides, *Jurundurella* has a ‘third’ seta on the cheliped carpus, and small prickly tubercles in pereopods 4–6 carpus, while *Ty. bolarticulus* lacks the ‘third’ seta and has apparently large prickly tubercles.

**Figure 38:**
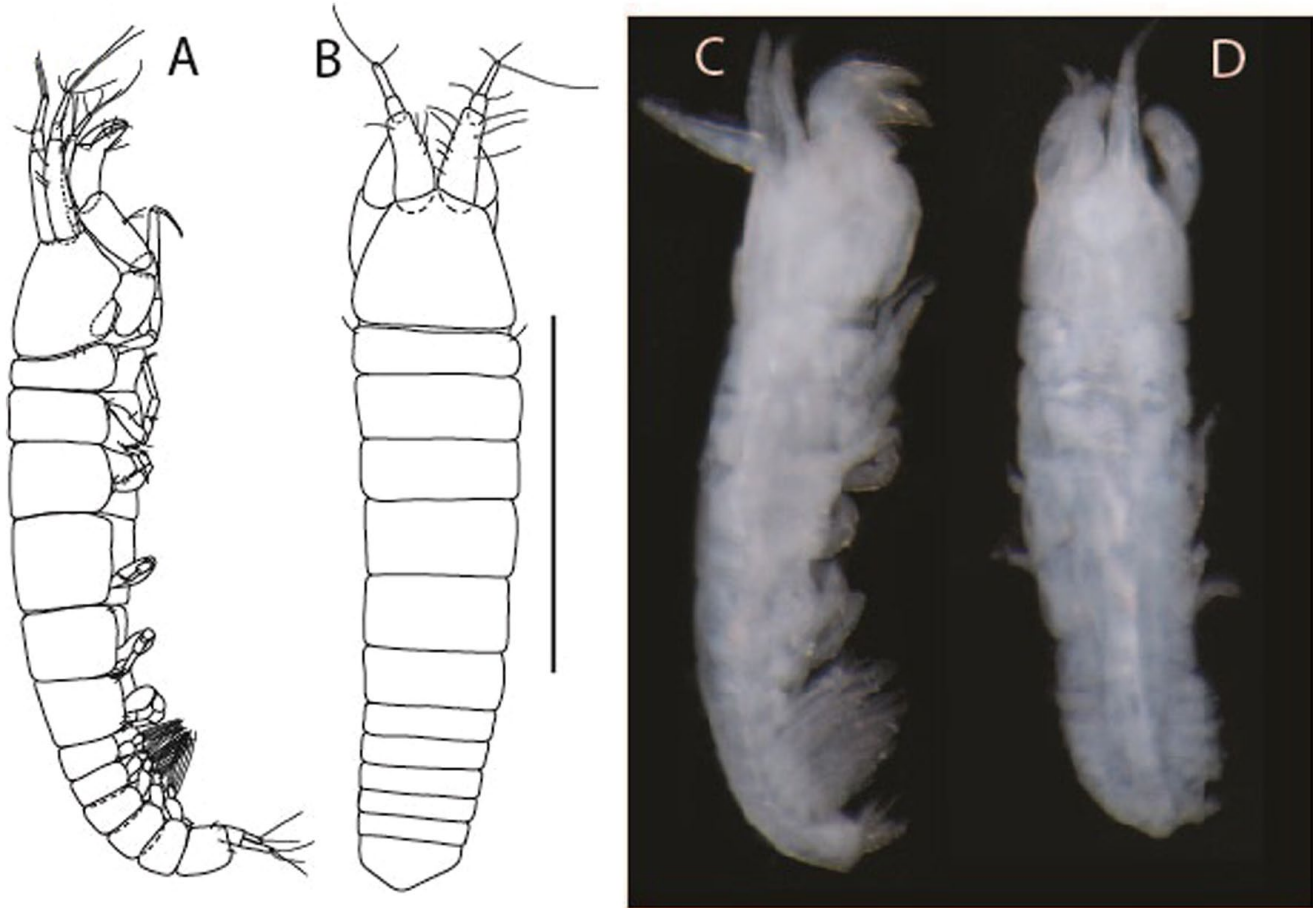
*Jurundurella bioice* n. sp., neuter (ICUL13955, ZMHK-64369), (**A**, **C**) Body, lateral view; (**B**, **D**) Body, dorsal view. Scale: A = 1 mm.

**Figure 39:**
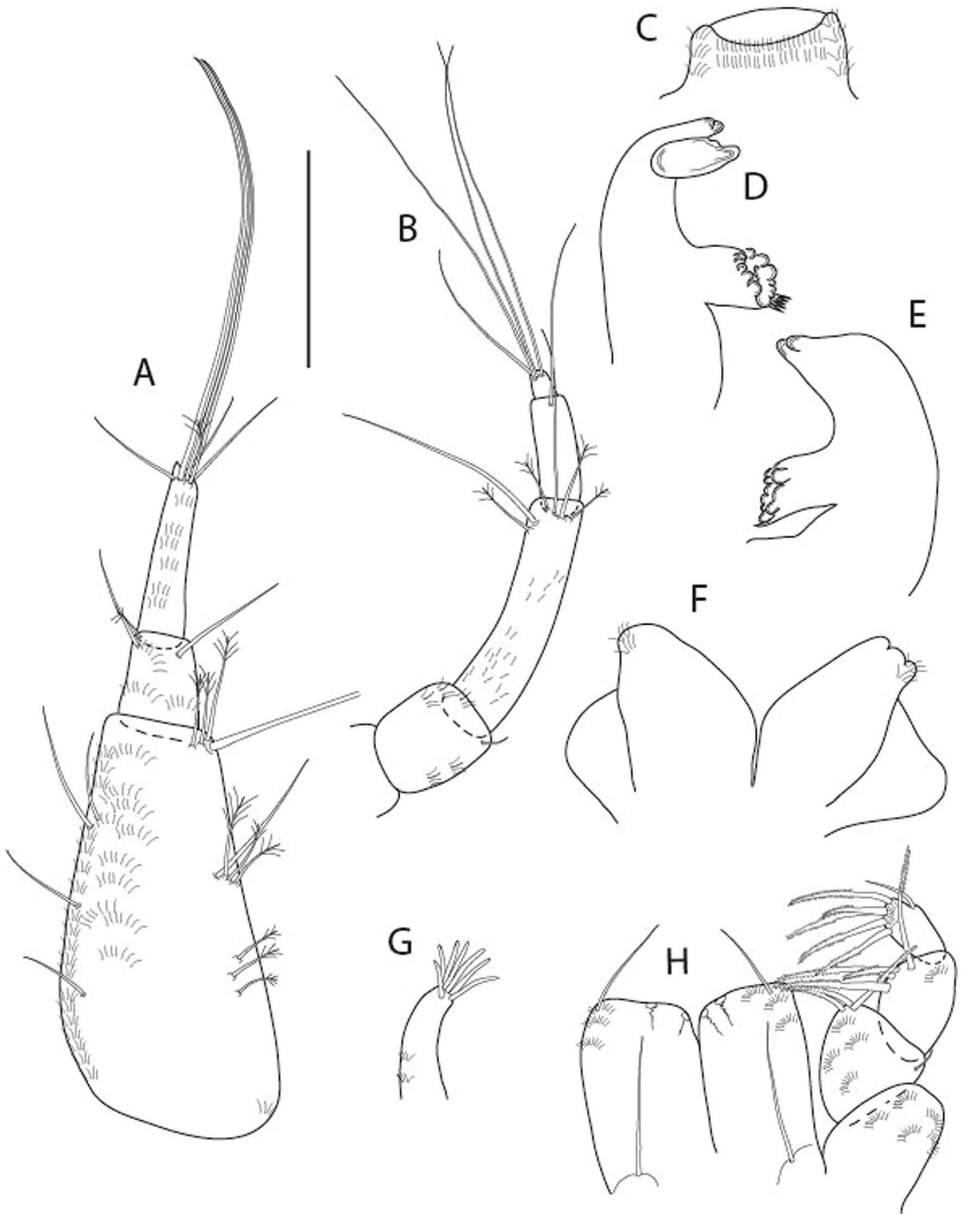
*Jurundurella bioice* n. sp., neuter (ICUL3395, ZMHK-64371), (**A**) Antennule; (**B**) Antenna; (**C**) Labrum; (**D**) Left mandible; (**E**) Right mandible; (**F**) Labium; (**G**) Maxillule; (**H**) Maxilliped. Scale: A–H = 0.1 mm.

**Figure 40:**
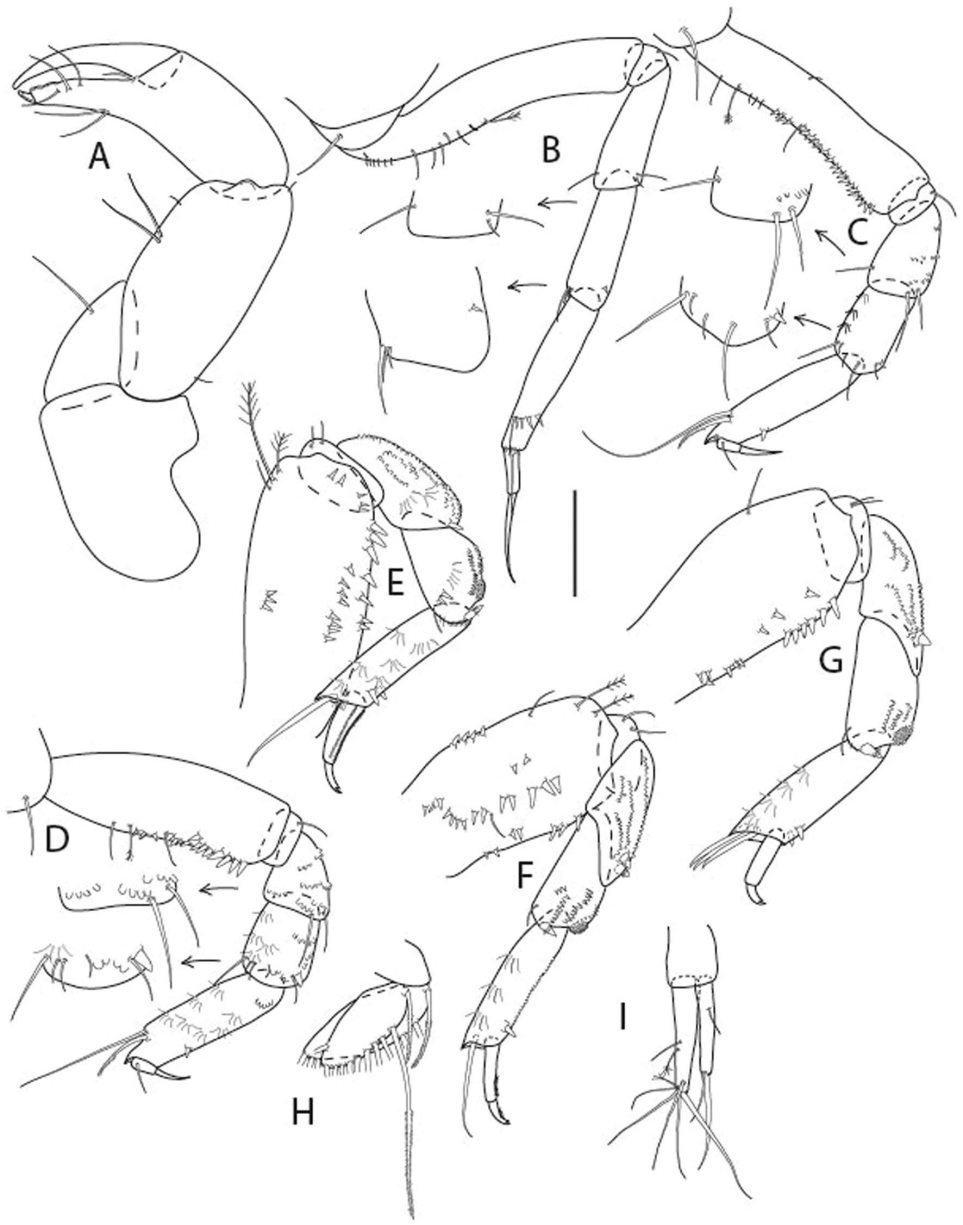
*Jurundurella bioice* n. sp., neuter (ICUL3395, ZMHK-64371), (**A**) Cheliped; (**B**) Pereopod-1; (**C**) Pereopod-2; (**D**) Pereopod-3; (**E**) Pereopod-4; (**F**) Pereopod-5; (**G**) Pereopod-6; (**H**) Pleopod; (**I**) Uropod. Scale: A–I = 0.1 mm.

**Figure 41:**
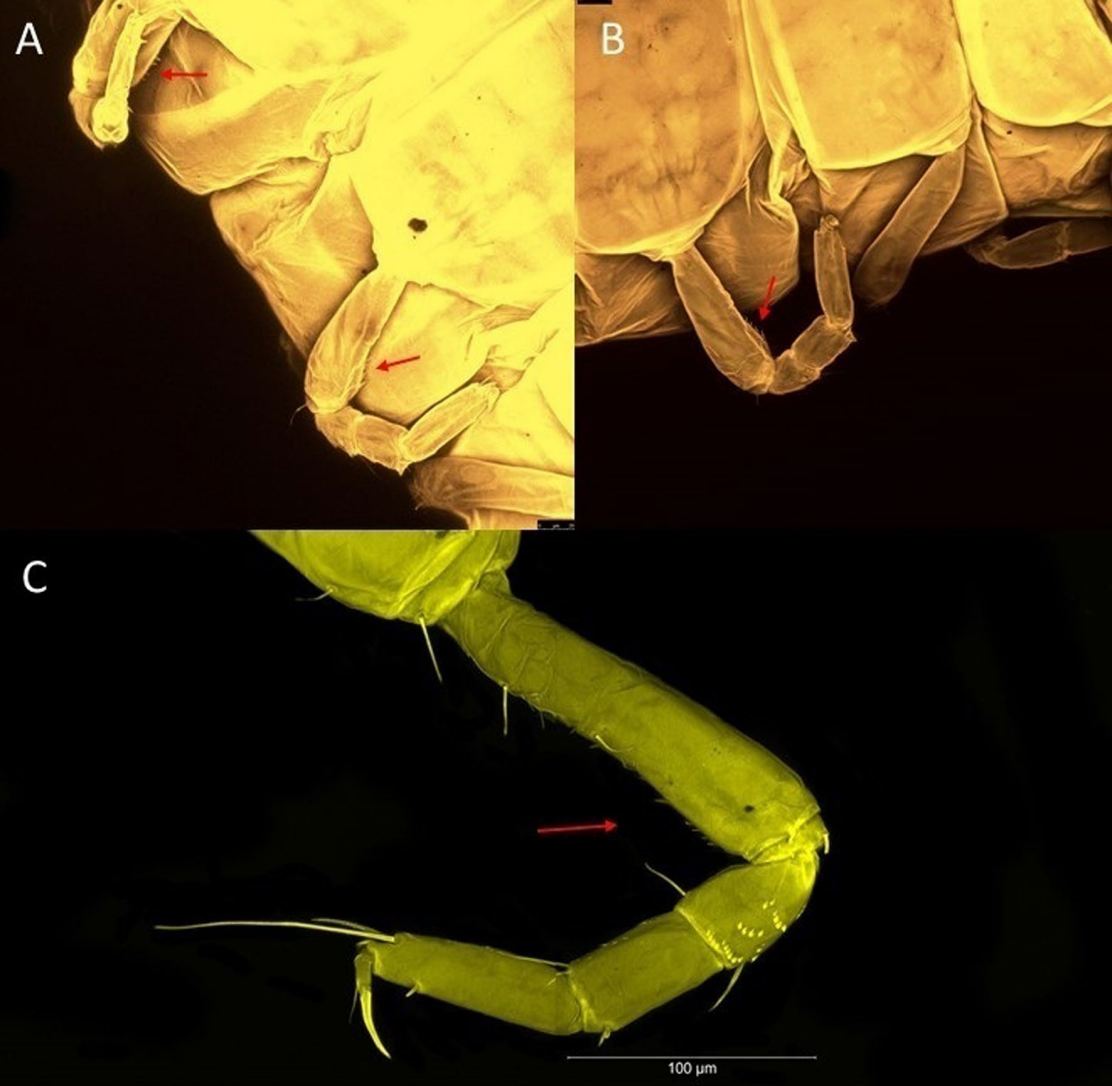
*Jurundurella bioice* n. sp., neuter (ICUL3395, ZMHK-64371), CLSM images: (**A**) Pereopods-4 and -5 basis with spines; (**B**) Pereopod-4 basis with spines; (**C**) Pereopod-4 basis with spines.

- ***Jurundurella bioice*** **Gellert, Błażewicz & Bird n. sp.**
- LSID urn:lsid:zoobank.org:act:53B9DB99-50D5-45A6-BA6B-006F81C00AD2.
- (Figs. 38, 39, 40 and 41).

#### Synonym

*Typhlotanais cornutus* Błażewicz et al. (2019)^67^: 4,7; Gellert et al. (2022)^28^: 1, 22–24.

#### Material examined

Holotype: neuter 1 mm IceAGE, St. 1072-1 (ICUL13955, ZMHK-64369).

Paratypes: IceAGE, St. 1010-1, neuter 1.8 mm (ICUL3356, ZMHK-64370; dissected); IceAGE, St. 1047-1, neuter 1.7 mm (ICUL3395, ZMHK-64371; dissected); IceAGE, St. 1072-1, neuter (ICUL2237, ZMHK-64372; broken); IceAGE, St. 1072-1, neuter 0.9 mm (ICUL10058, ZMHK-64373); IceAGE, St. 1072-1, neuter 1.1 mm (ICUL9828, ZMHK-64374; extraction).

#### Other material

AFEN 1996, St. 53726#1, neuter; BIOFAR, St. 264, seven neuters; BIOFAR, St. 27, two neuters; BIOICE, St. 2219, six neuters; BIOICE, St. 2288, two neuters; BIOICE, St. 2331, neuter; BIOICE, St. 2719, neuter; BIOICE, St. 2720, two neuters; BIOICE, St. 2873, two neuters; BIOICE, St. 3025, neuter; BIOICE, St. 3515, three neuters; DISCOVERY, St. 10111#8, two neuters; INCAL, St. CP04, neuter; INCAL, St. DS05, three neuters; INCAL, St. DS06, three neuters; SMBA, St. ES10, 97 neuters; SMBA, St. ES112, two neuters; SMBA, St. ES252, neuter; SMBA, St. ES34, 58 neuters; SMBA, St. ES99, neuter; SMBA, St. SBC155, neuter; SMBA, St. SBC66, two neuters; THALASSA 71, St. X305, three neuters; THALASSA 73, St. Z431, two neuters; THALASSA 73, St. Z435, neuter; THALASSA 73, St. Z453, neuter; THALASSA 73, St. Z459, neuter.

#### Diagnosis

As for the genus.

#### Etymology

This species is given after the project BIOICE.

#### Description of neuter,

Length 1.8 mm. Body (Fig. 38A−D) stout, 4.2 L:W. Cephalothorax trapezoidal, 0.8 L:W, 2.9× pereonite-1, with short lateral seta. Pereonites 1−6: 0.3, 0.4, 0.4, 0.5, 0.5 and 0.4 L:W, respectively; all pereonites rounded. Pereonite-1 0.6× pereonite-2, with short lateral seta; pereonite-2 as long as pereonite-3; pereonite-3 0.9× pereonite-4; pereonite-4 as long as pereonite-5; pereonite-5 1.3× pereonite-6. Pleon 0.2× BL; pleonites 1−5 of similar size – 0.2 L:W. Pleotelson 2.9× pereonite-6. Pleonites 1−5: all the same size – 0.3 L:W. Pleotelson 3.1× pereonite-6.

Antennule (Fig. 39A) 1.1× cephalothorax; article-1, 0.6 of antennule length, 2.7 L:W, with four setae and numerous microtrichia on inner margin, seta and six PSS on outer margin, and long seta (longer as article-2, broken) and three PSS distally; article-2 1.4 L:W, 0.2× article-1, with numerous microtrichia and PSS and distal seta on inner margin, and distal seta on outer margin; article-3 4.7 L:W, 1.8× article-2, with numerous microtrichia along article and PSS, three short and four long setae and terminal spur distally.

Antenna (Fig. 39B) article-2 broken during dissection; article-3 1.3 L:W, with distal seta and numerous microtrichia along article; article-4 4.9 L:W, 2.2× article-3, with numerous microtrichia along article and two long setae (longer than article-5) and four distal setae; article-5 4.2 L:W, 0.5× article-4, with long seta; article-6 1.0 L:W, with short seta and three long distal setae.

Mouthparts. Labrum (Fig. 39C) typical. Mandible (Fig. 39D−E) molar typical. Mandible left (Fig. 39D) incisor with two cusps, *lacinia mobilis* well developed, with two cusps; mandible right (Fig. 39E) incisor with one cusp. Labium (Fig. 39F) typical, outer corner of inner lobe and outer lobe with minute setae. Maxillule (Fig. 39G) endite with seven terminal spines, microtrichia along the article. Maxilla lost during dissection.

Maxilliped (Fig. 39H) endite cusps moderate, with seta, and microtrichia on outer margin; palp article-1 with numerous microtrichia; articles 2–3 typical, with microtrichia; article-4 typical. Epignath lost during dissection.

Cheliped (Fig. 40A) slender; basis 1.9 L:W, naked; merus seta long; carpus 2.0 L:W, with two long setae (shorter than carpus W) and short seta ventrally, dorsal margin setation typical; chela 1.1× carpus, 2.0 L:W; palm 1.5× fixed finger; fixed finger cutting edge with very weak, blunt distal cusp; dactylus with a short dorsoproximal seta.

Pereopod-1 (Fig. 40B) overall 15.2 L:W; coxa with seta; basis 5.9 L:W with PSS, four setae and nine short setae on dorsal margin; merus 3.0 L:W, with two ventrodistal setae (one short) and dorsodistal seta; carpus 3.2 L:W, 1.1× merus, with three dorsodistal setae and dorsodistal spine; propodus 5.0 L:W, 1.3× carpus, with seta and four spinules distally; dactylus 0.5× unguis, with seta, together 0.5× propodus.

Pereopod-2 (Fig. 40C) overall 12.8 L:W; coxa with seta; basis 4.8 L:W, with PSS, two setae and twenty-two small spines on dorsal margin and short subproximal seta on ventral margin; merus 1.7 L:W, with numerous calcified microtrichia along article and one dorsodistal and two ventrodistal setae; carpus 1.8 L:W; as long as merus, with three short and two long setae and small spine distally; propodus 4.2 L:W, 1.7× carpus, with two long, dorsodistal setae (one simple and one serrate) and ventrodistal spine; dactylus 0.6× unguis, with seta, together 0.4× propodus.

Pereopod-3 (Fig. 40D) similar to pereopod-2 but stouter, overall, 7.3 L:W; basis 2.9 L:W, with fifteen spines on dorsal margin; merus 1.2 L:W, with two ventrodistal setae; carpus 1.4 L:W, 1.2× merus, with numerous calcified microtrichia and microtrichia, three dorsodistal setae, ventrodistal seta, and spine; propodus 3.5 L:W, 1.9× carpus, with numerous microtrichia; dactylus 0.8× unguis, with seta, together 0.4× propodus.

Pereopod-4 (Figs. 39E, 41A−C) overall 5.5 L:W; basis robust, 2.2 L:W, with twenty-three spines at midlength, seta and two PPS ventrodistally; merus 2.1 L:W, with numerous calcified microtrichia along article and two distal spines; carpus 1.8 L:W, as long as merus, with moderate prickly tubercles, dorsodistal chemosensory seta, and distal spine/crotchet; propodus 4.2 L:W, with numerous microtrichia, two ventrodistal spines, and dorsodistal seta longer than claw; dactylus 2.1× unguis, together 0.7× propodus.

Pereopod-5 (Figs. 40F, 41A) similar to pereopod-4 but overall 5.1 L:W; basis 1.9 L:W, with twenty-seven spines and seta at midlength, and seta and two PSS ventrodistally; merus 2.8 L:W; carpus 2.1 L:W, 0.8× merus; propodus 3.6 L:W; dactylus 2.3× unguis, together 0.5× propodus.

Pereopod-6 (Fig. 40G) as pereopod-5, but basis with fifteen spines and midventral seta; propodus three dorsodistal setae longer than dactylus.

Pleopod (Fig. 40H) exopod with ten plumose setae on outer margin; endopod with fourteen.

Uropod (Fig. 40I) endopod with seta and a PSS at midlength, and PSS and five long terminal setae; exopod 1.2× endopod proximal article, with seta at midlength, the other setation typical.

#### Distribution

Known from 14 locations in the N Atlantic i.e. Celtic Slope, Faroe Plateau, Hebrides Slope, Iceland Basin, Iceland-Faroe Rise, Irminger Basin, N Biscay, N Feni Ridge, Porcupine Slope, Reykjanes Ridge, Rockall Trough, South Biscay Slope, S Feni Ridge, and W Shetland Slope, from the wide depth range 209.4–2540 m (Fig. 5) (this study).

- ***Sarsotanais*** **Gellert, Błażewicz & Bird n. gen.**
- LSID urn:lsid:zoobank.org:act:FCF16D42-BF73-4200-8E41-6EE328B015A8.

#### Diagnosis

Body stout, pereonite margins rounded. Antennule article-1 long (4.0 > L:W), inner margin with fewer than four setae. Maxilliped basis seta longer than endites, endite cusps small. Cheliped carpus slender (> 2.5 L:W), carpus with short ventral seta. Pereopod-1 merus L:W short (< 3.0 L:W), carpus two dorsodistal long setae; pereopods 2–3 carpus ventrodistal seta short, microtrichia large and calcified [spiniform]; pereopods 4–6 carpus with prickly tubercles, propodus distodorsal seta short, unguis bifurcate. Uropod endopod biarticulate, exopod uniarticulate.

#### Type species

*Sarsotanais georgi* n. sp. (by designation).

#### Etymology

Genus named in honour of George Ossian Sars Norwegian marine and freshwater biologist.

#### Species included

*Sarsotanais georgi* n. sp.; *Sarsotanais* sp. A (from Błażewicz-Paszkowycz, 2007^27^).

#### Remarks

The genus *Sarsotanais* n. gen. is defined as a ‘stout-bodied’ typhlotanaid group with its rounded-margin pereonites and the carpus of pereopods 2–3 carpus having spiniform, robust and calcified, microtrichia. The bifurcate unguis on the pereopods 4–6 distinguishes *Sarsotanais* from *Larsenotanais*, *Egregiella* and *Stuttotanais* (with simple pereopods 4–6 unguis), while the lack of seta on antenna article-3 separates it from *Caesatanais* and *G. gudmundssoni* that have a seta. Furthermore, the *Sarsotanais* has a short distodorsal seta on the propodus of pereopods 4–5, whereas all members of *Brevitanais* and *Caesatanais* have a long seta. Finally, the *Sarsotanais* has a long seta on the pereopod-1 carpus, in contrast to *Jurundurella bioice* and *Stuttotanais frenchae*, with a short seta.

**Figure 42:**
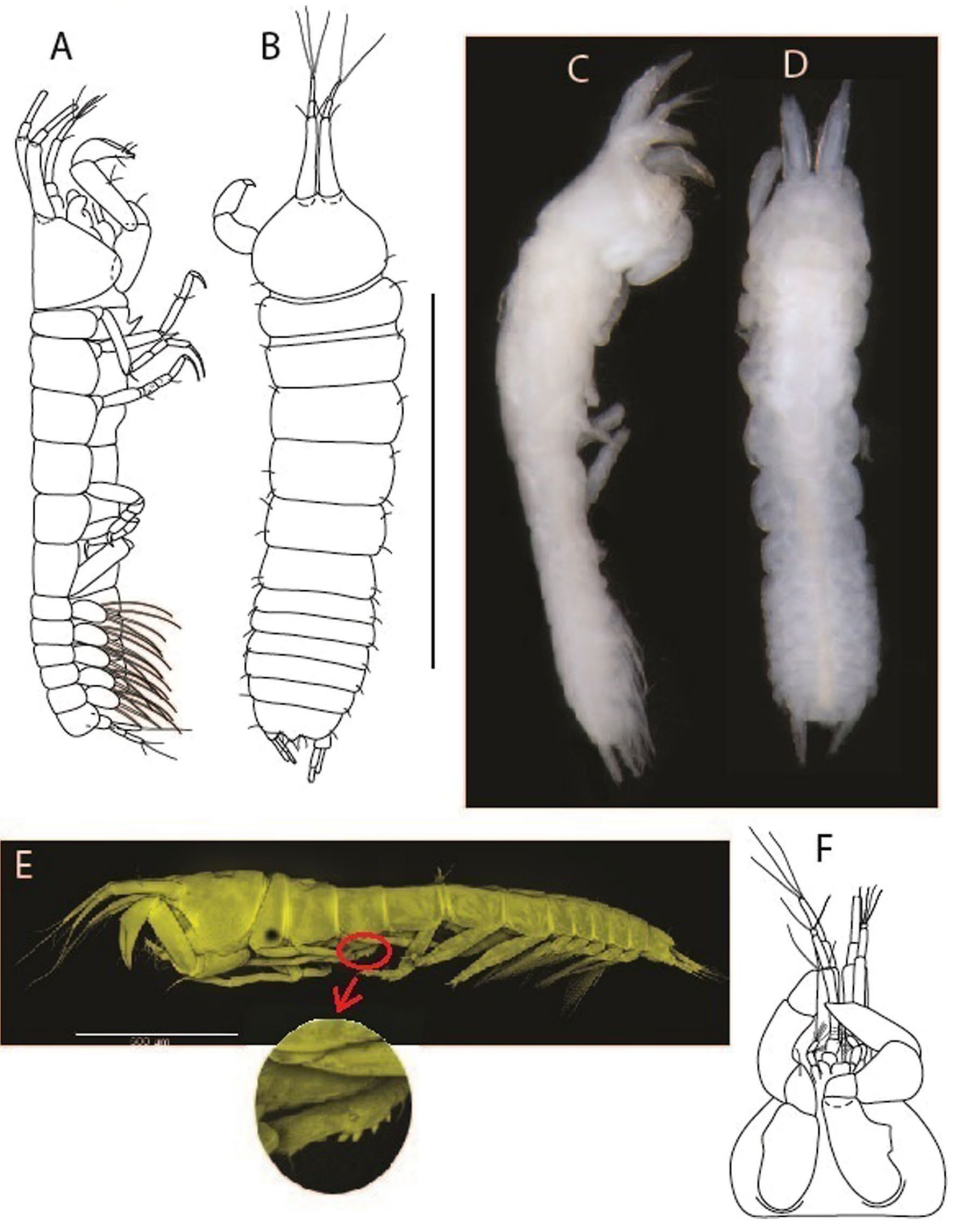
*Sarsotanais georgi* n. sp., neuter (ICUL8957, ZMHK-64375), (**A**, **C**) Body, lateral view; (**B**, **D**) Body, dorsal view; (**E**) CLSM images: Body, lateral view, pereopod-3 merus with numerous spiniform nodules; (**F**) Carapace ventral side. Scale: A, B = 1 mm., E = 0.2 mm.

**Figure 43:**
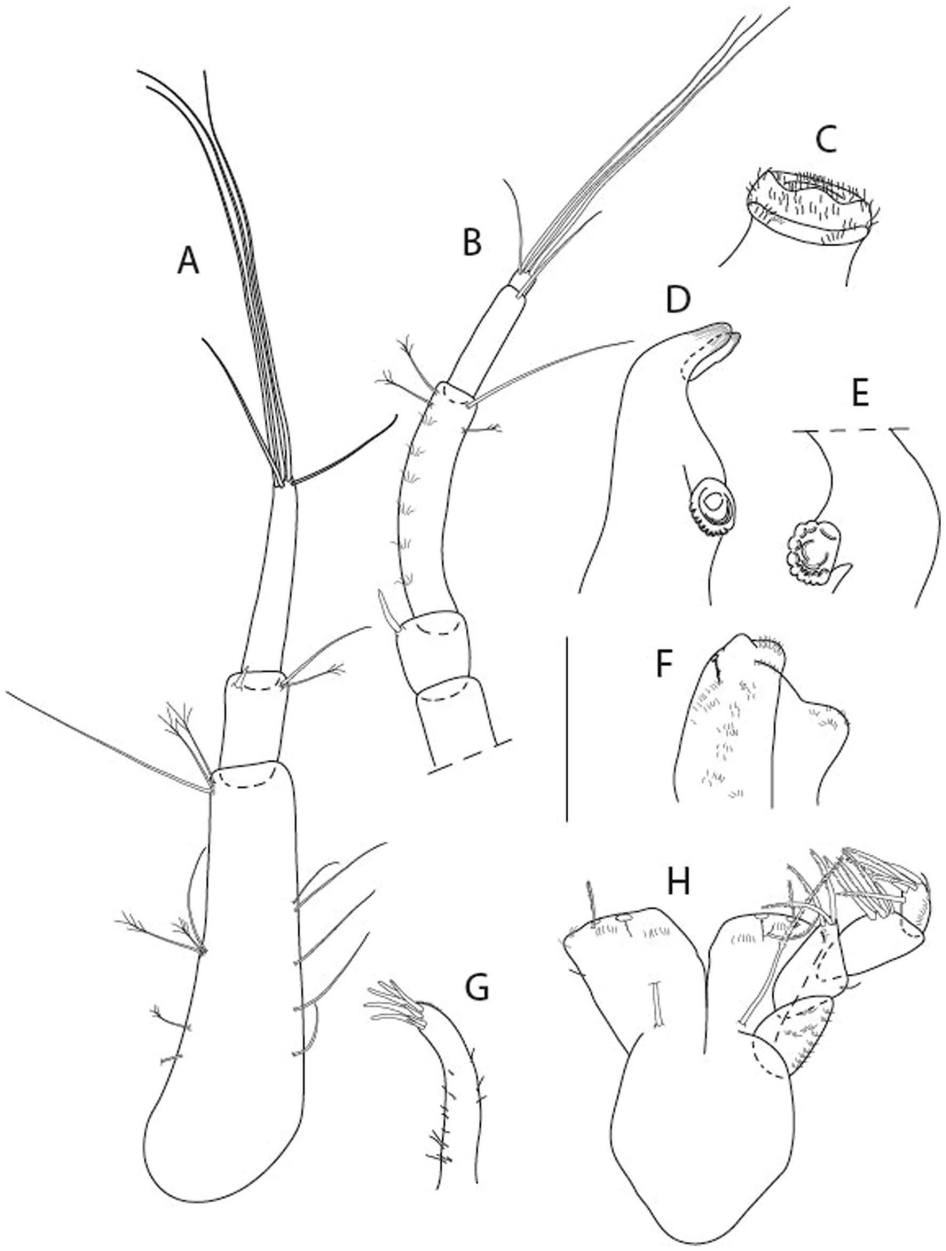
*Sarsotanais georgi* n. sp., neuter (ICUL3566, ZMHK-64375), (**A**) Antennule; (**B**) Antenna; (**C**) Labrum; (**D**) Left mandible; (**E**) Right mandible; (**F**) Labium; (**G**) Maxillule; (**H**) Maxilliped. Scale: A–H = 0.1 mm.

**Figure 44:**
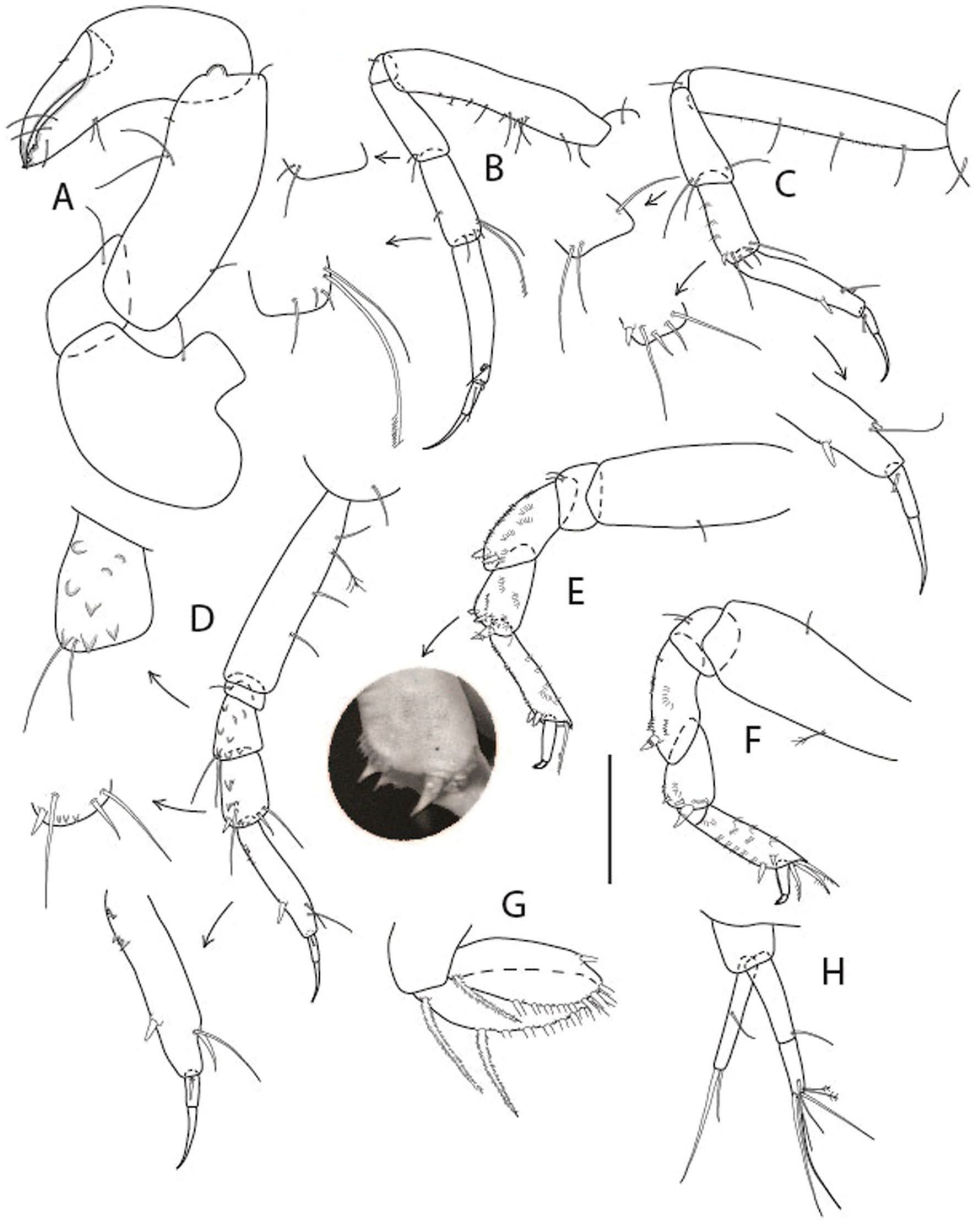
*Sarsotanais georgi* n. sp., neuter (ICUL3566, ZMHK-64377), (**A**) Cheliped; (**B**) Pereopod-1; (**C**) Pereopod-2; (**D**) Pereopod-3; (**E**) Pereopod-5; (**F**) Pereopod-6; (**G**) Pleopod; (**H**) Uropod. Scale: A–I = 0.1 mm.

- ***Sarsotanais georgi*** **Gellert, Błażewicz & Bird n. sp.**
- LSID urn:lsid:zoobank.org:act:77BA4134-34D4-4133-B207-1C466CBC3536.
- (Figs. 42, 43 and 44).

#### Synonym

*Typhlotanais eximius* Błażewicz et al. (2019)^67^: 4,7; Gellert et al. (2022)^28^: 1, 22–24.

#### Material examined

Holotype, neuter 1.6 mm, IceAGE, St. 963-1 (ICUL8957, ZMHK-64375).

Paratypes IceAGE, St. 1010-1, two neuters 1−1.5 mm (ICUL9015, ZMHK-64376); IceAGE, St. 1072-1, neuter (ICUL2238, broken, extraction*); IceAGE, St. 1072-1, neuter 1.1 mm (ICUL13948, extraction*); IceAGE, St. 1072-1, neuter 1.1 mm (ICUL3566, ZMHK-64377; dissected); IceAGE, St. 1072-1, neuter 1.2 mm (ICUL10115, ZMHK-64378; extraction); IceAGE, St. 1072-1, neuter 1.2 mm (ICUL9979, ZMHK-64379); IceAGE, St. 1072-1, neuter 1.5 mm (ICUL9983, ZMHK-64380); IceAGE, St. 1072-1, neuter 1.6 mm (ICUL1720, ZMHK-64382); IceAGE, St. 983-1, neuter 1.5 mm (ICUL10010, ZMHK-64381; extraction).*Individual not recovered after DNA extraction.

#### Other material

CHAIN, St. 106, 316, 18 neuters; CHAIN, St. 106, 318, 18 neuters; CHAIN, St. 106, 321, four neuters; CHAIN, St. 106, 323, seven neuters; CHAIN, St. 106, 326, 16 neuters; DISCOVERY, St. 10112#3, neuter; DISCOVERY, St. 7709#73, six neuters; DISCOVERY, St. 7709#85, neuter; INCAL, St. CP04, two neuters; INCAL, St. CP06, three neuters; INCAL, St. CP07, five neuters; INCAL, St. CP08, two neuters; INCAL, St. CP09, neuter; INCAL, St. CP10, two neuters; INCAL, St. DS05, 18 neuters; INCAL, St. DS06, six neuters; INCAL, St. DS07, neuter; INCAL, St. DS10, neuter; INCAL, St. OS01, two neuters; INCAL, St. WS01, five neuters; INCAL, St. WS02, 90 neuters; INCAL, St. WS03, three neuters. INCAL, St. WS04, neuter; INCAL, St. DS09, 15 neuters; POLYGAS, St. DS21, two neuters; SARSIA, St. n50, five neuters; SMBA, St. ES10, 16 neuters; SMBA, St. ES10, 194 neuters; SMBA, St. ES118, five neuters; SMBA, St. ES129, seven neuters; SMBA, St. ES135, six neuters; SMBA, St. ES137, 14 neuters; SMBA, St. ES140, two neuters; SMBA, St. ES147, 40 neuters; SMBA, St. ES152, three neuters; SMBA, St. ES164, six neuters; SMBA, St. ES172, two neuters; SMBA, St. ES180, four neuters; SMBA, St. ES180, two neuters; SMBA, St. ES185, neuter; SMBA, St. ES185, six neuters; SMBA, St. ES190, neuter; SMBA, St. ES190, neuter; SMBA, St. ES197, neuter; SMBA, St. ES204, four neuters; SMBA, St. ES207, eight neuters; SMBA, St. ES207, neuter; SMBA, St. ES231, two neuters; SMBA, St. ES27, neuter; SMBA, St. ES283, eight neuters; SMBA, St. ES283, neuter; SMBA, St. ES285, neuter; SMBA, St. ES34, 78 neuters; SMBA, St. ES34, six neuters; SMBA, St. ES56, neuter; SMBA, St. ES56, neuter; SMBA, St. ES59, neuter; SMBA, St. ES59, neuter.

#### Diagnosis

Body short and compact; cephalothorax 0.7 L:W. Pereonite-1 0.3 L:W. Cheliped carpus 2.9 L:W. Pereopods 1–3 basis with numerous setae at midlength. Uropod exopod 0.7× endopod.

#### Etymology

Species named in honour of George Ossian Sars, Norwegian marine and freshwater biologist.

#### Description of neuter,

Length 1.4 mm. Body (Fig. 42A−E) very stout, 3.8 L:W. Cephalothorax round, 0.8 L:W, 2.6× pereonite-1, with short lateral seta. Pereonites 1−6: 0.3, 0.3, 0.4, 0.5, 0.4 and 0.4 L:W, respectively. Pereonite-1 oval, 0.4× pereonite-2; pereonite-2 trapezoidal, 0.8× pereonite-3; pereonite-3 rounded laterally, 0.9× pereonite-4; pereonite-4 rounded laterally, 1.3× pereonite-5; pereonite-5 trapezoidal, 1.1× pereonite-6; pereonite-6 trapezoidal. Pereonites 1−3 with short lateral seta, pereonites 4−6 with two short lateral setae. Pleon 0.3× total BL; pleonites 1−5: of similar size – 0.2 L:W, with small lateral seta. Pleotelson 3.4× pereonite-6.

Antennule (Fig. 43A) 1.3× cephalothorax; article-1 0.6 of antennule length, 4.7 L:W, with five setae on inner margin, seta and four PSS (one broken) on outer margin and long seta (longer as article-2) and two PSS distally; article-2 2.0 L:W, 0.3× article-1, with distal spine on outer margin, seta, and distal PSS on inner margin; article-3 6.4 L:W, 1.8× article-2, with two short and three long setae distally.

Antenna (Fig. 43B) article-2 1.2 L:W, naked; article-3 1.2 L:W, 0.7× article-2, with distal spine; article-4 5.2 L:W, 2.8× article-3, with microtrichia along article and distal long seta (longer than article-5) and three PSS distally; article-5 4.8 L:W, 0.5× article-4, with long seta; article-6 1.0 L:W, with short seta and three long distal setae.

Mouthparts. Labrum (Fig. 43C) typical. Mandible (Fig. 43E) molar typical; left mandible (Fig. 43D) incisor with single cusp, *lacinia mobilis* well developed, with single cusp; right mandible (Fig. 43E) incisor broken during dissection. Labium (Fig. 43F) typical, outer corner of inner lobe and outer lobe with minute setae. Maxillule (Fig. 43G) endite typical, three innermost spines shorter than the others, microtrichia along article. Maxilla lost during dissection.

Maxilliped (Fig. 43H) basis finely serrate seta reaching beyond endites; endite cusps moderate, with serrate seta, and microtrichia on outer margin; palp article-1 with numerous microtrichia; articles 2–4 typical. Epignath lost during dissection.

Cheliped (Figs. 42F, 44A) slender; basis 1.7 L:W, with dorsolateral seta; merus (seta about merus W); carpus 2.7 L:W, with two long setae (about carpus W) and short seta ventrally, dorsal margin setation typical; chela subequal carpus, 2.9 L:W; palm 1.1× fixed finger; fixed finger cutting edge with four weak, distal blunt cusps; dactylus with a short dorsoproximal seta.

Pereopod-1 (Fig. 44B) overall 13.8 L:W; coxa with seta; basis 5.8 L:W with seven seta and four spines; merus 2.8 L:W with two ventrodistal setae; carpus 3.0 L:W; 0.9× merus, with three setae and two long setae (one simple and one serrate) distally; propodus 5.5 L:W, 1.5× carpus, with three distal setae; dactylus 0.6× unguis, with seta, 0.6× propodus.

Pereopod-2 (Fig. 44C) overall 13.7 L:W; coxa with seta; basis with three setae and numerous short setae; merus 3.0 L:W, with three distal setae (one dorsodistal and two ventrodistal); carpus 2.3 L:W, 1.1× merus, with numerous microtrichia along article, spine, two short and two long distal setae; propodus 5.3 L:W, 0.7× merus and carpus combined, with two dorsodistal setae and ventrodistal spine; dactylus 0.6× unguis, with seta, together 0.6× propodus.

Pereopod-3 (Fig. 44D) similar to pereopod-2 but slightly stouter, overall, 11.6 L:W; basis 4.1 L:W, with middorsal PSS and three setae; merus 1.7 L:W, with numerous spiniform nodules (Fig. 41E) and two ventrodistal setae; carpus 1.7 L:W, as long as merus, with numerous spiniform nodules (but fewer than merus) and spine, short and two long distal setae; propodus 5.0 L:W, 1.7× carpus; dactylus 0.8× unguis, with seta (broken), together 0.6× propodus.

Pereopod-4 as pereopod-5.

Pereopod-5 (Fig. 44E) overall 6.2 L:W; basis slender, 4.2 L:W, with middorsal seta; merus 2.4 L:W, with two distal serrate spines, and numerous microtrichia along article; carpus 2.0 L:W, 0.8× merus, with small prickly tubercles, dorsodistal chemosensory seta, and three distal spines/crotchets, one larger than the others; propodus 4.8 L:W, with numerous microtrichia, two ventrodistal serrate spines, and dorsodistal serrate seta shorter than claw; dactylus 2.5× unguis, together 0.4× propodus.

Pereopod-6 (Fig. 44F) as pereopod-5, but basis with a middorsal PSS and midventral seta; propodus three dorsodistal setae as long as claw.

Pleopod (Fig. 44G) exopod with ten plumose setae on outer margin; endopod with twelve.

Uropod (Fig. 44H) endopod about 7.0 L:W, proximal article 2.4× distal article, with distal seta; distal article with two PSS and five long terminal setae; exopod 1.3× endopod proximal article, with seta at midlength, the other setation typical.

#### Distribution

Known from nine locations off Iceland, the Abyssal N Biscay, Abyssal Porcupine, Denmark Strait, Hebrides Slope, Iceland Basin, Irminger Basin, N Rockall Trough, Porcupine Abyssal Plain, Porcupine Seabright, Porcupine Slope, Reykjanes Ridge, and the Rockall Trough (Fig. 31), at depths of 1074–4829 m (this study).

#### Remarks

*Sarsotanais georgi* n. sp. and *Sarsotanais* (= *Typhlotanais*) sp. A (recorded by Błażewicz-Paszkowycz from the Antarctic) share: similar body habit, rounded margins of pereonites, wider than long, pereonites 3–5 subequal and pereonite-1 shorter than the others with a sternal spur (hyposphenium). Nevertheless the species can be distinguished by the length of the cheliped carpus that is relatively stout (2.9 L:W) in *Sa. georgi* and more slender (3.6 L:W) in *Sarsotanais* sp. A. Also, the *Sa. georgi* species has many setae on the basis of pereopods 1–3, where *Sarsotanais* sp. A has only three.

- ***Sarsotanais*** **sp. A**
- *Typhlotanais* sp. A Błażewicz-Paszkowycz, 2007^27^ 1598: 132–135.

#### Diagnosis

Body stout and compact; cephalothorax 0.8 L: W; pereonite-1 0.2 L: W; cheliped carpus 3.6 L:W; pereopods 1–3 basis with three setae at midlength; uropod exopod 0.8× endopod.

#### Distribution

E Weddell Sea, at depths of 4390–4392 m^27^.

#### Remarks

See the remarks for *Sarsotanais georgi*.

- ***Stuttotanais*** **Gellert, Błażewicz & Bird n. gen.**
- LSID urn:lsid:zoobank.org:act:35A950C7-FFBC-4C92-8A9E-324452C5E178.

#### Diagnosis

Body stout, pereonites margin rounded. Antennule article-1 short (< 4.0 L:W), mesial margin with three setae. Maxilliped basis seta shorter than endites, endites cusps small. Cheliped carpus long (> 2.5 L:W), carpus with short ventral seta. Pereopod-1 merus L:W short (< 3.0 L:W), carpus without long seta; pereopods 2−3 carpus ventrodistal seta short, microtrichia calcified; pereopods 4−6 carpus with prickly tubercles, unguis bifurcate, propodus distodorsal seta short. Uropod endopod biarticulate, exopod uniarticulate.

#### Etymology

 Stuttur [is.] means short, reflecting the stout habitus of species classified in this genus.

#### Type species

*Stuttotanais carringtonae* n. sp.

#### Species included

*Stuttotanais carringtonae* n. sp.; *Stuttotanais frenchae* n. sp.

**Figure 45:**
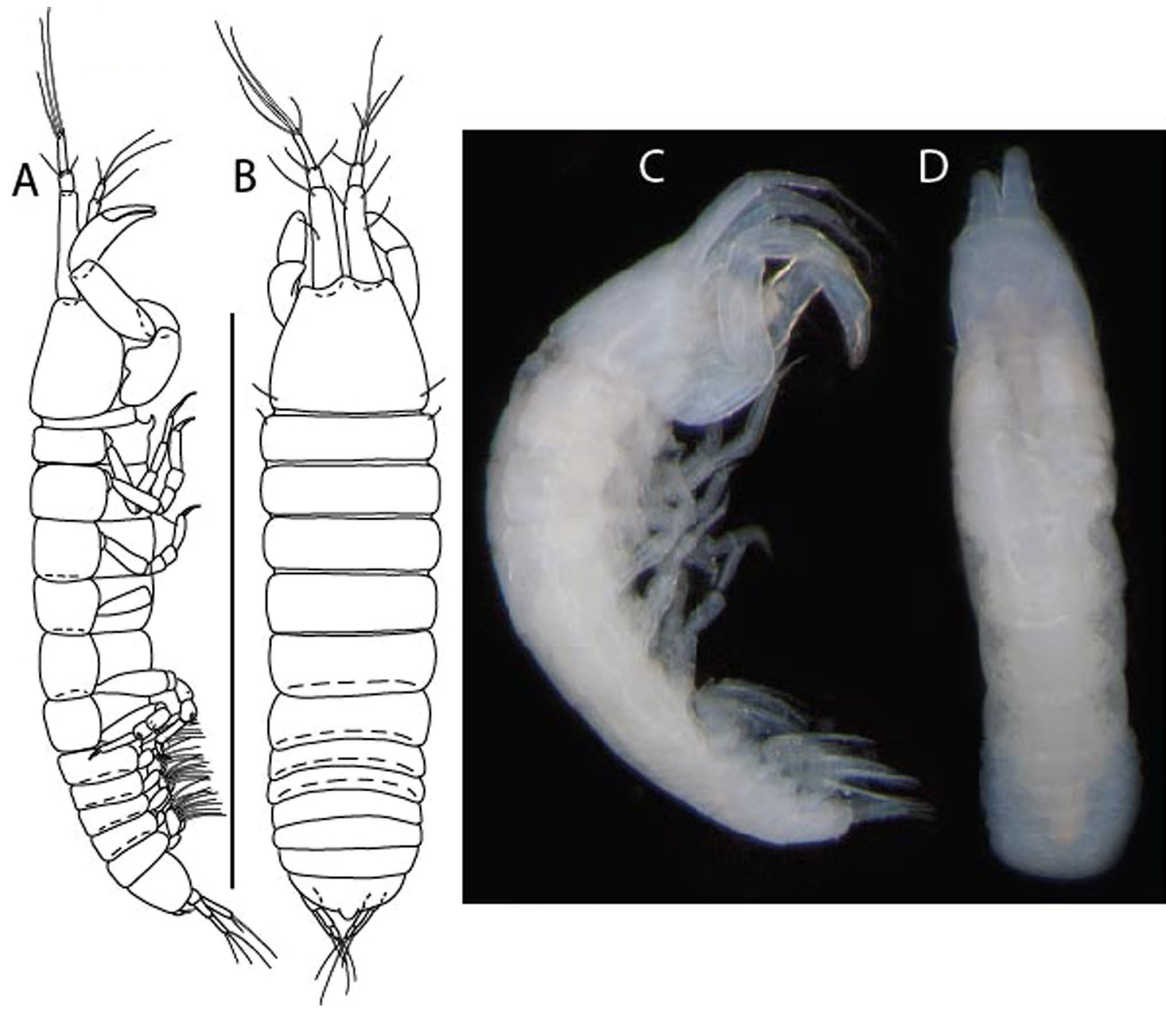
*Stuttotanais carringtonae* n. sp., neuter (ICUL13956, ZMHK-64383), (**A**, **C**) Body, lateral view; (**B**, **D**) Body, dorsal view. Scale: A = 1 mm.

**Figure 46:**
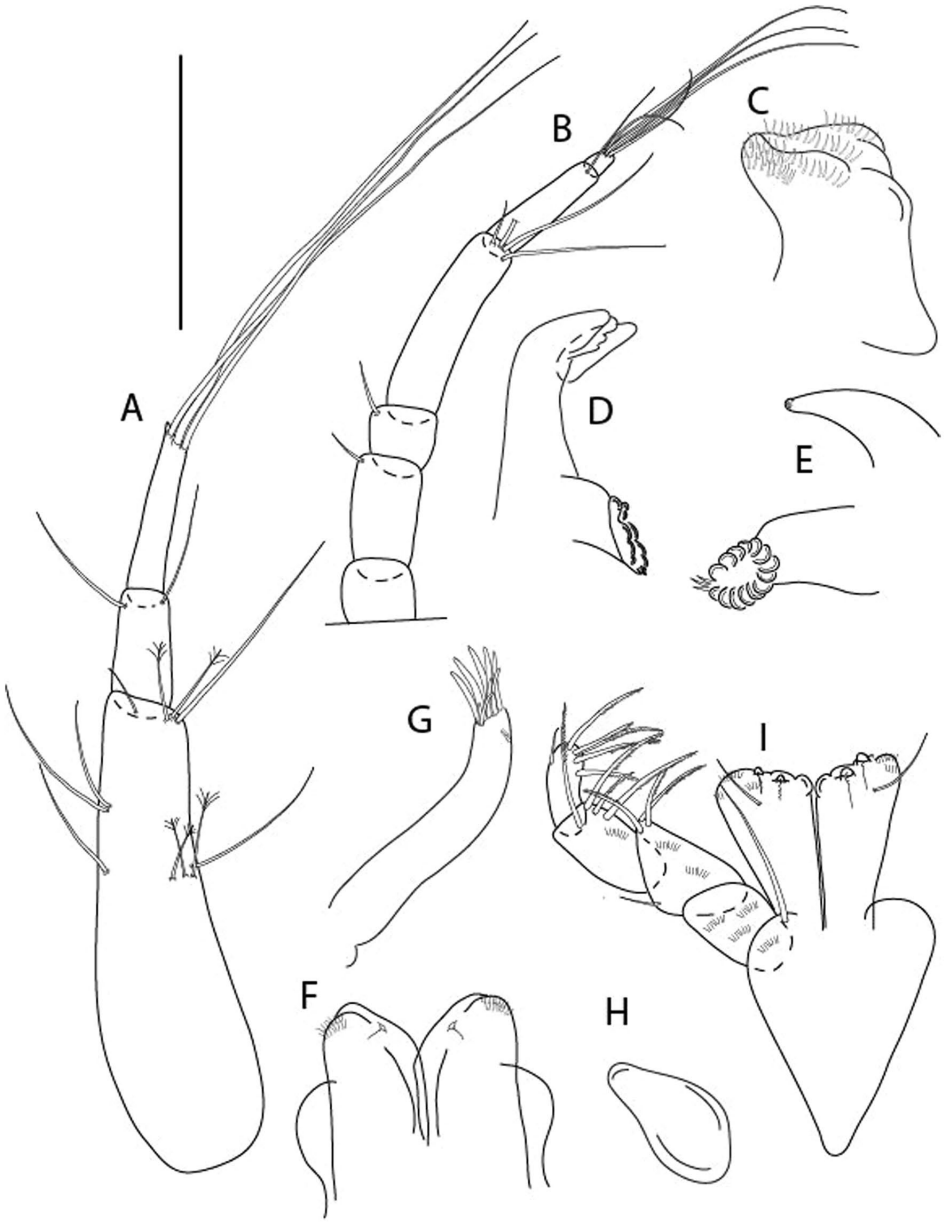
*Stuttotanais carringtonae* n. sp., neuter (ICUL13962, ZMHK-64384), (**A**) Antennule; (**B**) Antenna; (**C**) Labrum; (**D**) Left mandible; (**E**) Right mandible; (**F**) Labium; (**G**) Maxillule; (**H**) Maxilla; (**H**) Maxilliped. Scale: A–I = 0.1 mm.

**Figure 47:**
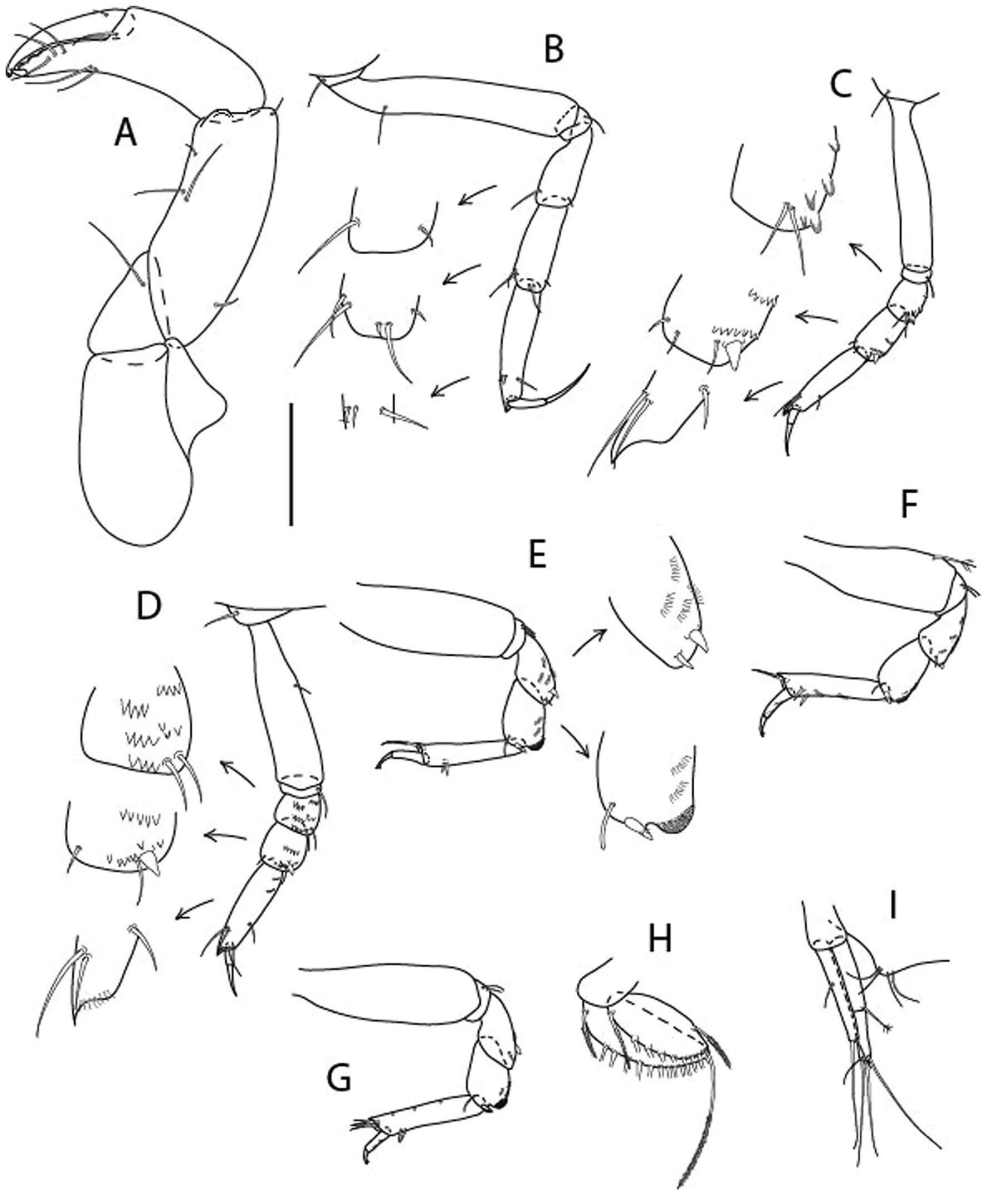
*Stuttotanais carringtonae* n. sp., neuter (ICUL13962, ZMHK-64384), (**A**) Cheliped; (**B**) Pereopod-1; (**C**) Pereopod-2; (**D**) Pereopod-3; (**E**) Pereopod-4; (**F**) Pereopod-5; (**G**) Pereopod-6; (**H**) Pleopod; (**I**) Uropod. Scale: A–I = 0.1 mm.

- ***Stuttotanais carringtonae*** **Gellert & Błażewicz n. sp.**
- LSID urn:lsid:zoobank.org:act:72F32C6B-038D-4D3D-8DE1-E9C410BDC6D2.
- (Figs. 45, 46 and 47).

#### Material examined

Holotype: neuter 1.1 mm, IceAGE II, St. 867-1 (ICUL13956, ZMHK-64383). Paratypes: IceAGE II, 867-1, neuter 1.1 mm (ICUL13962, ZMHK-64384; dissected); IceAGE II, 867-1, neuter 1.1 mm (ICUL9005, ZMHK-64385).

#### Diagnosis

Body stout and compact, pereonite margins rounded. Antennule article-1 long (> 4.0 L:W), mesial margin with three setae. Maxilliped basis seta shorter than endites, endite cusps small. Cheliped carpus slender (> 2.5 L:W), carpus with short ventral seta. Pereopod-1 merus L:W stout (< 3.0 L:W), carpus without long seta; pereopods 2−3 carpus ventrodistal seta short, microtrichia calcified; pereopods 4–6 carpus with prickly tubercles, propodus distodorsal seta short, unguis simple. Uropod endopod biarticulate, exopod uniarticulate.

#### Etymology

This species is dedicated to Dame Lisa Marie Carrington – flatwater canoeist and New Zealand’s most successful Olympian.

#### Description of neuter,

Length 1.1 mm. Body (Fig. 45A−D) stout, 4.3 L:W. Cephalothorax trapezoidal, 0.8 L:W, 3.2× pereonite-1, with short lateral seta. Pereonites 1−6: 0.2, 0.3, 0.3, 0.4, 0.4 and 0.4 L:W, respectively; all pereonites rounded. Pereonite-1 0.6× pereonite-2, with short lateral seta; pereonite-2 0.9× pereonite-3; pereonite-3 0.9× pereonite-4; pereonite-4 0.9× pereonite-5; pereonite-5 1.1× pereonite-6. Pleon 0.3× BL; pleonites 1−5: of similar size – 0.2 L:W. Pleotelson 3.4× pereonite-6.

Antennule (Fig. 46A) 1.2× cephalothorax; article-1 0.6 of antennule length, 4.2 L:W, with three setae at midlength on inner margin, seta and three PSS at midlength on outer margin, and two setae (one longer than article-2) and two PSS distally; article-2 2.4 L:W, 0.3× article-1, with two distal setae (one on inner and one on outer margin); article-3 5.8 L:W, 1.4× article-2, with three terminal setae and terminal spur.

Antenna (Fig. 46B) article-2 1.8 L:W, with distal seta; article-3 1.1 L:W, 0.6× article-2, with distal seta; article-4 4.0 L:W, 2.9× article-3, with four distal setae (two longer than article-5, one broken); article-5 4.2 L:W, 0.5× article-5, with long seta; article-6 1.6 L:W, with five distal setae.

Mouthparts. Labrum (Fig. 46C) typical. Mandible (Fig. 46D−E) molar typical. Left mandible (Fig. 46D) incisor with two cusps, *lacinia mobilis* well developed, with single cusp; right mandible (Fig. 46E) incisor with single cusp. Labium (Fig. 46F) typical, outer corner of inner lobe, with cusps. Maxillule (Fig. 46G) endite with nine terminal spines, four innermost spines shorter than the others. Maxilla (Fig. 46H) oval.

Maxilliped (Fig. 46I) basis 1.8 L:W; endite cusps moderate; palp article-1 with microtrichia along article; article-2 typical, with microtrichia along article; article-3 typical, with microtrichia along article; article-4 typical.

Cheliped (Fig. 47A) slender; basis 1.8 L:W, naked; merus seta long; carpus 2.7 L:W, with two long setae (shorter than carpus W) and short seta ventrally, dorsal margin setation typical; chela longer than carpus, 3.7 L:W; palm 1.6× fixed finger; fixed finger cutting edge with three weak, distal blunt cusps; dactylus slightly curved, with seta.

Pereopod-1 (Fig. 47B) overall 16.8 L:W; coxa with seta, basis 6.5 L:W, with dorsoproximal seta; merus 2.3 L:W with single dorsodistal and ventrodistal short setae; carpus 2.8 L:W, 1.1× merus, with five distal setae (two longer than the others); propodus 4.7 L:W, 1.3× carpus, with two dorsodistal setae (one broken) and ventrodistal seta; dactylus 0.7× unguis, together 0.7× propodus.

Pereopod-2 (Fig. 47C) overall 12.5 L:W; coxa with seta; basis 5.5 L:W, naked; merus 1.6 L:W, with two ventrodistal setae and numerous calcified microtrichia along article; carpus 1.9 L:W, 1.2× merus, with two dorsodistal setae, ventrodistal seta and spine, and calcified microtrichia along article; propodus 4.7 L:W, 0.9× merus and carpus combined, with two dorsodistal setae and ventrodistal seta; dactylus 0.7× unguis, together 0.5× propodus.

Pereopod-3 (Fig. 47D) similar to pereopod-2 but stouter, overall, 8.2 L:W; basis 3.9 L:W, with midventral seta; merus 1.1 L:W; carpus 1.3 L:W, 1.2× merus, with dorsodistal short seta and seta and spine ventrodistally; propodus 3.8 L:W, 2.0× carpus; dactylus 0.7× unguis.

Pereopod-4 (Fig. 47E) overall 7.5 L:W; basis robust, 2.9 L:W, naked; merus 2.2 L:W, with two distal spines, and microtrichia along article; carpus 1.9 L:W, as long as merus, with moderate prickly tubercles, and dorsodistal chemosensory seta, and distal spine/crotchet; propodus 5.2 L:W, with two ventrodistal spines, dorsodistal seta longer than dactylus; dactylus 3.4× unguis, together 0.5× propodus.

Pereopod-5 (Fig. 47F) similar to pereopod-4 but overall 7.2 L:W; basis robust, 2.7 L:W, with PSS; merus 1.8 L:W; carpus 3.6 L:W; propodus 4.8 L:W, with numerous microtrichia along article.

Pereopod-6 (Fig. 47G) as pereopod-5, but basis naked; propodus three dorsodistal setae about as long as dactylus.

Pleopods (Fig. 47H) exopod with ten plumose setae on outer margin; endopod with fourteen.

Uropod (Fig. 47I) endopod proximal article 1.4× distal article, with a distal PSS; distal article with four long terminal setae; exopod 1.4× endopod proximal article, with seta at midlength, the other setation typical.

#### Distribution

Known from one location off Iceland (Faroe Channel) (Fig. 31), at depths of 290–302.5 m (this study).

#### Remarks

From all the ‘stout-bodied’ typhlotanaids only members of two genera: the *Hansenotanais* and *Sarsotanais*, and two species *Gudmundotanais gudmundssoni* and *Stuttotanais carringtonae* n. sp. have a uropod with biarticulate endopod and uniarticulate exopod. Nevertheless only *Su. carringtonae* has a simple, rather than bifurcate pereopods 4–6 unguis as in all taxa mentioned above.

**Figure 48:**
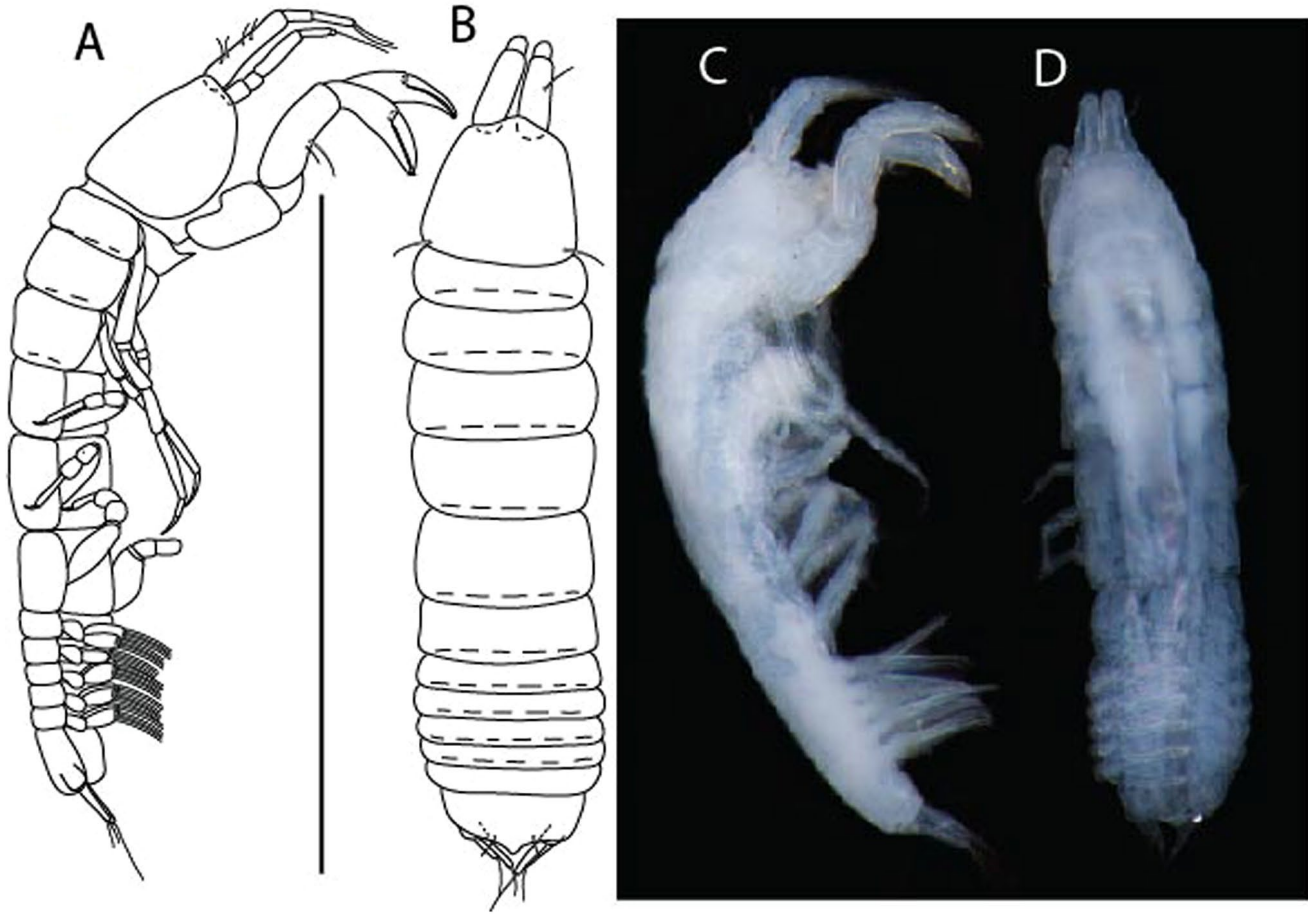
*Stuttotanais frenchae* n. sp., neuter (ICUL13952, ZMHK-64386), (**A**, **C**) Body, lateral view; (**B**, **D**) Body, dorsal view. Scale: A = 1 mm.

**Figure 49:**
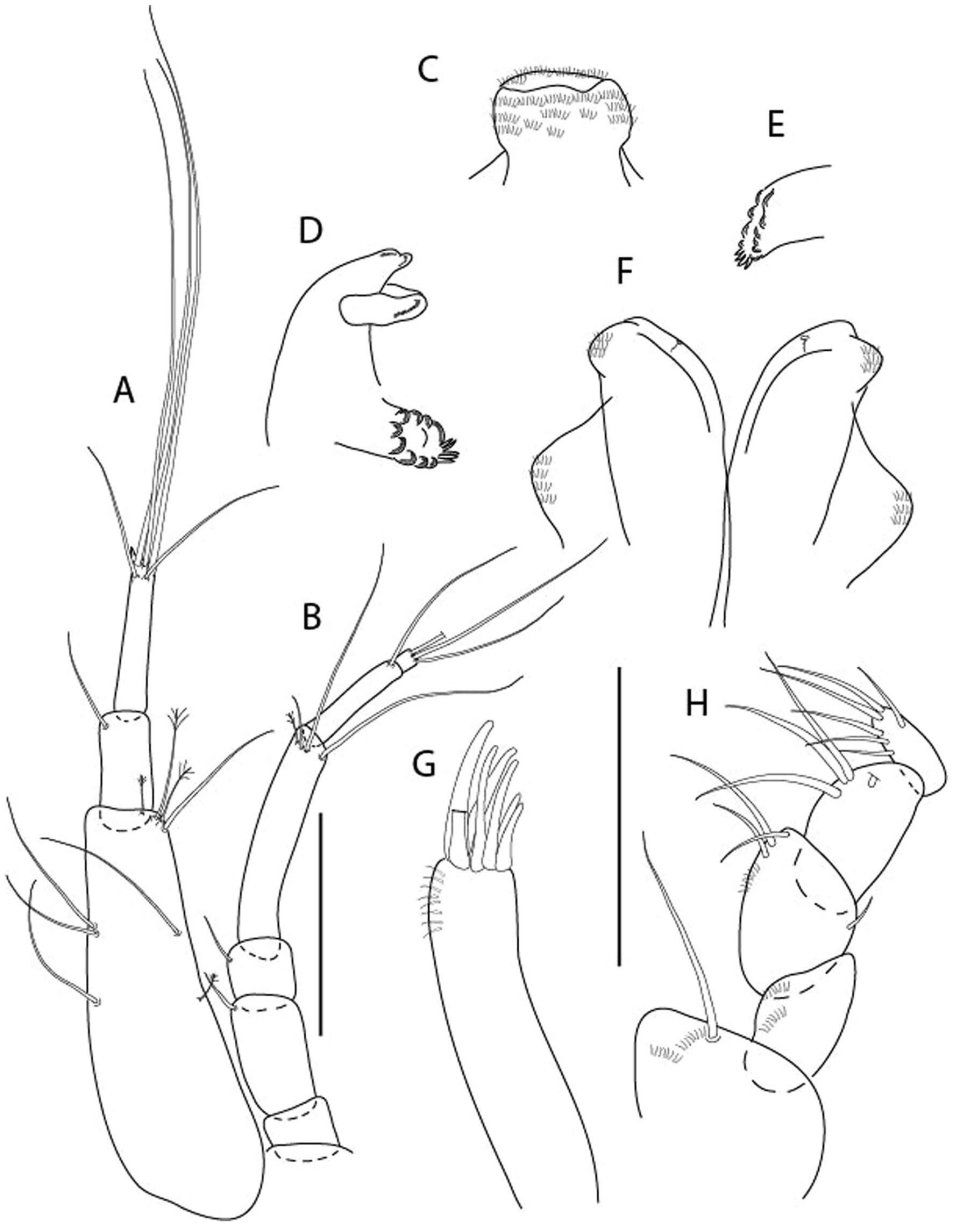
*Stuttotanais frenchae* n. sp., neuter (ICUL13959, ZMHK-64388), (**A**) Antennule; (**B**) Antenna; (**C**) Labrum; (**D**) Left mandible; (**E**) Right mandible; (**F**) Labium; (**G**) Maxillule; (**H**) Maxilliped. Scale: A–H = 0.1 mm.

**Figure 50:**
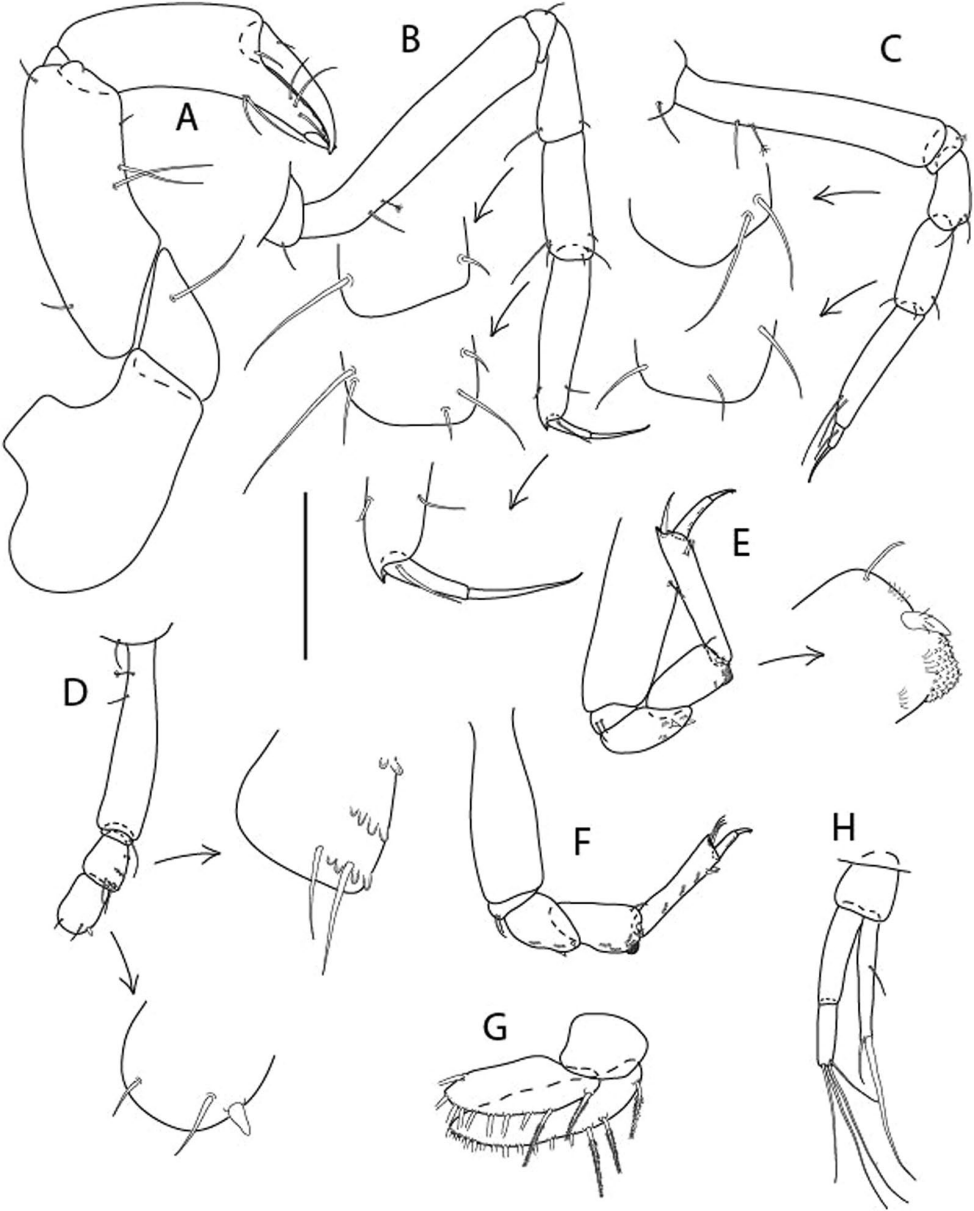
*Stuttotanais frenchae* n. sp., neuter (ICUL13959, ZMHK-64388), (**A**) Cheliped; (**B**) Pereopod-1; (**C**) Pereopod-2; (**D**) Pereopod-3; (**E**) Pereopod-4; (**F**) Pereopod-6; (**G**) Pleopod; (**H**) Uropod. Scale: A–H = 0.1 mm.

- ***Stuttotanais frenchae*** **Gellert & Błażewicz**
- LSID urn:lsid:zoobank.org:act:11B88AC0-B956-411E-84BA-26C75D79510C.
- (Figs. 48, 49 and 50).

#### Material examined

Holotype: neuter 1.1 mm, IceAGE II, St. 867-1 (ICUL13952, ZMHK-64386).

Paratypes: IceAGE II, St. 867-1, two neuters (ICUL8977, ZMHK-64387; broken, one ind. for extraction); IceAGE II, St. 867-1, neuter 1.1 mm (ICUL13959, ZMHK-64388; dissected); IceAGE II, St. 867-1, neuter 0.8 mm (ICUL9627, ZMHK-64389).

#### Diagnosis

Body stout, pereonite margins rounded. Antennule article-1 short (< 4.0 L:W), mesial margin with three setae. Maxilliped basis seta shorter than endites. Cheliped carpus long (> 2.5 L:W), carpus with short ventral seta. Pereopod-1 merus L:W short (< 3.0 L:W), carpus without long seta; pereopods 2–3 carpus ventrodistal seta short, microtrichia calcified; pereopods 4–6 carpus with prickly tubercles, propodus distodorsal seta short, unguis bifurcate. Uropod endopod biarticulate, exopod uniarticulate.

#### Etymology

This species is dedicated to Kate French – British Olympic champion in the modern pentathlon, women’s individual.

#### Description of neuter,

Length 1.0 mm. Body (Fig. 48A−D) stout, 4.3 L:W. Cephalothorax trapezoidal, 1.1 L:W, 2.6× pereonite-1, with short lateral seta. Pereonites 1−6: 0.3, 0.4, 0.5, 0.5, 0.6 and 0.4 L:W, respectively; all pereonites rounded. Pereonite-1 0.7× pereonite-2; pereonite-2 0.9× pereonite-3; pereonite-3 as long as pereonite-4; pereonite-4 0.5× pereonite-5; pereonite-5 1.4× pereonite-6. Pleon 0.2× BL; pleonites 1−5: of similar size – 0.2 L:W. Pleotelson 2.6× pereonite-6.

Antennule (Fig. 49A) 1.5× cephalothorax; article-1 3.9 L:W, with three setae on inner margin and seta and a PSS on outer margin, and seta and three PSS distally; article-2 2.4 L:W, 0.3× article-1, with dorsal simple seta on inner margin; article-3 5.6 L:W, 1.3× article-2, with five setae.

Antenna (Fig. 49B) article-2 1.9 L:W, with distal seta; article-3 1.2 L:W, 0.6× article-2, with seta; article-4 5.3 L:W, 3.1× article-3, with three setae (two longer than article-5) and a PSS distally; article-5 4.7 L:W, 0.5× article-4, with long seta; article-6 1.2 L:W, with three distal setae.

Mouthparts. Labrum (Fig. 49C) typical. Mandible (Fig. 49D−E) molar typical. Left mandible (Fig. 49D) incisor with two cusps, *lacinia mobilis* well developed, with single cusp; right mandible (Fig. 49E) incisor broken during dissection. Labium (Fig. 49F) typical, outer corner of inner lobe and outer lobe with minute setae. Maxillule (Fig. 49G) endite typical, two innermost spines shorter than the others, microtrichia along article. Maxilla lost during dissection.

Maxilliped (Fig. 49H) palp article-1 with numerous microtrichia; articles 2–4 typical. Epignath lost during dissection.

Cheliped (Fig. 50A) slender; basis 1.8 L:W, naked; merus (seta longer merus W); carpus 2.9 L:W, with two long setae (about carpus W) and short seta ventrally, dorsal margin setation typical; chela longer than carpus, 2.6 L:W, with seta in outer margin; palm 1.9× fixed finger; fixed finger cutting edge with three weak, distal blunt cusps; dactylus slightly curved, with seta.

Pereopod-1 (Fig. 50B) overall 15.4 L:W; coxa with seta, basis 5.7 L:W, with single dorsoproximal PSS and seta; merus 2.7 L:W, with dorsodistal and ventrodistal setae; carpus 2.7 L:W, 1.2× merus, with two dorsodistal and three ventrodistal setae (two short); propodus 4.5 L:W, 1.2× carpus, with dorsodistal (broken) and ventrodistal setae; dactylus 0.8× unguis, with seta, together 0.6× propodus.

Pereopod-2 (Fig. 50C) overall 15.3 L:W; coxa with seta; basis 6.4 L:W, with dorsoproximal a PSS and seta; merus 2.2 L:W, with two ventrodistal setae; carpus 2.4 L:W, 1.3× merus, with three distal setae; propodus 4.9 L:W, 0.7× merus and carpus combined, with two dorsodistal setae; dactylus 0.9× unguis, with seta, together 0.5× propodus.

Pereopod-3 (Fig. 50D) similar to pereopod-2 but basis 4.0 L:W; merus 1.5 L:W, with two ventrodistal setae and numerous calcified microtrichia; carpus 1.7 L:W, as long as merus, with dorsodistal and ventrodistal setae and spine; propodus, dactylus and unguis broken.

Pereopod-4 (Fig. 50E) overall 7.7 L:W; basis robust, 2.8 L:W, naked; merus 2.1 L:W, with two distal spines, and numerous microtrichia along article; carpus 2.1 L:W, 1.1× merus, with moderate prickly tubercles, dorsodistal chemosensory seta, and distal spine/crotchet; propodus 5.0 L:W, 1.7× carpus, with midventral PSS, two ventrodistal spines, and dorsodistal seta shorter than claw; dactylus 1.9× unguis, together 0.5× propodus; unguis bifurcate.

Pereopod-5 as pereopod-4.

Pereopod-6 (Fig. 50F) as pereopod-4 but propodus three dorsodistal setae as long as dactylus, and without midventral PSS.

Pleopod (Fig. 50G) exopod with ten plumose setae on outer margin; endopod with fifteen.

Uropod (Fig. 50H) endopod proximal article 1.6× distal article, naked; distal article with four long terminal setae; exopod 1.2× endopod proximal article, with seta at midlength, the other setation typical.

#### Distribution

Known from one station in the Faroe Channel, from depths of 290–302.5 m (Fig. 31) (this study).

#### Remarks

*Stuttotanais frenchae* n sp. shares a uniarticulate uropodal exopod and biarticulate endopod with two genera: *Hansenotanais* and *Sarsotanais*, but also with two species; *G. gudmundssoni* and *Su. carringtonae* (see below). The presence of typical prickly tubercles on the carpus of pereopods 4–6 separates *Su. frenchae* from *Hansenotanais* (has cusps in pereopod 4–6 carpus) and dorsodistal seta *G. gudmundssoni*, while a simple unguis in pereopod 4–6 from *Stuttotanais carringtonae* (bifurcate unguis in pereopod 4–6). Finally, *Su. frenchae* is a more slender species that has short meral setae on the pereopod-1, where the *Sarsotanais* has clearly robust body and has two long meral setae.

### Key for identification of neuters ‘stout-bodied’ forms*


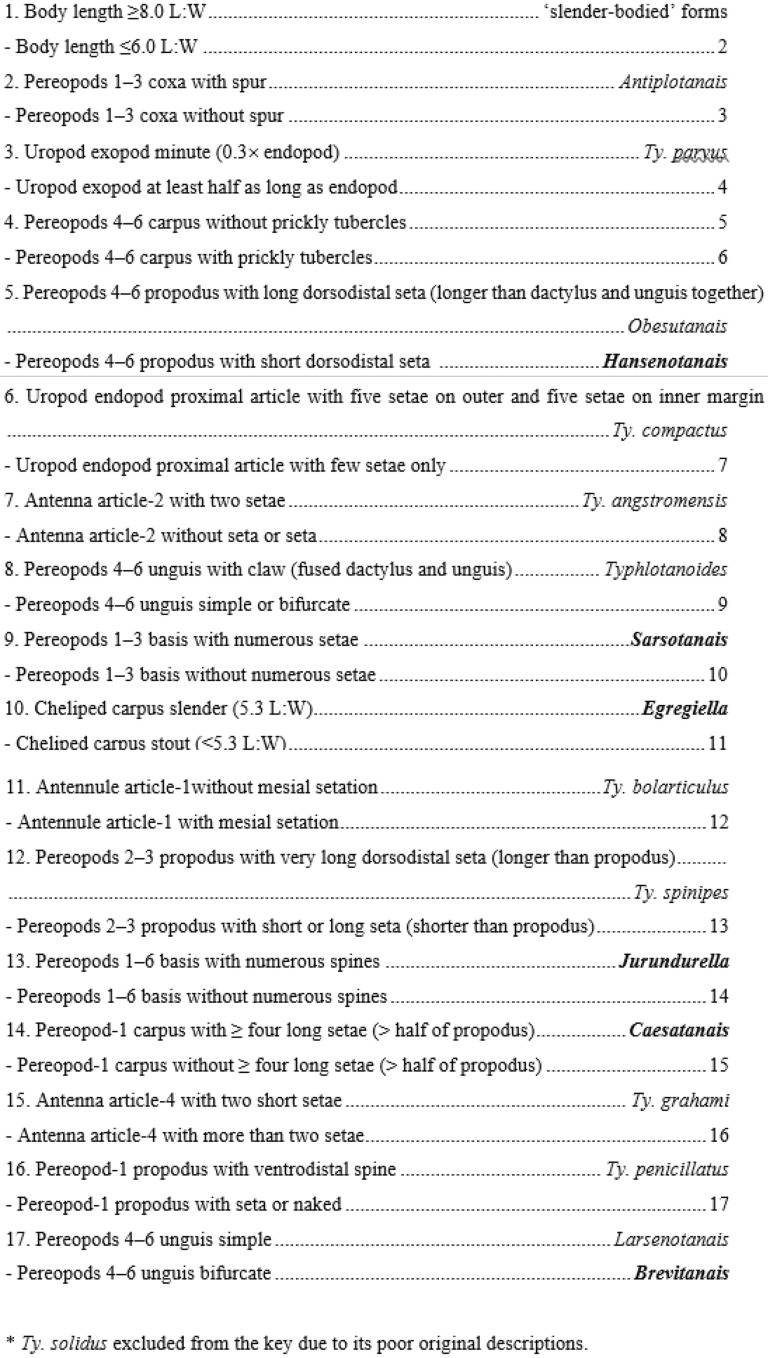


#### Distribution analysis

**Figure 51:**
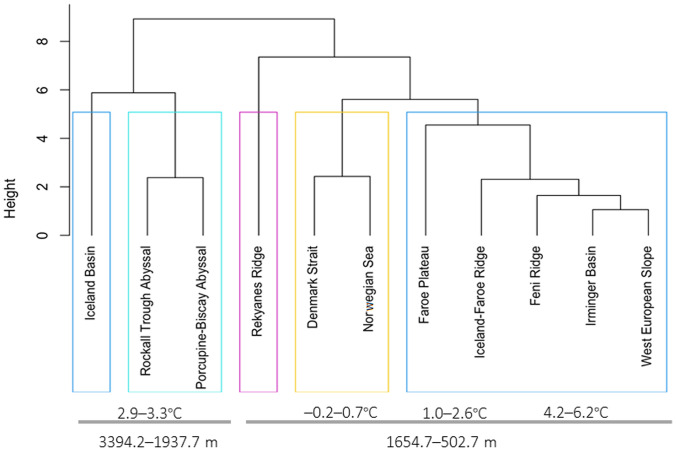
Dendrogram of predefined regions (Table 3) for typhlotanaid species (Canberra similarity, transformed data, complete method); the mean environmental parameters defining the regions are in Table 2. Abbreviations: DeS—Denmark Strait, Far—Faroe, FeR—Feni Ridge, GRE—Greenland-Iceland Rise, IcB—Iceland Basin, IrB—Irminger Basin, NoS—Norvegian Sea, PBA—Porcupine-Biscay Abyssal, RoT—Rockall, RR—Reykjanes Ridge, WES—West European Slope.

Cluster analysis applied to the distribution data yielded two distinct groups of regions (Fig. 51, Table 3). The samples from the Iceland Basin (IcB), Porcupine-Biscay Abyssal (PBA) and Rockall Trough (RoT) with extensive mean depths (1837–3394 m) are grouped, characterized by three species: *C. igae*, *C. isiae* and *G. gudmunsdssoni*. The other large clade includes shallower stations (503–1655 m) and consists of two subclusters: (1) Denmark Strait (DeS) and Norwegian Sea (NoS), both defined by low mean water temperature (from − 0.2 to 0.7 °C), and (2) the clade grouping the Faroe Plateau (FaP), Iceland-Faroe Ridge (IFR), Feni Ridge (FeR), Irminger Basin (IrB) and W European Slope (WES). FaP and IFR are characterized by low mean temperature (1.0–1.6 °C) and FeR, IrB and WES by much warmer mean temperatures (4.2–6.4 °C). *Brevitanais kozakowskae* and *Br. skolimowskae* are the key species in the first subclade. In the other subclade, no key species could be discerned, although the most distinct Faroe Plateau was represented by five species, three of which, *Hansenotanais hansjacobi*, *Stuttotanais frenchae* and *St. carringtonae* appear endemic. The Reykjanes Ridge (RR) was the most distinct region with the characteristic species *Brevitanais anitae*, *Br. sadleckae and Larsenotanais martini*. That region is defined by relatively shallow depths (730 m) and warm waters (5.8 °C).

**Table 3:** Number of samples with ‘stout-bodied’ typhlotanaids recorded from the studied collection in eleven distinguished regions.

Region/Species	*Br. anitae*	*Br. kozakowskae*	*Br. nagaye*	*Br. sadleckae*	*Br. skolimowskae*	*C. igae*	*C. isae*	*G. gudmundssoni*	*H. hansjacobi*	*H. inermis*	*J. bioice*	*L. martini*	*St. carringtonae*	*Sa. georgi*	*St. frenchae*	Total
Denmark Strait (DeS)	–	3	–	–	2	–	–	2	–	5	–	–	–	1	–	13
Faroe Plateau (FaP)	–	–	–	–	–	–	–	–	1	16	1	–	1	–	1	20
Feni Ridge (FeR)	–	–	–	–	–	–	–	1	–	–	3	–	–	–	–	4
Irminger Basin (IrB)	–	–	–	–	–	–	–	–	–	–	4	–	–	2	–	6
Norvegian Sea (NoS)	–	1	–	–	1	–	–	–	–	22	–	–	–	–	–	24
Rekyanes Ridge (RR)	1	–	–	2	–	–	–	–	–	–	3	1	–	1	–	7
Rockall Trough Abyssal (RoT)	–	–	–	–	–	20	–	30	–	–	5	–	–	26	–	81
W European Slope (WES)	–	–	–	–	–	–	–	–	–	–	9	–	–	4	–	13
Iceland-Faroe Ridge (IFR)	–	–	–	–	–	–	–	–	–	4	2	–	–	–	–	6
Iceland Basin (IcB)	–	–	2	–	–	4	2	3	–	4	4	–	–	13	–	32
Porcupine-Biscay Abyssal (PBA)	–	–	–	–	–	26	–	6	–	–	–	–	–	15	–	47
Total	1	4	2	2	3	50	2	42	1	51	31	1	1	62	1	253

**Figure 52:**
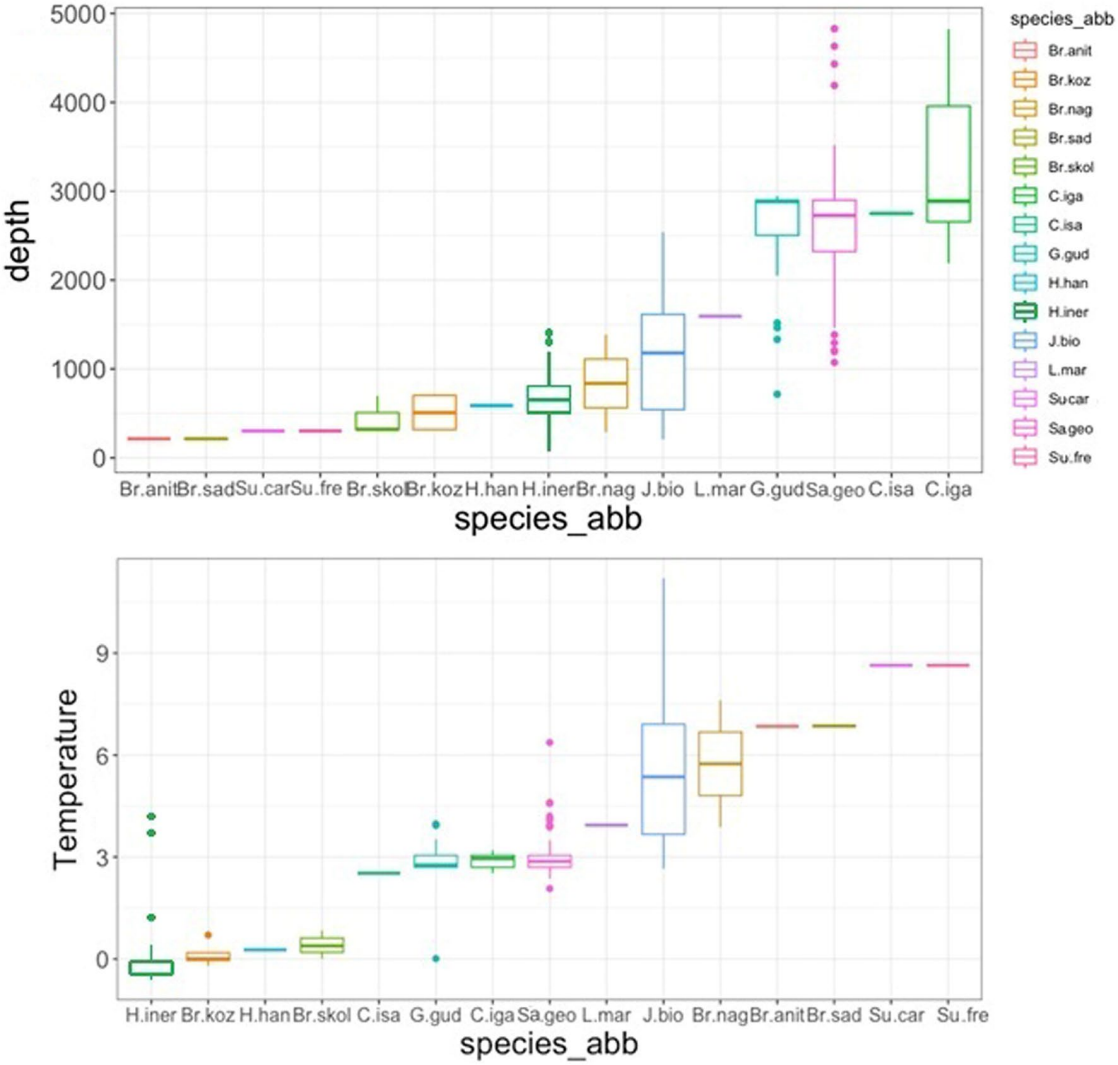
Depth and temperature distribution of analysed typhlotanaid species in the study area. Abbreviations: *Brevitanais skolimowskae*—Br. skol; *Br. anitae*—Br.anit; *Br. nagayae*—Br.nag; *Br. sadleckae*—Br.sad; *Br. kozakowskae*—Br.koz; *H. hansjacobi*—H.han; *H. inermis*—H.iner; *H. partykae*—H.par; *Gudmundotanais gudmundssoni*—G.gud; *Caesatanais isiae*—C. isa; *C. igae*—C.iga; *Sarsotanais georgi*—Sa.geo; *Jurundurella bioice*—J.bio; *Stuttotanais frenchae*—Su.fre; *Su. carringtonae*—Su.car.

The distribution of taxa in gradient of the depth and temperature (Fig. 52, Table S3) reveals a clear ‘preference’ of *Brevitanais* for shallow water, i.e. between 209 and 716 m (with one exception of *Br. nagayae* that was recorded at 1384.8 m), although the species are well separated by temperature: below < 0 °C, *Br. kozakowskae* and *Br. skolimowskae*, and 3.8–7.6 °C by *Br. nagayae, Br. sadleckae,* and *Br. anitae*. A distinctly low temperature affinity is observed for *Hansenotanais* species, which are present in the upper bathyal north of Iceland (Figs. 1, 52). The distribution of *H. inermis,* as *J. bioice,* is clearly limited to the upper bathyal (209– ~ 2000 m) of southern Iceland and significantly warmer waters (3.5–7.7 °C), but the temperature ranges for *J. bioice* has been recorded in W European Slope at 7.9–11.1 °C and Rockall Trough (~ 2.7 °C). *Gudmundotanais gudmundssoni* is a taxon of deeper bathyal regions (1332.5–2946 m; one station at 715 m) and present in a relatively narrow water temperatures range (4.0–2.7 °C). A similar distribution is shown by *Caesatanais igae* (2.5–3.2 °C) and *Sarsotanais georgi* (4.6–6.4 °C), although their bathymetric ranges extended from lower bathyal to abyssal e.g., 2191–4832 m and 1974–4829 m, respectively.

## Discussion

Incorporation of a variety of scientific methods for species delimitation, biodiversity and zoogeographical studies is extremely useful in the study of deep-sea fauna represented by a large number of rare and unknown species^12,13,24,69–72^. In our research genetic and morphological approaches were combined to examine a rich and diverse collection of historical and more recent deep-sea tanaidaceans collected in the N Atlantic. Despite these efforts, we were not able to obtain sequences from both markers for all targeted individuals and, as a result, could not construct a concatenated tree. Nevertheless, even with our limited results and relying on a conservative molecular marker such as 18S, it was possible to assess the diversity of 'stout bodied' typhlotanaids. In order to define a new genus and species we used integrative approach confronting 18S phylogeny and morphological diversity. Because 18S is very conservative marker, we assumed that each clade represented a taxonomical unit that we understand as a genus. The integrative approach was used for the first time for investigation of Tanaidacea and offered a solution through which deficiencies in our genetic or morphological data can be mutually supplemented and confirmed.

In our research we focused on ‘stout-bodied’ typhlotanaids recovered in 185 samples of over 1465 benthic samples from the N Atlantic over the last five decades (Table S1), supporting the view that the deep-sea fauna of the N Atlantic is relatively well-recognized. By discovering 15 new typhlotanaids species to science and establishing eight new genera and talking into account that our material represents only a small portion form a much larger collection of tanaids, we conclude that knowledge on the diversity of tanaidaceans in the N Atlantic is still far from being completed. In addition, the result of our study elevates the total number of known typhlotanaids species by 13%, and total number typhlotanaids genera by 35% compared to already published taxa (WoRMS accessed 6 April. 2023).

Before our research, three ‘stout-bodied’ typhlotanaids were recorded from the N Atlantic (*Typhlotanais cornutus*, *Typhlotanais eximius* and *Typhlotanais inermis*) and none of them was recovered by us. Eight taxa targeted by us taxa were delineated with a conservative DNA fragment (18S) and calculation of intra- and infra-species genetic variability allowed us to infer reliable species delimitation (Table S2). Except for a pair of closely related species (*Stuttotanais frenchae* and *Su. carringtonae*), we could not obtain COI sequences for all putative species, however calculated genetic distances prompted us to seek for morphological differences segregating both species and further morphological search to delineated morphologically similar species (Table 2).

### Morphology

The morphological characteristics that define the clades distinguished using the 18S marker contribute to defining the diagnoses for the genera we have distinguished, but also provide a better understanding of the features (or “characters”), many of which have been previously either underestimated or not even perceived^28,67,73^. Among the important and easy to observe diagnostic features is the articulation and character of the uropods (length relative to the body, and proportions of individual members). Direct observation of deep-sea tanaids is virtually impossible, but it is assumed that the uropod exposed at the posterior opening of the tube may acquire the environmental cues in posterior part of the tanaid analogous to the antennae exposed to its anterior part^60,74^, thus could be a subject to evolution by natural selection. Usually, all stout typhlotanaids have rather short uropods in relation to the body, in which the exopod usually reaches 0.8–0.9 of endopod length. The shortest exopod is in the genus *Hansenotanais* (0.6 × endopod), while the most elongate uropods are observed in *Egregiella*, where both uropod rami are almost equal.

It should be noted that, with some exceptions, none of the taxa we have distinguished can be diagnosed on the basis of a single apomorphy, but rather by a unique combination of several features (Table 2). For example, the setation of the pereopods and character of clinging apparatus in pereopods 4–6 was proved to be a diagnostic character ^27,28^. The modern imaging techniques^50^ applied in our research allowed us to demonstrate that the presence of prickly tubercles is not invariant in the typhlotanaids. Although many of the ‘stout-bodied’ typhlotanaids have them well developed, they are absent in genus *Hansenotanai*s (Fig. 35D) (also absent in *Typhlamia*). A bifurcate unguis in the pereopods 4–6 is present in several genera, *Brevitanais, Hansenotanais, Caesatanais, Jurundurella*, *Sarsotanais* and *Stuttotanais*, but only *Brevitanais* and *Caesatanais* have a long propodus dorsodistal seta in the pereopods 4–6. Furthermore, calcified microtrichia in pereopods 2 and 3 are unique for *Stuttotanais* and *Sarsotanais* (Figs. 44, 47, 50), although *Sarsotanais* has them in pereopod-2 merus and carpus, while *Stuttotanais* only on the pereopod-2 merus. Finally, *Jurundurella* has distinct spines on the basis of the pereopods 2–5 (Fig. 40) which are unique feature. Similar spines however are observed also in two other species: *Typhlotanais spinibasis* (although spines on basis of pereopods 4–6) and *Typhlotanais spinipes* (spines present in pereopods 2–5). However, *Ty. spinibasis* is a slender-bodied typhlotanaid (8.5 L:W) and because of long setae in merus, carpus and propodus in pereopods 1–3 was classified to ‘ptrispinosus’ group (Błażewicz-Paszkowycz, 2007), while *Ty. spinipes* has biarticulate uropod rami (unriarticulate in *Jurundurella*), long and slender antennule (short in *Jurundurella*), and ventrodistal seta in propodus 2–3 (spine in *Jurundurella*)^59,75^. Intriguingly, *Ty spinipes*, as *Jurundurella*, has long distal setae in pereopods 2–3 (Fig. 40), thus further study with more material and possible genetic data are needed to test if those taxa are congeneric or at least are phylogenetically closely related taxa.

### Phylogeny

The Typhlotanaidae is recognized to be a highly diverse tanaidomorphan family whose persived polyphyletic nature has been frequently underlined^27,28,39,76,77^. Our phylogenetic results, which were based on two markers, should be considered only as a primary step towards complex phylogenetic studies of 'stout-bodied' forms of this family. Nevertheless, the examination of reference barcodes 18S and H3 is given for five new genera (*Brevitanais, Caesatanais, Gudmundotanais, Hansenotanais* and *Stuttotanais*) and these data are a baseline for future assignment of new (and difficult-to-identify) 'stout-bodied' typhlotanaids species. The low support of some clades in the 18S tree can be explained by the insufficient number of genera included in the analysis, and it is expected that a more complete analysis with more genera will improve the statistical parameters of the tree. The unrelated clades group together by with low support. *Baratheonus* groups with the ‘stout-bodied’ clade in the 18S tree and several ‘stout-bodied’ genera (*Caesatanais* and *Gudmundotanais*) group with the ‘slender-bodied’ forms in the morphological tree. The gene H3 has an erratic pattern of substitution and is not resolved. Nevertheless, it is quite apparent that the ‘stout-bodied’ forms do constitute a monophyletic group (*Brevitanais, Egregiella, Gudmundotanais, Jurundurella, Sarsotanais* and *Stuttotanais*). The *Hansenotanais* that grouped with *Typhlamia* share the cusps in the pereopods 4–6 where the other 'stout-bodied' typhlotanaids have prickly tubercles. Moreover, the morphological tree is congruent with the genetic tree and consists of two large clades with ‘stout-bodied’ forms and ‘slender-bodied’ typhlotanaids. The latter clade is supported by the pereopod-1 propodus with a long ventrodistal seta. The appearance of two ‘stout-bodied’ forms (*Caesatanais* and *Gudmundotanais*) in this group may be coincidental. The exception is the genus *Hansenotanais*, which in molecular trees (18S and H3) is always grouped with *Typhlamia*.

Morphological affinities are considered insufficient for testing evolutionary hypotheses because of interference from homoplasy and convergence^78^. The scarcity of genetic data in our research, which could serve for haplotype and private allele inquiry, hamper inferences concerning the radiation of taxa grouped in specific clades. Nevertheless, grouping of all Icelandic *Brevitanais* in one clade in the morphological tree, is certainly worth noting, especially that *Ty. cornutus* (Kval Island, N Norway) and *Ty. adipatus* (W Antarctic) group in a paraphyletic node. Although, the lack of genetic, biogeographical data does not provide a way to go much beyond conjecture, we can hypothesize that high diversity of Icelandic *Brevitanais* might be result of panmictic populations that genetically diverged after fragmentation from the Quaternary Last Glacial Maxima (LGM). The grounded ice cover that growing on the Arctic shelf forced fauna to migrate to ice-free locations obtainable at greater depths i.e. lower shelf or bathyal or southerly waters^22,79–82^. *Brevitanais* taxa that reveal clear preference for lower shelf depths seems to be naturally predestined to survive glacial maxima on the Icelandic slope. The bathyal glacial shelters are not particularly thoroughly explored in the Arctic, where the research focused on the shallow littoral fauna^80,83^, but the Antarctic fauna could only survive a glaciation period in deeper glacial refuges due to its long and profound topographic isolation^24,84–86^.

### Diversity

The typhlotanaids are seen to be a diverse family of deep-sea communities with contribution of 11–25% to all tanaidaceans recovered from the NE Pacific, W Australian Slope or E Antarctic^55,87–90^. Regardless of the high diversity, the typhlotanaids were often recorded as unique taxa, represented by few individuals^91,92^. The low frequency and abundance of small deep-sea fauna, as tanaidaceans, are constricted by trophic and physio-chemical conditions of the ecosystem^93,94^, is more often low a consequence of sampling efforts resulted from high cost and logistical challenges of deep-sea surveys^9,95^. In our research, we analysed a well sampled area of the N Atlantic (Table 3). With four of eleven large regions particularly so (W European Seas, Rockall Trough, Iceland Basin, and Norwegian Sea) with 75% of the collected samples. Much less sampled, but also smaller, regions, were the Reykjanes and Feni Ridge (16 and 4 samples, respectively). Regardless of the sampling efforts, ‘stout-bodied’ typhlotanaids were present in 10.6–33.6% of samples, with the exception of best sampled WES region where the ‘stout-bodied’ typhlotanaids occurred in only 2.2% of all samples. With current knowledge, it is difficult to conclude why ‘stout-bodied’ typhlotanaids are relatively lacking in the WES region. Because average temperatures (6.1 ± 1.6 °C) and mean depth (1274.3 ± 489.3 m) of WES are similar to those in Irminger Basin where ‘stout-bodied’ typhlotanaids were recovered in 13% samples, it is rather doubtful that such distribution is the effect of environmental selection. Certainly, this result deserves further and more detailed research, including modelling based on extensive data covering the other groups of Typhlotanaidae, the other families of Tanaidacea, but also other groups of Peracarida.

### Distribution

In the distribution of the six species with a preference for greater depths, a clear split is drawn between the fauna of the north and south (Figs. 5, 9 and 31). The abundant and frequent species i.e. *C. igae*, *G. gudmundssoni*, *Sa. georgi, J. bioice*. and *L. martini*, are present south of Iceland, and only *H. inermis* was located in northern sites. The seas surrounding Iceland are separated by the well-defined latitudinally-oriented topographic structure Greenland-Iceland-Faroe (GIF) with the deepest sill depth at 840 m between the Faeroe Islands and Scotland^22,96^. This ridge is known as a prominent topographic barrier in the N Atlantic that hampers distribution of water masses between the Arctic and the deep basins of the Nordic seas. The complex system of ocean currents and oceanic ridges around Iceland separates neighbouring areas, where temperature, salinity, availability of organic matter and oxygen can significantly vary^12,22,97–100^, and thus shape habitat mosaic affects the species compositions of benthic invertebrates^13,23,101–103^. Moreover, the GIF is known to hamper geneflow and hybridization effect for genetic population structure of isopods^104^. As a consequence, this distinct topographic structure becomes a distinct zoogeographical barrier shaping a region extremely sensitive to climate change^12,13,23,105^.

The low dispersal mobility and high taxonomic diversity of the tanaidacean family Typhlotanaidae make it an excellent model in biodiversity studies and an ideal indicator in assessing the effects of anthropogenic activities. The climate change in oceanic zones will cause numerous shifts in hydrological conditions, although the scale is difficult to assess due to the marginal understanding of the biodiversity of deep-sea ecosystems. The character and the scale of disturbances caused in the ecosystems by anthropogenic activity is unpredictable, but it is assessed that the changes will be irreversible and will last for decades. Knowledge of biodiversity by integrative approaches along with the geographic distribution of newly discovered species and their environmental preferences can help protect exposed warming sites in the marine ecosystem. We are strongly convinced that the data presented here is an important piece of knowledge in the study of climate change affecting the N Atlantic.

## Materials and methods

### Sampling

The 4043 typhlotanaids specimens for the research were collected during 18 expeditions, with the distribution of the stations given in Table S1. Apart from the samples taken during the IceAGE 1 and 2 expeditions, all samples were fixed (and possibly stored) with formalin^106,107^. Distribution maps were prepared using the QGIS 3.28 software^108^. The environmental variables were collected from the cruise reports^106^, publications^109,110^ and complemented from World Ocean Atlas 2020 (https://www.ncei.noaa.gov/products/world-ocean-atlas). The variables for the defined region have been averaged. Correlation matrix of environmental variables reveals high correlation between depth, nitrogen, phosphorus, oxygen and (r > 0.88, *p* < 0.02) thus for the species distribution only temperature and depth were considered (Fig. S2).

The type-material and the other materials studied for this research are deposited in the Icelandic Museum of Natural History (Reykjavik), Kaldbak Marine Biological Laboratory (Faroe Islands), Muséum national d’Histoire naturelle (MNHM, Paris), Museum der Natur (ZMHK, Hamburg, Germany), National Museum of Scotland (NMS, Edinburgh), Natural History Museum (NHM, London), and Smithsonian National Museum of Natural History (Washington DC).

### Morphological analyses

Initial species identification was based on morphological observations with a dissecting microscope. The typhlotanaid collections were sorted to several morpho-groups, and 1918 individuals were preliminarily identified as ‘stout-bodied’ forms and were assigned for further comprehensive morphological study. From each group, several individuals were designated for thorough morphological analysis and dissected with chemically sharpened tungsten needles. The dissected cephalothorax, pereon, and pleon appendages were mounted on slides using glycerine and sealed with molten paraffin^111^. Morphological drawings were prepared using a light microscope (Nikon Eclipse 50i) equipped with a camera lucida. Digital pictures were completed using a graphic tablet following^112^.

Total body length (BL) was measured along the central axis of symmetry, from the rostrum to the tip of the pleotelson. In contrast, body width was assessed perpendicular to the symmetry axis at the widest point (BW). Body width and length of cephalothorax, pereonites, pleonites, and pleotelson were measured on whole specimens. All measurements were made with ImageJ^113^.

### Terminology and species description

Morphological terminology is largely as in Błażewicz-Paszkowycz 2007^27^. The seta types are recognized as: simple setae- without ornamentation, serrate- with serration or denticulation, penicillate- with a tuft of setules located distally and with a small knob on which seta is fixed to the tegument and chemosensory setae- slightly inflated distally and with a pore followed^73^, but is abbreviated here to PSS (penicillate/pinnate sensory seta) as used by Bird (2019)^114^, *inter alia*. Stout setae (L:W < 5.0) are called spines (= spiniform setae). Unspecified setae in the taxonomic descriptions are termed simple setae by default. The clinging apparatus (= prickly tubercles) is a system of various hooks, tubercles, thorns, and spines located on the carpus of pereopods 4−6^27,28,77^. The short ventral seta situated besides two long setae on the cheliped carpus is called ‘third’ seta. Błażewicz-Paszkowycz (2007)^27^ proposed a classification of typhlotanaids into ‘long-bodied’ (body ≥ 8.0 L:W) and ‘short-bodied’ (body < 6.0 L:W) taxa but here are replaced by ‘slender-bodied’ ‘stout-bodied’ respectively (see the Key for Typhlotanaidae genera and morpho-groups in Błażewicz-Paszkowycz, 2007^27^). The neuter is the post-manca stage, which cannot be classified as male or female. Two-letter genus abbreviations are used throughout the text to distinguish between genera: *Tq.*—*Torquella, Tm.*—*Typhlamia, Ty.*—*Typhlotanais*^28^, *Ba.*—*Baratheonus*, *Br.*—*Brevitanais*, *St.*—*Starkus*, *Sa.*—*Sarsotanais*, and *Su*.—*Stuttotanais*.

### DNA analyses

Given that most specimens were conserved in formaldehyde, a final set of 40 individuals were used for molecular study. Extraction of DNA from morphologically identified neuters was performed using the Chelex (InstaGene Matrix, Bio-Rad) method as in Jakiel et al*.* 2020^73^ and kit Sherlock AX (A&A Biotechnology) for manual isolation of genomic DNA are based on the mechanism of nucleic acid adsorption on ion exchange beads combined with the isopropanol precipitation of DNA. Vouchers specimens were recovered after extraction. The nuclear 18S rRNA region and histone H3 were PCR amplified using the primer pairs 18S 4F/2R (5′–CCA AGG AAG RCA GCA GGC ACG–3′ and 5′–GAG TCC CGT GTT GAG TCA ATT AAG C–3′)^115^, H3AF/ H3AR (5′–ATG GCT CGT ACC AAG CAG ACV GC–3′ and 5′–ATA TCC TTR GGC ATR ATR GTG AC–3′)^116^. The reaction protocol for 18S rRNA consisted of an initial denaturation at 95 °C for 2 min., 35× reaction cycles of 95 °C for 1 min., 53.6 °C for 45 s, 72 °C for 3 min., and a final extension at 72 °C for 10 min, and for histone H3 at 95 °C for 3 min., 35× reaction cycles of 95 °C for 30 s, 47 °C for 30 s, 72 °C for 1 min., and a final extension at 72 °C for 15 min. and for 18S rRNA at 95 °C for 2 min., 35 reaction cycles of 95 °C for 1 min., 53.6 °C for 45 s, 72 °C for 3 min., and a final extension at 72 °C for 10 min. After Sanger sequencing (Macrogen, Netherlands), alignment were built using Geneious aligner v9.1.3 (www.geneious.com) and compared with the GenBank database using BLAST^117^ to discard contamination from non-arthropod sources. Our newly obtained sequences and those obtained from GenBank were aligned using MAFFT and the L-INSi alignment option^118^. The 18S sequence alignment was trimmed to 964 bp and H3 to 340 bp. Phylogenetic tree was reconstructed using Maximum Likelihood and Bayesian inference. Nucleotide substitution model selection was performed according to the BIC 18S: (HKY + I + G4, H3: K2 + I + G4) The Hasegawa-Kishino-Yano (HKY + I + G4) showed the lowest BIC for 18S and the Kimmura 2-parameter model (K2 + G + I) for histone H3. The Bayesian phylogeny was reconstructed using MrBayes 3.2.7^120,121^. Four runs of Markov chain Monte Carlo (MCMC) were performed each 10 million generations-long, sampled every 1000 generations. All runs converge with standard deviation of split frequencies below 0.0 and potential scale reduction factor reaching 1. The trees were summarised using sumt command in MrBayes with 25% “burn-in” phase removed^122,123^. The Maximum Likelihood phylogenetic tree was made in RAxML Version 8^119,124^. Nodal support was assessed using 1000 bootstrap replicates. Patristic distances (i.e. sum of the length of all branches connecting two lineages in an evolutionary tree) were estimated using Mega version X.

### Morphological phylogeny analyses

To investigate the phylogenetic relationships among ‘stout-bodied’ form of Typhlotanaidae, a phylogenetic analysis was performed. The matrix with 39 morphological characters and 25 species including 14 newly described species and was compiled. 24 species were classified as an ingroup. The non-typhlotanaid *Akanthophoreus* sp. was selected as the outgroup. The classification of Tanaidacea is based primarily on the morphology of the females, and for most species and genera the males are not known at all. Therefore, the characters used in the matrix are based on descriptions of females or neuters. Ten of the 39 characters were continuous and 25 were numerical. Numerical characters included 16 binary and 13 multistate features. Characters were unordered and unweighted. The order of the characters in the matrix corresponds approximately to the order of the appendages, counting from the beginning of the cephalothorax to the end of the pleotelson. The data matrix was prepared in Excel and was converted for use in TNT^125^. A heuristic search was conducted in TNT v 1.5^125–127^ using a traditional search algorithm^125^ (suitable for a small database) with 1000 replications and 100 trees in each replication. A tree bisection and reconnection (TBR) branch swapping algorithm was used. Consistency Index, one measure of homoplasy; it is computed as the minimum number of changes divided by the number of changes requested in the tree and ranges from 0 to 1 (CI = 1 means no homoplasy). Retention Index (RI) shows how the tree fits the character and how well the synapomorphies explain the tree; it is computed as (Max steps–Observed steps)/(max steps–min steps); RI ranges from 0 to 1, if RI = 1, the character fits ideally^128^. Branch support was computed using Bremer's Relative Support—subtrees up to 10 steps; relative fit difference of 0.9 realized in TNT^129^.

### Morphological characters

#### Continuous

1. Cephalothorax (L:W).
2. Antennule article-1 (L:W).
3. Antenna article-2 (L:W).
4. Cheliped carpus (L:W).
5. Pereopod-1 basis/ pereopod-1 other articles.
6. Pereopod-1 merus (L:W).
7. Pereopod-2 merus (L:W).
8. Pereopod-2 carpus (L:W).
9. Pereopod-3 carpus (L:W).
10. Uropod exopod/ endopod.

#### Discrete

11. Body: 1—short and thin; 2—long.
12. Pereonites margin: 1—rounded; 2—straight.
13. Antennule article-1 mesial setation: 1—naked; 2—one seta; 3—two setae; 4—three setae; 5—4 ≤ setae.
14. Antennule apical spur: 1—absent; 2—present.
15. Antennule article-1 aesthetasc: 1—absent; 2—present.
16. Antenna articles-2 ornamentation: 1—naked; 2—one seta; 3—two setae.
17. Antenna articles-2 type of seta: 1—naked; 2—short; 3—long; 4—robust.
18. Antenna articles-3 ornamentation: 1—naked; 2—one seta.
19. Antenna articles-3 type of seta: 1—naked; 2—short; 3—long; 4—robust.
20. Maxilliped basis seta: 1—shorter than endites; 2—longer than endites.
21. Maxilliped cusps: 1—small; 2—medium; 3—large.
22. Pereopods 1–3 coxa with spur: 1—absent; 2—present.
23. Pereopods 1–3 merus with large spine: 1—absent; 2—present.
24. Pereopod-1 carpus with long setae: 1—absent; 2—two dorsodistal; 3—at least three.
25. Pereopod-1 propodus ventrodistal seta: 1—naked; 2—short; 3—long.
26. Pereopod-1 propodus type of ventrodistal seta: 1—naked; 2—simple; 3—bifurcate; 4—spine.
27. Pereopods 2–3 merus calcified microtrichia: 1—absent; 2—present.
28. Pereopods 2–3 carpus ventrodistal seta: 1—short; 2—long.
29. Pereopods 2–3 propodus dorsodistal with: 1—naked; 2—intermediate; 3—two intermediate; 4—long and short setae.
30. Pereopods 2–3 propodus with long dorsodistal seta (longer than dactylus): 1—short; 2—intermediate; 3—long.
31. Pereopods 2–3 propodus ventrodistal margin: 1—naked; 2—seta; 3—spine.
32. Pereopods 2–3 propodus ventrodistal margin size of seta/spine: 1—naked; 2—short; 3—long.
33. Pereopods 4–6 ischium with: 1—one seta; 2—two setae.
34. Pereopods 4–6 carpus with clinging apparatus (prickly tubercles): 1—present; 2—absent; 3—cusps (as in *Typhlamia,* see Gellert et al*.* 2022^28^).
35. Pereopods 4–6 propodus distodorsal seta: 1—short; 2—long.
36. Pereopods 4–6 unguis: 1—simple; 2—bifurcate.
37. Pleopods: 1—rudimentary; 2—normal shape.
38. Uropod endopod: 1—one-; 2—biarticulate.
39. Uropod exopod: 1—one-; 2—biarticulate.

### Imaging

Confocal laser scanning microscopy (CLSM) images were taken with an LSM 780 (Zeiss) microscope equipped with a Plan-Apochromat 63×/1.4 objective using the InTune tuneable excitation laser system (set to excitation wavelength 555 nm). Specimens were stained for 24 h with an equal volume mixture of saturated water solutions of Congo red and acid fuchsin. Before dissection and mounting in 100% glycerol, stained animals were washed thoroughly with 50% aqueous glycerol solution. Fluorescence was registered in single emission channel: 561–695 nm. Images were recorded as Z-stacks with 12.6 ms pixel dwell and two times line averaging with optical cross section of 0.5 mm. Collected data were pseudo-coloured in gold and reconstructed into a 3D image stack by maximum intensity projection using ZEN software (Zeiss). The microscope is situated in the Department of Molecular Biophysics, University of Lodz, Poland. Scanning electron microscopy (SEM) was used to illustrate tiny cuticular structures such as setae. SEM imaging was performed using a Phenom Pro X Scanning Electron Microscope at the Department of Invertebrate Zoology and Hydrobiology, University of Lodz, Poland from air-dried without sputter metallic layer^28^. The body habitus was photographed with a Leica MDG41and the LAS X program.

## Supplementary Information

Supplementary Information.

## Data Availability

The datasets presented in this study can be found in online repositories—GenBank https://www.ncbi.nlm.nih.gov/genbank/ or BOLD https://www.boldsystems.org/ (GenBank accession numbers: OQ107187–OQ107212 for H3 and OQ034236–OQ034255 for 18S rDNA; see Table 1).
