# Supplementary material for: Diversity under a magnifier lens: the case of Typhlotanaidae (Crustacea: Tanaidacea) in the N Atlantic
Source: Sci Rep. 2023 Jul 5;13:10905. doi: 10.1038/s41598-023-33616-y (PMC10322912; doi:10.1038/s41598-023-33616-y)

SUPPLEMENTARY MATERIAL

**Diversity under a magnifier lens: The case of Typhlotanaidae (Crustacea: Tanaidacea) in the N Atlantic**

Marta Gellert, Magdalena Błażewicz, Tomasz Mamos & Graham J. Bird

**Table S1.** Stations where ‘stout-bodied’ form of Typhlotanaidae were recorded.

| **New region** | **Survey_station** | **Latitude** | **Longitude** | **depth** |
| --- | --- | --- | --- | --- |
| **Denmark Strait (DeS)** | BIOICE_2912 | 65,10845 | -29,04085 | 1463,5 |
|  | IceAGE_1119-1 | 67,2135 | -26,24166 | 696,9 |
|  | IceAGE_1123-1 | 67,21383 | -26,2075 | 716,5 |
|  | IceAGE_1129-1 | 67,64616 | -29,74633 | 320,6 |
|  | IceAGE_1130-1 | 67,646 | -26,74666 | 318,9 |
|  | BIOICE_2317 | 67,96 | -24,72 | 996 |
|  | BIOICE_2570 | 67,051 | -23,986 | 370 |
|  | BIOICE_2591 | 67,208 | -22,424 | 333 |
|  | BIOICE_2741 | 67,6483 | -20,238 | 510 |
|  | BIOICE_2744 | 67,7493 | -20,4737 | 731 |
| **Faroe Plateau (FaP)** | BIOFAR_27 | 61,902 | -5,064 | 225 |
|  | IceAGE II_867-1 | 61,99716 | 0,50666 | 302,5 |
|  | IceAGE II_868-3 | 62,15233 | 0,2585 | 587,4 |
|  | AFEN 1996_53803#1 | 61,474 | -1,686 | 649 |
|  | AFEN 1996_53878#2 | 60,239 | -4,764 | 689 |
|  | AFEN 1996_53896#(1)/2 | 60,731 | -3,584 | 640 |
|  | AFEN 1996_53899#1 | 60,993 | -2,861 | 635 |
|  | AFEN 1996_53915#1 | 61,154 | -2,326 | 642 |
|  | BIOFAR_15 | 62,628 | -4,673 | 683 |
|  | BIOFAR_168 | 62,693 | -3,62 | 899 |
|  | BIOFAR_169 | 61,033 | -5,233 | 509 |
|  | BIOFAR_170 | 62,532 | -3,518 | 699 |
|  | BIOFAR_171 | 62,424 | -3,527 | 601 |
|  | BIOFAR_477 | 60,945 | -4,306 | 1150 |
|  | DTI 2000_55273#1 | 60,1925 | -5,0433 | 793 |
|  | DTI 2000_55300#1 | 61,6135 | -1,9817 | 1306 |
|  | DTI 2000_55404#1,2 | 61,077 | -2,6093 | 650 |
|  | SMBA_ES87 | 61,217 | -3,983 | 1050 |
| **Feni Ridge (FR)** | SMBA_ES112 | 55,2 | -15,833 | 1900 |
|  | SMBA_ES252 | 58,867 | -12,883 | 1510 |
|  | SMBA_ES99 | 60 | -10,583 | 1160 |
|  | SMBA_SBC263 | 56,5 | -13,5 | 2049 |
| **Irminger Basin (IrB)** | BIOICE_2219 | 64,209 | -25,271 | 265 |
|  | BIOICE_2257 | 63,243 | -26,485 | 1209 |
|  | BIOICE_2704 | 63,8417 | -27,7133 | 1295 |
|  | BIOICE_2719 | 64,428 | -26,403 | 305 |
|  | BIOICE_2720 | 64,43 | -26,4033 | 304 |
|  | BIOICE_2873 | 64,627 | -27,2385 | 555 |
| **Norvegian Sea (NoS)** | IceAGE_1136-1 | 67,63583 | -17,7665 | 315,9 |
|  | IceAGE_1209-1 | 66,53816 | -12,86483 | 315,9 |
|  | BIOICE_2011 | 65,34805 | -11,1711 | 761,5 |
|  | BIOICE_2014 | 65,41225 | -10,8064 | 974 |
|  | BIOICE_2023 | 66,33085 | -13,15465 | 300 |
|  | BIOICE_2088 | 67,239 | -17,857 | 617 |
|  | BIOICE_2089 | 67,218 | -17,839 | 517 |
|  | BIOICE_2091 | 67,19 | -17,774 | 405 |
|  | BIOICE_2118 | 67,487 | -19,545 | 393 |
|  | BIOICE_2124 | 67,186 | -19,564 | 347 |
|  | BIOICE_2136 | 66,726 | -18,954 | 417 |
|  | BIOICE_2315 | 64,1 | -9,05 | 991 |
|  | BIOICE_2318 | 64,033 | -9,617 | 772 |
|  | BIOICE_2579 | 64,117 | -9,05 | 996 |
|  | BIOICE_2619 | 67,281 | -16,6295 | 603 |
|  | BIOICE_2629 | 67,3285 | -16,1183 | 603 |
|  | BIOICE_2644 | 68,0192 | -15,2445 | 1198 |
|  | BIOICE_2648 | 68,0848 | -15,3108 | 1306 |
|  | BIOICE_2660 | 67,2402 | -15,4737 | 271 |
|  | BIOICE_2779 | 68,34375 | -15,78075 | 1408 |
|  | BIOICE_2786 | 67,404915 | -18,24325 | 684 |
|  | BIOICE_3115 | 67,42115 | -19,47925 | 604 |
|  | BIOICE_3124 | 68,0937 | -17,5876 | 876,5 |
|  | BIOICE_3247 | 66,1032 | -12,0129 | 243 |
| **Rekyanes Ridge (RR)** | IceAGE_1043-1 | 63,92433 | -25,961 | 213,9 |
|  | IceAGE_1045-1 | 63,92833 | -25,9535 | 218,4 |
|  | IceAGE_1047-1 | 63,9345 | -25,94216 | 209,4 |
|  | IceAGE_1072-1 | 63,00766 | -28,06816 | 1593,8 |
| **Rockall Trough Abyssal (RoT)** | INCAL_CP04 | 56,533 | -11,2 | 2513 |
|  | INCAL_CP05 | 55 | -12,517 | 2884 |
|  | INCAL_CP06 | 55,033 | -12,683 | 2893 |
|  | INCAL_CP07 | 55,067 | -12,767 | 2897 |
|  | INCAL_DS05 | 56,45 | -11,2 | 2503 |
|  | INCAL_DS06 | 56,417 | -11,167 | 2494 |
|  | INCAL_DS07 | 55,017 | -12,533 | 2884 |
|  | INCAL_DS08 | 55,017 | -12,56 | 2884 |
|  | INCAL_DS09 | 55,133 | -12,883 | 2897 |
|  | SMBA_ES10 | 56,617 | -11,067 | 2540 |
|  | SMBA_ES118 | 54,65 | -12,233 | 2910 |
|  | SMBA_ES12 | 56,817 | -10,25 | 2076 |
|  | SMBA_ES129 | 54,65 | -12,283 | 2900 |
|  | SMBA_ES135 | 54,65 | -12,267 | 2900 |
|  | SMBA_ES137 | 54,567 | -12,317 | 2900 |
|  | SMBA_ES140 | 54,667 | -12,267 | 2912 |
|  | SMBA_ES143 | 54,683 | -12,233 | 2892 |
|  | SMBA_ES147 | 54,6 | -12,317 | 2921 |
|  | SMBA_ES152 | 54,7 | -12,333 | 2900 |
|  | SMBA_ES164 | 54,617 | -12,367 | 2925 |
|  | SMBA_ES172 | 54,65 | -12,283 | 2910 |
|  | SMBA_ES180 | 54,7 | -12,192 | 2886 |
|  | SMBA_ES185 | 54,733 | -12,25 | 2876 |
|  | SMBA_ES190 | 54,683 | -12,3 | 2898 |
|  | SMBA_ES197 | 57,35 | -10,483 | 2200 |
|  | SMBA_ES204 | 54,667 | -12,333 | 2904 |
|  | SMBA_ES207 | 54,667 | -12,183 | 2906 |
|  | SMBA_ES218 | 57,367 | -10,4 | 2175 |
|  | SMBA_ES231 | 54,7 | -12,2 | 2898 |
|  | SMBA_ES244 | 57,383 | -10,333 | 2150 |
|  | SMBA_ES266 | 56,4 | -11,983 | 2591 |
|  | SMBA_ES27 | 54,667 | -12,267 | 2880 |
|  | SMBA_ES283 | 54,65 | -12,25 | 2946 |
|  | SMBA_ES285 | 54,65 | -12,233 | 2906 |
|  | SMBA_ES289 | 57,317 | -10,417 | 2190 |
|  | SMBA_ES34 | 56,6 | -11,5 | 2515 |
|  | SMBA_ES56 | 54,667 | -12,267 | 2886 |
|  | SMBA_ES59 | 54,667 | -12,333 | 2900 |
|  | SMBA_ES6 | 55,05 | -12,483 | 2900 |
| **West European Slope (WES)** | AFEN 1996_53726#1 | 60,6245 | -3,583 | 530 |
|  | AFEN 1998_54601#1/2 | 58,914 | -8,6675 | 1583 |
|  | AFEN 1998_54616#2A/B | 58,8845 | -8,064 | 1195 |
|  | DISCOVERY_10111#8 | 49,558 | -13,098 | 1635 |
|  | DISCOVERY_9753#7 | 50,908 | -12,182 | 1942 |
|  | SMBA_SBC155 | 48,45 | -10,333 | 1330 |
|  | SMBA_SBC66 | 56,65 | -9,4 | 1200 |
|  | THALASSA 71_X305 | 44,083 | -5 | 463 |
|  | THALASSA 73_Z431 | 48,633 | -9,783 | 800 |
|  | THALASSA 73_Z435 | 48,833 | -9,883 | 1050 |
|  | THALASSA 73_Z453 | 48,55 | -10,833 | 2023 |
|  | THALASSA 73_Z459 | 48,617 | -9,883 | 1180 |
| **Iceland-Faroe Ridge (IFR)** | BIOFAR_264 | 62,14 | -9,982 | 780 |
|  | BIOICE_3025 | 63,5386 | -12,4405 | 563 |
|  | BIOFAR_424 | 62,837 | -9,573 | 509 |
|  | BIOICE_2003 | 65,34892 | -13,6040835 | 66 |
|  | BIOICE_2363 | 64,483 | -10,433 | 493 |
|  | BIOICE_2364 | 64,583 | -10,05 | 605 |
| **Iceland Basin (IcB)** | BIOICE_2288 | 62,37 | -22,678 | 1390 |
|  | BIOICE_2331 | 62,917 | -12,217 | 563 |
|  | BIOICE_2410 | 62,86 | -21,735 | 1074 |
|  | BIOICE_2853 | 62,6717 | -16,97 | 1840 |
|  | BIOICE_2859 | 61,837165 | -16,8875 | 2271 |
|  | BIOICE_2860 | 61,7238 | -16,958 | 2296 |
|  | BIOICE_2863 | 61,1697 | -18,0428 | 2400 |
|  | BIOICE_3181 | 60,5274 | -26,4801 | 1552,5 |
|  | BIOICE_3263 | 62,318 | -19,3935 | 1680,5 |
|  | BIOICE_3515 | 62,22185 | -18,229 | 1332,5 |
|  | BIOICE_3538 | 62,23905 | -14,1335 | 1518 |
|  | DISCOVERY_7709#72 | 60,115 | -19,707 | 2656 |
|  | DISCOVERY_7709#73 | 60,118 | -19,506 | 2641 |
|  | DISCOVERY_7709#85 | 59,962 | -19,917 | 2708 |
|  | IceAGE_1010-1 | 62,55166 | -20,39516 | 1384,8 |
|  | IceAGE_1032-1 | 63,3085 | -23,15766 | 289,4 |
|  | IceAGE_963-1 | 60,0455 | -21,46766 | 2749,4 |
|  | IceAGE_967-1 | 60,04616 | -21,47566 | 2750,4 |
|  | IceAGE_983-1 | 60,35733 | -18,135666 | 2567,7 |
|  | BIOFAR_274 | 63,014 | -7,82 | 698 |
|  | BIOFAR_275 | 63,053 | -7,693 | 804 |
|  | BIOFAR_458 | 62,916 | -7,004 | 675 |
|  | BIOICE_2323 | 63,917 | -10,083 | 623 |
| **Porcupine-Biscay Abyssal (PBA)** | BIOGAS III_DS42 | 47,533 | -9,6 | 4104 |
|  | BIOGAS III_DS44 | 47,55 | -9,7 | 3992 |
|  | BIOGAS VI_DS76 | 47,583 | -9,55 | 4228 |
|  | BIOGAS VI_DS77 | 47,533 | -9,583 | 4240 |
|  | CHAIN 106_316 | 50,97 | -13,023 | 2191 |
|  | CHAIN 106_318 | 50,45 | -13,34 | 2506 |
|  | CHAIN 106_321 | 50,206 | -13,597 | 2879 |
|  | CHAIN 106_323 | 50,139 | -13,872 | 3347 |
|  | CHAIN 106_326 | 50,084 | -14,406 | 3859 |
|  | CHAIN 106_328 | 50,079 | -15,747 | 4431 |
|  | CHAIN 106_330 | 50,723 | -17,87 | 4632 |
|  | DISCOVERY_10112#3 | 50,325 | -13,44 | 2748 |
|  | DISCOVERY_50604#1 | 50,102 | -13,883 | 3520 |
|  | INCAL_CP08 | 50,25 | -13,233 | 2644 |
|  | INCAL_CP09 | 50,233 | -13,267 | 2691 |
|  | INCAL_CP10 | 48,433 | -15,15 | 4823 |
|  | INCAL_DS10 | 50,217 | -13,267 | 2719 |
|  | INCAL_DS13 | 46,033 | -10,283 | 4822 |
|  | INCAL_OS01 | 50,25 | -13,183 | 2634 |
|  | INCAL_OS04 | 46,05 | -10,183 | 4796 |
|  | INCAL_OS07 | 47,517 | -9,567 | 4249 |
|  | INCAL_WS01 | 50,317 | -13,1 | 2539 |
|  | INCAL_WS02 | 50,333 | -12,917 | 2505 |
|  | INCAL_WS03 | 48,317 | -15,367 | 4829 |
|  | INCAL_WS04 | 48,3 | -15,217 | 4829 |
|  | INCAL_WS05 | 46,05 | -10,233 | 4796 |
|  | INCAL_WS09 | 47,45 | -9,567 | 4277 |
|  | POLYGAS_DS20 | 47,6 | -8,667 | 4226 |
|  | POLYGAS_DS21 | 47,533 | -8,75 | 4190 |
|  | POLYGAS_DS22 | 47,567 | -9,633 | 4144 |
|  | SARSIA_Stn50 | 43,778 | -3,633 | 2379 |
|  | SMBA_SBC205 | 54,667 | -12,25 | 2906 |

**Table S2.** Genetic 18S p-distances among taxa used for analysis. Bold – the smallest and the largest p-distances.

|  | ICUL10010 *Sa. georgi* | ICUL2238 *Sa. georgi* | ICUL10117 *Br. kozakowskae* | ICUL10118 *Br. kozakowskae* | ICUL12663 *Br. kozakowskae* | ICUL2237 *J. bioice* | ICUL9828 *J. bioice* | ICUL10114 *Typhlotanais* sp. | ICUL8977 *Su. frenchae* | ICUL9005 *Su. carringtonae* | ICUL9627 *Su. carringtonae* | ICUL1119 *G. gudmunssoni* | ICUL7967 *Ba. roberti* | ICUL10108 *C. igae* | ICUL7900 *St. sirene* | ICUL7965 *Tm. genesis* | ICUL9817 *H. partykae* | ICUL10140 *Ty. proctagon* | ICUL8966 *'greenwichensis*' group | ICUL5004 *Pulcherella* sp. | LC633683 *Akanthophoreus* sp. |
| --- | --- | --- | --- | --- | --- | --- | --- | --- | --- | --- | --- | --- | --- | --- | --- | --- | --- | --- | --- | --- | --- |
| ICUL10010 *Sa. georgi* |  |  |  |  |  |  |  |  |  |  |  |  |  |  |  |  |  |  |  |  |  |
| ICUL2238 *Sa. georgi* | 0,005 ± 0,003 |  |  |  |  |  |  |  |  |  |  |  |  |  |  |  |  |  |  |  |  |
| ICUL10117 *Br. kozakowskae* | 0,037 ± 0,007 | 0,035 ± 0,007 |  |  |  |  |  |  |  |  |  |  |  |  |  |  |  |  |  |  |  |
| ICUL10118 *Br. kozakowskae* | 0,037 ± 0,007 | 0,035 ± 0,007 | 0,000 |  |  |  |  |  |  |  |  |  |  |  |  |  |  |  |  |  |  |
| ICUL12663 *Br. kozakowskae* | 0,038 ± 0,008 | 0,037 ± 0,007 | 0,000 | 0,000 |  |  |  |  |  |  |  |  |  |  |  |  |  |  |  |  |  |
| ICUL2237 *J. bioice* | 0,038 ± 0,007 | 0,037 ± 0,007 | 0,029 ± 0,006 | 0,029 ± 0,006 | 0,03 ± 0,007 |  |  |  |  |  |  |  |  |  |  |  |  |  |  |  |  |
| ICUL9828 *J. bioice* | 0,038 ± 0,007 | 0,037 ± 0,007 | 0,029 ± 0,006 | 0,029 ± 0,006 | 0,03 ± 0,007 | 0,000 |  |  |  |  |  |  |  |  |  |  |  |  |  |  |  |
| ICUL10114 *Typhlotanais* sp. | 0,039 ± 0,008 | 0,038 ± 0,007 | 0,042 ± 0,008 | 0,042 ± 0,008 | 0,044 ± 0,008 | 0,042 ± 0,008 | 0,042 + 0,008 |  |  |  |  |  |  |  |  |  |  |  |  |  |  |
| ICUL8977 *Su. frenchae* | 0,055 ± 0,009 | 0,054 ± 0,009 | 0,043 ± 0,008 | 0,043 ± 0,008 | 0,045 ± 0,008 | 0,051 ± 0,009 | 0,051 ± 0,009 | 0,019 ± 0,005 |  |  |  |  |  |  |  |  |  |  |  |  |  |
| ICUL9005 *Su. carringtonae* | 0,048 ± 0,008 | 0,046 ± 0,007 | 0,035 ± 0,007 | 0,035 ± 0,007 | 0,037 ± 0,008 | 0,043 ± 0,008 | 0,043 ± 0,008 | **0,011 ± 0,004** | 0,008 ± 0,003 |  |  |  |  |  |  |  |  |  |  |  |  |
| ICUL9627 *Su. carringtonae* | 0,048 ± 0,008 | 0,046 ± 0,008 | 0,035 ± 0,007 | 0,035 ± 0,007 | 0,037 ± 0,007 | 0,043 ± 0,008 | 0,043 ± 0,008 | **0,011 ± 0,004** | 0,008 ± 0,003 | 0,000 |  |  |  |  |  |  |  |  |  |  |  |
| ICUL1119 *G. gudmunssoni* | 0,049 ± 0,008 | 0,048 ± 0,008 | 0,045 ± 0,008 | 0,045 ± 0,008 | 0,046 ± 0,008 | 0,046 ± 0,008 | 0,046 ± 0,008 | 0,014 ± 0,005 | 0,022 ± 0,005 | 0,014 ± 0,004 | 0,014 ± 0,004 |  |  |  |  |  |  |  |  |  |  |
| ICUL7967 *Ba. roberti* | 0,057 ± 0,009 | 0,055 ± 0,009 | 0,069 ± 0,010 | 0,069 ± 0,010 | 0,072 ± 0,010 | 0,065 ± 0,010 | 0,065 ± 0,010 | 0,077 ± 0,011 | 0,086 ± 0,011 | 0,078 ± 0,010 | 0,078 ± 0,010 | 0,083 ± 0,011 |  |  |  |  |  |  |  |  |  |
| ICUL10108 *C. igae* | 0,055 ± 0,009 | 0,054 ± 0,009 | 0,052 ± 0,009 | 0,052 ± 0,009 | 0,055 ± 0,009 | 0,051 ± 0,009 | 0,051 ± 0,009 | 0,06 ± 0,010 | 0,065 ± 0,010 | 0,057 ± 0,010 | 0,057 ± 0,010 | 0,061 ± 0,010 | 0,068 ± 0,010 |  |  |  |  |  |  |  |  |
| ICUL7900 *St. sirene* | 0,091 ± 0,011 | 0,089 ± 0,011 | 0,074 ± 0,010 | 0,074 ± 0,010 | 0,077 ± 0,011 | 0,088 ± 0,011 | 0,088 ± 0,011 | 0,091 ± 0,011 | 0,088 ± 0,011 | 0,08 ± 0,010 | 0,08 ± 0,010 | 0,086 ± 0,011 | 0,078 ± 0,010 | 0,09 ± 0,011 |  |  |  |  |  |  |  |
| ICUL7965 *Tm. genesis* | 0,091 ± 0,011 | 0,089 ± 0,011 | 0,086 ± 0,011 | 0,086 ± 0,011 | 0,087 ± 0,011 | 0,086 ± 0,011 | 0,086 ± 0,011 | 0,091 ± 0,011 | 0,092 ± 0,011 | 0,085 ± 0,011 | 0,085 ± 0,011 | 0,091 ± 0,011 | 0,083 ± 0,011 | 0,082 ± 0,011 | 0,1 ± 0,011 |  |  |  |  |  |  |
| ICUL9817 *H. partykae* | 0,072 ± 0,010 | 0,071 ± 0,010 | 0,082 ± 0,011 | 0,082 ± 0,011 | **0,085 ± 0,011** | 0,072 ± 0,010 | 0,072 ± 0,010 | 0,077 ± 0,011 | **0,085 ± 0,011** | 0,077 ± 0,010 | 0,077 ± 0,010 | 0,083 ± 0,011 | 0,078 ± 0,011 | 0,079 ± 0,011 | 0,102 ± 0,012 | 0,059 ± 0,009 |  |  |  |  |  |
| ICUL10140 *Ty. proctagon* | 0,078 ± 0,011 | 0,08 ± 0,011 | 0,082 ± 0,011 | 0,082 ± 0,011 | 0,085± 0,011 | 0,085 ± 0,011 | 0,085± 0,011 | 0,074 ± 0,010 | 0,088 ± 0,011 | 0,08 ± 0,011 | 0,08 ± 0,011 | 0,08 ± 0,011 | 0,085 ± 0,011 | 0,074 ± 0,011 | 0,105 ± 0,012 | 0,096 ± 0,012 | 0,08 ± 0,010 |  |  |  |  |
| ICUL8966 *'greenwichensis*' group | 0,083 ± 0,011 | 0,082 ± 0,011 | 0,076 ± 0,010 | 0,076 ± 0,010 | 0,079 ± 0,011 | 0,072 ± 0,010 | 0,072 ± 0,010 | 0,085 ± 0,011 | 0,092 ± 0,012 | 0,086 ± 0,011 | 0,086 ± 0,011 | 0,092 ± 0,011 | 0,079 ± 0,010 | 0,074 ± 0,011 | 0,096 ± 0,012 | 0,088 ± 0,011 | 0,074 ± 0,010 | 0,054 ± 0,009 |  |  |  |
| ICUL5004 *Pulcherella* sp. | 0,083 ± 0,011 | 0,082± 0,011 | 0,088 ± 0,011 | 0,088 ± 0,011 | 0,09 ± 0,011 | 0,079 ± 0,011 | 0,079 ± 0,011 | 0,08 ± 0,011 | 0,088 ± 0,011 | 0,08 ± 0,011 | 0,08 ± 0,011 | 0,077 ± 0,011 | 0,068 ± 0,010 | 0,079 ± 0,011 | 0,09 ± 0,011 | 0,068 ± 0,010 | 0,063 ± 0,010 | 0,091 ± 0,011 | 0,09 ± 0,011 |  |  |
| LC633683 *Akanthophoreus* sp. | 0,217 ± 0,016 | 0,217 ± 0,016 | 0,211 ± 0,016 | 0,211 ± 0,016 | 0,22 ± 0,017 | 0,217 ± 0,016 | 0,217 ± 0,016 | 0,22 ± 0,016 | 0,217 ± 0,016 | 0,211 ± 0,016 | 0,211 ± 0,016 | 0,211 ± 0,016 | 0,214 ± 0,016 | 0,21 ± 0,016 | 0,209 ± 0,016 | 0,226 ± 0,017 | 0,225 ± 0,017 | 0,219± 0,016 | 0,216 ± 0,016 | 0,214 ± 0,017 |  |
| ICUL3575 *Paratyphlotanais* sp. | 0,142 ± 0,013 | 0,144 ± 0,014 | 0,142 ± 0,014 | 0,142 ± 0,014 | 0,148 ± 0,014 | 0,141 ± 0,014 | 0,141 ± 0,014 | 0,153 ± 0,014 | 0,157 ± 0,015 | 0,15 ± 0,014 | 0,15 ± 0,014 | 0,155 ± 0,014 | 0,1389 ± 0,014 | 0,149 ± 0,014 | 0,157 ± 0,014 | 0,157 ± 0,015 | 0,152 ± 0,014 | 0,141 ± 0,014 | 0,141 ± 0,014 | 0,143 ± 0,014 | 0,217 ± 0,017 |

**Table S3.** Environmental variables, total number of samples and samples with ‘stout-bodied’ typhlotanaid collected in distinguished regions.

**Figure S1.** The evolutionary tree of Typhlotanaidae species from the histone H3


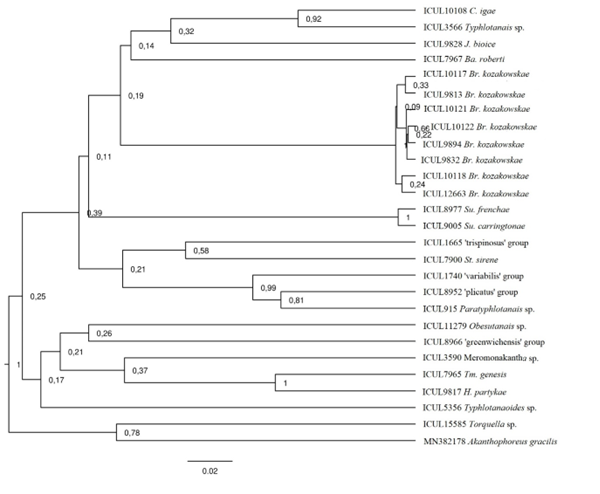


**Figure S2.** Pairwise correlation between six explanatory variables: depth, temperature (T), salinity (S), oxygen (O), nitrogen (N) and phosphorus (P).


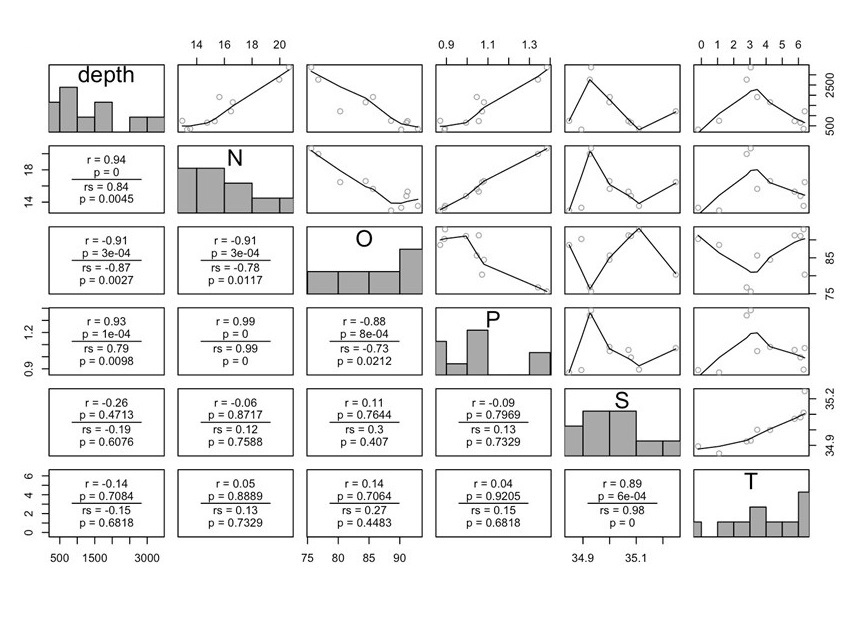

Supplement: Supplementary file 1 — Supplementary Information. [file 41598_2023_33616_MOESM1_ESM.docx]
